# Supplementary material for: Convergent insulin and TGF‐β signalling drives cancer cachexia by promoting aberrant fat body ECM accumulation in a Drosophila tumour model
Source: EMBO Rep. 2023 Nov 28;24(12):e57695. doi: 10.15252/embr.202357695 (PMC10702797; doi:10.15252/embr.202357695)
Supplement: Supplementary file 1 — Appendix S1 [file EMBR-24-e57695-s009.pdf]

Table of contents

Appendix Figure S1: Systemic ecdysone levels does not affect muscle detachment
Appendix Figure S2: Modulation of TGF-ß signalling does not affect insulin signalling in the muscle or wing imaginal discs
Appendix Figure S3: Pan-cancer expression of CHRD compared to BMP7.

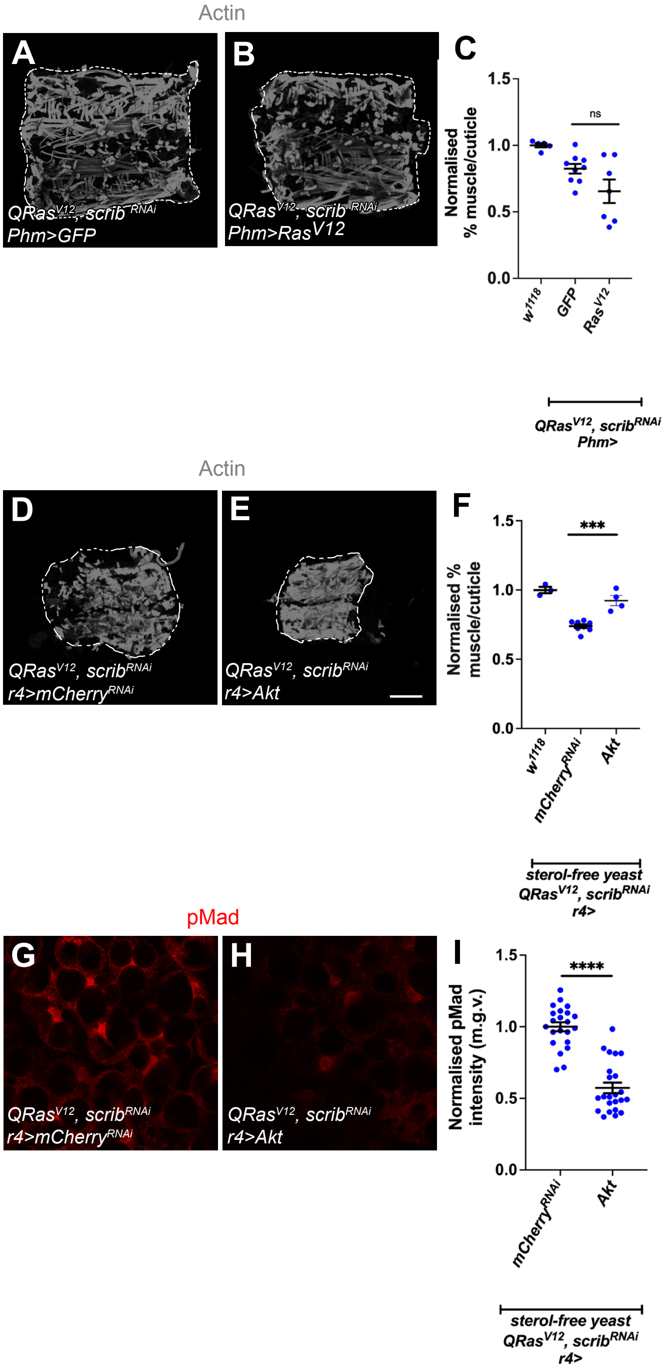

Appendix Figure S1: Systemic ecdysone levels does not affect muscle detachment

(A-B) Muscle fillets from animals where *GFP* or *Ras*^{V12} was expressed in the prothoracic gland (*Phm-GAL4*) of *QRas*^{V12}*scrib*^{RNAi} tumour-bearing animals, exhibited similar amount of muscle detachment.

(C) Quantification of muscle detachment in A-B. *w*^{1118} (n=5), *GFP* (n=9), *Ras*^{v12} (n=7).

(D-E) Muscle fillets stained with phalloidin (Actin) from animals where *mCherry*^{RNAi} or *UAS-Akt* was specifically expressed in the fat body (*r4-GAL4*) of *QRas*^{V12}*scrib*^{RNAi} tumour-bearing animals fed on a sterol-free diet.

(F) Quantification of muscle detachment in D-E. *mCherry*^{RNAi} (n=6), *UAS-Akt* (n=10)

(G-H) Fat body from animals expressing *mCherry*^{RNAi} or UAS-*Akt* under the control of *r4-GAL4* in *QRas*^{V12}*scrib*^{RNAi} tumour-bearing animals fed on a sterol-free diet stained with pMad.

(I) Quantification of normalized fat body pMad intensity in G-H. *mCherry* ^{RNAi} (n=21), *UAS-Akt* (n=23).

Scale bar is 500µm for muscle fillets and 50µm for fat body staining.

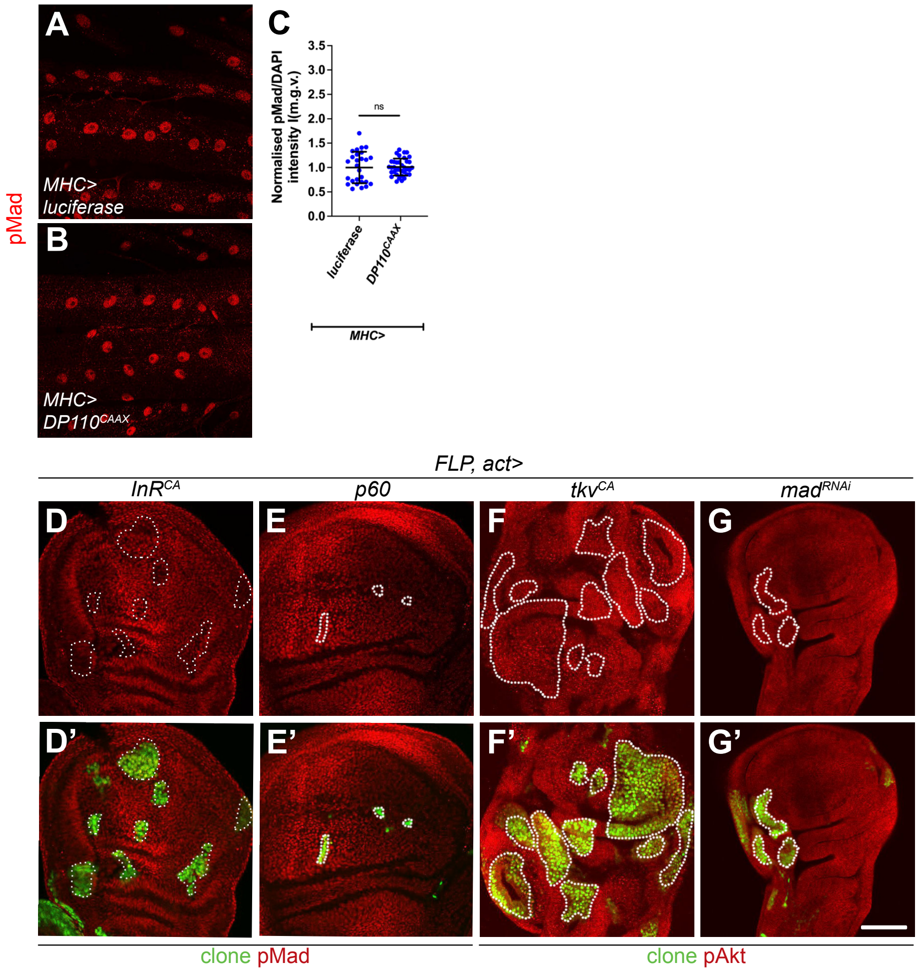

Appendix Figure S2: Modulation of TGF-ß signalling does not affect insulin signalling in the muscle or wing imaginal discs

(A-B) pMad staining in muscle fillets from larvae expressing *luciferase* or *DP110*^{CAAX} with *MHC-GAL4*.

(C) Quantification of pMad staining in A-B. *luciferase* (n=27), *DP110*^{CAAX} (n=36).

(D-D’) Heatshock induced flip-out clones (marked with GFP, circled with dotted lines) of *InR*^{CA} in the wing imaginal discs, readout of TGF-ß signalling levels indicated by pMad is not altered in the clones vs. surrounding tissues.

(E-E’) Heatshock induced flip-out clones (marked with GFP, circled with dotted lines) of *p60* in the wing imaginal discs, readout of TGF-ß signalling levels indicated by pMad is not altered in the clones vs. surrounding tissues.

(F-F’) Heatshock induced flip-out clones (marked with GFP, circled with dotted lines) of *tkv*^{CA} in the wing imaginal discs, readout of insulin signalling levels indicated by pAkt is not altered in the clones vs. surrounding tissues.

(G-G’) Heatshock induced flip out clones (marked with GFP, circled with dotted lines) of *mad*^{RNAi} in the wing imaginal discs, readout of insulin signalling levels indicated by pAkt is not altered in the clones vs. surrounding tissues.

Scale bar is 50 µm.

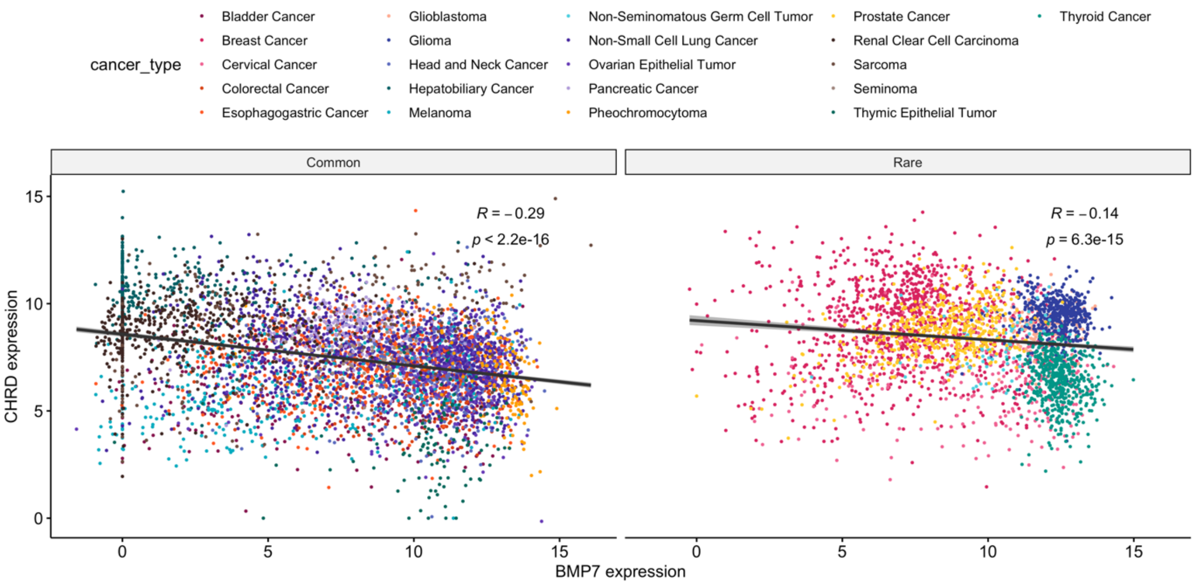

Appendix Figure S3: **Pan-cancer expression of CHRD compared to BMP7.** Data is RSEM batch normalized log2(value + 1) as per cBioPortal, points coloured by cancer type. Common cancers associated with cachexia include, bladder, colorectal, esophagogastric, Head and Neck, Hepatobiliary, Melanoma, Non-small cell lung cancer, Ovarian epithelial tumour, Pancreatic cancer, Pheochromocytoma, Renal Clear Cell carcinoma, Carcoma, Seminoma and Thymic epithelial tumour. Cancers rarely associated with cachexia include, Breast, Glioblastoma, Glioma, Seminomatous Germ cell Tumour, Prostate, Cervical and Thyroid. The date analyses 5441 samples from cancers where cachexia is common, 3127 from rare, and 841 of unknown. Spearman correlation R and p-values shown. Regression line is shown in black.
