# Supplementary material for: Convergent insulin and TGF‐β signalling drives cancer cachexia by promoting aberrant fat body ECM accumulation in a Drosophila tumour model
Source: EMBO Rep. 2023 Nov 28;24(12):e57695. doi: 10.15252/embr.202357695 (PMC10702797; doi:10.15252/embr.202357695)
Supplement: Supplementary file 2 — Expanded View Figures PDF [file EMBR-24-e57695-s004.pdf]

Expanded View Figures

Figure EV1. Tumour secreted Gbb and ImpL2 rescue muscle integrity additively.

A–D Muscle fillets of *Ras^{V12}dlg1^{RNAi}* tumour-bearing animals that express in the tumour *lacZ^{RNAi}*; *mCherry^{RNAi}* or *ImpL2^{RNAi}*; *lacZ^{RNAi}*, or *gbb^{RNAi}*; *mCherry^{RNAi}* or *ImpL2^{RNAi}*; *gbb^{RNAi}*.

E Quantification of muscle detachment in (A–D). *lacZ^{RNAi}*; *mCherry^{RNAi}* (n = 8), *ImpL2^{RNAi}*; *lacZ^{RNAi}* (n = 11), *gbb^{RNAi}*; *mCherry^{RNAi}* (n = 8), *ImpL2^{RNAi}*; *gbb^{RNAi}* (n = 16).

F, G OPP staining detecting protein translation in the fat body of *ω^{1118}* and *Ras^{V12}dlg^{RNAi}* tumour-bearing animals.

H Quantification of OPP in (F, G). *ω^{1118}* (n = 19) *Ras^{V12}dlg1^{RNAi}* (n = 26)

I–L OPP staining detecting protein translation in the fat body of *Ras^{V12}dlg1^{RNAi}* tumour-bearing animals, that express in the tumour *lacZ^{RNAi}*; *mCherry^{RNAi}* or *ImpL2^{RNAi}*; *lacZ^{RNAi}*, or *gbb^{RNAi}*; *mCherry^{RNAi}* or *ImpL2^{RNAi}*; *gbb^{RNAi}*.

M Quantification of OPP in (I–L). *ω^{1118}* (n = 10); *LacZ^{RNAi}*; *mCherry^{RNAi}* (n = 22), *ImpL2^{RNAi}*; *lacZ^{RNAi}* (n = 13), *gbb^{RNAi}*; *mCherry^{RNAi}* (n = 15), *ImpL2^{RNAi}*; *gbb^{RNAi}* (n = 14).

N, O Nidogen staining detecting ECM localisation in the fat body of *ω^{1118}* and *Ras^{V12}dlg1^{RNAi}* animals.

P Quantification of nidogen staining in (N, O). *ω^{1118}* (n = 23) *Ras^{V12}dlg1^{RNAi}* (n = 20).

Q–T *Ras^{V12}dlg1^{RNAi}* tumour-bearing animals that express *lacZ^{RNAi}*; *mCherry^{RNAi}* or *ImpL2^{RNAi}*; *lacZ^{RNAi}*, or *gbb^{RNAi}*; *mCherry^{RNAi}* or *ImpL2^{RNAi}*; *gbb^{RNAi}*.

U Quantification of Nidogen in (Q, T). *ω^{1118}* (n = 9); *LacZ^{RNAi}*; *mCherry^{RNAi}* (n = 17), *ImpL2^{RNAi}*; *lacZ^{RNAi}* (n = 22), *gbb^{RNAi}*; *mCherry^{RNAi}* (n = 9), *ImpL2^{RNAi}*; *gbb^{RNAi}* (n = 9).

Data information: Scale bar is 50 μm for fat body staining (F, G, I–L, N, O, Q–T) and 500 μm for muscle fillets (A–D). Graphs are represented as Mean ± SEM, n = the number of samples. (*) $P < 0.05$ (**) $P < 0.01$, (***) $P < 0.001$, (****) $P < 0.0001$. For experiments with two genotypes, two-tailed unpaired student's t-tests were used to test for significant differences. The Welch's correction was applied in cases of unequal variances. For experiments with more than two genotypes, significant differences between specific genotypes were tested using a one-way ANOVA and a subsequent Šidák *post-hoc* test.

© 2023 The Authors

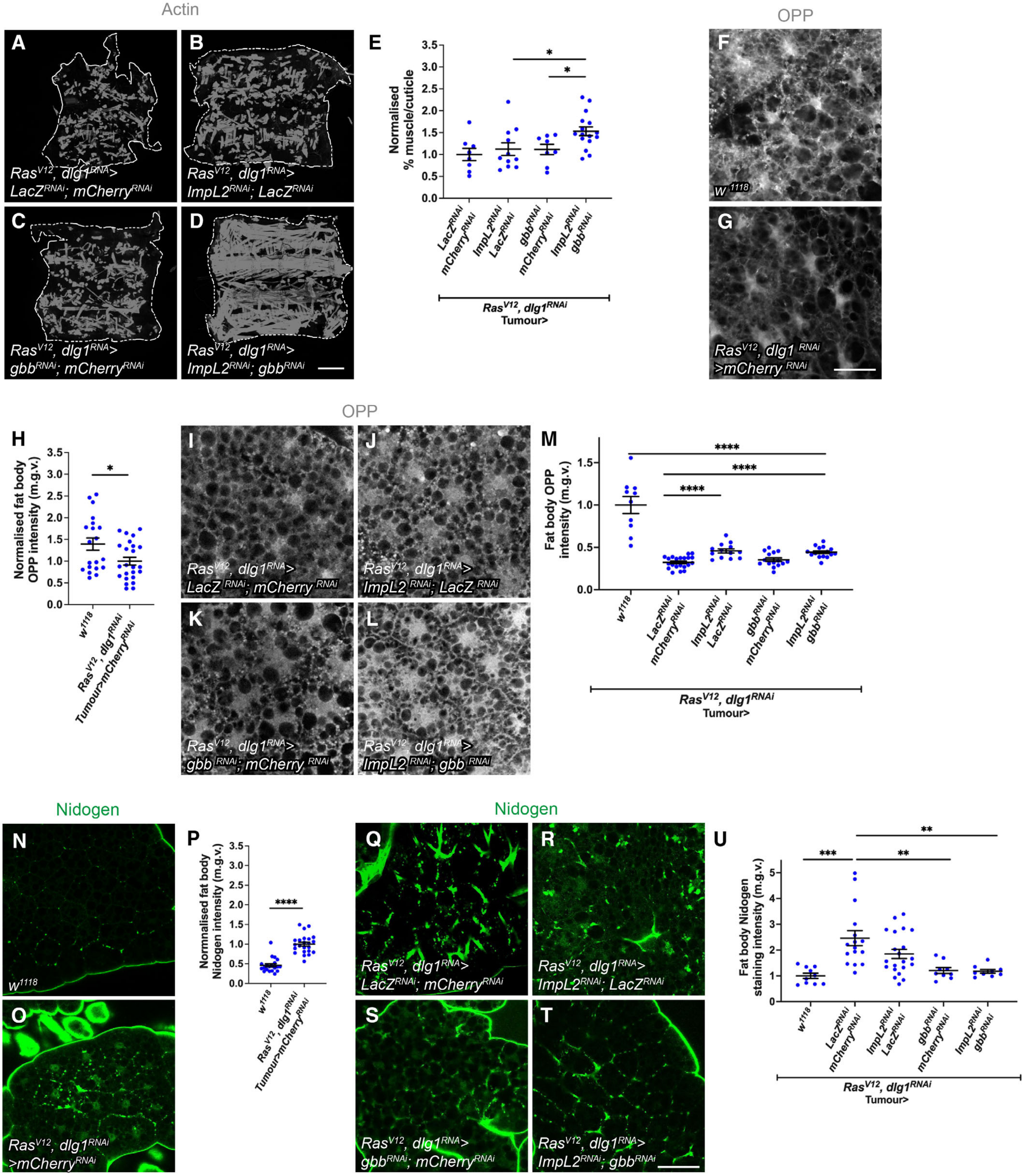

Figure EV1.

 © 2023 The Authors

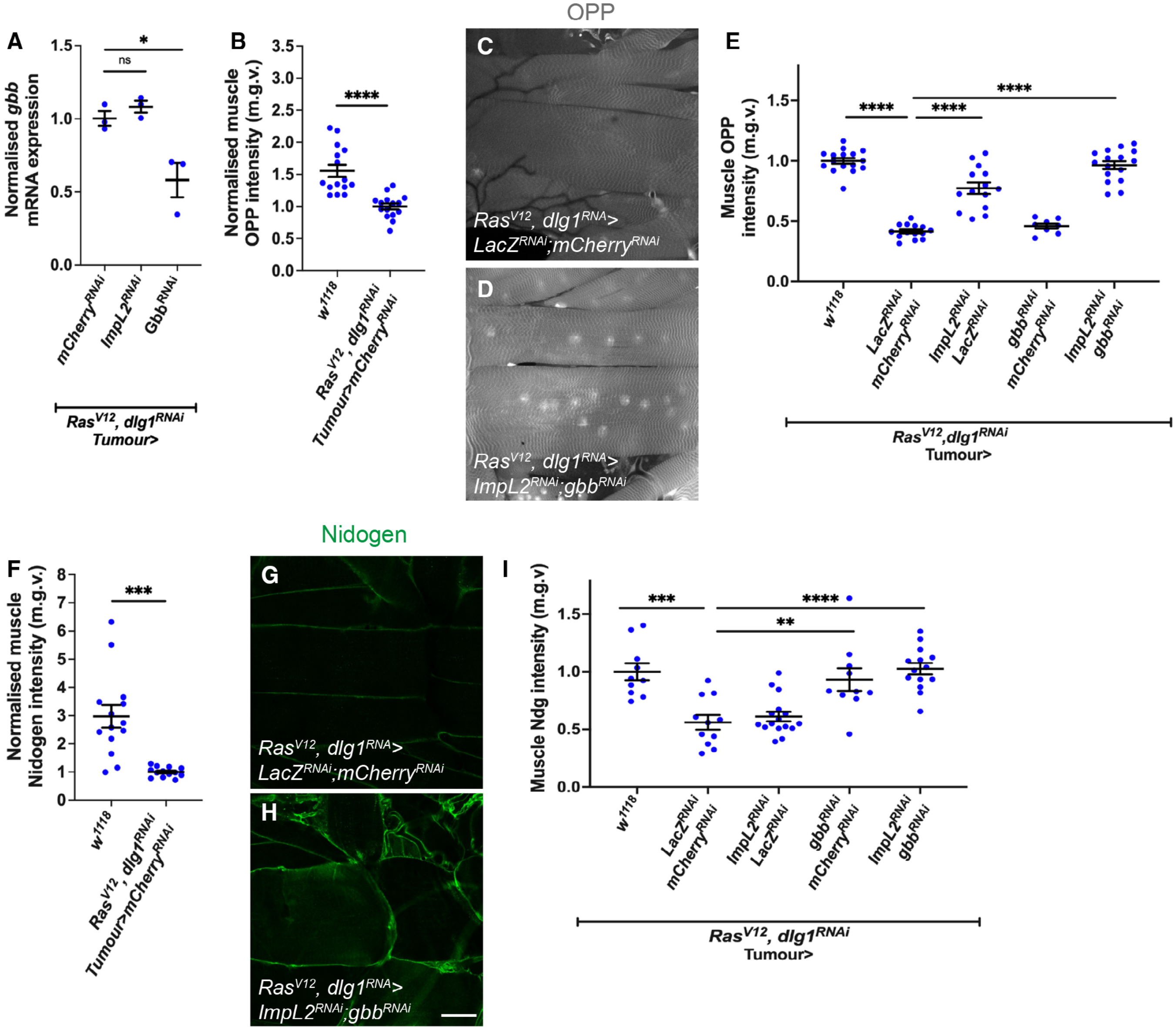

Figure EV2. Knockdown of tumour-derived Gbb and ImpL2 rescues muscle OPP and ECM protein Nidogen.

A Tumour qPCRs showing mRNA expression levels of *gbb* in *Ras*^{V12}*dlg1*^{RNAi} larvae expressing *mCherry*^{RNAi}, *ImpL2*^{RNAi}, *Gbb*^{RNAi} in the tumour ($n = 3$).

B Quantification of normalised muscle OPP intensity in ω^{1118} and *Ras*^{V12}*dlg1*^{RNAi} tumour-bearing animals, where *mCherry*^{RNAi} is specifically expressed in the tumour. ω^{1118} ($n = 15$) and *Ras*^{V12}*dlg1*^{RNAi} ($n = 15$).

C, D OPP staining detecting protein translation in the muscles of *Ras*^{V12}*dlg1*^{RNAi} tumour-bearing animals that express *lacZ*^{RNAi}; *mCherry*^{RNAi} or *ImpL2*^{RNAi}; *gbb*^{RNAi}.

E Quantification of OPP in the muscles of wild type ω^{1118} ($n = 18$) and tumour-bearing animals expressing *LacZ*^{RNAi};*mCherry*^{RNAi} ($n = 16$), *ImpL2*^{RNAi}; *lacZ*^{RNAi} ($n = 14$), *gbb*^{RNAi}; *mCherry*^{RNAi} ($n = 8$), *ImpL2*^{RNAi}; *gbb*^{RNAi} ($n = 15$).

F Quantification of normalised muscle Nidogen intensity in ω^{1118} and *Ras*^{V12}*dlg1*^{RNAi} tumour-bearing animals, where *mcherry*^{RNAi} is specifically expressed in the tumour. ω^{1118} ($n = 14$) and *Ras*^{V12}*dlg1*^{RNAi} ($n = 13$)

G, H Nidogen staining detecting ECM localisation in the muscles of *Ras*^{V12}*dlg1*^{RNAi} tumour-bearing animals that express *lacZ*^{RNAi}; *mCherry*^{RNAi} or *ImpL2*^{RNAi};*gbb*^{RNAi}.

I Quantification of Nidogen in wild type ω^{1118} ($n = 10$) and tumour-bearing animals expressing *LacZ*^{RNAi};*mCherry*^{RNAi} ($n = 11$), *ImpL2*^{RNAi}; *lacZ*^{RNAi} ($n = 16$), *gbb*^{RNAi}; *mCherry*^{RNAi} ($n = 10$), *ImpL2*^{RNAi}; *gbb*^{RNAi} ($n = 14$).

Data information: Scale bar is 50 μm. Graphs are represented as Mean ± SEM, n = the number of samples. (*) $P < 0.05$ (**) $P < 0.01$, (***) $P < 0.001$, (****) $P < 0.0001$, (ns) $P > 0.05$. For experiments with two genotypes, two-tailed unpaired student's *t*-tests were used to test for significant differences. The Welch's correction was applied in cases of unequal variances. For experiments with more than two genotypes, significant differences between specific genotypes were tested using a one-way ANOVA and a subsequent Šidák *post-hoc* test.

© 2023 The Authors

Figure EV3. Muscle overexpression of *Akt* (but not fat body *Akt* or *mad RNAi* overexpression) rescues atrophy in cachectic animals, and TGF-ß signalling in the muscle is responsive to modulation in tumour insulin signalling but is not required for muscle integrity in cachexia.

A, B Muscle fillets stained with phalloidin (Actin) where *luciferase* or *Akt* was expressed under the control of *MHC-GAL4* in $QRas^{V12}scrib^{RNAi}$ tumour-bearing animals.
C Quantification of normalised muscle detachment in (A, B). Wilt type control ($MHC>mCherry^{RNAi}$, n = 3), *UAS-luciferase* (n = 10), *Akt* (n = 17).
D, E Muscle segment (outlined) where *luciferase* or *Akt* was expressed under the control of *MHC-GAL4* in $QRas^{V12}scrib^{RNAi}$ tumour-bearing animals.
F Quantification of normalised muscle width/length in (D, E). Wild type control (n = 15), *luciferase* (n = 9), *Akt* (n = 16).
G, H Muscle Nidogen staining where *luciferase* or *Akt* was expressed under the control of *MHC-GAL4* in $QRas^{V12}scrib^{RNAi}$ tumour-bearing animals.
I Quantification of normalised muscle Nidogen staining in (G, H). *luciferase* (n = 16), *Akt* (n = 15).
J, K Muscle segment from $QRas^{V12}scrib^{RNAi}$ tumour-bearing animals, where $mCherry^{RNAi}$ or *Akt* was expressed in the fat body (*r4-GAL4*).
L Quantification of normalised muscle width/length in (J, K). $r4>mCherry^{RNAi}$(n = 9), $QRas^{V12}scrib^{RNAi}$;$r4>mCherry^{RNAi}$ (n = 5), $QRas^{V12}scrib^{RNAi}$;$r4>Akt$ (n = 8).
M, N Muscle segment from $QRas^{V12}scrib^{RNAi}$ tumour-bearing animals, where $mCherry^{RNAi}$ or mad^{RNAi} was expressed in the fat body (*r4-GAL4*).
O Quantification of normalised muscle width/length in (M, N). $r4>mCherry^{RNAi}$(n = 11), $QRas^{V12}scrib^{RNAi}$; $r4>mCherry^{RNAi}$ (n = 18), $QRas^{V12}scrib^{RNAi}$; $r4>mad^{RNAi}$ (n = 15).
P Quantification of normalised muscle OPP intensity where *luciferase* (n = 10) or *Akt* (n = 16) was expressed under the control of *MHC-GAL4* in $QRas^{V12}scrib^{RNAi}$ tumour-bearing animals.
Q, R Muscle from $Ras^{V12}dlg1^{RNAi}$ tumour-bearing animals expressing either $mCherry^{RNAi}$ or $ImpL2^{RNAi}$ in the tumour, where TGF-ß signalling activation is indicated by pMad staining.
S Quantification of normalised muscle pMad intensity in (Q, R). w^{1118} (n = 30), $mCherry^{RNAi}$ (n = 30), $ImpL2^{RNAi}$ (n = 26).
T Quantification of normalised muscle detachment where $MHC>mCherry^{RNAi}$ (n = 5) or $mCherry^{RNAi}$ (n = 9) or $lacZ^{RNAi}$; mad^{RNAi} (n = 6) was expressed under the control of *MHC-GAL4* in $QRas^{V12}scrib^{RNAi}$ tumour-bearing animals.

Data information: Scale bar is 500 μm for muscle fillets (A, B), 100 μm for muscle segment atrophy measurements (D, E, J, K, M, N) and 50 μm for muscle Nidogen and pMad staining (G, H, Q, R). Muscles in tumour bearing animals were dissected at day 6 after tumour induction. Graphs are represented as Mean ± SEM, n = the number of samples. (*) $P < 0.05$ (**) $P < 0.01$, (***) $P < 0.001$, (****) $P < 0.0001$, (ns) $P > 0.05$. For experiments with two genotypes, two-tailed unpaired student's *t*-tests were used to test for significant differences. The Welch's correction was applied in cases of unequal variances. For experiments with more than two genotypes, significant differences between specific genotypes were tested using a one-way ANOVA and a subsequent Šidák *post-hoc* test.

© 2023 The Authors

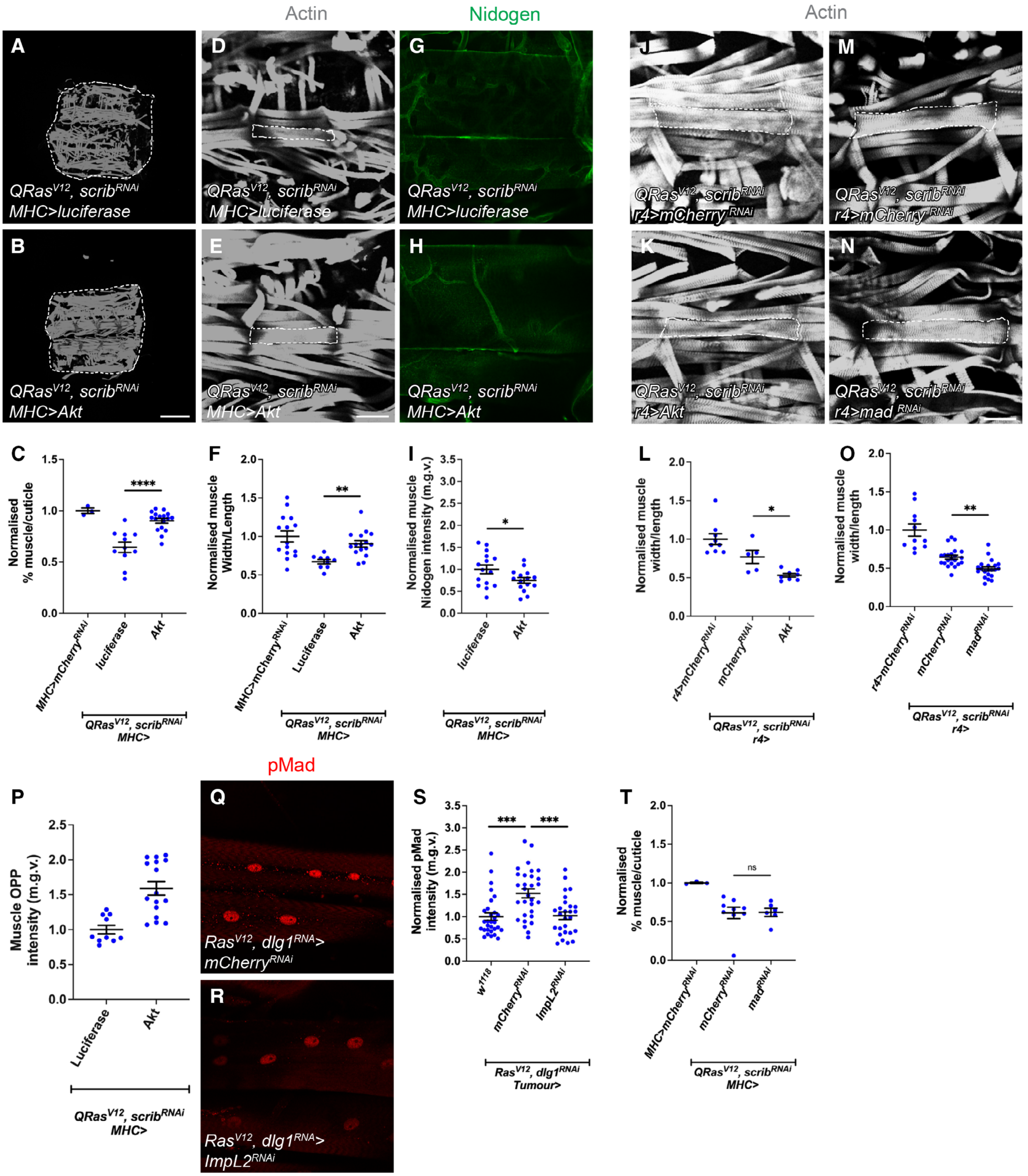
Actin
Nidogen
Actin
A
QRasV12, scribRNAi MHC>luciferase
B
QRasV12, scribRNAi MHC>Akt
D
QRasV12, scribRNAi MHC>luciferase
E
QRasV12, scribRNAi MHC>Akt
G
QRasV12, scribRNAi MHC>luciferase
H
QRasV12, scribRNAi MHC>Akt
J
QRasV12, scribRNAi r4>mCherry RNAi
M
QRasV12, scribRNAi r4>mCherry RNAi
K
QRasV12, scribRNAi r4>Akt
N
QRasV12, scribRNAi r4>mad RNAi
C
Normalised % muscle/cuticle
MHC>mCherryRNAi
luciferase
Akt
QRasV12, scribRNAi MHC>
F
Normalised muscle Width/Length
MHC>mCherryRNAi
Luciferase
Akt
QRasV12, scribRNAi MHC>
I
Normalised muscle Nidogen intensity (m.g.v.)
luciferase
Akt
QRasV12, scribRNAi MHC>
L
Normalised muscle width/length
r4>mCherryRNAi
mCherryRNAi
Akt
QRasV12, scribRNAi r4>
O
Normalised muscle width/length
r4>mCherryRNAi
mCherryRNAi
madRNAi
QRasV12, scribRNAi r4>
P
Muscle OPP intensity (m.g.v.)
Luciferase
Akt
QRasV12, scribRNAi MHC>
pMad
Q
RasV12, dlg1RNAi> mCherryRNAi
R
RasV12, dlg1RNAi> ImpL2RNAi
S
Normalised pMad intensity (m.g.v.)
w1118
mCherryRNAi
ImpL2RNAi
RasV12, dlg1RNAi Tumour>
T
Normalised % muscle/cuticle
ns
MHC>mCherryRNAi
mCherryRNAi
madRNAi
QRasV12, scribRNAi MHC>

Figure EV3.

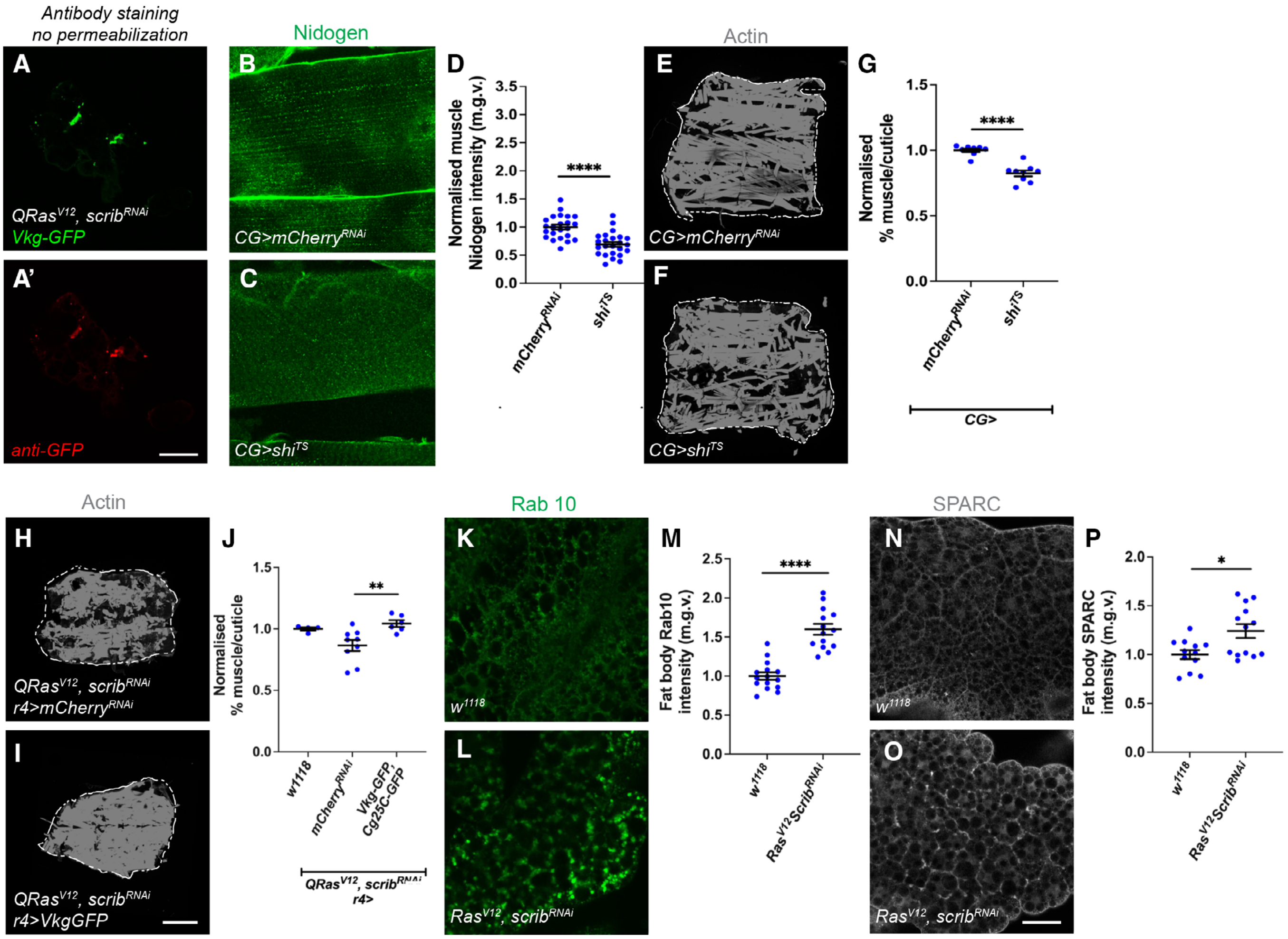

Figure EV4. Inhibition of fat body endocytosis causes muscle detachment, increasing Vkg expression in the fat body improves muscle integrity in tumour-bearing animals.

- A, A′ Antibody staining against GFP without permeabilisation can detect Vkg-GFP.
- B, C Nidogen staining in muscles of animals where *mCherry*^{RNAi} or *shi*^{TS} was expressed in the fat body via *CG-GAL4*.
- D Quantification of muscle nidogen staining in (B, C). *mCherry*^{RNAi} (n = 23), *shi*^{TS} (n = 24).
- E, F Muscle fillets stained with phalloidin (Actin) from animals where *mCherry*^{RNAi} or *shi*^{TS} was expressed in the fat body via *CG-GAL4*.
- G Quantification of normalised muscle detachment in (E, F). *mCherry*^{RNAi} (n = 8), *shi*^{TS} (n = 9).
- H, I Muscle fillets stained with phalloidin (Actin) from *QRas*^{V12}*scrib*^{RNAi} tumour-bearing animals, where *mCherry*^{RNAi} or *UAS-VkgGFP;UAS-Cg25C-GFP* was expressed in the fat body (*r4-GAL4*). Day 6 muscles used here.
- J Quantification of normalised muscle detachment in (H, I). ω^{1118} (n = 4), *QRas*^{V12}*scrib*^{RNAi}*;r4>mCherry*^{RNAi} (n = 9), *QRas*^{V12}*scrib*^{RNAi}*;r4>UAS-VkgGFP;UAS-Cg25C-GFP* (n = 6).
- K, L Fat body staining for Rab10 in ω^{1118} and QRas^{V12}scrib^{RNAi} tumour-bearing animals.
- M Quantification of normalised Rab10 levels in (K, L). ω^{1118} (n = 15), *QRas*^{V12}*scrib*^{RNAi} (n = 14).
- N, O Fat body staining for SPARC in ω^{1118} and QRas^{V12}scrib^{RNAi} tumour-bearing animals.
- P Quantification of normalised SPARC levels in (N, O). ω^{1118} (n = 12), *QRas*^{V12}*scrib*^{RNAi} (n = 13).

Data information: Scale bar is 500 µm for muscle fillets (E, F, H, I) 50 µm for muscle Nidogen and GFP staining (A, A′, B, C) and 50 µm for fat body staining (K, L, N, O). Muscles in *Shi*^{TS} animals were dissected at day 5 ALH, muscles and fat body in tumour bearing animals were dissected at day 6 after tumour induction. Graphs are represented as Mean ± SEM, n = the number of samples. (*) $P < 0.05$ (**) $P < 0.01$, (****) $P < 0.0001$. For experiments with two genotypes, two-tailed unpaired student's t-tests were used to test for significant differences. The Welch's correction was applied in cases of unequal variances. For experiments with more than two genotypes, significant differences between specific genotypes were tested using a one-way ANOVA and a subsequent Šidák *post-hoc* test.

 © 2023 The Authors

Figure EV5. Modulating fat body TGF-ß signalling alters sparc and rab10 transcription, and modulating fat body InR alters sog transcription.

A Knockdown efficiency of *sog*^{RNAi} and *mad*^{RNAi} as indicated by qPCR and western blotting respectively. For qPCR, error bars represent SEM, n = 3 for biological replicates.

B Fat body qPCR showing normalised mRNA expression levels of SPARC and Rab10 upon the expression of *mCherry*^{RNAi} compared to *UAS-Mad*. Error bars represent SD, n = 3 for biological replicates.

C Fat body qPCRs showing normalised mRNA expression levels of TGF-ß receptors and ligands in *InR*^{CA} or *mCherry*^{RNAi} larvae (raised at 18°C) with *CG-GAL4* (n = 3, biological replicates). Error bars represent SD.

D Summary diagram—*Left*: during development, insulin signalling in the fat body activates the transcription of *sog*, which inhibits Gbb and prevents the activation of TGF-ß signalling in the fat body. This allows fat body ECM to be secreted to function in the muscle. Insulin signalling in the muscle in parallel enhances translation and muscle growth. *Right*: in tumour-bearing/cachectic animals, tumours secrete two ligands: ImpL2 and Gbb. In the fat body, ImpL2 inhibits insulin signalling, preventing the transcription of *sog* and thus Sog can no longer inhibit Gbb. In addition, tumour secreted Gbb binds to Tkv to activate TGF-ß signalling in the fat body, resulting in an accumulation of ECM proteins, to prevent ECM transport out of the fat body to reach the muscle. In the muscle, ImpL2 inhibits insulin signalling, which inhibits translation and muscle growth. Fat body in tumour bearing animals were dissected at day 6 after tumour induction. Fat body in non-tumour bearing animals were dissected at day 5 ALH.

Data information: (*) $P < 0.05$ (**) $P < 0.01$, (***) $P < 0.001$, (****) $P < 0.0001$. Two-tailed unpaired student's t-tests were used to test for significant differences. The Welch's correction was applied in cases of unequal variances.

© 2023 The Authors

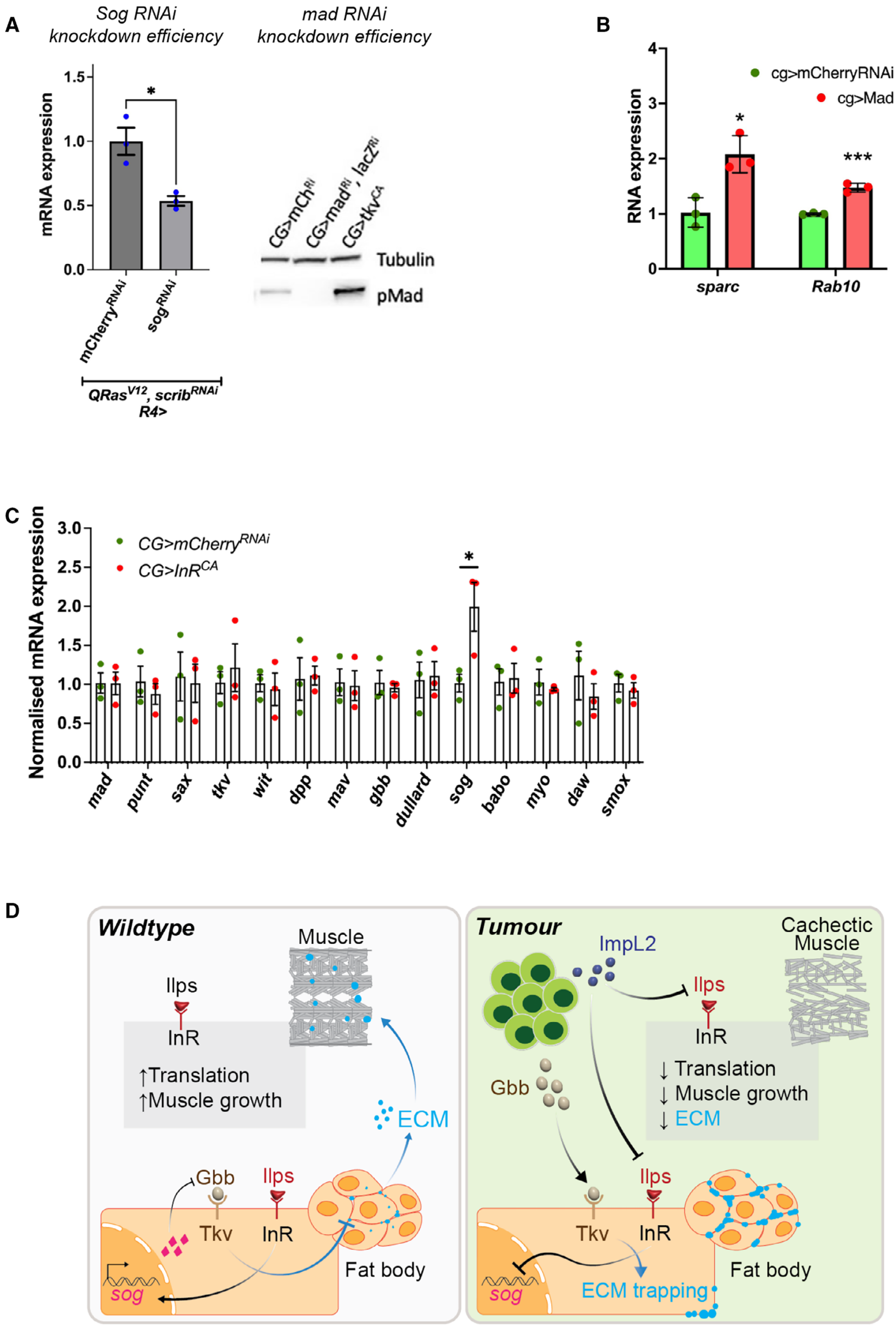

Figure EV5.

 © 2023 The Authors
