# Supplementary material for: Convergent insulin and TGF‐β signalling drives cancer cachexia by promoting aberrant fat body ECM accumulation in a Drosophila tumour model
Source: EMBO Rep. 2023 Nov 28;24(12):e57695. doi: 10.15252/embr.202357695 (PMC10702797; doi:10.15252/embr.202357695)
Supplement: Supplementary file 3 — PDF+ [file EMBR-24-e57695-s012.pdf]

Article

SOURCE DATA TRANSPARENT PROCESS OPEN ACCESS

EMBO reports

Convergent insulin and TGF-β signalling drives cancer cachexia by promoting aberrant fat body ECM accumulation in a *Drosophila* tumour model

Daniel Bakopoulos[1,2], Sofya Golenkina[1], Callum Dark[1,2], Elizabeth L Christie[1,2], Besaiz J Sánchez-Sánchez[3], Brian M Stramer[3] & Louise Y Cheng[1,2,4,*]

Abstract

In this study, we found that in the adipose tissue of wildtype animals, insulin and TGF-β signalling converge via a BMP antagonist short gastrulation (sog) to regulate ECM remodelling. In tumour bearing animals, Sog also modulates TGF-β signalling to regulate ECM accumulation in the fat body. TGF-β signalling causes ECM retention in the fat body and subsequently depletes muscles of fat body-derived ECM proteins. Activation of insulin signalling, inhibition of TGF-β signalling, or modulation of ECM levels via SPARC, Rab10 or Collagen IV in the fat body, is able to rescue tissue wasting in the presence of tumour. Together, our study highlights the importance of adipose ECM remodelling in the context of cancer cachexia.

Keywords cachexia; *Drosophila*; ECM; insulin; TGF-β
Subject Categories Cancer; Metabolism; Signal Transduction
DOI 10.15252/embr.202357695 | Received 21 June 2023 | Revised 16 October 2023 | Accepted 26 October 2023
EMBO Reports (2023) e57695

Introduction

Cachexia is a metabolic wasting syndrome characterised by the loss of adipose tissue and muscle. Cachexia occurs in approximately 80% of advanced-stage cancer patients and is ultimately responsible for approximately 30% of cancer mortalities. Despite the clinical significance of cachexia in cancer and diseases such as HIV or other chronic diseases, there is no gold standard for its treatment (Baracos *et al*, 2018). Cachexia is often resolved when the tumour can be resected in early disease; however, there are few strategies to treat cachexia in late stage or metastatic patients, where removal of the tumour is not an option. Therefore, understanding the mechanisms that drive wasting in peripheral tissues and how to target these deregulations can offer new therapeutic avenues to treat cachexia.

Drosophila melanogaster is emerging as an excellent model to identify tumour-secreted factors that drive cancer cachexia. Adult and larval tumour models that induce cachectic phenotypes have been established in *Drosophila*, and these models have revealed several different mechanisms by which tumours induce these cachectic phenotypes (Figueroa-Clarevega & Bilder, 2015; Kwon *et al*, 2015; Song *et al*, 2019; Newton *et al*, 2020; Ding *et al*, 2021; Hodgson *et al*, 2021; Khezri *et al*, 2021; Lee *et al*, 2021; Lodge *et al*, 2021; Santabárbara-Ruiz & Léopold, 2021). We recently demonstrated that eye imaginal disc tumours, caused by the expression of constitutively activate *Ras* (Ras^{V12}) and an RNAi against the polarity protein disclarge 1 ($dlg1^{RNAi}$), secrete two cachectic factors: ImpL2 and Matrix metalloproteinase 1 (Mmp1) (Lodge *et al*, 2021). ImpL2 is a secreted protein that induces cachexia by binding to circulating Insulin-like peptides (Ilps) to prevent them from activating Insulin-like receptor (InR) in host tissues, thus causing host tissue wasting (Honegger *et al*, 2008). This mechanism is likely conserved, as Insulin Growth Factor Binding Protein-2 (IGFBP-2), which also antagonises insulin signalling, is correlated with muscle atrophy in pancreatic ductal adenocarcinoma (PDAC) patients (Dong *et al*, 2021). In addition, Mmp1 drives cachexia by increasing the amount of the Transforming growth factor-β (TGF-β) ligand Glass bottom boat (Gbb). Gbb subsequently induces an elevation of TGF-β signalling in the *Drosophila* adipose tissue (called the fat body), to induce muscle detachment. Together, our findings highlighted the importance of fat body signalling in promoting muscle degradation during cachexia. However, what occurs downstream of these signals to drive fat body and muscle disruption remains unclear.

In this study, we demonstrate that tumour-derived ImpL2 and Gbb facilitate muscle degradation during cachexia via two main mechanisms. Firstly, ImpL2 mediates a reduction in fat body insulin signalling, which in turn activates fat body TGF-β signalling by upregulating *short gastrulation* (*sog*), a TGF-β antagonist. As Gbb also activates fat body TGF-β signalling, our findings reveal that ImpL2 and Gbb converge on this activation. We further show that fat body TGF-β signalling activation subsequently causes an

1 Peter MacCallum Cancer Centre, Melbourne, Vic, Australia
2 Sir Peter MacCallum Department of Oncology, The University of Melbourne, Melbourne, Vic, Australia
3 Kings College London, Randall Centre for Cell & Molecular Biophysics, London, UK
4 Department of Anatomy and Physiology, The University of Melbourne, Melbourne, Vic, Australia
*Corresponding author. Tel: +61 450053363; E-mail: louise.cheng@petermac.org

© 2023 The Authors. Published under the terms of the CC BY 4.0 license.

aberrant upregulation of ECM proteins in the fat body. As muscle ECM proteins are mostly fat body-derived (Dai *et al*, 2017, 2018), this in turn causes a reduction in muscle ECM to promote muscle detachment. Modulating SPARC, a collagen binding protein (Shahab *et al*, 2015) or Rab10, a regulator of basement membrane fibril formation (Isabella & Horne-Badovinac, 2016) can ameliorate ECM accumulation in the fat body in tumour bearing animals, and in turn improve muscle integrity. Secondly, tumour secreted ImpL2 causes a reduction in muscle insulin signalling, which contributes towards reduced translation and increased muscle atrophy. Enhanced activation of insulin in the muscle can specifically improve muscle atrophy (but not ECM). Together, the two mechanisms attribute towards the muscle detachment phenotype we have observed. Therefore, our data demonstrate that in addition to targeting tumour secreted factors, modulating key signalling and ECM regulators in peripheral tissues could be an effective strategy to treat cachexia.

Results

Reduced insulin signalling promotes the activation of TGF-β signalling in the cachectic fat body

In this study, we utilise two *Drosophila* larval tumour models to study cachexia. In the first model, the tumour is induced via the *GAL4-UAS* mediated overexpression of Ras^{V12} and Discs Large 1 (*dlg1*) RNAi in the eye (Fig 1A). Using this system, we can knockdown or overexpress candidate genes in the tumour. In the second model, the tumour is induced via the *QF2-QUAS* mediated overexpression of Ras^{V12} and *scrib* RNAi (Fig 1A), allowing us to knockdown or overexpress genes of interest in the fat body of tumour-bearing animals using drivers such as *R4-GAL4*. We previously showed that Ras^{V12}, $dlg1^{RNAi}$ eye imaginal disc tumours express elevated levels of the dIlp antagonist *ImpL2*, consequently leading to a reduction in fat body insulin signalling (Lodge *et al*, 2021). In parallel, tumour secreted Gbb accounts for the upregulation of TGF-β signalling. Here, we show that tumours induced by the overexpression of Ras^{V12} and an RNAi against the polarity protein Scribble ($scrib^{RNAi}$) using the QF/QUAS system, caused a downregulation of insulin signalling (pAkt, Fig 1B–D), and an upregulation of TGF-β signalling (pMad, Fig 1E–G) in the fat body.

Tumour-specific ImpL2 inhibition was sufficient to elevate fat body pAkt levels (Lodge *et al*, 2021) and ameliorate tumour-induced muscle disruption (Honegger *et al*, 2008; Figueroa-Clarevega & Bilder, 2015; Kwon *et al*, 2015; Lee *et al*, 2021). Surprisingly, we found that the knockdown of ImpL2 in the tumour (Ras^{V12}, $dlg1^{RNAi}$) caused a reduction in fat body pMad staining (Fig 1H–J), suggesting that there may be signalling crosstalk between the insulin/PI3K and TGF-β pathways in the fat body. Furthermore, expression of *Akt* overexpression in the fat body of tumour animals ($QRas^{V12}$, $scrib^{RNAi}$), caused an upregulation of pAkt (Fig 2A–C), but an unexpected downregulation of the TGF-β signalling readout pMad (Fig 2D–F), indicating that an upregulation of insulin/PI3K signalling maybe correlated with a downregulation of TGF-β signalling. However, conversely, TGF-β signalling does not seem to affect the activation of PI3K signalling pathway. Upon the expression of the TGF-β inhibitor *sog* (Biehs *et al*, 1996) in the fat body (*r4-GAL4*) of tumour bearing animals ($QRas^{V12}$, $scrib^{RNAi}$), we observed a significant reduction in fat body pMad levels (Fig 2G–I) but no significant effects on pAkt levels (Fig 2J–L).

Enhancing insulin signalling in the fat body improves muscle integrity in cachectic animals

Next, we tested whether fat body-specific changes in Insulin signalling influences muscle breakdown during cachexia. Similar to *GAL4*-induced Ras^{V12}, $scrib^{RNAi}$ tumour, *QF2* induced Ras^{V12}, $scrib^{RNAi}$ tumours were able to induce a 40% reduction in muscle/cuticle attachment (Lodge *et al*, 2021; Dark *et al*, 2022) (Fig 3A–E). Upon overexpression of *Akt* in the fat body (*r4-GAL4*) of tumour bearing animals ($QRas^{V12}$, $scrib^{RNAi}$), we found there was a significant improvement in muscle morphology (Fig 3F–H). Similarly, overexpression of InR^{CA} in the fat body of tumour bearing animals ($QRas^{V12}$, $scrib^{RNAi}$) also improved muscle integrity (Fig 3I–K). Together, our data indicate that the elevation of fat body insulin signalling level can improve muscle integrity in the context of cachexia.

To test if fat body insulin signalling facilitates cachexia progression via TGF-β signalling, we next activated insulin signalling via fat body expression of InR^{CA} and simultaneously knocked down TGF-β signalling via fat body expression of mad^{RNAi} (Fig 3L–O) in tumour bearing animals ($QRas^{V12}$, $scrib^{RNAi}$). We have previously shown that mad^{RNAi} was sufficient to rescue muscle integrity (Lodge *et al*, 2021) and here found that InR^{CA} (InR^{CA};$mCherry^{RNAi}$) was similarly able to rescue muscle integrity (Fig 3I–K). Co-expression of InR^{CA} and mad^{RNAi} gave a similar rescue as either InR^{CA} (InR^{CA}; $mCherry^{RNAi}$) or mad^{RNAi} (mad^{RNAi};$LacZ^{RNAi}$) alone, suggesting that TGF-β signalling likely acts downstream of insulin signalling in the fat body of tumour-bearing animals.

Fat body insulin signalling has been known to modulate the overall size of the animal by altering systemic ecdysone levels and thus we considered whether the insulin signalling pathway may influence cachexia via this growth hormone (Caldwell *et al*, 2005; Colombani *et al*, 2005; Mirth *et al*, 2005). We have previously shown that decreasing systemic ecdysone levels, via prothoracic gland (PG)-specific expression of an RNAi against *torso*, increased overall body size, but was not sufficient to cause muscle detachment (Lodge *et al*, 2021). Here we show that a reduction in body size by increasing global ecdysone levels through PG-specific expression of Ras^{V12} (Caldwell *et al*, 2005) failed to alter the level of muscle detachment in tumour bearing animals (Appendix Fig S1A–C, $QRas^{V12}$, $scrib^{RNAi}$). Finally, in tumour bearing animals fed a sterol-free diet, that underwent a prolonged 3rd instar stage due to reduced ecdysone levels (Parkin & Burnet, 1986), we activated insulin signalling in the fat body via Akt overexpression ($QRas^{V12}$, $scrib^{RNAi}$). We found that this manipulation caused a significant decrease in pMad levels in the fat body (Appendix Fig S1G–I) and a rescue of muscle detachment (Appendix Fig S1D–F), similar to animals fed a standard diet (Fig 3F–H). Together, our data suggest that systemic ecdysone levels are unlikely to be involved in mediating the effects of fat body Insulin/TGF-β signalling on muscle detachment.

Tumour secreted ImpL2 and Gbb act additively to affect muscle integrity

Although our data suggest that insulin and TGF-β signalling act in a linear pathway in the fat body to facilitate cachexia, it is unclear

 © 2023 The Authors

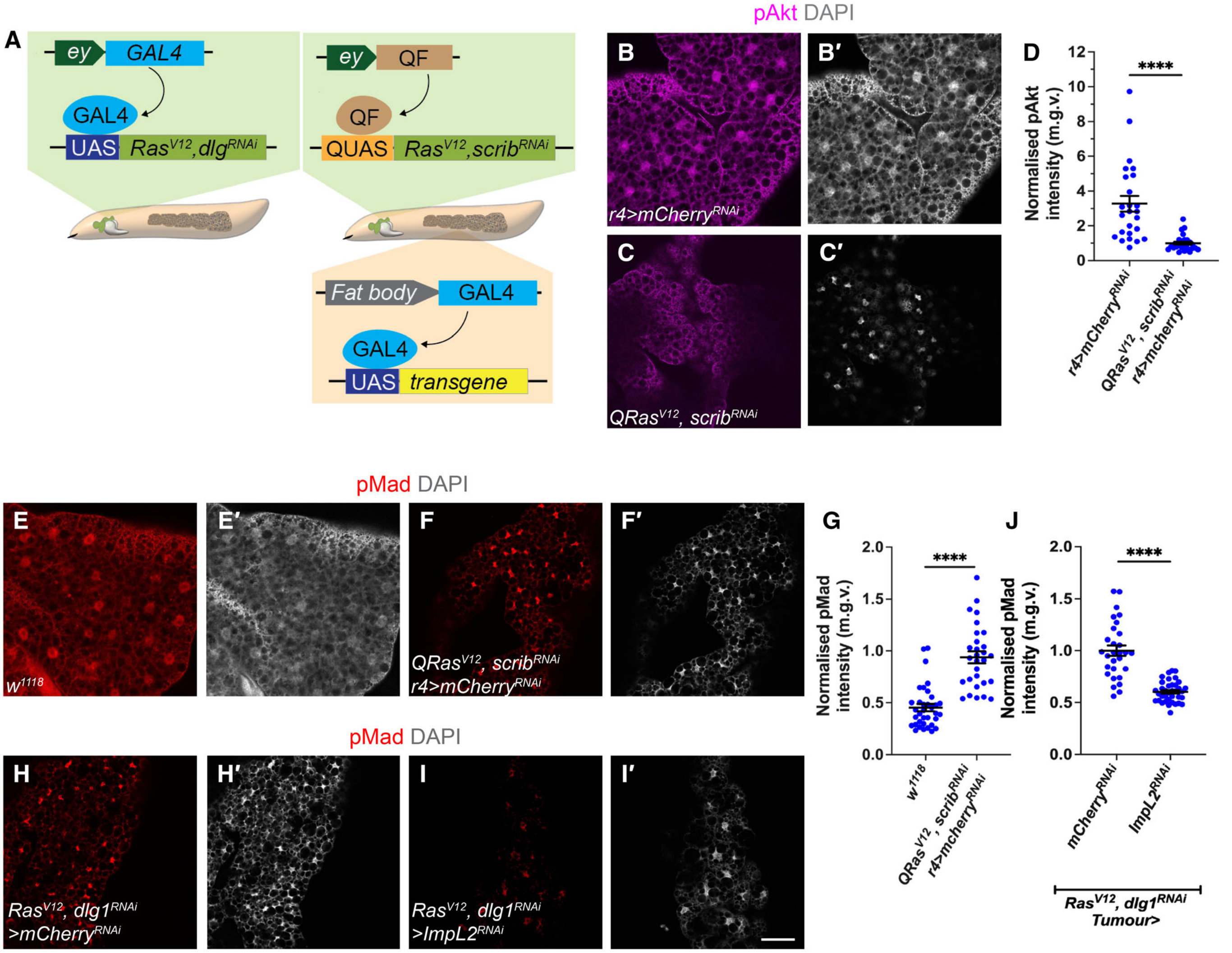

Figure 1. Insulin signalling in the fat body negatively inhibits TGFß signalling in cachexia.

A Schematic depicting the two tumour models utilised in this study. The *GAL4-UAS* induced *Ras*^{V12};*dlg1*^{RNAi} tumour, or the *QF2-QUAS* induced *Ras*^{V12}*scrib*^{RNAi} tumour.

B–C Fat body stained for pAkt from control (*r4>mCherry*^{RNAi}) and *QRas*^{V12}*scrib*^{RNAi}; *r4>mCherry*^{RNAi} tumour-bearing animals, counter stained in (B′–C′) for DAPI.

D Quantification of normalised (to tumour) fat body pAkt intensity in (B–C). *r4>mCherry*^{RNAi} (n = 28), *QRas*^{V12}*scrib*^{RNAi}; *r4>mCherry*^{RNAi} (n = 25).

E–F Fat body stained for pMad from control (ω^{1118}) and *QRas*^{V12}*scrib*^{RNAi}; *r4>mCherry*^{RNAi} tumour-bearing animals, counter stained in (E′–F′) for DAPI.

G Quantification of normalised (to tumour) fat body pMad intensity in (E–F). ω^{1118} (n = 32), *QRas*^{V12}*scrib*^{RNAi}; *r4>mCherry*^{RNAi} (n = 31).

H–I Fat body of *Ras*^{V12}*dlg1*^{RNAi} tumour-bearing animals where *mCherry*^{RNAi} or *ImpL2*^{RNAi} was expressed in the tumour, stained for pMad, counter stained in (H′–I′) for DAPI.

J Quantification of fat body pMad intensity in (H–I). *mCherry*^{RNAi} (n = 29), *ImpL2*^{RNAi} (n = 37).

Data information: Scale bar is 50 μm for fat body pAkt and pMad staining, fat body is stained at 6 days after tumour induction. Graphs are represented as Mean ± SEM, n = the number of samples. (****) $P < 0.0001$, two-tailed unpaired student's t-tests were used to test for significant differences. The Welch's correction was applied in cases of unequal variances.
Source data are available online for this figure.

how the tumour-secreted proteins ImpL2 and Gbb interact with each other to facilitate the progression of cachexia. To determine if the two proteins interact, we first assessed whether ImpL2 regulates TGF-β signalling by influencing *gbb* expression in the tumour. Upon the knockdown of *Impl2*, we found that tumour *gbb* was not significantly altered (Fig EV2A). Next, to determine whether ImpL2 and Gbb acted synergistically to affect muscle integrity, we knocked down both *gbb* and *ImpL2* in the tumour (*Ras*^{V12}, *dlg1*^{RNAi}). Inhibition of both ligands resulted in a greater rescue in muscle integrity than knockdown of either ligand alone (Fig EV1A–E). We suspected that these additive effects of the proteins were because each protein rescued different aspects of cachexia and thus tested their effects on two important aspects of cachexia: protein synthesis and ECM accumulation. Protein translation (measured using the O-propargyl-puromycin incorporation assay, OPP assay) is significantly downregulated during cachexia (Fig EV1F–H), and ECM

© 2023 The Authors

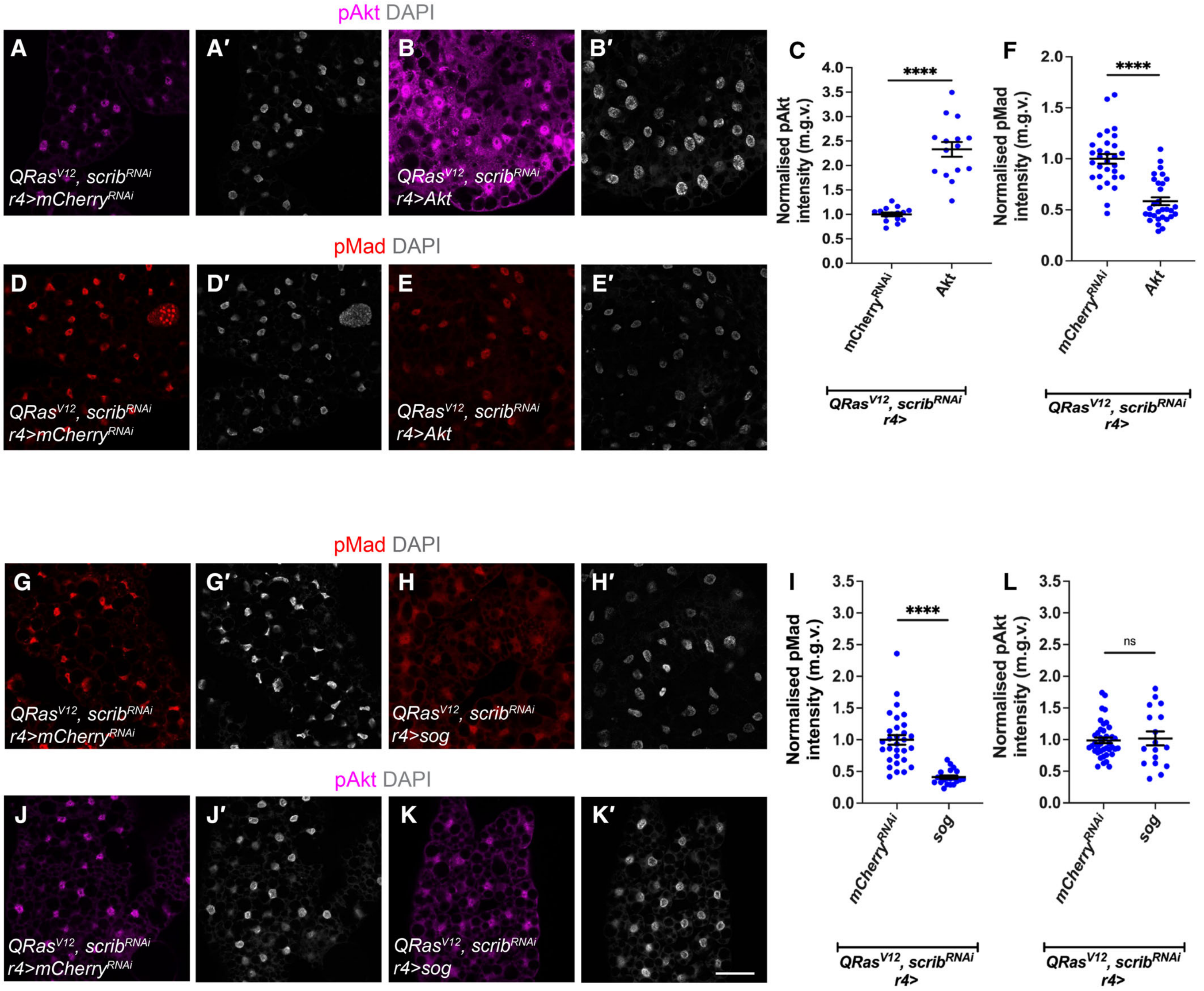

Figure 2. Insulin signalling negatively inhibits TGFß signalling, but TGFß signalling does not affect insulin signalling in the fat bodies of cachectic animals.

A–B Fat body stained for pAkt of *QRas*^{V12}*scrib*^{RNAi} tumour-bearing animals where *mCherry*^{RNAi} or *Akt* was expressed in the fat body (*r4-GAL4*), counter stained in (A′–B′) for DAPI.

C Quantification of normalised fat body pAkt intensity in (A–B). *QRas*^{V12}*scrib*^{RNAi}; *r4>mCherry*^{RNAi} (n = 14), *QRas*^{V12}*scrib*^{RNAi}; *r4>Akt* (n = 14).

D–E Fat body of *QRas*^{V12}*scrib*^{RNAi} tumour-bearing animals where *mCherry*^{RNAi} or *Akt* was expressed in the fat body (*r4-GAL4*), with TGF-ß signalling activation indicated by pMad staining, counter stained in (D′–E′) for DAPI.

F Quantification of normalised fat body pMad intensity in (D–E). *mCherry*^{RNAi} (n = 30), *Akt* (n = 30).

G–H Fat body from animals expressing *mCherry*^{RNAi} or *sog* under the control of *r4-GAL4* in *QRas*^{V12}*scrib*^{RNAi} tumour-bearing animals stained with pMad, counter stained in (G–H′) for DAPI.

I Quantification of normalised fat body pMad intensity in (G–H). *mCherry* ^{RNAi} (n = 30), *sog* (n = 20).

J–K Fat body from animals expressing *mCherry*^{RNAi} or *sog* under the control of *r4-GAL4* in *QRas*^{V12}*scrib*^{RNAi} tumour-bearing animals stained with pAkt, counter stained in (J′–K′) for DAPI.

L Quantification of normalised fat body pAkt intensity in (J–K). *mCherry* ^{RNAi} (n = 40), *sog* (n = 17).

Data information: Scale bar is 50 μm for fat body pAkt and pMad staining, fat body is stained at 6 days after tumour induction. Graphs are represented as Mean ± SEM, n = the number of samples. (****) $P < 0.0001$, (ns) $P > 0.05$), two-tailed unpaired student's t-tests were used to test for significant differences. The Welch's correction was applied in cases of unequal variances.
Source data are available online for this figure.

proteins such as Nidogen accumulated in the fat body of tumour bearing animals (Lodge *et al*, 2021, Fig EV1N–P). We next examined whether the knockdown of *gbb* and *ImpL2*, either alone or together in the tumour, rescued protein synthesis or ECM accumulation. Knockdown of *gbb* alone in the tumour did not significantly rescue protein synthesis in the fat body, however, *ImpL2*^{RNAi} alone

 © 2023 The Authors

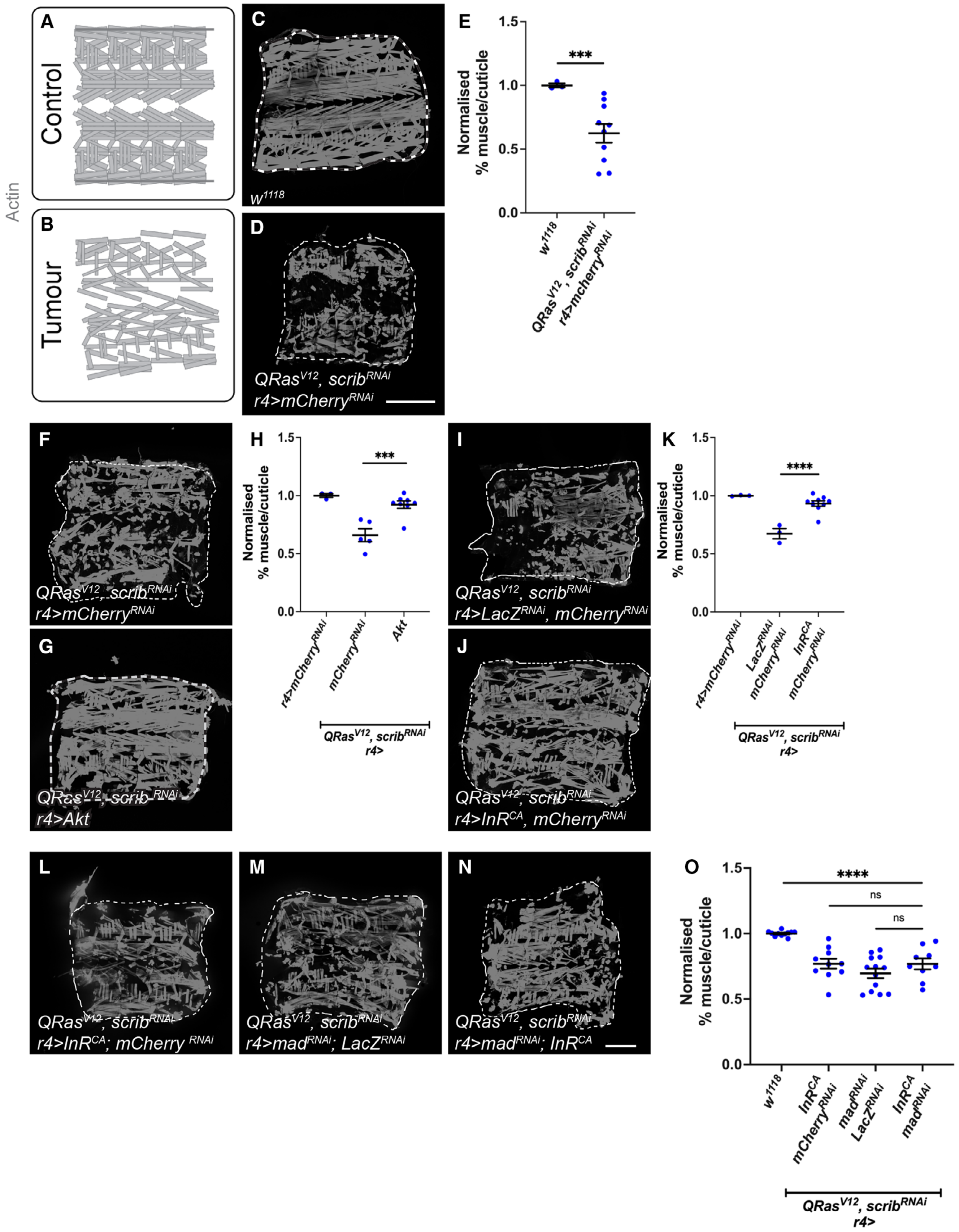
A
Control
Actin
B
Tumour
C
w1118
D
QRasV12, scribRNAi
r4>mCherryRNAi
E
Normalised
% muscle/cuticle
***
w1118
QRasV12, scribRNAi
r4>mcherryRNAi
F
QRasV12, scribRNAi
r4>mCherryRNAi
G
QRasV12, scribRNAi
r4>Akt
H
***
r4>mCherryRNAi
mCherryRNAi
Akt
QRasV12, scribRNAi
r4>
I
QRasV12, scribRNAi
r4>LacZRNAi, mCherryRNAi
J
QRasV12, scribRNAi
r4>InRCA, mCherryRNAi
K
****
r4>mCherryRNAi
LacZRNAi
mCherryRNAi
InRCA
mCherryRNAi
L
QRasV12, scribRNAi
r4>InRCA; mCherry RNAi
M
QRasV12, scribRNAi
r4>madRNAi; LacZRNAi
N
QRasV12, scribRNAi
r4>madRNAi; InRCA
O
****
ns
ns
InRCA
mCherryRNAi
madRNAi
LacZRNAi
InRCA
madRNAi

Figure 3.

◀ **Figure 3. Insulin signalling in the fat body improves muscle integrity in cachectic animals.**

A, B Schematic depicting intact muscle fillet in control animals, versus deteriorated muscle fillets in tumour animals.

C, D Muscle fillets stained with phalloidin (Actin) from ω^{1118} or $QRas^{V12}scrib^{RNAi}$ tumour-bearing animals where $mCherry^{RNAi}$ was expressed in the fat body (*r4-GAL4*). Detachments are marked with yellow arrows.

E Quantification of normalised muscle detachment in (C, D). ω^{1118} (n = 3), $QRas^{V12}scrib^{RNAi}$ (n = 10).

F, G Muscle fillets stained with phalloidin (Actin) from $QRas^{V12}scrib^{RNAi}$ tumour-bearing animals raised at 25°C, where in the fat body, either $mCherry^{RNAi}$, or *Akt* was overexpressed.

H Quantification of normalised muscle detachment in (F, G), $r4{>}mCherry^{RNAi}$ (n = 3). $QRas^{V12}scrib^{RNAi}$; $r4{>}mCherry^{RNAi}$ (n = 5), $QRas^{V12}scrib^{RNAi}$; *r4>Akt* (n = 8).

I, J Muscle fillets stained with phalloidin (Actin) from $QRas^{V12}scrib^{RNAi}$ tumour-bearing animals raised at 25°C, where in the fat body, either $lacZ;mCherry^{RNAi}$, or InR^{CA}; $mCherry^{RNAi}$ was overexpressed.

K Quantification of normalised muscle detachment in (I, J), $r4{>}mCherry^{RNAi}$ (n = 3). $QRas^{V12}scrib^{RNAi}$; $r4{>}lacZ;mCherry^{RNAi}$ (n = 3), $QRas^{V12}scrib^{RNAi}$; $r4{>}InR^{CA}$; $mCherry^{RNAi}$ (n = 9).

L–N Muscle fillets of $QRas^{V12}scrib^{RNAi}$ tumour-bearing animals raised at 25°C where InR^{CA}; $mCherry^{RNAi}$ or mad^{RNAi}; $LacZ^{RNAi}$ or InR^{CA}; mad^{RNAi} was expressed in the fat body (*r4-GAL4*).

O Quantification of muscle detachment in (L–N). ω^{1118} (n = 10), InR^{CA}; $mCherry^{RNAi}$ (n = 8) or mad^{RNAi}; $LacZ^{RNAi}$ (n = 14) or InR^{CA}; mad^{RNAi} (n = 9).

Data information: Scale bar is 200 μm for muscle fillet staining carried out at 7 days after tumour induction. Graphs are represented as Mean ± SEM, n = the number of samples. (***) $P < 0.001$, (****) $P < 0.0001$, (ns) $P > 0.05$. For experiments with two genotypes, two-tailed unpaired student's t-tests were used to test for significant differences. The Welch's correction was applied in cases of unequal variances. For experiments with more than two genotypes, significant differences between specific genotypes were tested using a one-way ANOVA and a subsequent Šidák *post-hoc* test.
Source data are available online for this figure.

or $ImpL2^{RNAi}$ and gbb^{RNAi} together rescued protein synthesis (Fig EV1I–M). Conversely, knockdown of *gbb* alone or knockdown of *gbb* together with *ImpL2* significantly rescued the Nidogen over-accumulation defects observed at the plasma membrane of fat body from tumour-bearing animals, while $ImpL2^{RNAi}$ alone did not (Fig EV1Q–U). Finally, the knockdown of *ImpL2* alone or the co-knockdown of *gbb* and *ImpL2* in the tumour significantly rescued the reduction in OPP levels observed in the muscles of tumour-bearing animals (Fig EV2B–E). Whereas the knockdown of *gbb* alone or knockdown of *gbb* together with *ImpL2* significantly rescued the reduction in Nidogen levels in the muscles of tumour-bearing animals (Fig EV2F–I). Altogether, our data indicate that $ImpL2^{RNAi}$ and gbb^{RNAi} rescue different aspects of cachexia to additively rescue muscle degradation.

Muscle insulin signalling regulates atrophy and muscle integrity in cachexia

A key feature of cancer-induced wasting is the reduction in myofiber size, termed atrophy. This reduction in muscle size can be measured as a ratio of muscle width/length of the VL 3 muscle (Lodge *et al*, 2021; Graca *et al*, 2022). Since tumour-specific *ImpL2* inhibition significantly rescued muscle translation, we suspected that insulin signalling in the muscle may play a role in modulating muscle integrity. To test this, we activated insulin signalling in the muscle of tumour bearing animals ($QRas^{V12}$, $scrib^{RNAi}$) by overexpressing *Akt* with the muscle-specific driver *MHC-GAL4*. This manipulation significantly rescued muscle integrity (Fig EV3A–C), muscle width/length ratio (Fig EV3D–F), as well as muscle protein translation as indicated by OPP (Fig EV3P). Surprisingly, the muscle ECM levels was further reduced by muscle-specific Akt overexpression (Fig EV3G–I). This suggests that muscle insulin signalling predominantly regulate translation and atrophy. Interestingly, while muscle pMad levels are elevated in tumour-bearing animals and that tumour-specific *ImpL2* inhibition was able to reduce muscle TGF-β signalling in tumour-bearing animals (Fig EV3Q–S); muscle-specific expression of mad^{RNAi} in tumour bearing animals ($QRas^{V12}$, $scrib^{RNAi}$) using *MHC-GAL4* (Fig EV3T) was not able to improve muscle integrity. This suggests that although muscle TGF-β signalling is responsive to circulating insulin levels, muscle TGF-β signalling is functionally dispensable in facilitating muscle degradation during cachexia.

Fat body TGF-β signalling, Rab10 and SPARC regulate ECM accumulation and muscle integrity in cachexia

Next, we examined whether modulating fat body insulin or TGF-β signalling can improve muscle integrity. We found that fat body specific Akt expression was not able to rescue muscle atrophy caused by $QRas^{V12}Scrib^{RNAi}$ tumours (Fig EV3J–L). As TGF-β signalling acts downstream of insulin signalling in the fat body of tumour bearing animals, we next asked if inhibition of TGF-β signalling in the fat body via the expression of an RNAi against *mad* improved muscle atrophy in tumour bearing animals ($QRas^{V12}Scrib^{RNAi}$). Interestingly, although fat body mad^{RNAi} expression was able to improve overall muscle integrity (Lodge *et al*, 2021), this manipulation did not significantly improve muscle atrophy (Fig EV3M–O). It however was able to significantly reduced ECM accumulation in the fat body (Fig 4A–B and E) which consequently led to increased muscle Nidogen levels (Fig 4C, D and F). Together, these data suggest that fat body insulin and TGF-β signalling converge to specify ECM levels in the fat body and in turn, the muscle during cachexia.

It has previously been shown that muscle ECM proteins are mostly derived from the fat body and blood cells (Dai *et al*, 2018); furthermore, in the context of cachexia, we observed a correlation between ECM accumulation in the fat body, and ECM depletion in the muscle. We therefore hypothesised that cachectic fat body may be trapping ECM proteins and preventing ECM secretion to the muscle, causing muscle degradation. We first examined whether this ECM accumulation is intracellular or extracellular. We found that without detergent, we could still detect GFP labelled Viking with an anti-GFP antibody, suggesting that these accumulations are extracellular (Fig EV4A–A'). To explore whether this may be the case, we blocked endocytosis in the fat body (*CG-GAL4*) through expression of a temperature-sensitive dominant negative form of *shibire* (shi^{ts}) (Zang *et al*, 2015). It was previously shown that fat body *shibire*

 © 2023 The Authors

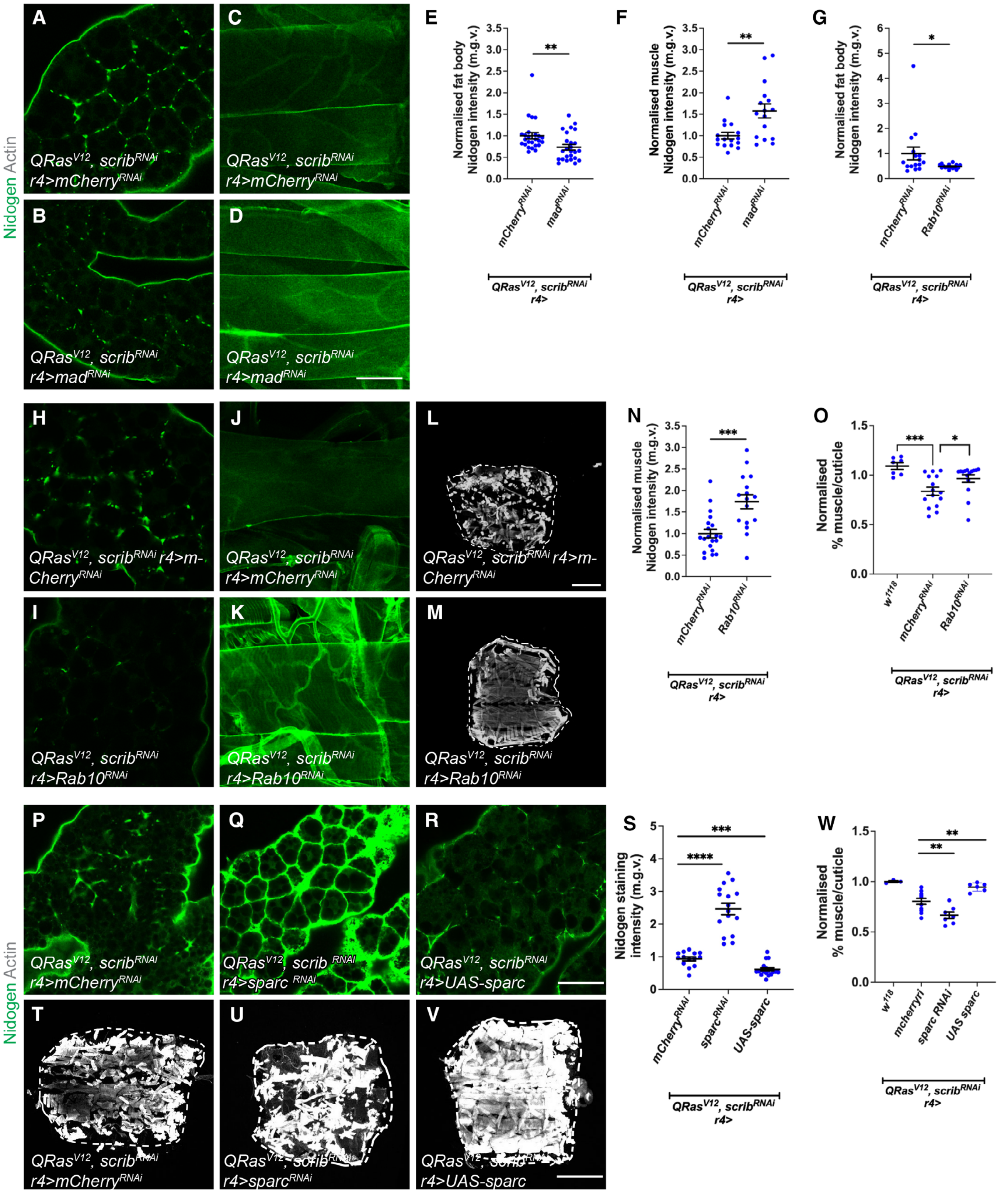
Nidogen Actin
A
QRas^{V12}, scrib^{RNAi} r4>mCherry^{RNAi}
B
QRas^{V12}, scrib^{RNAi} r4>mad^{RNAi}
C
QRas^{V12}, scrib^{RNAi} r4>mCherry^{RNAi}
D
QRas^{V12}, scrib^{RNAi} r4>mad^{RNAi}
E
Normalised fat body Nidogen intensity (m.g.v.)
**
mCherry^{RNAi}
mad^{RNAi}
QRas^{V12}, scrib^{RNAi} r4>
F
Normalised muscle Nidogen intensity (m.g.v.)
**
mCherry^{RNAi}
mad^{RNAi}
QRas^{V12}, scrib^{RNAi} r4>
G
Normalised fat body Nidogen intensity (m.g.v.)
*
mCherry^{RNAi}
Rab10^{RNAi}
QRas^{V12}, scrib^{RNAi} r4>
H
QRas^{V12}, scrib^{RNAi} r4>m-Cherry^{RNAi}
I
QRas^{V12}, scrib^{RNAi} r4>Rab10^{RNAi}
J
QRas^{V12}, scrib^{RNAi} r4>mCherry^{RNAi}
K
QRas^{V12}, scrib^{RNAi} r4>Rab10^{RNAi}
L
QRas^{V12}, scrib^{RNAi} r4>m-Cherry^{RNAi}
M
QRas^{V12}, scrib^{RNAi} r4>Rab10^{RNAi}
N
Normalised muscle Nidogen intensity (m.g.v.)
***
mCherry^{RNAi}
Rab10^{RNAi}
QRas^{V12}, scrib^{RNAi} r4>
O
Normalised % muscle/cuticle
***
*
w^{1118}
mCherry^{RNAi}
Rab10^{RNAi}
QRas^{V12}, scrib^{RNAi} r4>
Nidogen Actin
P
QRas^{V12}, scrib^{RNAi} r4>mCherry^{RNAi}
Q
QRas^{V12}, scrib ^{RNAi} r4>sparc ^{RNAi}
R
QRas^{V12}, scrib^{RNAi} r4>UAS-sparc
S
Nidogen staining intensity (m.g.v.)
***
****
mCherry^{RNAi}
sparc^{RNAi}
UAS-sparc
QRas^{V12}, scrib^{RNAi} r4>
W
Normalised % muscle/cuticle
**
**
w^{118}
mcherryi
sparc RNAi
UAS sparc
QRas^{V12}, scrib^{RNAi} r4>
T
QRas^{V12}, scrib^{RNAi} r4>mCherry^{RNAi}
U
r4>sparc^{RNAi}
V
r4>UAS-sparc

Figure 4.

◀ **Figure 4. TGF-ß signalling in the fat body rescues muscle detachment via regulating fat body ECM secretion.**

A, B Fat body stained with the ECM protein Nidogen from *QRas*^{V12}*scrib*^{RNAi} tumour-bearing animals, where *mCherry*^{RNAi} or *mad*^{RNAi} was expressed in the fat body (*r4-GAL4*).

C, D Muscles stained with Nidogen from *QRas*^{V12}*scrib*^{RNAi} tumour-bearing animals, where *mCherry*^{RNAi} or *mad*^{RNAi} was expressed in the fat body (*r4-GAL4*).

E Quantification of fat body Nidogen staining in (A, B). *mCherry*^{RNAi} (n = 25), *mad*^{RNAi} (n = 25).

F Quantification of muscle Nidogen in (C, D). *mCherry* ^{RNAi} (n = 16), *mad*^{RNAi} (n = 16).

G Quantification of normalised fat body Nidogen staining in (H, I). *mCherry*^{RNAi} (n = 16), *Rab10*^{RNAi} (n = 16).

H, I Fat body stained with the ECM protein Nidogen from *QRas*^{V12}*scrib*^{RNAi} tumour-bearing animals, where *mCherry*^{RNAi} or *Rab10*^{RNAi} was expressed in the fat body (*r4-GAL4*).

J, K Muscles stained with Nidogen from *QRas*^{V12}*scrib*^{RNAi} tumour-bearing animals, where *mCherry*^{RNAi} or *Rab10*^{RNAi} was expressed in the fat body (*r4-GAL4*).

L, M Muscle fillets stained with phalloidin (Actin) from *QRas*^{V12}*scrib*^{RNAi} tumour-bearing animals, where *mCherry*^{RNAi} or *Rab10*^{RNAi} was expressed in the fat body (*r4-GAL4*). Day 6 muscles used here.

N Quantification of normalised muscle Nidogen staining in (J, K). *mCherry*^{RNAi} (n = 20), *Rab10*^{RNAi} (n = 16).

O Quantification of normalised muscle detachment in (L, M). *ω*^{1118} (n = 5), *QRas*^{V12}*scrib*^{RNAi}*; r4>mCherry*^{RNAi} (n = 11), *QRas*^{V12}*scrib*^{RNAi}*;r4>Rab10*^{RNAi} (n = 11).

P–R Fat body stained with the ECM protein nidogen from *QRas*^{V12}*scrib*^{RNAi} tumour-bearing animals, where *mCherry*^{RNAi} or *sparc*^{RNAi} or *UAS-sparc* was expressed in the fat body (*r4-GAL4*).

S Quantification of normalised fat body nidogen staining in (P–R). *mCherry*^{RNAi} (n = 12), *sparc*^{RNAi} (n = 16), *UAS-sparc* (n = 10).

T–V Muscle fillets stained with phalloidin (Actin) from *QRas*^{V12}*scrib*^{RNAi} tumour-bearing animals, where *mCherry*^{RNAi} or *sparc*^{RNAi} or *UAS-sparc* was expressed in the fat body (*r4-GAL4*).

W Quantification of normalised muscle fillet in (T–V). *ω*^{1118} (n = 3), *QRas*^{V12}*scrib*^{RNAi}*; r4>mCherry*^{RNAi} (n = 10), *QRas*^{V12}*scrib*^{RNAi}*; r4>sparc*^{RNAi} (n = 6), *QRas*^{V12}*scrib*^{RNAi}*; r4>UAS-sparc* (n = 6).

Data information: Scale bar is 200 µm for muscle fillet (L, M, T–V) and 50 µm for muscle Viking staining done at day 7 after tumour induction (C, D, J, K), and scale bar is 50 µm for fat body staining (A, B, H, I, P–R) done at day 6 after tumour induction. Graphs are represented as Mean ± SEM, n = the number of samples. (*) $P < 0.05$ (**) $P < 0.01$, (***) $P < 0.001$, (****) $P < 0.0001$. For experiments with two genotypes, two-tailed unpaired student's *t*-tests were used to test for significant differences. The Welch's correction was applied in cases of unequal variances. For experiments with more than two genotypes, significant differences between specific genotypes were tested using a one-way ANOVA and a subsequent Šidák *post-hoc* test.
Source data are available online for this figure.

knockdown causes trapping of outgoing ECM proteins, such as Nidogen, in the fat body membrane (Zang *et al*, 2015). Consistent with our hypothesis, we found that blocking endocytosis resulted in a significant downregulation of Nidogen in the muscles (Fig EV4B–D) and an increase in muscle detachment (Fig EV4E–G). Together, these data suggest that fat body ECM accumulation may contribute to the muscle ECM deficit and muscle detachment.

To test whether ECM accumulation in the fat body can affect muscle detachment in the context of cancer cachexia, we next tried to modulate ECM levels in the fat body of tumour bearing animals (*QRas*^{V12}*Scrib*^{RNAi}). We found that an increase in the expression of Collagen IV (*viking* [*vkg*] and *Collagen at 25C* [*Cg25C*]) (Pastor-Pareja & Xu, 2011) in the fat body of tumour bearing animals was sufficient to improve muscle integrity (Fig EV4H–J). It was previously reported that the overexpression of a small GTPase protein called Rab10 can cause an accumulation of ECM proteins (Isabella & Horne-Badovinac, 2016). We found that Rab10 protein levels was indeed elevated in the fat body of tumour bearing animals (Fig EV4K–M). The overexpression of *Rab10*^{RNAi} in the fat body of tumour bearing animals was sufficient to reduce the accumulation of fat body ECM protein Nidogen (Fig 4G–I), increased muscle Nidogen levels (Fig 4J, K and N), and improved muscle attachment (Fig 4L, M and O), suggesting that modulating fat body ECM secretion in cachectic animals can have a direct effect on muscle ECM levels and integrity.

SPARC, the *Drosophila* homologue of BM40/SPARC/osteonectin, is known to be required for ColIV secretion and has been shown to be a chaperone protein for ColIV both intracellularly and extracellularly (Duncan *et al*, 2020). Reminiscent of ColIV accumulation in the cachectic fat body, we found that SPARC also accumulated at the cachectic fat body adhesion sites (Fig EV4N–P), suggesting that SPARC together with ColIV might be stuck within the fat body. It has been shown previously that the disruption of SPARC caused the retention of ColIV in the membranes of fat body cells (Pastor-Pareja & Xu, 2011; Shahab *et al*, 2015). We found that the expression of a *sparc* RNAi in the fat body of tumour bearing animals caused further accumulation of Nidogen, and additional deterioration of muscle morphology (Fig 4P–W). Conversely, the overexpression of *sparc* (Portela *et al*, 2010), significantly reduced Nidogen accumulation in the fat body, and improved muscle morphology (Fig 4P–W), suggesting that by over-riding the secretion problems from the fat body, SPARC can help with ECM secretion and muscle integrity. Finally, the activation of TGF-β signalling pathway, via the overexpression of Mad, caused the transcriptional upregulation of both SPARC and Rab10 (Fig EV5B). This suggests that in the wildtype fat body, TGF-β signalling can modulate the expression level of these ECM secretion modulators. Together, our data suggest that the activation of TGF-β signalling pathway can contribute to an accumulation of ECM proteins in the fat body, through the modulation of ECM regulators SPARC and Rab10. In the context of cachexia, this can result in a deficit of ECM proteins in the muscle, causing muscle detachment.

Insulin signalling can function via TGF-β signalling in the wildtype fat body

Next, we assessed whether the cross-regulatory relationship between insulin signalling and TGF-β signalling observed in the cachectic fat body is relevant in a developmental context. For this, we first starved larvae by subjecting animals to a diet of 1% agarose in PBS for 24 h to reduce their levels of circulating Ilps (Brogiolo *et al*, 2001). Starved larvae exhibited the expected downregulation in fat body pAkt, and a significant increase in fat body pMad levels (Fig 5A–C) compared to controls fed on a standard laboratory diet.

 © 2023 The Authors

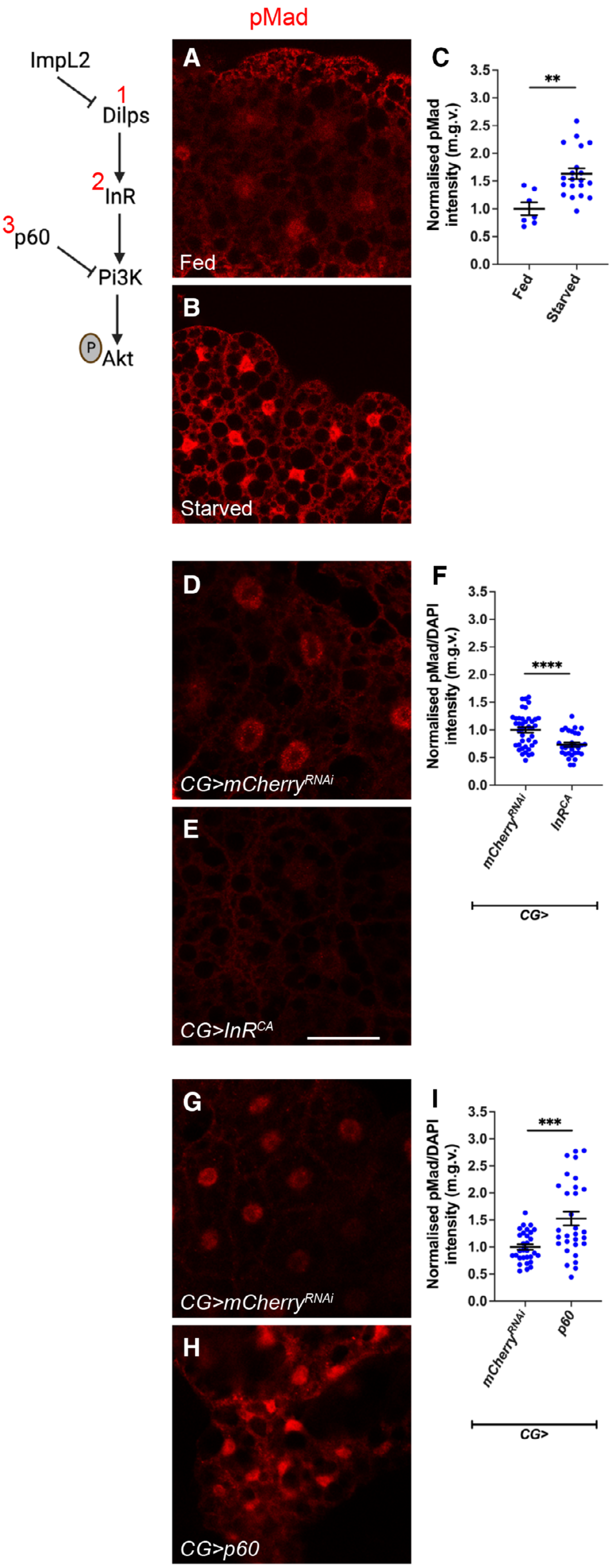

Figure 5. Fat body insulin signalling negatively regulates TGF-ß signalling.

Schematic on the left depicts the insulin-Pi3K signalling pathway, where the components manipulated are labelled with numbers and are referred to below.

A, B Fat body from fed and starved animals where Dilps level is reduced (1) with TGF-ß signalling activation indicated by pMad staining, dissected at day 5 after larval hatching (ALH).

C Quantification of normalised pMad staining in (A, B). Fed (n = 7), starved (n = 20).

D, E Fat body from animals raised at 18°C, dissected at day 7 ALH, that express *mCherry*^{RNAi} or *InR*^{CA} (2) under the control of *CG-GAL4*, with TGF-ß signalling activation indicated by pMad staining.

F Quantification of normalised pMad staining in (D, E). *mCherry*^{RNAi} (n = 30), *InR*^{CA} (n = 40).

G, H Fat body from animals expressing *mCherry*^{RNAi} or *p60* (3) under the control of *CG-GAL4*, with TGF-ß signalling activation indicated by pMad staining, dissected at day 5 ALH.

I Quantification of normalised pMad staining in (G, H). *mCherry*^{RNAi} (n = 30), *p60* (n = 30).

Data information: Scale bar is 50 μm. Graphs are represented as Mean ± SEM, n = the number of samples. (**) $P < 0.01$, (***) $P < 0.001$, (****) $P < 0.0001$, two-tailed unpaired student's t-tests were used to test for significant differences. The Welch's correction was applied in cases of unequal variances. Source data are available online for this figure.

Next, we used *CG-GAL4* to express a constitutive activate form of *InR* (*InR*^{CA}). This caused an upregulation in fat body pAkt and significant reduction in fat body pMad levels (Fig 5D–F). Conversely, when *CG-GAL4* was used to overexpress the gene for the adaptor protein *p60* which acts downstream of InR to recruit DP110 and downregulates insulin signalling when overexpressed (Weinkove *et al*, 1999), we observed an upregulation in fat body pMad levels (Fig 5G–I). Together, our data suggest that the insulin signalling pathway inhibits TGF-β signalling in the wildtype fat body.

We next tested if reduced Insulin signalling in the fat body phenocopies the Vkg accumulation observed upon fat body activation of TGF-β signalling. Vkg accumulated in the membranes of fat body upon the overexpression of a dominant negative form of *Target of Rapamycin* (*TOR*^{DN}) using *r4-GAL4* (Fig 6A–C). Similarly, Vkg accumulation was observed upon *p60* overexpression (Fig 6E–G). Moreover, as with the activation of Tkv (Lodge *et al*, 2021), expression of *TOR*^{DN} did not induce muscle detachment (Fig 6D). This indicates that these signals are indeed functionally equivalent and thus wild-type fat body insulin signalling may also act through TGF-β. Interestingly, however, fat body *p60* overexpression caused a small but significant reduction in muscle attachment, suggesting that fat body insulin signalling may play additional roles to induce muscle detachment that are independent of TGF-β (Fig 6H).

To determine whether insulin signalling knockdown induces ECM accumulation in the fat body via TGF-β signalling, we expressed *p60* while simultaneously knocking down TGF-β signalling using *mad*^{RNAi}. Similar to *CG>lacZ*^{RNAi}; *mad*^{RNAi}, very little Vkg was detected at the plasma membrane of *CG>p60*; *mad*^{RNAi} fat bodies (Fig 6I–K). Together, these data suggest that the accumulation of ECM proteins in the fat bodies of *CG>p60* or *CG>TOR*^{DN} larvae is dependent on *mad*, functionally placing insulin signalling upstream of TGF-β signalling in the wild-type fat body.

Insulin signalling affects TGF-β signalling via TOR and S6K

pAkt triggers phosphorylation cascades that result in deactivation of the transcription factor Forkhead box, sub-group O (FOXO) and

© 2023 The Authors

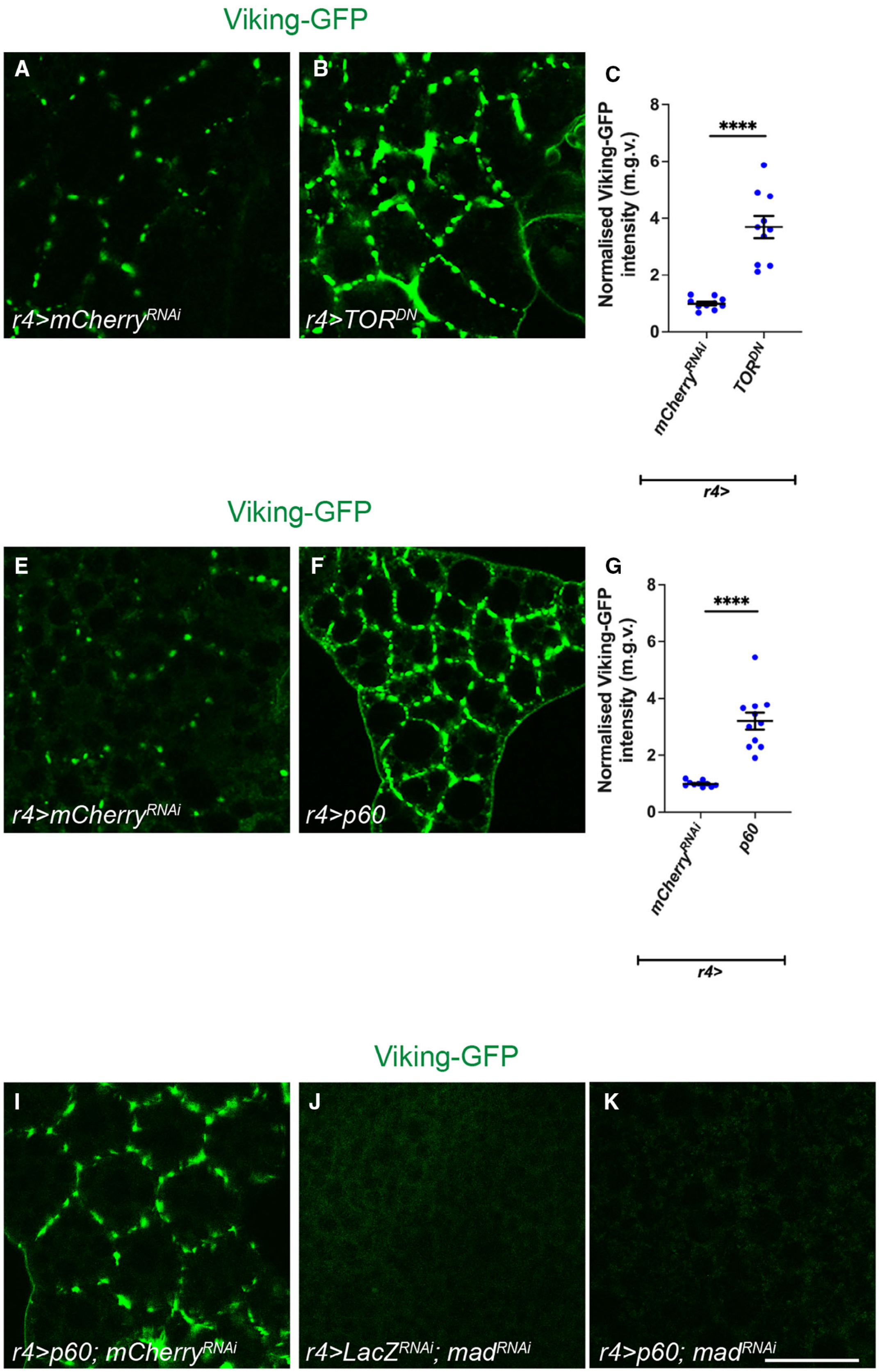

Figure 6.

© 2023 The Authors

Figure 6. Fat body insulin signalling negatively regulates ECM accumulation.

A, B Viking::GFP localisation in fat body from animals expressing *mCherry*^{RNAi} or *TOR*^{DN} under the control of *r4-GAL4*.
C Quantification of normalised Viking::GFP intensity in (A, B). *mCherry*^{RNAi} (n = 10) *TOR*^{DN} (n = 9).
D Quantification of normalised muscle detachment caused by the expression of *mCherry*^{RNAi} or *TOR*^{DN} under the control of *r4-GAL4*. *mCherry*^{RNAi} (n = 8) *TOR*^{DN} (n = 7).
E, F Viking::GFP localisation in fat body from animals expressing *mCherry*^{RNAi} or *p60* under the control of *r4-GAL4*.
G Quantification of normalised Viking::GFP intensity in (E, F). *mCherry*^{RNAi} (n = 9), *p60* (n = 10).
H Quantification of normalised muscle detachment caused by the expression of *mCherry*^{RNAi} or *p60* under the control of *r4-GAL4*. *mCherry*^{RNAi} (n = 12), *p60* (n = 16).
I–K Viking::GFP localisation in fat body from animals expressing *p60; mCherry*^{RNAi} or *lacZ*^{RNAi}; *mad*^{RNAi} or *p60; mad*^{RNAi} under the control of *r4-GAL4*.

Data information: Scale bar is 50 μm for fat body staining, dissected at day 5 ALH. Graphs are represented as Mean ± SEM, n = the number of samples. (*) $P < 0.05$ (****) $P < 0.0001$, (ns) $P > 0.05$, two-tailed unpaired student's t-tests were used to test for significant differences. The Welch's correction was applied in cases of unequal variances.
Source data are available online for this figure.

activation of TOR. Once activated, TOR activates S6K and deactivates 4E-BP respectively by inducing their phosphorylation (Zhang *et al*, 2000). Together, FOXO, TOR, S6K and 4E-BP make up the most downstream components of the insulin signalling pathway and induce the cellular changes triggered by the activation of the pathway (Giannakou & Partridge, 2007; Semaniuk *et al*, 2021). To test which of these factors regulate TGF-β signalling, we used *CG-GAL4* to overexpress *FOXO*, *TOR*^{DN} or constitutively activated forms of *S6K* (*S6K*^{CA}) or *4E-BP* (*4E-BP*^{CA}) in the fat body. We found that fat body from *CG>4E-BP*^{CA} and *CG>FOXO* larvae exhibited no change in pMad levels (Fig 7D–F and J–L), whereas fat body from *CG>TOR*^{DN} larvae exhibited increased pMad levels (Fig 7A–C) and *CG>S6K*^{CA} fat body exhibited reduced pMad levels (Fig 7G–I). To assess whether *InR* inhibited TGF-β signalling in the fat body via *TOR*, we tested the epistatic relationship between *TOR*^{DN} and *InR*^{CA} expression. We found that the co-expression of *TOR*^{DN} and *InR*^{CA} caused elevated pMad, phenocopying knockdown of *TOR* (*TOR*^{DN}) alone, suggesting that insulin signalling modulates fat body TGF-β signalling via *TOR* (Fig 7M–Q).

Insulin does not regulate TGF-β in the muscle or the wing imaginal discs

To determine whether the effect of insulin signalling on TGF-β signalling is fat body specific, we next assessed whether modulating insulin signalling in the muscle, or the wing discs affects pMad levels in these tissues. We found that overexpression of a constitutively active form of *DP110* (*DP110*^{CAAX}) (Leevers *et al*, 1996) using the muscle driver *MHC-GAL4* did not significantly alter muscle pMad staining (Appendix Fig S2A–C). In the wing imaginal disc, heat shock-induced clones overexpressing *InR*^{CA} or *p60* exhibited expected changes in clone size (Appendix Fig S2D–E') and pAkt levels. However, these alterations in insulin signalling did not significantly alter TGF-β signalling (Appendix Fig S2D–E'). Similarly, *tkv*^{CA} or *mad*^{RNAi} clones caused altered clone size (Appendix Fig S2F–G') and changes in pMad levels but caused no significant changes in pAkt levels in the wing (Appendix Fig S2F–G'). This indicates that the cross-regulation of these signalling pathways does not hold in the muscle or the wing imaginal discs.

Insulin signalling influences TGF-β signalling by modulating fat body *sog* expression

Since the Bone Morphogenic Protein (BMP) arm of the TGF-β signalling pathway controls Mad phosphorylation, we suspected that the insulin signalling pathway may modulate TGF-β signalling levels by directly modulating the transcription of components or regulators of the BMP signalling pathway. To test this, qPCR was conducted on the fat body of *CG>InR*^{CA} and *CG>mCherry*^{RNAi} animals (Fig EV5C). We assessed the expression levels of the BMP ligand encoding genes

Figure 7. Fat body TOR signalling negatively regulates TGF-ß signalling.

Schematic on the left depicts Insulin/Tor signalling pathway, where the components manipulated are labelled with numbers and are referred to below.
A, B Fat body from animals expressing *mCherry*^{RNAi} or *TOR*^{DN} (1) under the control of *CG-GAL4*, with TGF-ß signalling activation indicated by pMad staining.
C Quantification of normalised pMad (to DAPI) staining in (A, B). *mCherry*^{RNAi} (n = 21), *TOR*^{DN} (n = 14).
D, E Fat body from animals expressing *mCherry*^{RNAi} or *4E-BP*^{CA} (4) under the control of *CG-GAL4*, with TGF-ß signalling activation indicated by pMad staining.
F Quantification of normalised pMad staining in (D, E). *mCherry*^{RNAi} (n = 15), *4E-BP*^{CA} (n = 15).
G, H Fat body from animals expressing *mCherry*^{RNAi} or *S6K*^{CA} (2) under the control of *CG-GAL4*, with TGF-ß signalling activation indicated by pMad staining.
I Quantification of normalised pMad staining in (G, H). *mCherry*^{RNAi} (n = 20), *S6K*^{CA} (n = 30).
J, K Fat body from animals expressing *mCherry*^{RNAi} or *FOXO* (3) under the control of *CG-GAL4*, with TGF-ß signalling activation indicated by pMad staining.
L Quantification of normalised pMad staining in (J, K). *mCherry*^{RNAi} (n = 30), *FOXO* (n = 30).
M–P Fat body from animals expressing *mCherry*^{RNAi} or *Tor*^{DN} *(1) or InR*^{CA}; *mCherry*^{RNAi} (5) or *InR*^{CA};*Tor*^{DN} *(5, 1)* under the control of *r4-GAL4*, with TGF-ß signalling activation indicated by pMad staining (the same *mCherry*^{RNAi} sample was used in Fig 8A, as these experiments were carried out together).
Q Quantification of normalised pMad staining in (M–P). *mCherry*^{RNAi} (n = 16), *Tor*^{DN} (n = 20), *InR*^{CA}; *mCherry*^{RNAi} (n = 12), *InR*^{CA};*Tor*^{DN} (n = 16). The same *mCherry*^{RNAi} and *InR*^{CA}; *mCherry*^{RNAi} data points were used as in Fig 8F.

Data information: Scale bar is 50 μm, dissection carried out at 5 days ALH. Graphs are represented as Mean ± SEM, n = the number of samples. (*) $P < 0.05$ (**) $P < 0.01$, (****) $P < 0.0001$, (ns) $P > 0.05$. For experiments with two genotypes, two-tailed unpaired student's t-tests were used to test for significant differences. The Welch's correction was applied in cases of unequal variances. For experiments with more than two genotypes, significant differences between specific genotypes were tested using a one-way ANOVA and a subsequent Šidák *post-hoc* test.
Source data are available online for this figure.

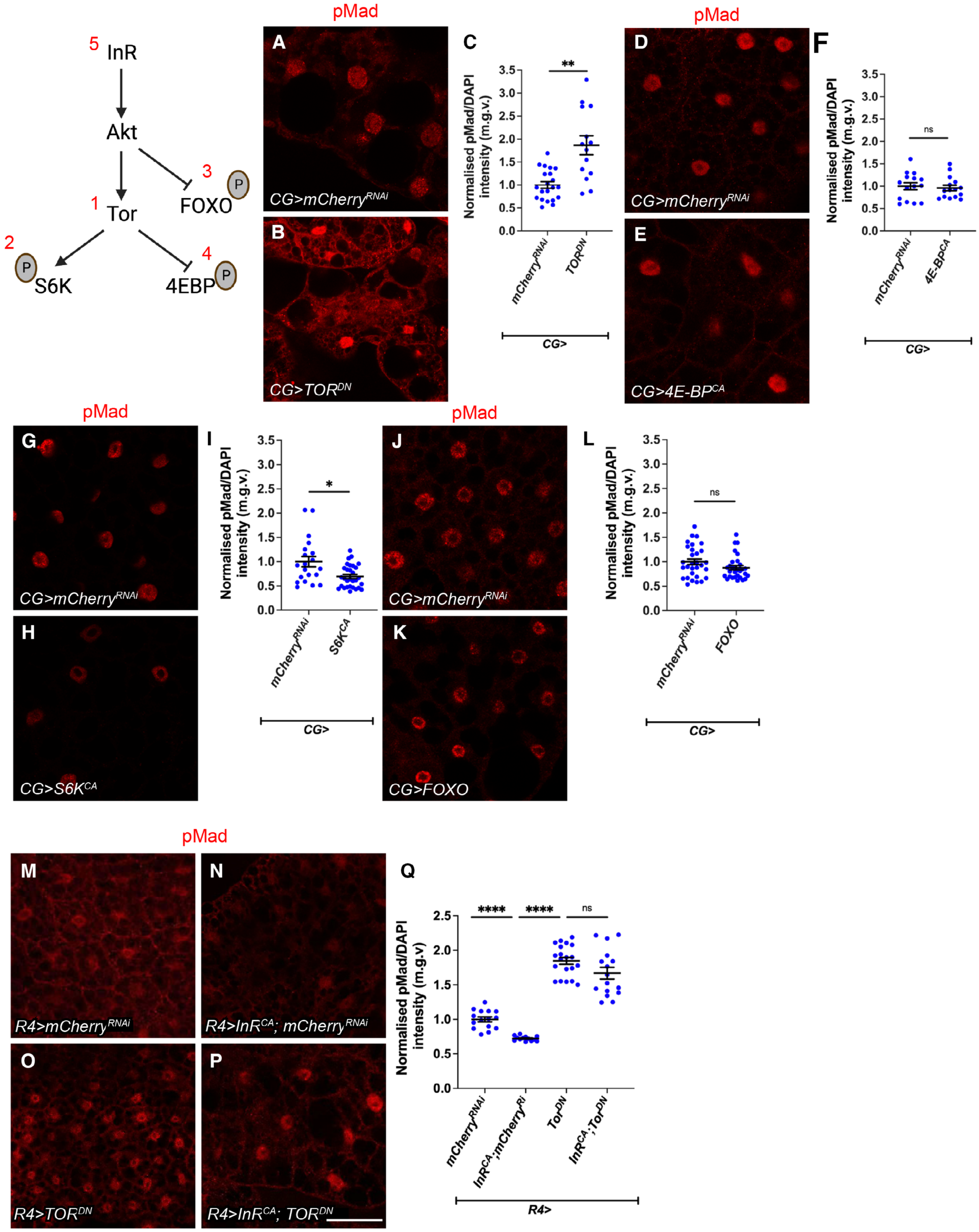

InR
Akt
Tor
FOXO
S6K
4EBP
pMad
A
CG>mCherryRNAi
B
CG>TORDN
C
Normalised pMad/DAPI intensity (m.g.v.)
mCherryRNAi
TORDN
CG>
D
CG>mCherryRNAi
E
CG>4E-BPCA
F
4E-BPCA
G
H
CG>S6KCA
I
S6KCA
J
K
CG>FOXO
L
FOXO
M
R4>mCherryRNAi
N
R4>InRCA; mCherryRNAi
O
R4>TORDN
P
R4>InRCA; TORDN
Q
InRCA;mCherryRi
TorDN
InRCA;TorDN
R4>

Figure 7.

(*gbb*, *decapentaplegic* [*dpp*] and *maverick* [*mav*], but not *scarecrow* as it is only expressed in the CNS during the larval stage, Yoo *et al*, 2020), the four BMP receptor encoding genes (*thickveins* [*tkv*], *saxophone* [*sax*], *punt* and *wishful thinking* [*wit*]), the BMP signalling inhibitor *sog*, the transcription factor *mad*, and the putative regulator of Mad dephosphorylation *dullard* (Urrutia *et al*, 2016). Since non-BMP components of the TGF-β signalling pathway have also been observed to influence Mad phosphorylation (Gesualdi & Haerry, 2007; Peterson *et al*, 2012), we also assessed the transcription of several other TGF-β signalling pathway genes: *babo*, *myoglianin* (*myo*), *dawdle* (*daw*) and *smad on X* (*smox*). Strikingly, we found that *sog* transcription was increased two-fold in the fat body of $CG{>}InR^{CA}$ larvae, while none of the other genes tested exhibited any changes in transcription levels (Fig EV5C). To assess whether Sog mediates the downstream effects of insulin signalling on TGF-β, we expressed sog^{RNAi} in the fat body of larvae expressing InR^{CA} and tested whether *sog* knockdown inhibits the effects of InR^{CA} on fat body pMad levels (Fig 8A–E). We found that *sog* knockdown increased pMad level, and the expression of sog^{RNAi} together with InR^{CA} resulted in an increase in pMad level similar to sog^{RNAi} expression alone. These findings suggest that Sog lies downstream of insulin signalling and mediates the effects of insulin signalling on TGF-β signalling.

Sog is a critical regulator of cachexia

Since insulin signalling likely regulates TGF-β signalling by modulating *sog* expression, we assessed whether Sog modulation is relevant in cachectic animals. Analysis of our previously published hemolymph proteomics data (Lodge *et al*, 2021) showed that Sog is one of the most downregulated circulating proteins in tumour-bearing animals (Fig 8R). The downregulation of haemolymph Sog appears to be dependent on tumour-derived Mmps. Upon the inhibition of Mmps in the tumour via the overexpression of TIMP, we found that circulating Sog levels is restored to wildtype levels (Fig 8R). Together, this suggests that circulating Sog levels are low in the tumour bearing animals. Consistent with this, increasing Sog levels specifically in the fat body, muscle or tumour via Sog overexpression, significantly improved tumour induced muscle detachment (Fig 8F–H, I–K and L–N). Furthermore, the overexpression of Sog in the fat body significantly improved Nidogen accumulation in the fat body (Fig 8O–Q), consistent with our model that TGF-β signalling regulates fat body ECM accumulation. Together, our data show that Sog is a critical regulator of cachexia.

Discussion

It has been shown that cachexia is mediated by humoral factors secreted from the tumour, and the complete removal of cachexia-associated tumours can reverse cachexia (Ni *et al*, 2012). However, in late stage/metastatic cancer patients, the removal of the tumour is often not an option. In this study, we demonstrate, using a *Drosophila* tumour model, that targeting signalling deregulations in the peripheral tissues can be a novel strategy to treat cachexia even when tumours are present, and the signalling status of peripheral tissues can serve as diagnostic biomarker for cachexia. We show that the insulin and TGF-β signalling pathways are deregulated in the peripheral tissues in tumour bearing animals, their disruption can recapitulate some aspects of tissue wasting, and their modulation can rescue tissue wasting in the presence of a tumour.

We have identified the fat body as a central node in our cachexia model. In both fly and mouse cachexia models, the breakdown of fat precedes that of the muscles (Das *et al*, 2011; Lodge *et al*, 2021). Here, we find that tumour secreted proteins ImpL2 and Gbb converge in the fat body to induce the activation of TGF-β signalling (Fig EV5D). TGF-β activation then drives an aberrant accumulation of ECM at the inter-adipocyte junctions in the fat body, which likely prevents ECM secretion and subsequent transport to the muscle. We found that the modulation of ECM secretion via SPARC or Rab10 can ameliorate the cachexia phenotype. It was previously reported that plasma membrane (PM) overgrowth induced by increased

Figure 8. Insulin signalling activates the TGF-ß inhibitor Sog.

A–D′ Overexpression of $mCherry^{RNAi}$, InR^{CA}; $mCherry^{RNAi}$, Sog^{RNAi}; $mCherry^{RNAi}$ and Sog^{RNAi};InR^{CA} under the control of *r4-GAL4* stained for pMad and DAPI. The same $mCherry^{RNAi}$ sample was used in Fig 7M, as these experiments were carried out together).

E Quantification of pMad levels of (A–D′). $mCherry^{RNAi}$ (n = 17), InR^{CA}; $mCherry^{RNAi}$(n = 7), Sog^{RNAi}; $mCherry^{RNAi}$ (n = 26), and Sog^{RNAi};InR^{CA}(n = 19), The same $mCherry^{RNAi}$ and InR^{CA}; $mCherry^{RNAi}$ data points were used as in Fig 6Q.

F, G Muscle fillets stained with phalloidin (Actin) from tumour-bearing ($QRas^{V12}scrib^{RNAi}$) animals where $mCherry^{RNAi}$ or *sog* was expressed in the fat body using *r4-GAL4*.

H Quantification of muscle detachment in (F, G). w^{1118} (n = 3), $mCherry^{RNAi}$ (n = 10), *sog* (n = 8).

I, J Muscle fillets stained with phalloidin (Actin) from upon tumour-specific overexpression of $mCherry^{RNAi}$ or *Sog* in $Ras^{V12}dlg1^{RNAi}$ tumour-bearing animals.

K Quantification of muscle detachment in (I, J). w^{1118} (n = 3), $mCherry^{RNAi}$ (n = 4), *sog* (n = 6).

L, M Muscle fillets stained with phalloidin (Actin) from tumour-bearing animals with overexpression of *luciferase* or *sog* in the muscle via *MEF2-GAL4*.

N Quantification of muscle detachment in (L, M). w^{1118} (n = 3), *luciferase* (n = 10), *Sog* (n = 16).

O, P Fat body stained with the ECM protein Nidogen from $QRas^{V12}scrib^{RNAi}$ tumour-bearing animals, where $mCherry^{RNAi}$ or *UAS-sog* was expressed in the fat body (*r4-GAL4*).

Q Quantification of normalised fat body Nidogen staining in (O, P). $mCherry^{RNAi}$ (n = 9), *UAS-sog* (n = 10). Scale bar is 50 μm for fat body.

R Sog levels in the haemolymph of w^{1118}, $Ras^{V12}dlg1^{RNAi}$ and UAS-TIMP; $Ras^{V12}dlg1^{RNAi}$ tumour-bearing animals (n = 4,4,3).

Data information: Scale bar is 200 μm for muscle fillet done at day 7 after tumour induction (F, G, I, J, L,M) and scale bar is 50 μm for fat body staining done at day 5 for wildtype fat body (A–D′) and 6 days after tumour induction (O, P). Graphs are represented as Mean ± SEM, n = the number of samples. (*) $P < 0.05$ (**) $P < 0.01$, (****) $P < 0.0001$, (ns) $P > 0.05$. For experiments with two genotypes, two-tailed unpaired student's t-tests were used to test for significant differences. The Welch's correction was applied in cases of unequal variances. For experiments with more than two genotypes, significant differences between specific genotypes were tested using a one-way ANOVA and a subsequent Šidák *post-hoc* test.
Source data are available online for this figure.

© 2023 The Authors

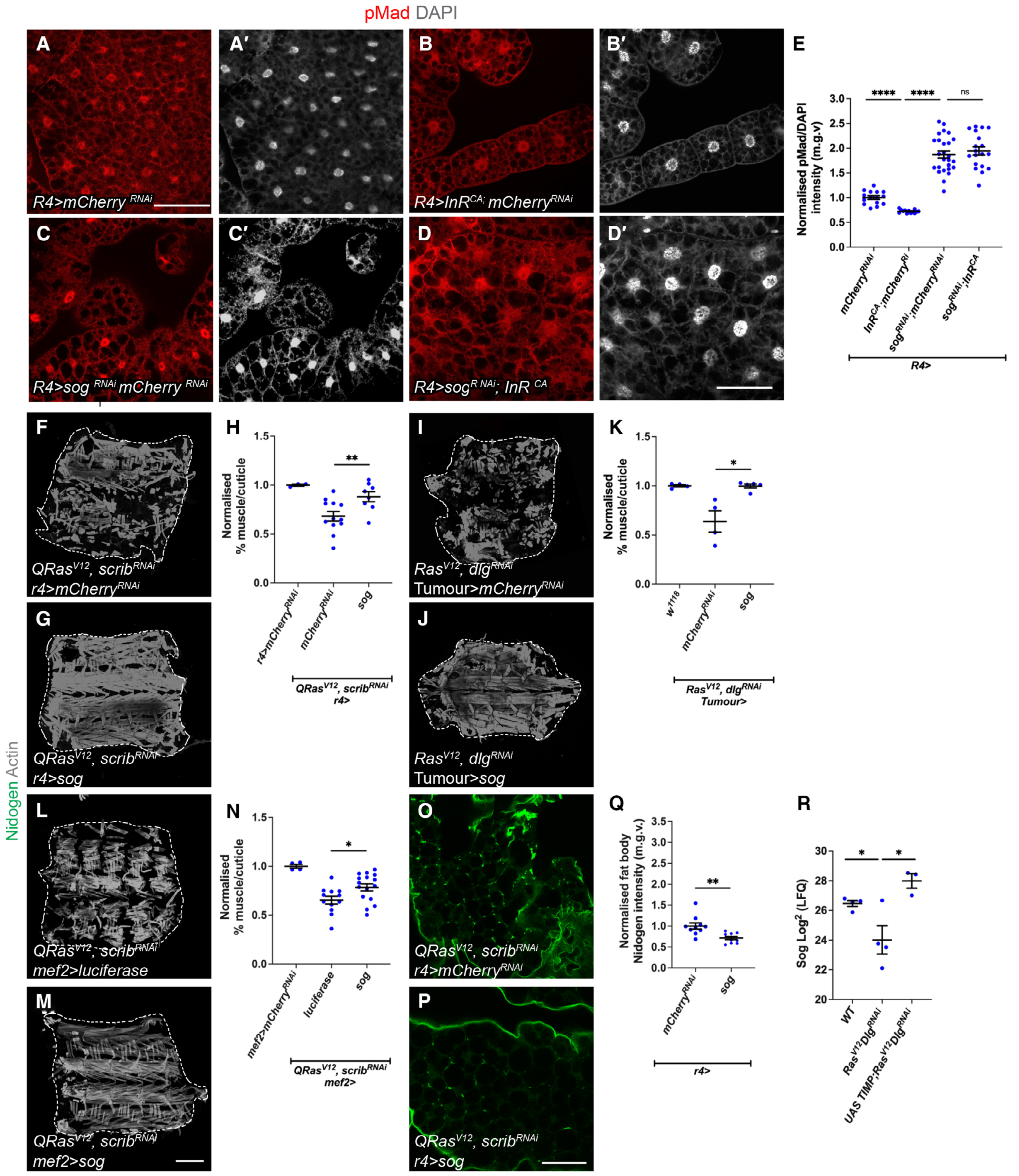

Figure 8.

inflammation can cause pericellular collagen accumulation in the fat body (Zang *et al*, 2015). In the cachectic fat body, we have not observed PM thickening, therefore, it is likely this occurs in cachexia via a different mechanism. Aberrant ECM accumulation in the adipose tissue has been observed in human cachexia patients, and this accumulation has been associated with elevated levels of

© 2023 The Authors

TGF-β signalling (Alves *et al*, 2017). Furthermore, reduced ECM levels have been reported in the muscles in a rat cachexia model (Moraes *et al*, 2017). Thus, altered ECM localisation appears to be a conserved mechanism in cancer cachexia.

In this study, we show that insulin signalling (regulated by tumour-derived ImpL2) directly affects muscle translation rates and atrophy. This, together with fat body TGF-β activation/ECM accumulation, appears to contribute towards the regulation of muscle integrity in the context of cachexia (Fig EV5D). Additional tumour-secreted signals likely exist. Whether these signals also act through the fat body (through regulation of ECM production or via alternative mechanisms), or act directly on the muscle or even via other target tissues, remains to be addressed. Studying these signals, their downstream signalling, as well as identifying additional target tissues where the signalling is relevant, remain important areas of future research.

The insulin signalling pathway plays many important roles in the regulation of growth and metabolism. TGF-β signalling is best known for roles in development, growth and differentiation. More recently, these pathways have been shown to intersect in metabolic contexts. In the fat body, it has been shown under high fat diet induced obesity, that fat body derived Gbb can cause insulin resistance via negative regulation of the insulin signalling pathway (Hong *et al*, 2016). Furthermore, in both *Drosophila* and *C. elegans*, it has been shown that BMP signalling can regulate Ilps to modulate lipid homeostasis (Ballard *et al*, 2010; Clark *et al*, 2021). In the developing muscle, it was recently shown that the Activin branch of the TGF-β signalling pathway positively regulates the insulin signalling pathway via the regulation of a structural protein, MHC (Kim & O'Connor, 2021). In contrast to these studies, we show that, in the fat body, insulin signalling regulates TGF-β signalling by its modulation at the extracellular level via the secreted BMP inhibitor Sog. Interestingly, it has been shown in Zebrafish that IGF signalling can influence the expression of BMPs via Chordin (Sog homologue) during embryonic pattering, indicating that this relationship is conserved across distantly related species (Eivers *et al*, 2004). Although it was previously shown that the insulin signalling effector FOXO can directly bind to the promoter of Sog in adult flies (Birnbaum *et al*, 2019), this does not seem to be the case in the larval fat body as FOXO overexpression did not significantly alter fat body pMad levels. Instead, our data suggest that insulin signalling regulates *sog* expression via TOR and S6K. In support of this, TOR has been shown to influence other transcriptional regulators such as Raptor and Yorkie, thus it may regulate *sog* expression by modulating one of these transcriptional regulators (Parker & Struhl, 2015; Tiebe *et al*, 2015).

Sog has previously been shown to be important in embryonic patterning as well as at the developing cross-veins in the pupal wing. Our study also demonstrates that the inhibition of extracellular BMP ligands via Sog is a novel link between insulin and TGF-β signalling in the fat body. We show that Sog is highly relevant in the context of cachexia, as it is found to be highly downregulated in the haemolymph of tumour-bearing animals. In addition, tissue specific overexpression of *sog* in the tumour, fat body and muscle significantly rescued muscle integrity in the tumour bearing animals. Therefore, it appears that Sog levels in the tumour, as well as in peripheral tissues, play a role in mediating cachexia. As RNAseq data from the peripheral tissues of cachexia patients are not readily available through public data bases, we instead examined cBioPortal data which assesses gene expression in the tumour (5,541 samples in 25 pan-cancer studies) (Cerami *et al*, 2012; Gao *et al*, 2013). We found that there is a mild negative correlation between BMP7 expression (52% homology with gbb) and CHORDIN (CHRD, 40% homology with Sog) in cancers commonly associated with cachexia as well as non-cachectic cancers (Appendix Fig S3), where the correlation is slightly strongly in the cachexia-related cancers. Together, this suggests that BMP and its regulators are expressed in many types of cancers, and that they may regulate each other's expression. As such, CHRD may also be relevant for the progression of cachexia in humans, thus it would be interesting to explore whether circulating CHRD can serve as a potential biomarker for the diagnosis of cachexia. Together, our data suggest that TGF-β and insulin signalling crosstalk is relevant in the context of cancer cachexia. Our data also reveal potential therapeutic avenues to treat cachexia in the clinic when resection of the tumour is not an option in late stage/metastatic patients. These include targeting BMP antagonists such as Sog/CHRD, inhibition of TGF-β signalling, activation of insulin signalling, or enhancement of ECM secretion in the adipose tissue.

Material and Methods

Drosophila stocks and husbandry

The following stocks were used from the Bloomington *Drosophila* stock centre: *CG-GAL4* (BL7011), *Mef2-GAL4* (BL27391), *MHC-GAL4* (BL55133), *r4-GAL4* (BL33832), *UAS-4E-BP*^{CA} (BL24854), *UAS-Akt* (BL8191), *UAS-Dp110*^{CAAX} (BL25908), *UAS-Gbb*$^{RNAi\#2}$ (BL34898), *UAS-InR*^{CA} (BL8263), *UAS-luciferase* (BL64774), *UAS-mad*^{RNAi} (BL31316), *UAS-mCherry*^{RNAi} (BL35785), *UAS-S6K*^{CA} (BL6914), *UAS-Ras*^{V12} (BL64195), *UAS-Shi*^{TS} (BL44222), *UAS-FOXO* (BL9575), *UAS-tkv*^{CA} (BL36536), *UAS-GFP* (BL4775), *UAS-Sparc3* (Helena Richardson), *ey-FLP1;act>CD2>GAL4*, *UAS-GFP* (Lodge *et al*, 2021), *UAS-lacZ*^{RNAi} (BL31562), *sog-lacZ* (BL10132). The following stocks were obtained from the Vienna Drosophila Resource Centre: *UAS-Gbb*^{RNAi} (v330684), *UAS-Impl2*^{RNAi} (v30931), *UAS-Sparc*^{RNAi} (v16677), *UAS-Sog*^{RNAi} (v37405) (Hattori *et al*, 2013). The following stock was obtained from the Kyoto stock centre: *Vkg-GFP* (110692). The following stocks were also used: *Ey-FLP1; QUAS-Ras*^{V12}, *QUAS-scrib*^{RNAi}*/CyOQS; act>CD2>QF, UAS-RFP/TMBQS* (Lodge *et al*, 2021) *Ey-FLP1; UAS-Ras*^{V12}, *UAS-dlg1*^{RNAi}*/CyO, GAL80; act>CD2>GAL4, UAS-GFP* (Manent *et al*, 2017), *Phm-GAL4* (McBrayer *et al*, 2007), *UAS-p60* (Cheng *et al*, 2011), *UAS-Sog-HA* (Yu *et al*, 2000), *UAS-TOR*^{DN} (Zhang *et al*, 2006), *UAS-cg25C;UAS-Vkg* (Van De Bor *et al*, 2015). The knockdown efficiency of *mad*^{RNAi} and *sog*^{RNAi} are shown in Fig EV5.

For the *r4>InR*^{CA}; *mad*^{RNAi} interaction experiment and analysis of atrophy in *QRas*^{V12}*scrib*^{RNAi} larvae carrying *r4>mad*^{RNAi} larvae were reared at 25°C. For experiments with *CG>InR*^{CA} larvae were reared at 18°C. For experiments with *CG>shi*^{TS}, adults were allowed to lay for 24 h at 25°C and progeny transferred to 31°C after an additional 48 h. For all other experiments, adults were allowed to lay for 24 h at 25°C and the progeny then moved to 29°C. Animals were dissected at wandering stage in non-tumour bearing animals (with

© 2023 The Authors

the exception of the *CG>shi*^{TS} experiments, conducted on day 7 animals), and for tumour-bearing animals, they were dissected on 7 days after egg lay or as indicated throughout.

Immunostaining

For muscle staining, larvae were filleted as previously described (Lodge *et al*, 2021; Dark *et al*, 2022), fixed for 30 min in PBS containing 4% formaldehyde and washed with PBS containing 0.3% Triton-X (PBST-0.3). Fat body was fixed for 45 min and washed with PBS containing 0.2% Triton-X (PBST-0.2). Wing discs were fixed for 20 min and washed with PBS containing 0.2% Triton-X (PBST-0.2). Tissues were then stained as per the manufacturer's specifications. Samples were mounted in glycerol and imaged on an Olympus FV3000 confocal microscope. Within a given experiment, all images were acquired using identical settings. Fat body samples were imaged the same day as mounting. To assay for translation in the muscle, we used Click-iT™ Plus OPP Alexa Fluor™ 488 Protein Synthesis Assay Kit (Thermo Fisher, #C10456). Primary antibodies used: ßgal (1:100, Promega), pMad (1:800, Cell Signalling, #9516, used for fat body staining), pMad (1:100, abcam, ab52903, used for muscle staining) pAkt (Cell signalling, 1:100, #4060), Nidogen (gift of Anne Holtz, 1:500), SPARC (1:500, (Shahab *et al*, 2015). Secondary donkey antibodies conjugated to Alexa 555 and Alexa 647 (Molecular Probes) were used at 1:200. DAPI (Molecular Probes) was used at 1:10,000, Phalloidin (Molecular Probes) was used at 1:10,000. Actin muscle stains were conducted on day 7 animals when dissected from *QRas*^{V12}*scrib*^{RNAi} animals except when raised at 25°C and on day 8 animals when dissected from *Ras*^{V12}*dlg1*^{RNAi}. All other muscle and fat body staining were conducted at day 6 when dissected from *QRas*^{V12}*scrib*^{RNAi} animals and at day 7 when dissected from *Ras*^{V12}*dlg1*^{RNAi} animals (except when specified in the figure legend).

Western blotting

Fatbodies dissected from three wandering stage larvae (or day 6 larvae where tumour-bearing animals were used) were homogenised in RIPA lysis buffer containing EDTA-free cOmplete ULTRA protease inhibitor (Roche, #05892791001). Lysate protein concentrations were determined using the DC protein assay (BioRad, #5000112) and protein concentrations in all samples equalised before adding 10 mM DTT and Bolt LDS sample buffer (Novex, #B0007) to samples and then boiling at 70°C for 10 min. Samples were run in 12-well Bolt 4–12% Bis-Tris Plus precast gels (Invitrogen, #NW04122BOX) using Bolt MOPS SDS running buffer (Invitrogen, #B0001) and transferred to an Immobilon-FL PVDF membrane (Sigma-Aldrich, #IPFL00010) via wet transfer using transfer buffer (50 mM Tris, 38 mM Glycine, 20% ethanol). Membranes were blocked in 5% skim milk (w/v) diluted in TBS containing 0.2% Tween-20 (TBST), incubated with antibodies as per the manufacturer's specifications, developed using Amersham ECL Prime Western Blotting Reagent (Cytiva, #RPN2236) and imaged on the iBright 1,500 (Invitrogen). Primary antibodies used: pMad (1:1,000, Cell Signalling, #9516). Secondary antibodies used: anti-Mouse HRP (1:10,000, Jackson ImmunoResearch Labs, #115-035-003), anti-Rabbit HRP (1:10,000, Jackson ImmunoResearch Labs, #111-035-003).

Image analysis

All images were quantified using ImageJ. In wildtype fat body and muscle samples pMad intensity was normalised to DAPI, pMad and DAPI levels were quantified by drawing a circle around the nucleus in the DAPI channel, and the mean grey value (m.g.v.) determined for pMad and DAPI channels. In tumour bearing fat body, pMad, pAkt and OPP were not normalised to DAPI levels, as fat body tissue penetrance is altered in the presence of tumour. Here, the ROI circle was drawn around the nucleus in the pMad, pAkt or OPP channel. To measure fluorescence intensity of Vkg-GFP and Ndg, a line was drawn around membrane of a single cell in the fat body or along the edge of a single muscle segment in fillets on the z-plane where fluorescence was most intense, the line made to be five pixels wide, and m.g.v. determined along the line. %muscle/cuticle was determined using ImageJ as previously described (Lodge *et al*, 2021; Dark *et al*, 2022). For muscle atrophy measurements, muscle length and width measurements were done on the VL3 muscle on tiled 10X images of individual fillets using FIJI software with the line selection tool.

qPCR

For each biological replicate, three to five larvae were selected at day 6 (for fat body samples). Samples were dissected in cold PBS and snap-frozen in liquid nitrogen. Frozen tissue was then lysed in 300 µL of TRI Reagent (Invitrogen, #10296010) Total RNA was extracted using a Direct-zol RNA Microprep Kit (Zymo Research, #R2061). cDNA was obtained by reverse transcription of 0.5 µg of total RNA using ProtoScript II First Strand cDNA Synthesis Kit (NEB, #E6560S). Three independent biological replicates were prepared for each genotype. qPCR was performed with the Fast SYBR™ Green PCR Master Mix (Applied Biosystems, #4385612) on a LightCycler 480 (Roche). Gene expression was normalised to the geometric mean of the reference gene *rpl32*.

TCGA tumour data analyses

mRNA expression data for CHRD and BMP7 were downloaded for 24 TCGA studies from cBioPortal on 3rd August 2022. A total of 9,409 samples had RSEM data that had been batch normalised for both genes, and Spearman correlation was performed.

Statistical analysis

Hemolymph analysis was done using Limma. All other statistical analyses were conducted using GraphPad Prism. At least three animals per genotype were used for all muscle and fat body experiments. For staining intensity quantifications, individual data points represent fluorescence intensity of a single cell. For muscle integrity quantifications, individual data points represent a single larva. Data in Fig 4H was confirmed to be normally distributed using the Shapiro–Wilk test. For experiments with two genotypes or treatments, two-tailed unpaired student's *t*-tests were used to test for significant differences. The Welch's correction was applied in cases of unequal variances. For experiments with more than two genotypes, significant differences between specific genotypes were tested using a one-way ANOVA and a subsequent Šidák *post-hoc* test. The results

 © 2023 The Authors

for all *post-hoc* tests conducted in each analysis are shown in graphs. For all graphs, error bars represent SEM. P and adjusted-P values are reported as follows: $P > 0.05$, ns (not significant); $P < 0.05$, *; $P < 0.01$, **; $P < 0.001$, ***; $P < 0.0001$, ****.

Data availability

These data include no data deposited in external repositories.

Expanded View for this article is available online.

Acknowledgements

We are grateful to Kieran Harvey, Helena Richardson, Gary Hime, Anne Holz, Travis Johnson, Christen Mirth, Maurice Ringuette and Ana Traven for generous sharing of fly stocks, yeast stock and antibodies. We would like to thank Bloomington Drosophila Stock Center, Vienna Drosophila Resource Center and Developmental Studies Hybridoma Bank for fly stocks and antibodies. We would like to also thank OZDros for *Drosophila* quarantine, Peter MacCallum Cancer Institute CAHM for microscopy assistance. We are grateful to Kellie Veen and Khanh Phuong Nguyen for assistance with graphics production. LYC's laboratory is supported by funding from the NHMRC Ideas Grant (APP1182847), and the Peter MacCallum Cancer Foundation (APP2218).

Author contributions

Daniel Bakopoulos: Conceptualization; data curation; formal analysis; investigation; methodology; writing – original draft; writing – review and editing. **Sofya Golenkina:** Conceptualization; data curation; formal analysis; investigation; methodology; writing – review and editing. **Callum Dark:** Conceptualization; data curation; formal analysis; investigation; methodology; writing – review and editing. **Elizabeth L Christie:** Data curation. **Besaiz J Sánchez-Sánchez:** Resources. **Brian M Stramer:** Resources.
Louise Y Cheng: Conceptualization; resources; data curation; formal analysis; supervision; funding acquisition; investigation; methodology; writing – original draft; project administration; writing – review and editing.

Disclosure and competing interests statement

The authors declare that they have no conflict of interest.

References

Alves MJ, Figuerêdo RG, Azevedo FF, Cavallaro DA, Neto NIP, Lima JDC, Matos-Neto E, Radloff K, Riccardi DM, Camargo RG *et al* (2017) Adipose tissue fibrosis in human cancer cachexia: the role of TGFβ pathway. *BMC Cancer* 17: 190

Ballard SL, Jarolimova J, Wharton KA (2010) Gbb/BMP signaling is required to maintain energy homeostasis in *Drosophila*. *Dev Biol* 337: 375–385

Baracos VE, Martin L, Korc M, Guttridge DC, Fearon KCH (2018) Cancer-associated cachexia. *Nat Rev Dis Primers* 4: 1–18

Biehs B, François V, Bier E (1996) The *Drosophila* short gastrulation gene prevents Dpp from autoactivating and suppressing neurogenesis in the neuroectoderm. *Genes Dev* 10: 2922–2934

Birnbaum A, Wu X, Tatar M, Liu N, Bai H (2019) Age-dependent changes in transcription factor FOXO targeting in female *Drosophila*. *Front Genet* 10: 312

Brogiolo W, Stocker H, Ikeya T, Rintelen F, Fernandez R, Hafen E (2001) An evolutionarily conserved function of the *Drosophila* insulin receptor and insulin-like peptides in growth control. *Curr Biol* 11: 213–221

Caldwell PE, Walkiewicz M, Stern M (2005) Ras activity in the *Drosophila* prothoracic gland regulates body size and developmental rate via ecdysone release. *Curr Biol* 15: 1785–1795

Cerami E, Gao J, Dogrusoz U, Gross BE, Sumer SO, Aksoy BA, Jacobsen A, Byrne CJ, Heuer ML, Larsson E *et al* (2012) The cBio cancer genomics portal: an open platform for exploring multidimensional cancer genomics data. *Cancer Discov* 2: 401–404

Cheng LY, Bailey AP, Leevers SJ, Ragan TJ, Driscoll PC, Gould AP (2011) Anaplastic lymphoma kinase spares organ growth during nutrient restriction in *Drosophila*. *Cell* 146: 435–447

Clark JF, Ciccarelli EJ, Kayastha P, Ranepura G, Yamamoto KK, Hasan MS, Madaan U, Meléndez A, Savage-Dunn C (2021) BMP pathway regulation of insulin signaling components promotes lipid storage in *Caenorhabditis elegans*. *PLoS Genet* 17: e1009836

Colombani J, Bianchini L, Layalle S, Pondeville E, Dauphin-Villemant C, Antoniewski C, Carré C, Noselli S, Léopold P (2005) Antagonistic actions of ecdysone and insulins determine final size in *Drosophila*. *Science* 310: 667–670

Dai J, Ma M, Feng Z, Pastor-Pareja JC (2017) Inter-adipocyte adhesion and signaling by collagen IV intercellular concentrations in *Drosophila*. *Curr Biol* 27: 2729–2740

Dai J, Estrada B, Jacobs S, Sánchez-Sánchez BJ, Tang J, Ma M, Magadán-Corpas P, Pastor-Pareja JC, Martín-Bermudo MD (2018) Dissection of nidogen function in *Drosophila* reveals tissue-specific mechanisms of basement membrane assembly. *PLoS Genet* 14: e1007483

Dark C, Cheung S, Cheng LY (2022) Analyzing cachectic phenotypes in the muscle and fat body of *Drosophila* larvae. *STAR Protoc* 3: 101230

Das SK, Eder S, Schauer S, Diwoky C, Temmel H, Guertl B, Gorkiewicz G, Tamilarasan KP, Kumari P, Trauner M *et al* (2011) Adipose triglyceride lipase contributes to cancer-associated cachexia. *Science* 333: 233–238

Ding G, Xiang X, Hu Y, Xiao G, Chen Y, Binari R, Comjean A, Li J, Rushworth E, Fu Z *et al* (2021) Coordination of tumor growth and host wasting by tumor-derived Upd3. *Cell Rep* 36: 109553

Dong J, Yu J, Li Z, Gao S, Wang H, Yang S, Wu L, Lan C, Zhao T, Gao C *et al* (2021) Serum insulin-like growth factor binding protein 2 levels as biomarker for pancreatic ductal adenocarcinoma-associated malnutrition and muscle wasting. *J Cachexia Sarcopenia Muscle* 12: 704–716

Duncan S, Delage S, Chioran A, Sirbu O, Brown TJ, Ringuette MJ (2020) The predicted collagen-binding domains of *Drosophila* SPARC are essential for survival and for collagen IV distribution and assembly into basement membranes. *Dev Biol* 461: 197–209

Eivers E, McCarthy K, Glynn C, Nolan CM, Byrnes L (2004) Insulin-like growth factor (IGF) signalling is required for early dorso-anterior development of the zebrafish embryo. *Int J Dev Biol* 48: 1131–1140

Figueroa-Clarevega A, Bilder D (2015) Malignant *Drosophila* tumors interrupt insulin signaling to induce cachexia-like wasting. *Dev Cell* 33: 47–55

Gao J, Aksoy BA, Dogrusoz U, Dresdner G, Gross B, Sumer SO, Sun Y, Jacobsen A, Sinha R, Larsson E *et al* (2013) Integrative analysis of complex cancer genomics and clinical profiles using the cBioPortal. *Sci Signal* 6: pl1

Gesualdi SC, Haerry TE (2007) Distinct signaling of *Drosophila* activin/TGF-beta family members. *Fly (Austin)* 1: 212–221

Giannakou ME, Partridge L (2007) Role of insulin-like signalling in *Drosophila* lifespan. *Trends Biochem Sci* 32: 180–188

Graca FA, Rai M, Hunt LC, Stephan A, Wang Y-D, Gordon B, Wang R, Quarato G, Xu B, Fan Y *et al* (2022) The myokine Fibcd1 is an endogenous determinant of myofiber size and mitigates cancer-induced myofiber atrophy. *Nat Commun* 13: 2370

Hattori Y, Usui T, Satoh D, Moriyama S, Shimono K, Itoh T, Shirahige K, Uemura T (2013) Sensory-neuron subtype-specific transcriptional

programs controlling dendrite morphogenesis: genome-wide analysis of Abrupt and Knot/Collier. *Dev Cell* 27: 530 – 544
Hodgson JA, Parvy J-P, Yu Y, Vidal M, Cordero JB (2021) *Drosophila* larval models of invasive tumorigenesis for *in vivo* studies on tumour/peripheral host tissue interactions during cancer cachexia. *Int J Mol Sci* 22: 8317
Honegger B, Galic M, Köhler K, Wittwer F, Brogiolo W, Hafen E, Stocker H (2008) Imp-L2, a putative homolog of vertebrate IGF-binding protein 7, counteracts insulin signaling in *Drosophila* and is essential for starvation resistance. *J Biol* 7: 10
Hong S-H, Kang M, Lee K-S, Yu K (2016) High fat diet-induced TGF-β/Gbb signaling provokes insulin resistance through the tribbles expression. *Sci Rep* 6: 30265
Isabella AJ, Horne-Badovinac S (2016) Rab10-mediated secretion synergizes with tissue movement to build a polarized basement membrane architecture for organ morphogenesis. *Dev Cell* 38: 47 – 60
Khezri R, Holland P, Schoborg TA, Abramovich I, Takáts S, Dillard C, Jain A, O'Farrell F, Schultz SW, Hagopian WM *et al* (2021) Host autophagy mediates organ wasting and nutrient mobilization for tumor growth. *EMBO J* 40: e107336
Kim M-J, O'Connor MB (2021) *Drosophila* Activin signaling promotes muscle growth through InR/TORC1-dependent and -independent processes. *Development* 148: dev190868
Kwon Y, Song W, Droujinine IA, Hu Y, Asara JM, Perrimon N (2015) Systemic organ wasting induced by localized expression of the secreted insulin/IGF antagonist ImpL2. *Dev Cell* 33: 36 – 46
Lee J, Ng KG-L, Dombek KM, Eom DS, Kwon YV (2021) Tumors overcome the action of the wasting factor ImpL2 by locally elevating Wnt/Wingless. *Proc Natl Acad Sci USA* 118: e2020120118
Leevers SJ, Weinkove D, MacDougall LK, Hafen E, Waterfield MD (1996) The *Drosophila* phosphoinositide 3-kinase Dp110 promotes cell growth. *EMBO J* 15: 6584 – 6594
Lodge W, Zavortink M, Golenkina S, Froldi F, Dark C, Cheung S, Parker BL, Blazev R, Bakopoulos D, Christie EL *et al* (2021) Tumor-derived MMPs regulate cachexia in a *Drosophila* cancer model. *Dev Cell* 56: 2664 – 2680
Manent J, Banerjee S, de Matos Simoes R, Zoranovic T, Mitsiades C, Penninger JM, Simpson KJ, Humbert PO, Richardson HE (2017) Autophagy suppresses Ras-driven epithelial tumourigenesis by limiting the accumulation of reactive oxygen species. *Oncogene* 36: 5576 – 5592
McBrayer Z, Ono H, Shimell M, Parvy J-P, Beckstead RB, Warren JT, Thummel CS, Dauphin-Villemant C, Gilbert LI, O'Connor MB (2007) Prothoracicotropic hormone regulates developmental timing and body size in *Drosophila*. *Dev Cell* 13: 857 – 871
Mirth C, Truman JW, Riddiford LM (2005) The role of the prothoracic gland in determining critical weight for metamorphosis in *Drosophila melanogaster*. *Curr Biol* 15: 1796 – 1807
Moraes LN, Fernandez GJ, Vechetti-Júnior IJ, Freire PP, Souza RWA, Villacis RAR, Rogatto SR, Reis PP, Dal-Pai-Silva M, Carvalho RF (2017) Integration of miRNA and mRNA expression profiles reveals microRNA-regulated networks during muscle wasting in cardiac cachexia. *Sci Rep* 7: 6998
Newton H, Wang Y-F, Camplese L, Mokochinski JB, Kramer HB, Brown AEX, Fets L, Hirabayashi S (2020) Systemic muscle wasting and coordinated tumour response drive tumourigenesis. *Nat Commun* 11: 4653
Ni X, Yang J, Li M (2012) Imaging-guided curative surgical resection of pancreatic cancer in a xenograft mouse model. *Cancer Lett* 324: 179 – 185
Parker J, Struhl G (2015) Scaling the *Drosophila* wing: TOR-dependent target gene access by the Hippo pathway transducer Yorkie. *PLoS Biol* 13: e1002274
Parkin CA, Burnet B (1986) Growth arrest of *Drosophila melanogaster* on erg-2 and erg-6 sterol mutant strains of *Saccharomyces cerevisiae*. *J Insect Physiol* 32: 463 – 471
Pastor-Pareja JC, Xu T (2011) Shaping cells and organs in *Drosophila* by opposing roles of fat body-secreted collagen IV and perlecan. *Dev Cell* 21: 245 – 256
Peterson AJ, Jensen PA, Shimell M, Stefancsik R, Wijayatonge R, Herder R, Raftery LA, O'Connor MB (2012) R-Smad competition controls activin receptor output in *Drosophila*. *PloS One* 7: e36548
Portela M, Casas-Tinto S, Rhiner C, López-Gay JM, Domínguez O, Soldini D, Moreno E (2010) *Drosophila* SPARC is a self-protective signal expressed by loser cells during cell competition. *Dev Cell* 19: 562 – 573
Santabárbara-Ruiz P, Léopold P (2021) An Oatp transporter-mediated steroid sink promotes tumor-induced cachexia in *Drosophila*. *Dev Cell* 56: 2741 – 2751
Semaniuk U, Piskovatska V, Strilbytska O, Strutynska T, Burdyliuk N, Vaiserman A, Bubalo V, Storey KB, Lushchak O (2021) *Drosophila* insulin-like peptides: from expression to functions – a review. *Entomol Exp Appl* 169: 195 – 208
Shahab J, Baratta C, Scuric B, Godt D, Venken KJT, Ringuette MJ (2015) Loss of SPARC dysregulates basal lamina assembly to disrupt larval fat body homeostasis in *Drosophila melanogaster*. *Dev Dyn* 244: 540 – 552
Song W, Kir S, Hong S, Hu Y, Wang X, Binari R, Tang H-W, Chung V, Banks AS, Spiegelman B *et al* (2019) Tumor-derived ligands trigger tumor growth and host wasting via differential MEK activation. *Dev Cell* 48: 1 – 17
Tiebe M, Lutz M, De La Garza A, Buechling T, Boutros M, Teleman AA (2015) REPTOR and REPTOR-BP regulate organismal metabolism and transcription downstream of TORC1. *Dev Cell* 33: 272 – 284
Urrutia H, Aleman A, Eivers E (2016) *Drosophila* Dullard functions as a Mad phosphatase to terminate BMP signaling. *Sci Rep* 6: 32269
Van De Bor V, Zimniak G, Papone L, Cerezo D, Malbouyres M, Juan T, Ruggiero F, Noselli S (2015) Companion blood cells control ovarian stem cell niche microenvironment and homeostasis. *Cell Rep* 13: 546 – 560
Weinkove D, Neufeld TP, Twardzik T, Waterfield MD, Leevers SJ (1999) Regulation of imaginal disc cell size, cell number and organ size by *Drosophila* class IA phosphoinositide 3-kinase and its adaptor. *Curr Biol* 9: 1019 – 1029
Yoo S, Nair S, Kim H, Kim Y, Lee C, Lee G, Park JH (2020) Knock-in mutations of scarecrow, a *Drosophila* homolog of mammalian Nkx2.1, reveal a novel function required for development of the optic lobe in *Drosophila melanogaster*. *Dev Biol* 461: 145 – 159
Yu K, Srinivasan S, Shimmi O, Biehs B, Rashka KE, Kimelman D, O'Connor MB, Bier E (2000) Processing of the *Drosophila* Sog protein creates a novel BMP inhibitory activity. *Development* 127: 2143 – 2154
Zang Y, Wan M, Liu M, Ke H, Ma S, Liu L-P, Ni J-Q, Carlos Pastor-Pareja J (2015) Plasma membrane overgrowth causes fibrotic collagen accumulation and immune activation in *Drosophila* adipocytes. *Elife* 4: e07187
Zhang H, Stallock JP, Ng JC, Reinhard C, Neufeld TP (2000) Regulation of cellular growth by the *Drosophila* target of rapamycin dTOR. *Genes Dev* 14: 2712 – 2724
Zhang Y, Billington CJ, Pan D, Neufeld TP (2006) *Drosophila* target of rapamycin kinase functions as a multimer. *Genetics* 172: 355 – 362

License: This is an open access article under the terms of the Creative Commons Attribution License, which permits use, distribution and reproduction in any medium, provided the original work is properly cited.

© 2023 The Authors

Expanded View Figures

Figure EV1. Tumour secreted Gbb and ImpL2 rescue muscle integrity additively.

A–D Muscle fillets of *Ras*^{V12}*dlg1*^{RNAi} tumour-bearing animals that express in the tumour *lacZ*^{RNAi}; *mCherry*^{RNAi} or *ImpL2*^{RNAi}; *lacZ*^{RNAi}, or *gbb*^{RNAi}; *mCherry*^{RNAi} or *ImpL2*^{RNAi}; *gbb*^{RNAi}.

E Quantification of muscle detachment in (A–D). *lacZ*^{RNAi}; *mCherry*^{RNAi} ($n = 8$), *ImpL2*^{RNAi}; *lacZ*^{RNAi} ($n = 11$), *gbb*^{RNAi}; *mCherry*^{RNAi} ($n = 8$), *ImpL2*^{RNAi}; *gbb*^{RNAi} ($n = 16$).

F, G OPP staining detecting protein translation in the fat body of ω^{1118} and *Ras*^{V12}*dlg*^{RNAi} tumour-bearing animals.

H Quantification of OPP in (F, G). ω^{1118} ($n = 19$) *Ras*^{V12}*dlg1*^{RNAi} ($n = 26$)

I–L OPP staining detecting protein translation in the fat body of *Ras*^{V12}*dlg1*^{RNAi} tumour-bearing animals, that express in the tumour *lacZ*^{RNAi}; *mCherry*^{RNAi} or *ImpL2*^{RNAi}; *lacZ*^{RNAi}, or *gbb*^{RNAi}; *mCherry*^{RNAi} or *ImpL2*^{RNAi}; *gbb*^{RNAi}.

M Quantification of OPP in (I–L). ω^{1118} ($n = 10$); *LacZ*^{RNAi}; *mCherry*^{RNAi} ($n = 22$), *ImpL2*^{RNAi}; *lacZ*^{RNAi} ($n = 13$), *gbb*^{RNAi}; *mCherry*^{RNAi} ($n = 15$), *ImpL2*^{RNAi}; *gbb*^{RNAi} ($n = 14$).

N, O Nidogen staining detecting ECM localisation in the fat body of ω^{1118} and *Ras*^{V12}*dlg1*^{RNAi} animals.

P Quantification of nidogen staining in (N, O). ω^{1118} ($n = 23$) *Ras*^{V12}*dlg1*^{RNAi} ($n = 20$).

Q–T *Ras*^{V12}*dlg1*^{RNAi} tumour-bearing animals that express *lacZ*^{RNAi}; *mCherry*^{RNAi} or *ImpL2*^{RNAi}; *lacZ*^{RNAi}, or *gbb*^{RNAi}; *mCherry*^{RNAi} or *ImpL2*^{RNAi}; *gbb*^{RNAi}.

U Quantification of Nidogen in (Q, T). ω^{1118} ($n = 9$); *LacZ*^{RNAi}; *mCherry*^{RNAi} ($n = 17$), *ImpL2*^{RNAi}; *lacZ*^{RNAi} ($n = 22$), *gbb*^{RNAi}; *mCherry*^{RNAi} ($n = 9$), *ImpL2*^{RNAi}; *gbb*^{RNAi} ($n = 9$).

Data information: Scale bar is 50 μm for fat body staining (F, G, I–L, N, O, Q–T) and 500 μm for muscle fillets (A–D). Graphs are represented as Mean ± SEM, n = the number of samples. (*) $P < 0.05$ (**) $P < 0.01$, (***) $P < 0.001$, (****) $P < 0.0001$. For experiments with two genotypes, two-tailed unpaired student's *t*-tests were used to test for significant differences. The Welch's correction was applied in cases of unequal variances. For experiments with more than two genotypes, significant differences between specific genotypes were tested using a one-way ANOVA and a subsequent Šidák *post-hoc* test.

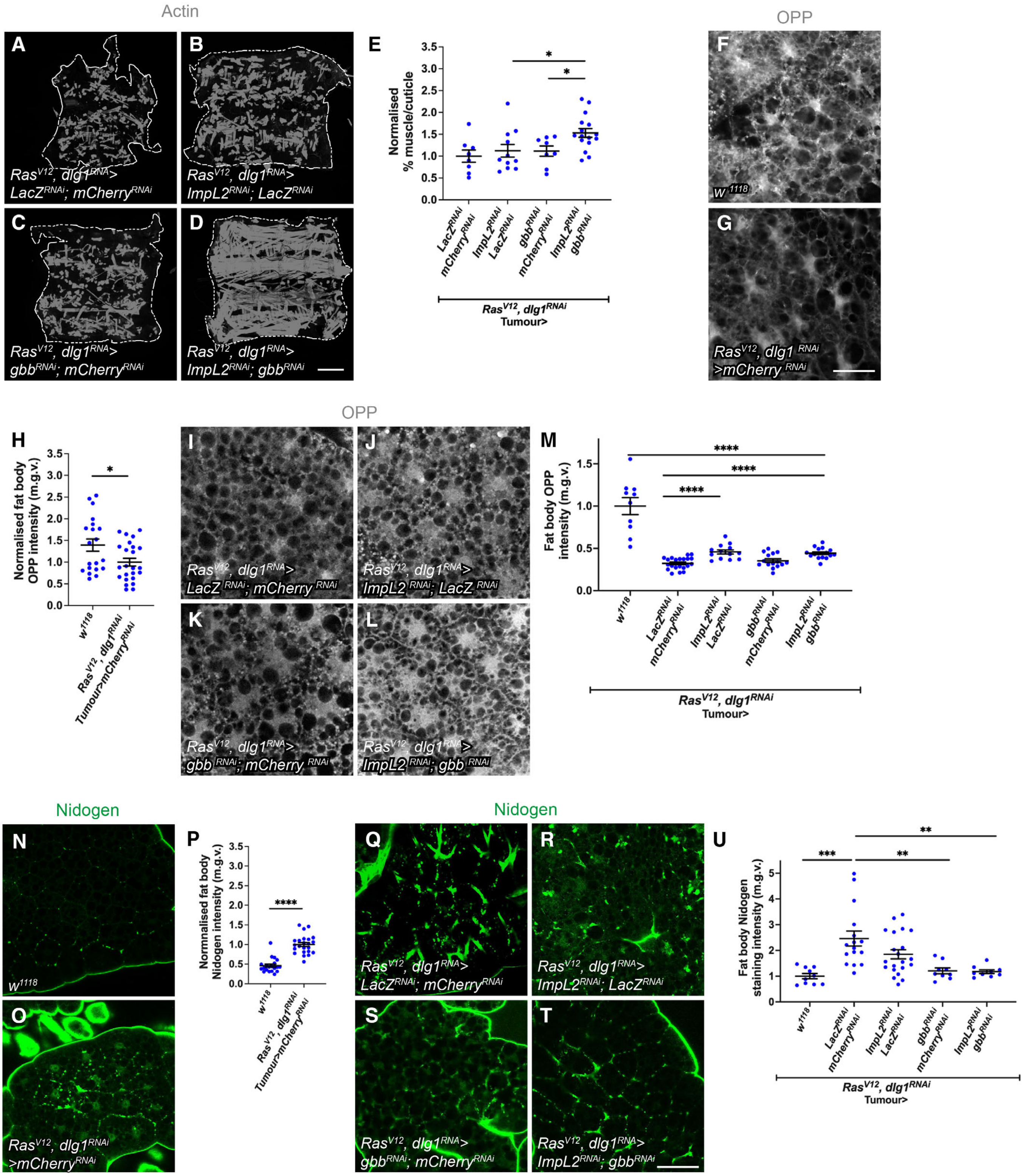

Figure EV1.

© 2023 The Authors

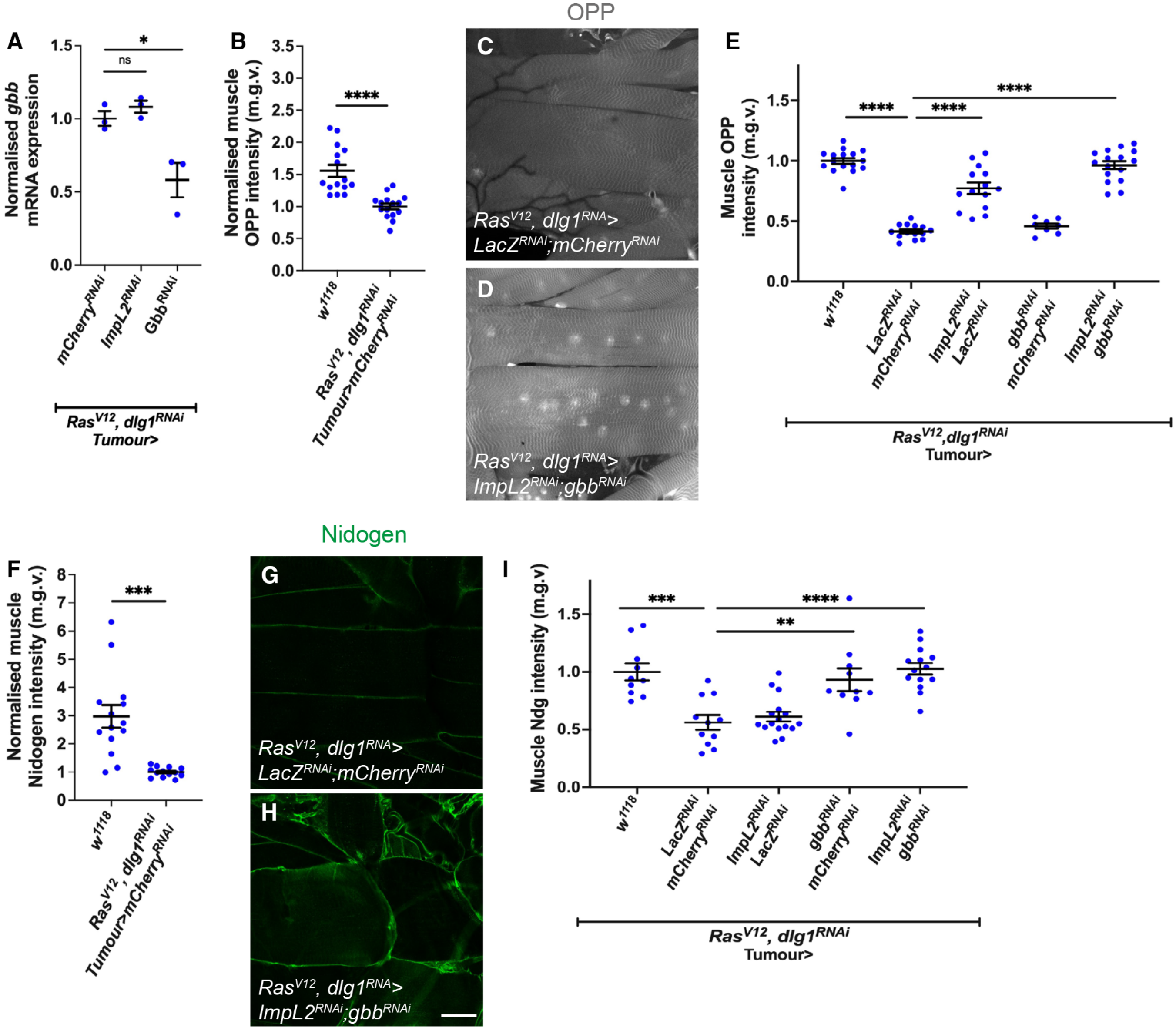

Figure EV2. Knockdown of tumour-derived Gbb and ImpL2 rescues muscle OPP and ECM protein Nidogen.

A Tumour qPCRs showing mRNA expression levels of *gbb* in *$Ras^{V12}dlg1^{RNAi}$* larvae expressing *$mCherry^{RNAi}$*, *$ImpL2^{RNAi}$*, *Gbb^{RNAi}* in the tumour (n = 3).

B Quantification of normalised muscle OPP intensity in w^{1118} and *$Ras^{V12}dlg1^{RNAi}$* tumour-bearing animals, where *$mCherry^{RNAi}$* is specifically expressed in the tumour. w^{1118} (n = 15) and *$Ras^{V12}dlg1^{RNAi}$* (n = 15).

C, D OPP staining detecting protein translation in the muscles of *$Ras^{V12}dlg1^{RNAi}$* tumour-bearing animals that express *$lacZ^{RNAi}$; $mCherry^{RNAi}$* or *$ImpL2^{RNAi}$; gbb^{RNAi}*.

E Quantification of OPP in the muscles of wild type w^{1118} (n = 18) and tumour-bearing animals expressing *$LacZ^{RNAi}$;$mCherry^{RNAi}$* (n = 16), *$ImpL2^{RNAi}$; $lacZ^{RNAi}$* (n = 14), *gbb^{RNAi}; $mCherry^{RNAi}$* (n = 8), *$ImpL2^{RNAi}$; gbb^{RNAi}* (n = 15).

F Quantification of normalised muscle Nidogen intensity in w^{1118} and *$Ras^{V12}dlg1^{RNAi}$* tumour-bearing animals, where *$mcherry^{RNAi}$* is specifically expressed in the tumour. w^{1118} (n = 14) and *$Ras^{V12}dlg1^{RNAi}$* (n = 13)

G, H Nidogen staining detecting ECM localisation in the muscles of *$Ras^{V12}dlg1^{RNAi}$* tumour-bearing animals that express *$lacZ^{RNAi}$; $mCherry^{RNAi}$* or *$ImpL2^{RNAi}$;gbb^{RNAi}*.

I Quantification of Nidogen in wild type w^{1118} (n = 10) and tumour-bearing animals expressing *$LacZ^{RNAi}$;$mCherry^{RNAi}$* (n = 11), *$ImpL2^{RNAi}$; $lacZ^{RNAi}$* (n = 16), *gbb^{RNAi}; $mCherry^{RNAi}$* (n = 10), *$ImpL2^{RNAi}$; gbb^{RNAi}* (n = 14).

Data information: Scale bar is 50 μm. Graphs are represented as Mean ± SEM, n = the number of samples. (*) $P < 0.05$ (**) $P < 0.01$, (***) $P < 0.001$, (****) $P < 0.0001$, (ns) $P > 0.05$. For experiments with two genotypes, two-tailed unpaired student's t-tests were used to test for significant differences. The Welch's correction was applied in cases of unequal variances. For experiments with more than two genotypes, significant differences between specific genotypes were tested using a one-way ANOVA and a subsequent Šidák *post-hoc* test.

© 2023 The Authors

Figure EV3. Muscle overexpression of *Akt* (but not fat body *Akt* or *mad RNAi* overexpression) rescues atrophy in cachectic animals, and TGF-ß signalling in the muscle is responsive to modulation in tumour insulin signalling but is not required for muscle integrity in cachexia.

A, B Muscle fillets stained with phalloidin (Actin) where *luciferase* or *Akt* was expressed under the control of *MHC-GAL4* in $QRas^{V12}scrib^{RNAi}$ tumour-bearing animals.
C Quantification of normalised muscle detachment in (A, B). Wilt type control ($MHC>mCherry^{RNAi}$, n = 3), *UAS-luciferase* (n = 10), *Akt* (n = 17).
D, E Muscle segment (outlined) where *luciferase* or *Akt* was expressed under the control of *MHC-GAL4* in $QRas^{V12}scrib^{RNAi}$ tumour-bearing animals.
F Quantification of normalised muscle width/length in (D, E). Wild type control (n = 15), *luciferase* (n = 9), *Akt* (n = 16).
G, H Muscle Nidogen staining where *luciferase* or *Akt* was expressed under the control of *MHC-GAL4* in $QRas^{V12}scrib^{RNAi}$ tumour-bearing animals.
I Quantification of normalised muscle Nidogen staining in (G, H). *luciferase* (n = 16), *Akt* (n = 15).
J, K Muscle segment from $QRas^{V12}scrib^{RNAi}$ tumour-bearing animals, where $mCherry^{RNAi}$ or *Akt* was expressed in the fat body (*r4-GAL4*).
L Quantification of normalised muscle width/length in (J, K). $r4>mCherry^{RNAi}$(n = 9), $QRas^{V12}scrib^{RNAi}$;$r4>mCherry^{RNAi}$ (n = 5), $QRas^{V12}scrib^{RNAi}$;$r4>Akt$ (n = 8).
M, N Muscle segment from $QRas^{V12}scrib^{RNAi}$ tumour-bearing animals, where $mCherry^{RNAi}$ or mad^{RNAi} was expressed in the fat body (*r4-GAL4*).
O Quantification of normalised muscle width/length in (M, N). $r4>mCherry^{RNAi}$(n = 11), $QRas^{V12}scrib^{RNAi}$; $r4>mCherry^{RNAi}$ (n = 18), $QRas^{V12}scrib^{RNAi}$; $r4>mad^{RNAi}$ (n = 15).
P Quantification of normalised muscle OPP intensity where *luciferase* (n = 10) or *Akt* (n = 16) was expressed under the control of *MHC-GAL4* in $QRas^{V12}scrib^{RNAi}$ tumour-bearing animals.
Q, R Muscle from $Ras^{V12}dlg1^{RNAi}$ tumour-bearing animals expressing either $mCherry^{RNAi}$ or $ImpL2^{RNAi}$ in the tumour, where TGF-ß signalling activation is indicated by pMad staining.
S Quantification of normalised muscle pMad intensity in (Q, R). w^{1118} (n = 30), $mCherry^{RNAi}$ (n = 30), $ImpL2^{RNAi}$ (n = 26).
T Quantification of normalised muscle detachment where $MHC>mCherry^{RNAi}$ (n = 5) or $mCherry^{RNAi}$ (n = 9) or $lacZ^{RNAi}$; mad^{RNAi} (n = 6) was expressed under the control of *MHC-GAL4* in $QRas^{V12}scrib^{RNAi}$ tumour-bearing animals.

Data information: Scale bar is 500 μm for muscle fillets (A, B), 100 μm for muscle segment atrophy measurements (D, E, J, K, M, N) and 50 μm for muscle Nidogen and pMad staining (G, H, Q, R). Muscles in tumour bearing animals were dissected at day 6 after tumour induction. Graphs are represented as Mean ± SEM, n = the number of samples. (*) $P < 0.05$ (**) $P < 0.01$, (***) $P < 0.001$, (****) $P < 0.0001$, (ns) $P > 0.05$. For experiments with two genotypes, two-tailed unpaired student's t-tests were used to test for significant differences. The Welch's correction was applied in cases of unequal variances. For experiments with more than two genotypes, significant differences between specific genotypes were tested using a one-way ANOVA and a subsequent Šidák *post-hoc* test.

 © 2023 The Authors

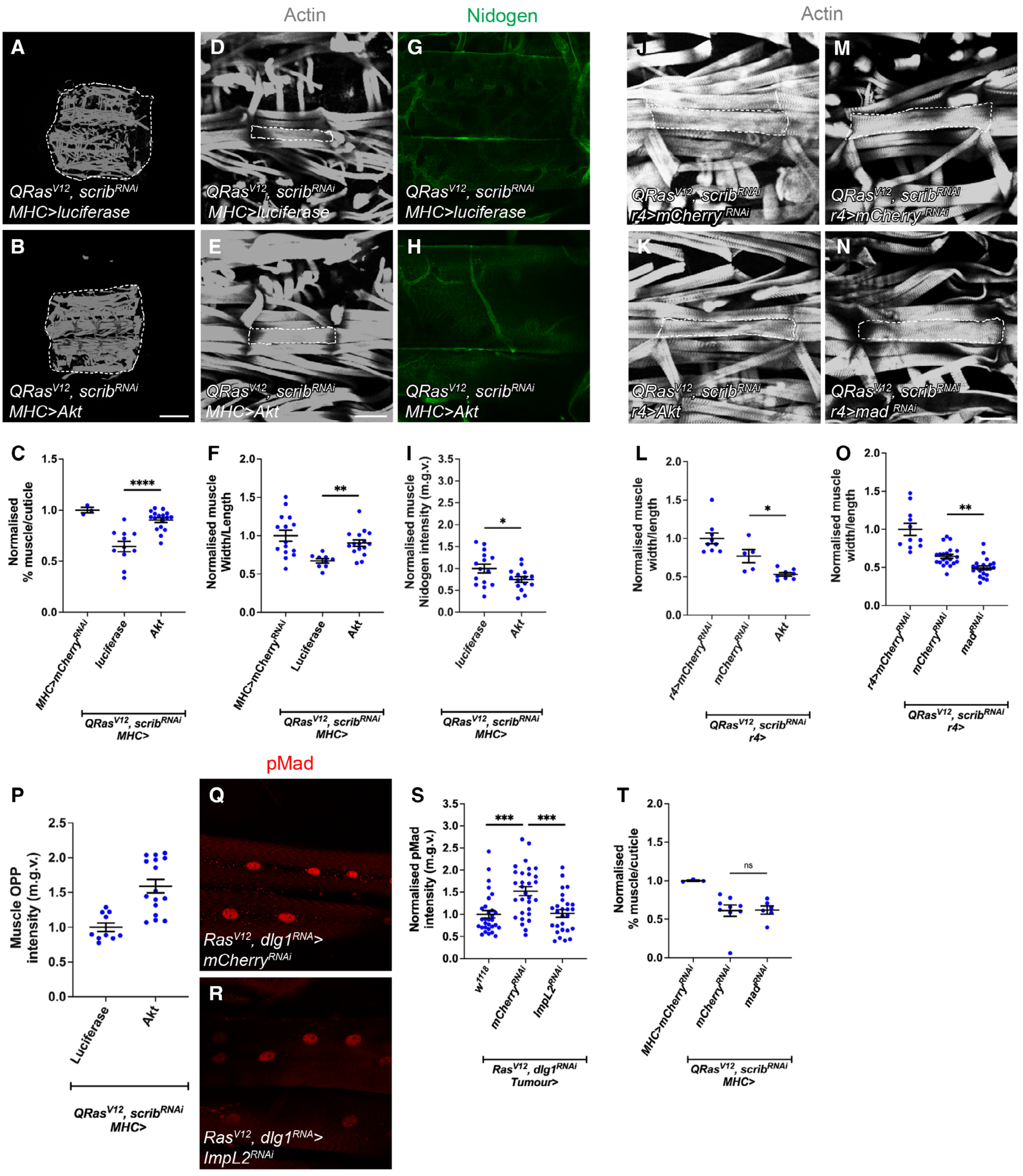

Actin
Nidogen
Actin
A
QRasV12, scribRNAi MHC>luciferase
B
QRasV12, scribRNAi MHC>Akt
D
QRasV12, scribRNAi MHC>luciferase
E
QRasV12, scribRNAi MHC>Akt
G
QRasV12, scribRNAi MHC>luciferase
H
QRasV12, scribRNAi MHC>Akt
J
QRasV12, scribRNAi r4>mCherry RNAi
M
QRasV12, scribRNAi r4>mCherry RNAi
K
QRasV12, scribRNAi r4>Akt
N
QRasV12, scribRNAi r4>mad RNAi
C
Normalised % muscle/cuticle
****
MHC>mCherryRNAi
luciferase
Akt
QRasV12, scribRNAi MHC>
F
Normalised muscle Width/Length
**
MHC>mCherryRNAi
Luciferase
Akt
QRasV12, scribRNAi MHC>
I
Normalised muscle Nidogen intensity (m.g.v.)
*
luciferase
Akt
QRasV12, scribRNAi MHC>
L
Normalised muscle width/length
*
r4>mCherryRNAi
mCherryRNAi
Akt
QRasV12, scribRNAi r4>
O
Normalised muscle width/length
**
r4>mCherryRNAi
mCherryRNAi
madRNAi
QRasV12, scribRNAi r4>
P
Muscle OPP intensity (m.g.v.)
Luciferase
Akt
QRasV12, scribRNAi MHC>
pMad
Q
RasV12, dlg1RNA> mCherryRNAi
R
RasV12, dlg1RNA> ImpL2RNAi
S
Normalised pMad intensity (m.g.v.)
***
***
w1118
mCherryRNAi
ImpL2RNAi
RasV12, dlg1RNAi Tumour>
T
Normalised % muscle/cuticle
ns
MHC>mCherryRNAi
mCherryRNAi
madRNAi
QRasV12, scribRNAi MHC>

Figure EV3.

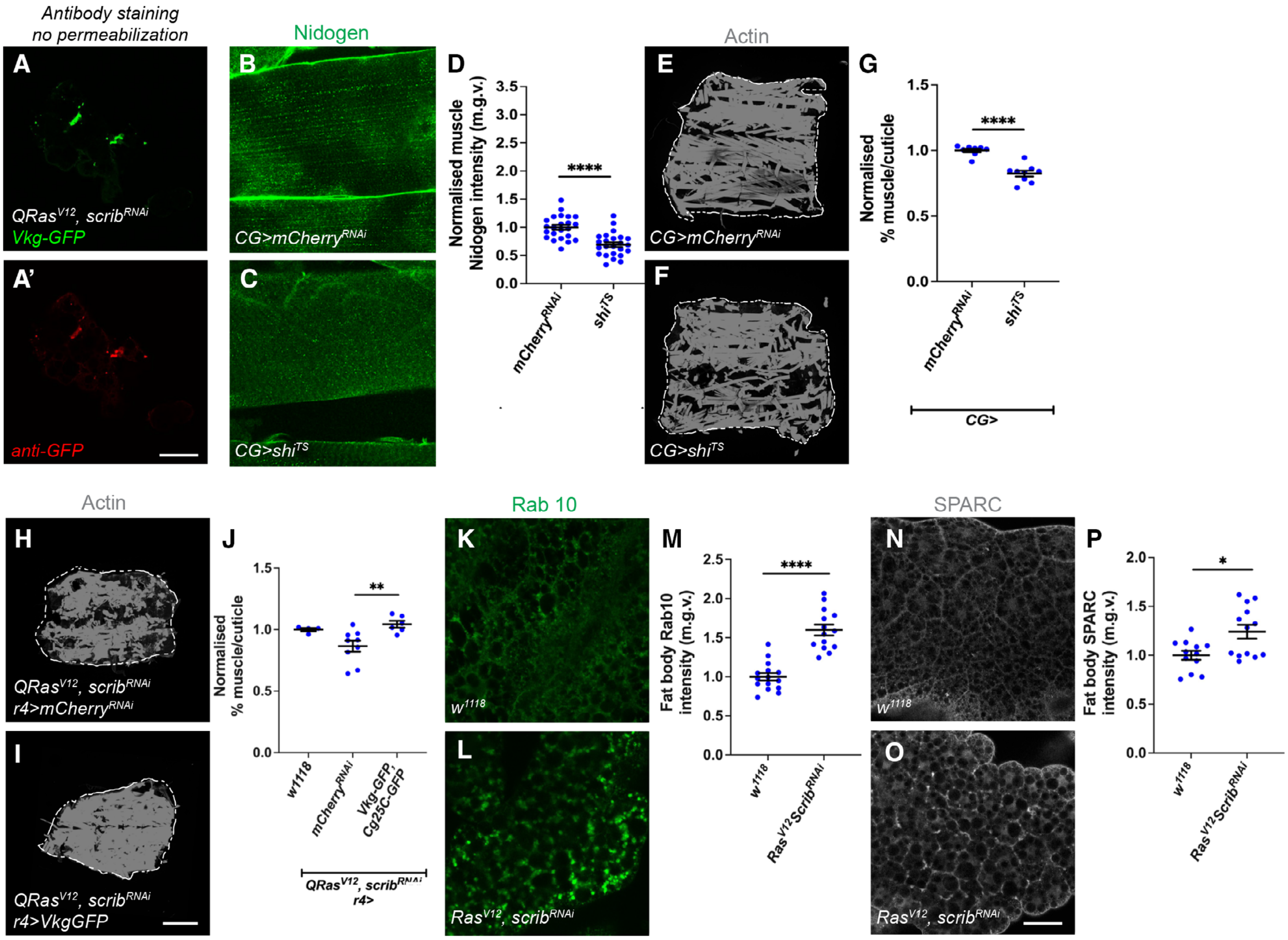

Figure EV4. Inhibition of fat body endocytosis causes muscle detachment, increasing Vkg expression in the fat body improves muscle integrity in tumour-bearing animals.

A, A' Antibody staining against GFP without permeabilisation can detect Vkg-GFP.

B, C Nidogen staining in muscles of animals where *mCherry*^{RNAi} or *shi*^{TS} was expressed in the fat body via *CG-GAL4*.

D Quantification of muscle nidogen staining in (B, C). *mCherry*^{RNAi} (n = 23), *shi*^{TS} (n = 24).

E, F Muscle fillets stained with phalloidin (Actin) from animals where *mCherry*^{RNAi} or *shi*^{TS} was expressed in the fat body via *CG-GAL4*.

G Quantification of normalised muscle detachment in (E, F). *mCherry*^{RNAi} (n = 8), *shi*^{TS} (n = 9).

H, I Muscle fillets stained with phalloidin (Actin) from *QRas*^{V12}*scrib*^{RNAi} tumour-bearing animals, where *mCherry*^{RNAi} or *UAS-VkgGFP;UAS-Cg25C-GFP* was expressed in the fat body (*r4-GAL4*). Day 6 muscles used here.

J Quantification of normalised muscle detachment in (H, I). ω^{1118} (n = 4), *QRas*^{V12}*scrib*^{RNAi}*;r4>mCherry*^{RNAi} (n = 9), *QRas*^{V12}*scrib*^{RNAi}*;r4>UAS-VkgGFP;UAS-Cg25C-GFP* (n = 6).

K, L Fat body staining for Rab10 in ω^{1118} and QRas^{V12}scrib^{RNAi} tumour-bearing animals.

M Quantification of normalised Rab10 levels in (K, L). ω^{1118} (n = 15), *QRas*^{V12}*scrib*^{RNAi} (n = 14).

N, O Fat body staining for SPARC in ω^{1118} and QRas^{V12}scrib^{RNAi} tumour-bearing animals.

P Quantification of normalised SPARC levels in (N, O). ω^{1118} (n = 12), *QRas*^{V12}*scrib*^{RNAi} (n = 13).

Data information: Scale bar is 500 µm for muscle fillets (E, F, H, I) 50 µm for muscle Nidogen and GFP staining (A, A', B, C) and 50 µm for fat body staining (K, L, N, O). Muscles in *Shi*^{TS} animals were dissected at day 5 ALH, muscles and fat body in tumour bearing animals were dissected at day 6 after tumour induction. Graphs are represented as Mean ± SEM, n = the number of samples. (*) $P < 0.05$ (**) $P < 0.01$, (****) $P < 0.0001$. For experiments with two genotypes, two-tailed unpaired student's t-tests were used to test for significant differences. The Welch's correction was applied in cases of unequal variances. For experiments with more than two genotypes, significant differences between specific genotypes were tested using a one-way ANOVA and a subsequent Šidák *post-hoc* test.

 © 2023 The Authors

Figure EV5. Modulating fat body TGF-ß signalling alters sparc and rab10 transcription, and modulating fat body InR alters sog transcription.

A Knockdown efficiency of *sog*^{RNAi} and *mad*^{RNAi} as indicated by qPCR and western blotting respectively. For qPCR, error bars represent SEM, $n = 3$ for biological replicates.

B Fat body qPCR showing normalised mRNA expression levels of SPARC and Rab10 upon the expression of *mCherry*^{RNAi} compared to *UAS-Mad*. Error bars represent SD, $n = 3$ for biological replicates.

C Fat body qPCRs showing normalised mRNA expression levels of TGF-ß receptors and ligands in *InR*^{CA} or *mCherry*^{RNAi} larvae (raised at 18°C) with *CG-GAL4* ($n = 3$, biological replicates). Error bars represent SD.

D Summary diagram—*Left*: during development, insulin signalling in the fat body activates the transcription of *sog*, which inhibits Gbb and prevents the activation of TGF-ß signalling in the fat body. This allows fat body ECM to be secreted to function in the muscle. Insulin signalling in the muscle in parallel enhances translation and muscle growth. *Right*: in tumour-bearing/cachectic animals, tumours secrete two ligands: ImpL2 and Gbb. In the fat body, ImpL2 inhibits insulin signalling, preventing the transcription of *sog* and thus Sog can no longer inhibit Gbb. In addition, tumour secreted Gbb binds to Tkv to activate TGF-ß signalling in the fat body, resulting in an accumulation of ECM proteins, to prevent ECM transport out of the fat body to reach the muscle. In the muscle, ImpL2 inhibits insulin signalling, which inhibits translation and muscle growth. Fat body in tumour bearing animals were dissected at day 6 after tumour induction. Fat body in non-tumour bearing animals were dissected at day 5 ALH.

Data information: (*) $P < 0.05$ (**) $P < 0.01$, (***) $P < 0.001$, (****) $P < 0.0001$. Two-tailed unpaired student's *t*-tests were used to test for significant differences. The Welch's correction was applied in cases of unequal variances.

© 2023 The Authors

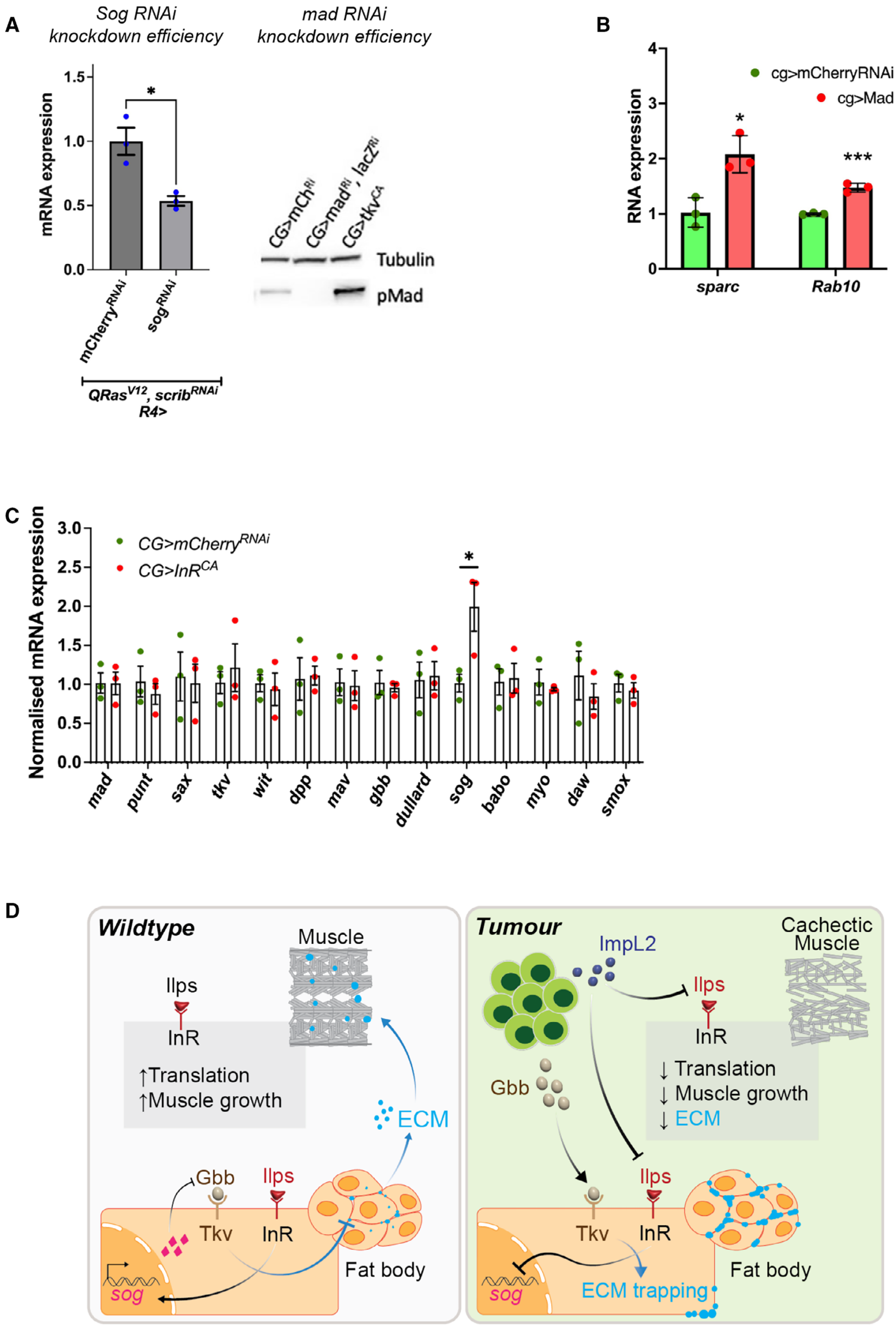

Figure EV5.

 © 2023 The Authors

Table of contents

Appendix Figure S1: Systemic ecdysone levels does not affect muscle detachment
Appendix Figure S2: Modulation of TGF-ß signalling does not affect insulin signalling in the muscle or wing imaginal discs
Appendix Figure S3: Pan-cancer expression of CHRD compared to BMP7.

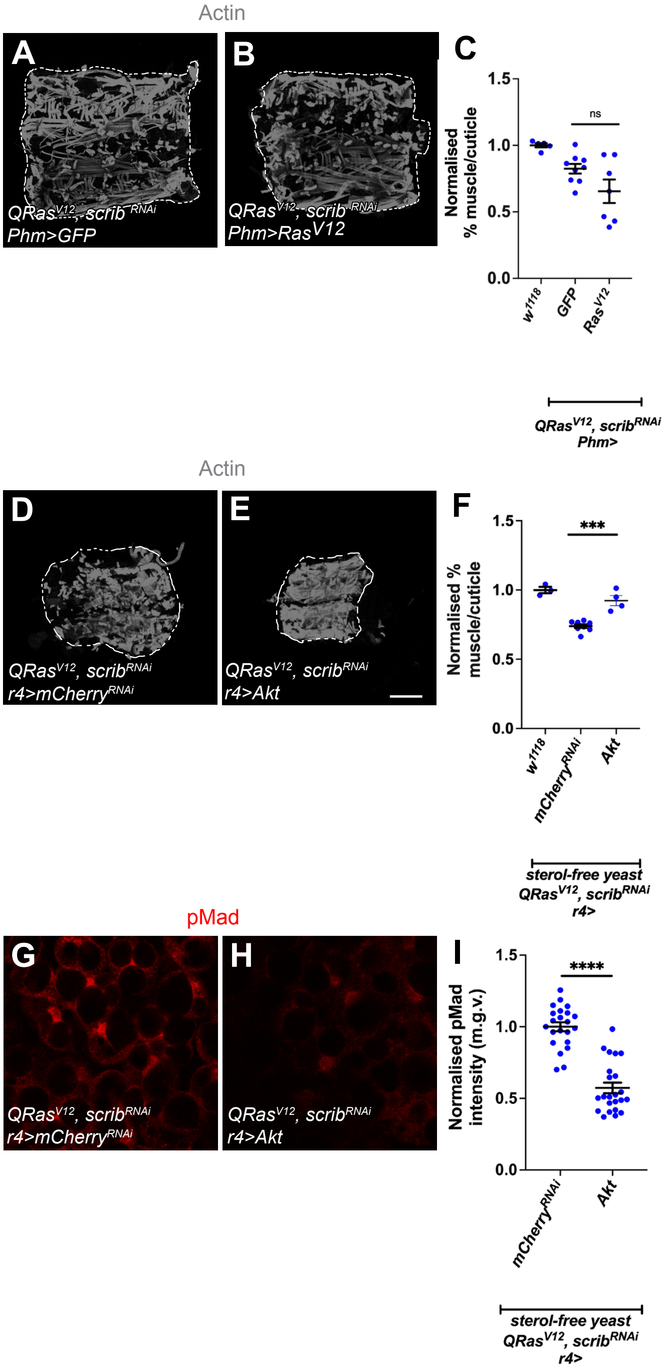

Appendix Figure S1: Systemic ecdysone levels does not affect muscle detachment

(A-B) Muscle fillets from animals where *GFP* or Ras^{V12} was expressed in the prothoracic gland (*Phm-GAL4*) of $QRas^{V12}scrib^{RNAi}$ tumour-bearing animals, exhibited similar amount of muscle detachment.

(C) Quantification of muscle detachment in A-B. w^{1118} (n=5), *GFP* (n=9), Ras^{v12} (n=7).

(D-E) Muscle fillets stained with phalloidin (Actin) from animals where *mCherry*^{RNAi} or *UAS-Akt* was specifically expressed in the fat body (*r4-GAL4*) of *QRas*^{V12}*scrib*^{RNAi} tumour-bearing animals fed on a sterol-free diet.

(F) Quantification of muscle detachment in D-E. *mCherry*^{RNAi} (n=6), *UAS-Akt* (n=10)

(G-H) Fat body from animals expressing *mCherry*^{RNAi} or UAS-*Akt* under the control of *r4-GAL4* in *QRas*^{V12}*scrib*^{RNAi} tumour-bearing animals fed on a sterol-free diet stained with pMad.

(I) Quantification of normalized fat body pMad intensity in G-H. *mCherry* ^{RNAi} (n=21), *UAS-Akt* (n=23).

Scale bar is 500µm for muscle fillets and 50µm for fat body staining.

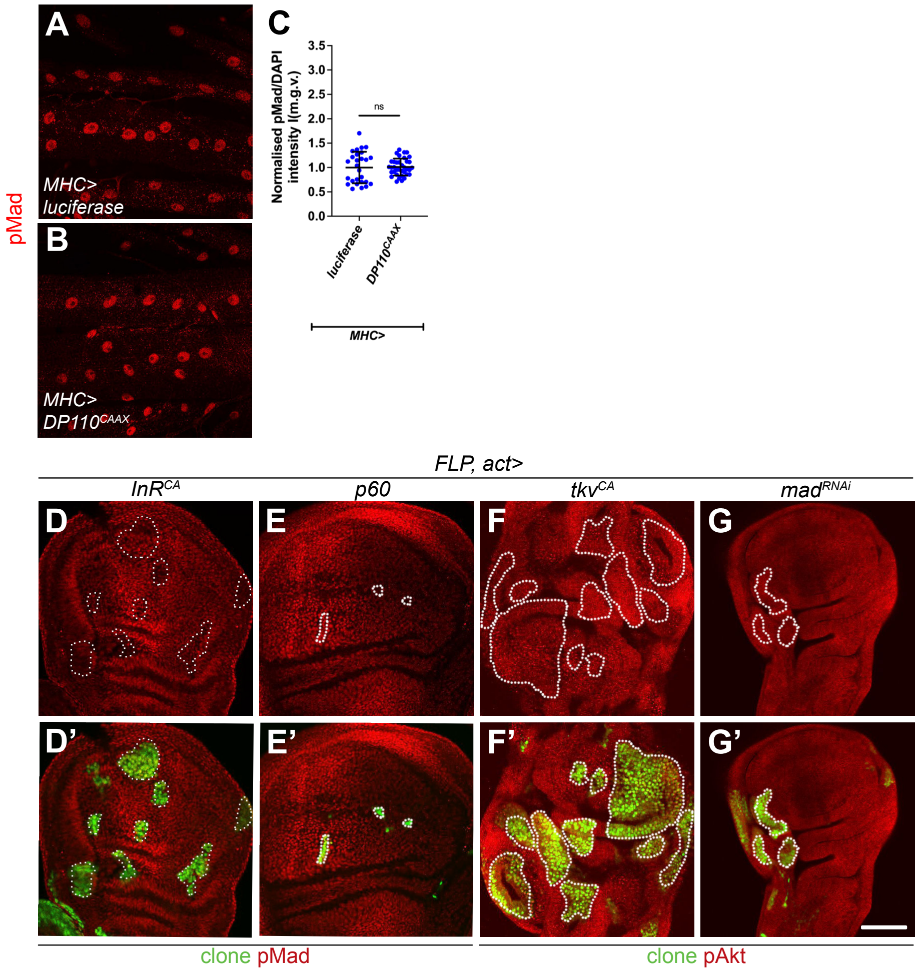

Appendix Figure S2: Modulation of TGF-ß signalling does not affect insulin signalling in the muscle or wing imaginal discs

(A-B) pMad staining in muscle fillets from larvae expressing *luciferase* or *DP110*^{CAAX} with *MHC-GAL4*.

(C) Quantification of pMad staining in A-B. *luciferase* (n=27), *DP110*^{CAAX} (n=36).

(D-D’) Heatshock induced flip-out clones (marked with GFP, circled with dotted lines) of *InR*^{CA} in the wing imaginal discs, readout of TGF-ß signalling levels indicated by pMad is not altered in the clones vs. surrounding tissues.

(E-E’) Heatshock induced flip-out clones (marked with GFP, circled with dotted lines) of *p60* in the wing imaginal discs, readout of TGF-ß signalling levels indicated by pMad is not altered in the clones vs. surrounding tissues.

(F-F’) Heatshock induced flip-out clones (marked with GFP, circled with dotted lines) of *tkv*^{CA} in the wing imaginal discs, readout of insulin signalling levels indicated by pAkt is not altered in the clones vs. surrounding tissues.

(G-G’) Heatshock induced flip out clones (marked with GFP, circled with dotted lines) of *mad*^{RNAi} in the wing imaginal discs, readout of insulin signalling levels indicated by pAkt is not altered in the clones vs. surrounding tissues.

Scale bar is 50 µm.

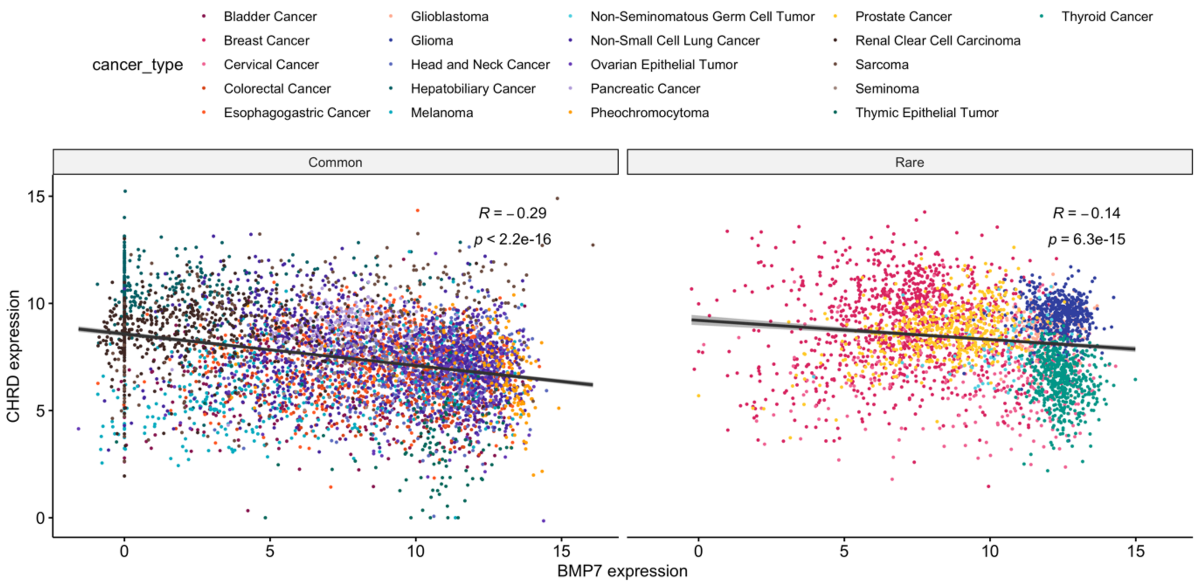

Appendix Figure S3: **Pan-cancer expression of CHRD compared to BMP7.** Data is RSEM batch normalized log2(value + 1) as per cBioPortal, points coloured by cancer type. Common cancers associated with cachexia include, bladder, colorectal, esophagogastric, Head and Neck, Hepatobiliary, Melanoma, Non-small cell lung cancer, Ovarian epithelial tumour, Pancreatic cancer, Pheochromocytoma, Renal Clear Cell carcinoma, Carcoma, Seminoma and Thymic epithelial tumour. Cancers rarely associated with cachexia include, Breast, Glioblastoma, Glioma, Seminomatous Germ cell Tumour, Prostate, Cervical and Thyroid. The date analyses 5441 samples from cancers where cachexia is common, 3127 from rare, and 841 of unknown. Spearman correlation R and p-values shown. Regression line is shown in black.
