# Supplementary figures and images for: Convergent insulin and TGF‐β signalling drives cancer cachexia by promoting aberrant fat body ECM accumulation in a Drosophila tumour model
Source: EMBO Rep. 2023 Nov 28;24(12):e57695. doi: 10.15252/embr.202357695 (PMC10702797; doi:10.15252/embr.202357695)

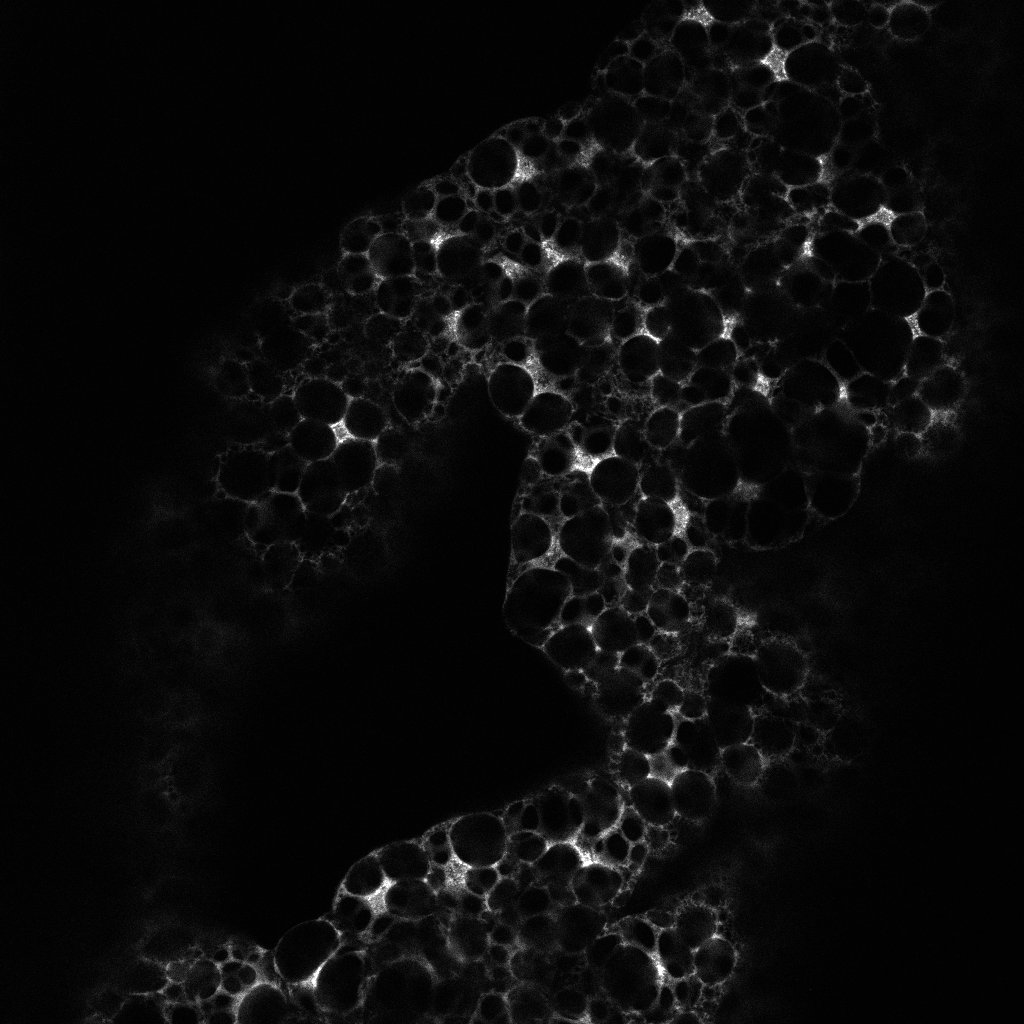

Supplement: Supplementary file 4 — Source Data for Figure 1 [file EMBR-24-e57695-s002.zip › Figure 1/E-F/C2-tumour dapi pmad-1.jpg]

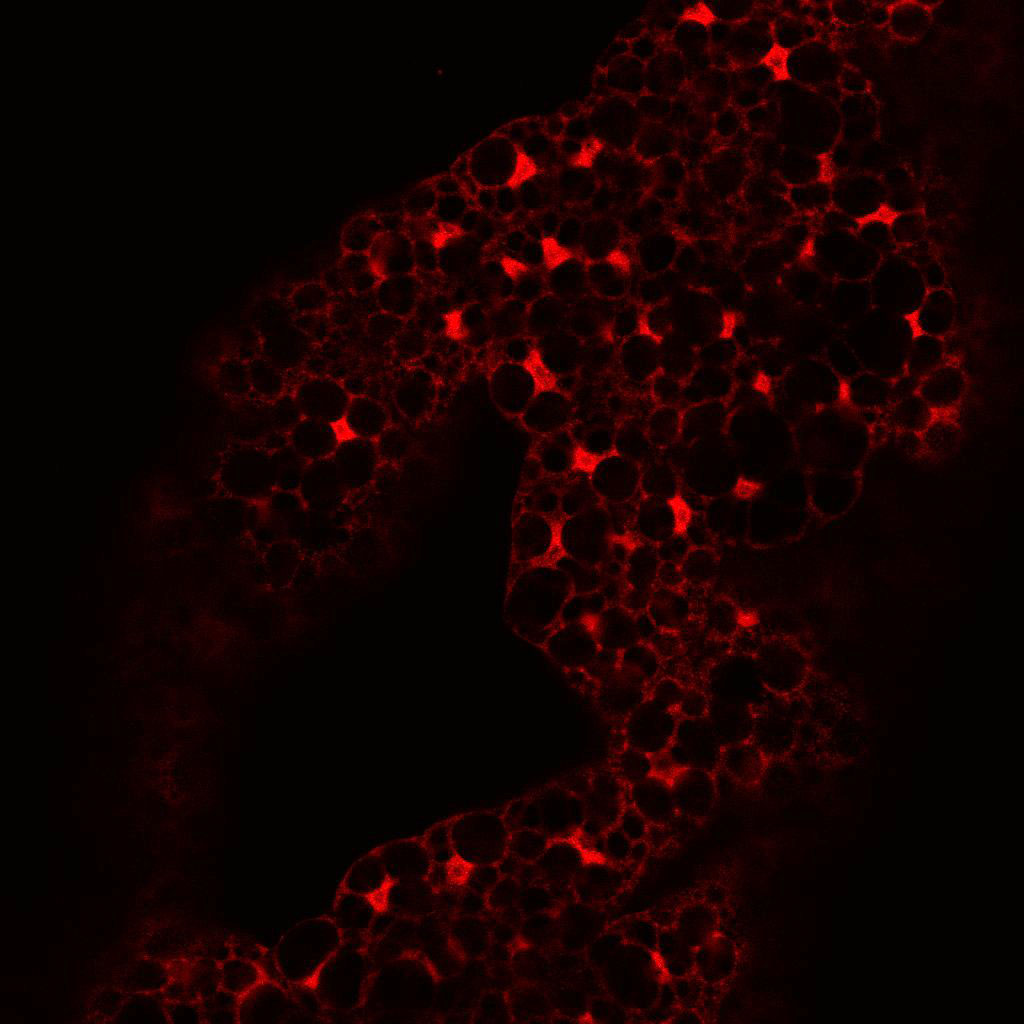

Supplement: Supplementary file 4 — Source Data for Figure 1 [file EMBR-24-e57695-s002.zip › Figure 1/E-F/C1-tumour dapi pmad-1.jpg]

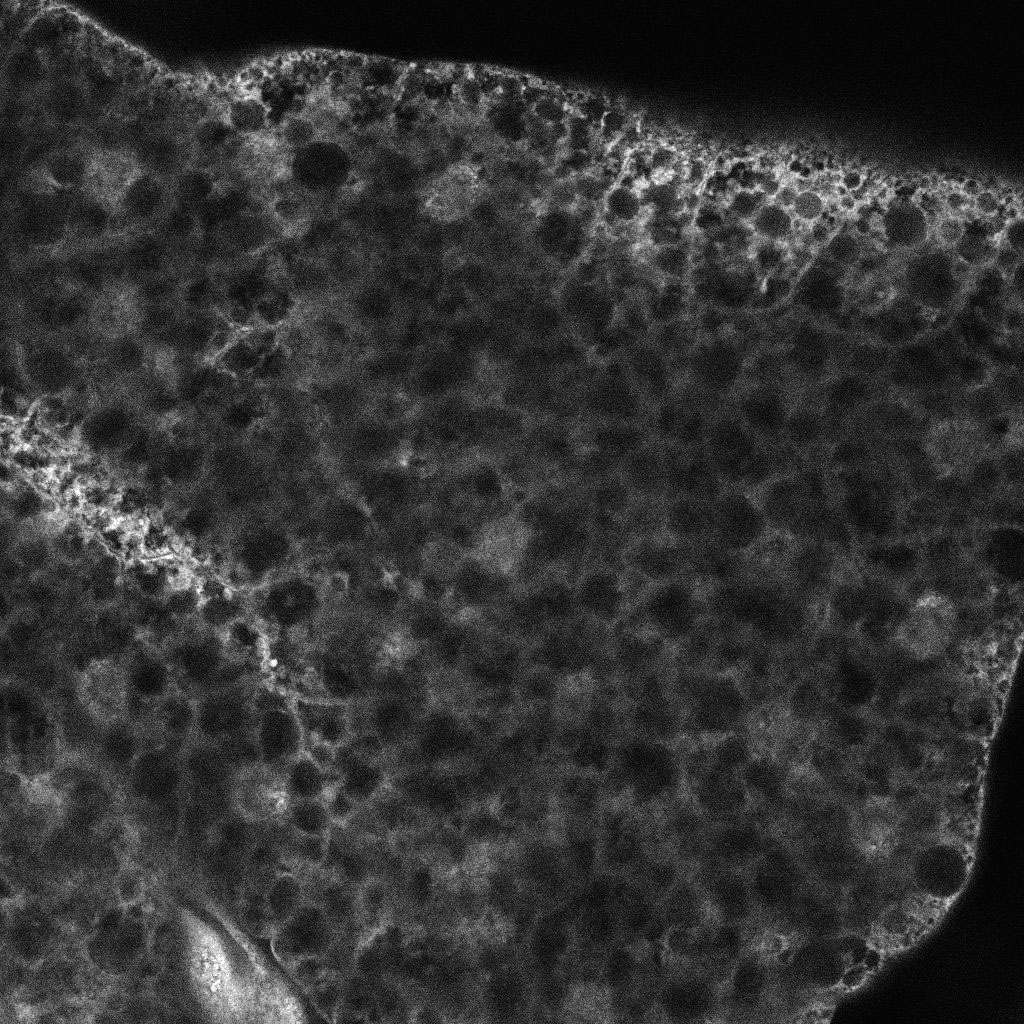

Supplement: Supplementary file 4 — Source Data for Figure 1 [file EMBR-24-e57695-s002.zip › Figure 1/E-F/C2-w118 dapi pmad-1.jpg]

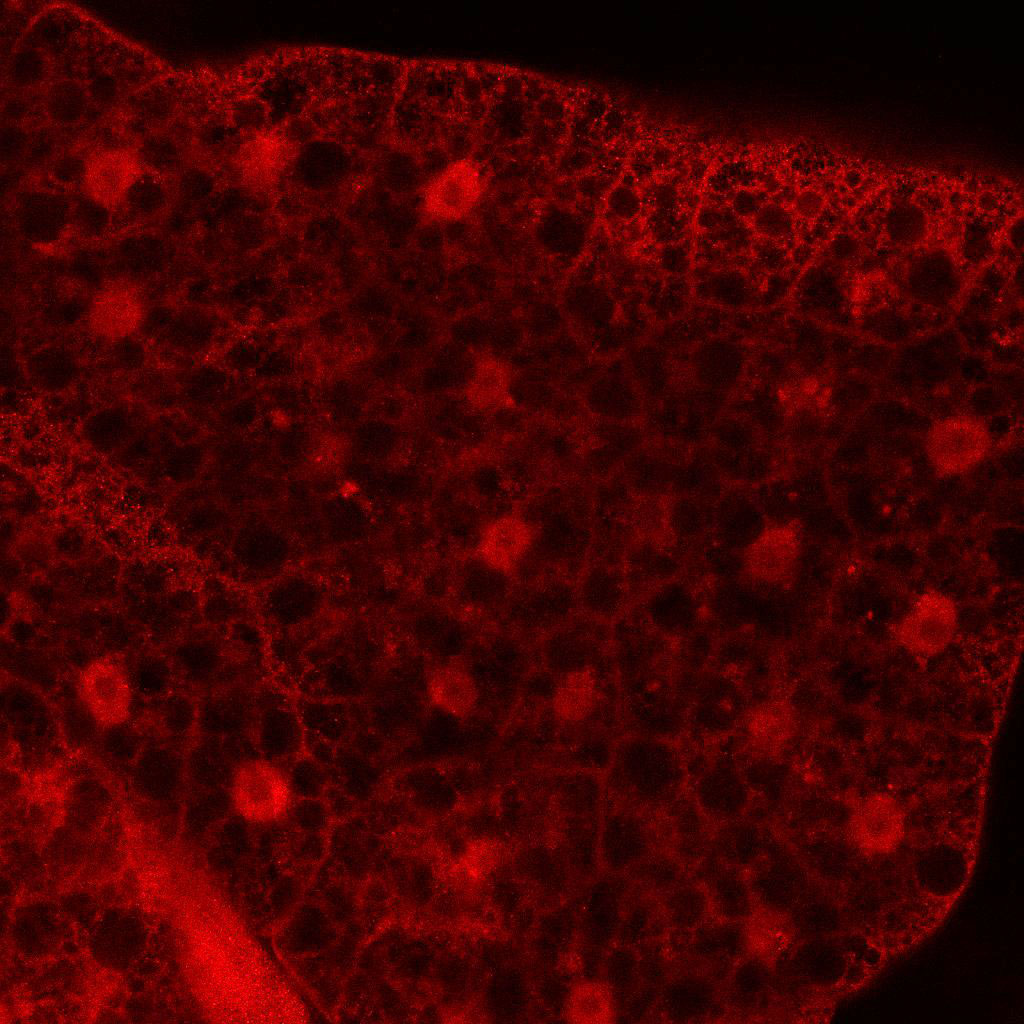

Supplement: Supplementary file 4 — Source Data for Figure 1 [file EMBR-24-e57695-s002.zip › Figure 1/E-F/C1-w118 dapi pmad-1.jpg]

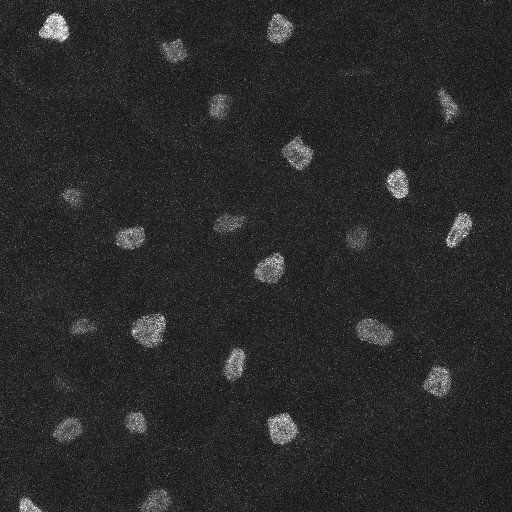

Supplement: Supplementary file 4 — Source Data for Figure 1 [file EMBR-24-e57695-s002.zip › Figure 1/H-I/MAX_Rasdlg pmad.lif - Series002.jpg]

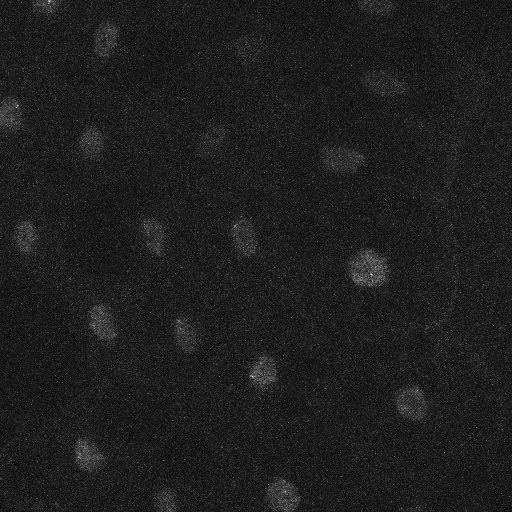

Supplement: Supplementary file 4 — Source Data for Figure 1 [file EMBR-24-e57695-s002.zip › Figure 1/H-I/MAX_Rasdlg impl2Ri pmad.lif - Series006.jpg]

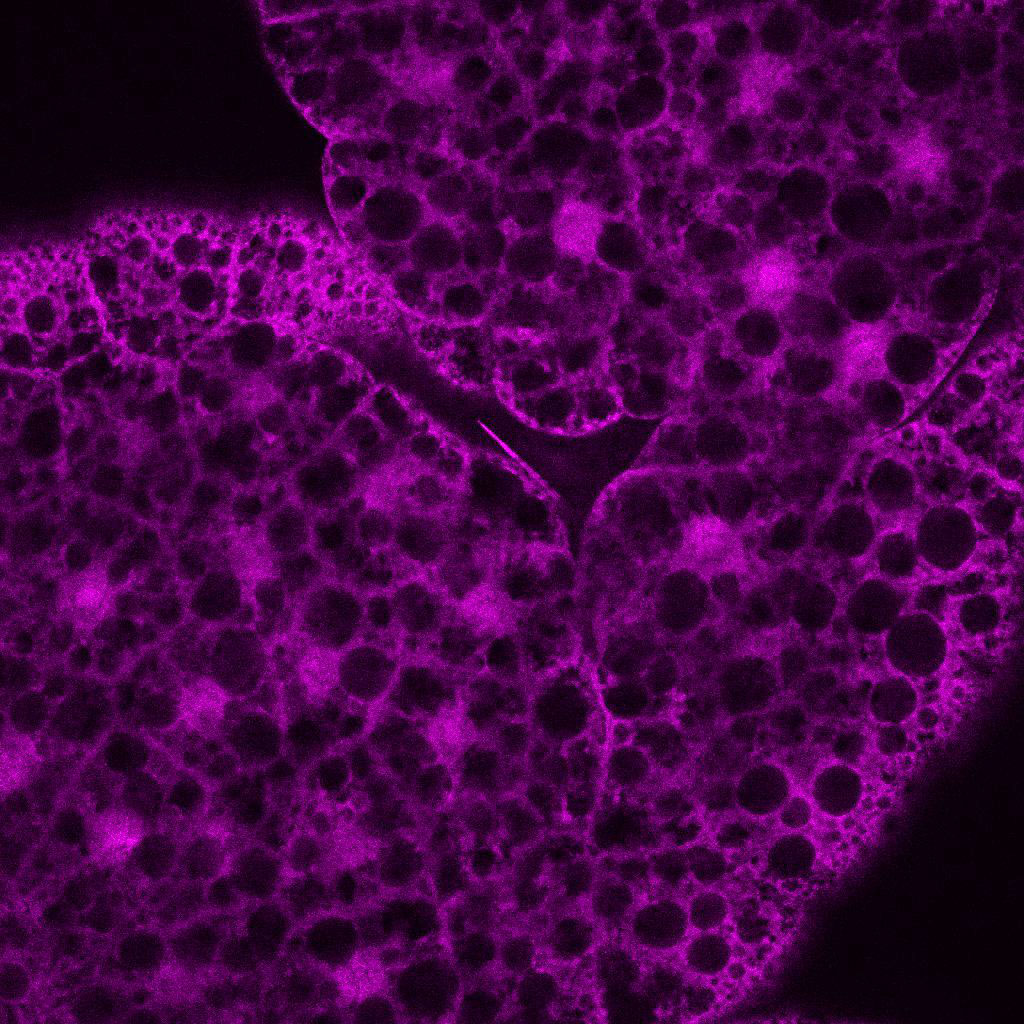

Supplement: Supplementary file 4 — Source Data for Figure 1 [file EMBR-24-e57695-s002.zip › Figure 1/B-C/C2-WT pakt DAPO_0001-1.jpg]

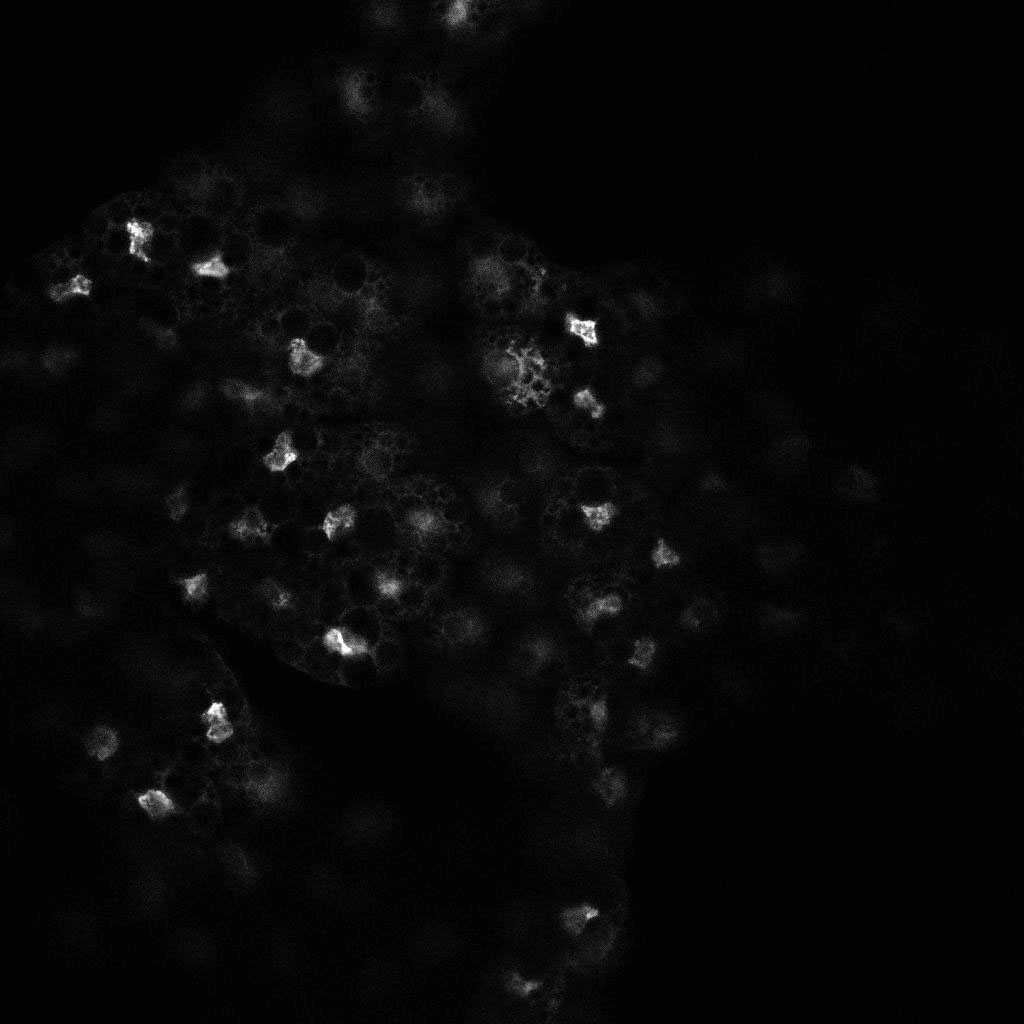

Supplement: Supplementary file 4 — Source Data for Figure 1 [file EMBR-24-e57695-s002.zip › Figure 1/B-C/C1-r4hack pakt DAPO_0003.jpg]

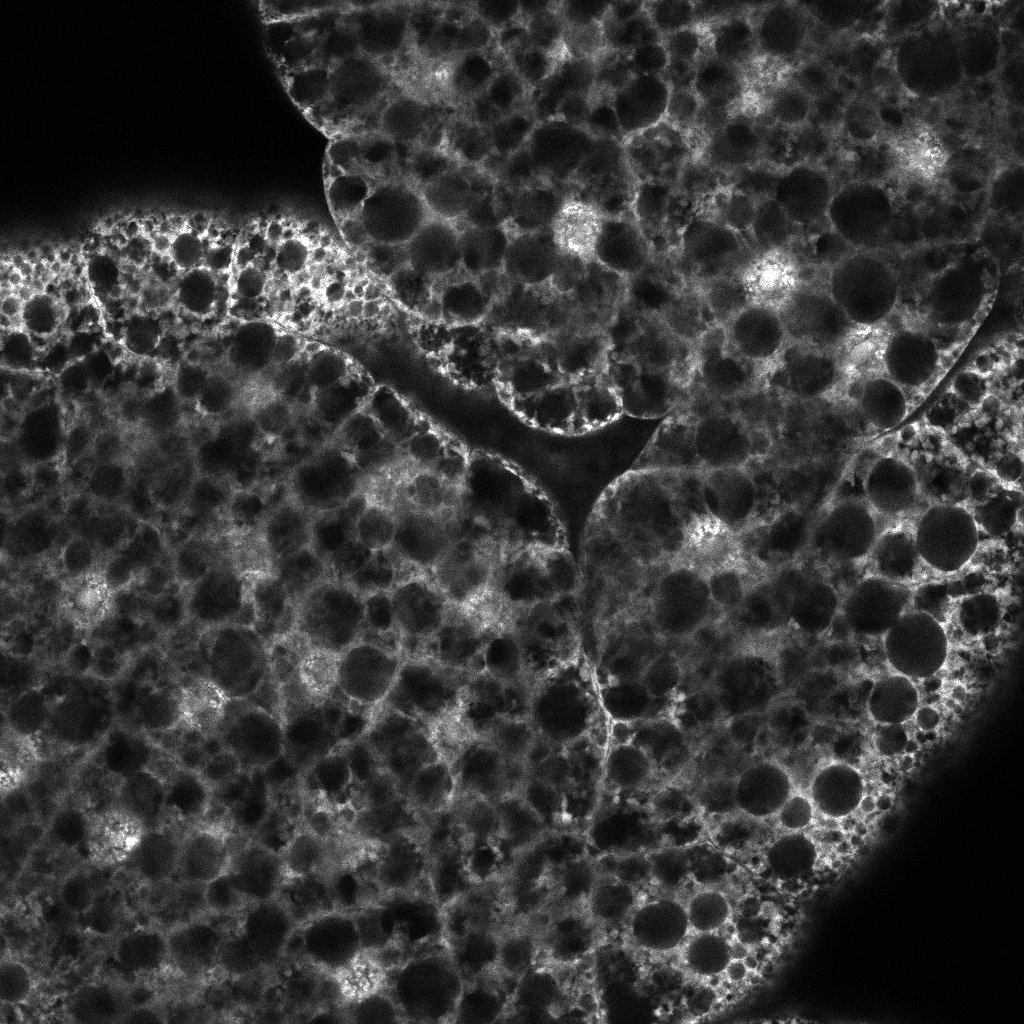

Supplement: Supplementary file 4 — Source Data for Figure 1 [file EMBR-24-e57695-s002.zip › Figure 1/B-C/C1-WT pakt DAPO_0001-1.jpg]

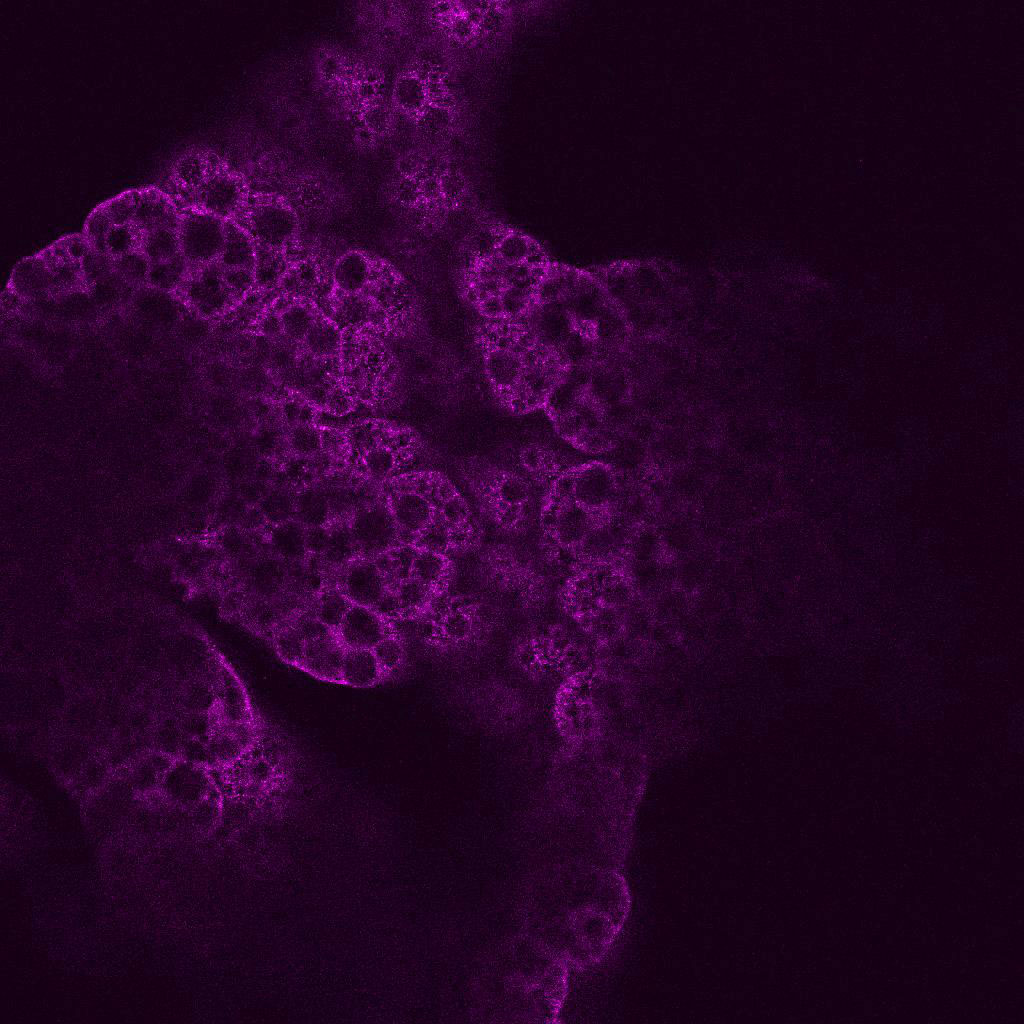

Supplement: Supplementary file 4 — Source Data for Figure 1 [file EMBR-24-e57695-s002.zip › Figure 1/B-C/C2-r4hack pakt DAPO_0003.jpg]

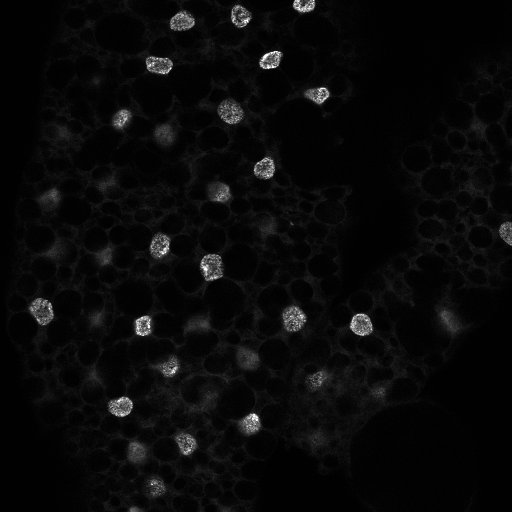

Supplement: Supplementary file 5 — Source Data for Figure 2 [file EMBR-24-e57695-s010.zip › Figure 2/Figure 2 J-K/C1-pAkt; 1-3, HACKr4mChRi; 4-5, HACKr4Sog, both FBs from same larva_A01_G001_0001-1.jpg]

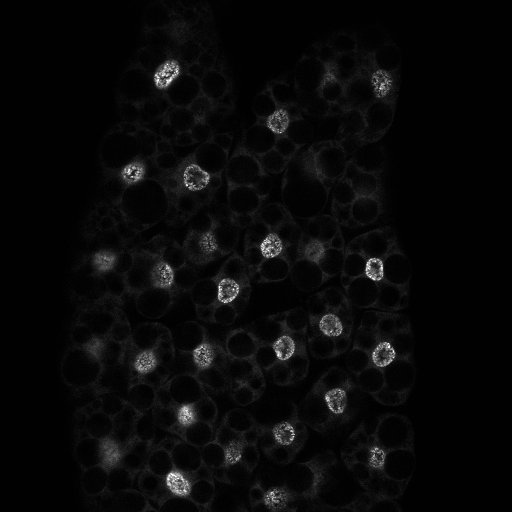

Supplement: Supplementary file 5 — Source Data for Figure 2 [file EMBR-24-e57695-s010.zip › Figure 2/Figure 2 J-K/C1-pAkt; 1-3, HACKr4mChRi; 4-5, HACKr4Sog, both FBs from same larva_A01_G003_0001-1.jpg]

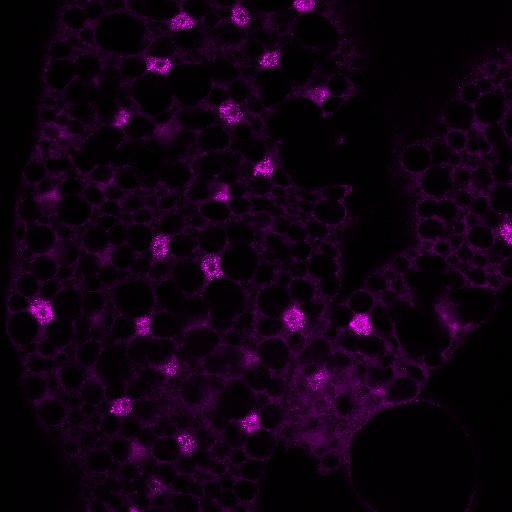

Supplement: Supplementary file 5 — Source Data for Figure 2 [file EMBR-24-e57695-s010.zip › Figure 2/Figure 2 J-K/C2-pAkt; 1-3, HACKr4mChRi; 4-5, HACKr4Sog, both FBs from same larva_A01_G001_0001-1.jpg]

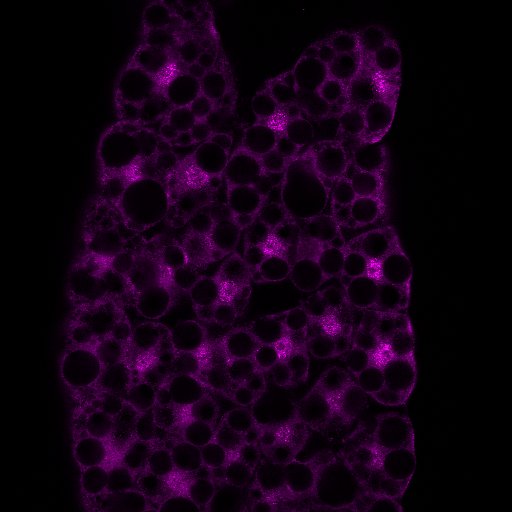

Supplement: Supplementary file 5 — Source Data for Figure 2 [file EMBR-24-e57695-s010.zip › Figure 2/Figure 2 J-K/C2-pAkt; 1-3, HACKr4mChRi; 4-5, HACKr4Sog, both FBs from same larva_A01_G003_0001-1.jpg]

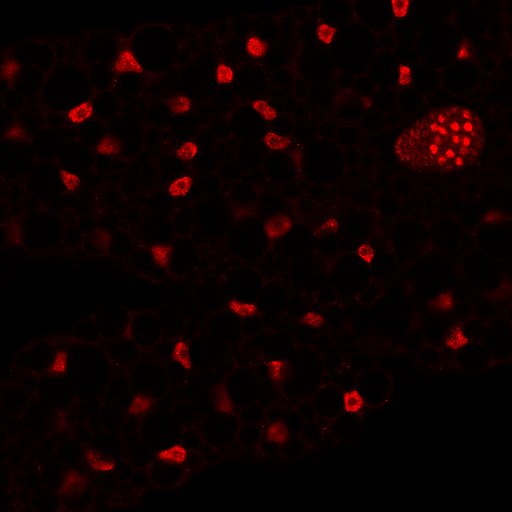

Supplement: Supplementary file 5 — Source Data for Figure 2 [file EMBR-24-e57695-s010.zip › Figure 2/Figure 2 D-E/C2-pMad; 1-3, HACKr4mChRi d6; 4-6, HACKr4Akt d6_A01_G001_0001-1.jpg]

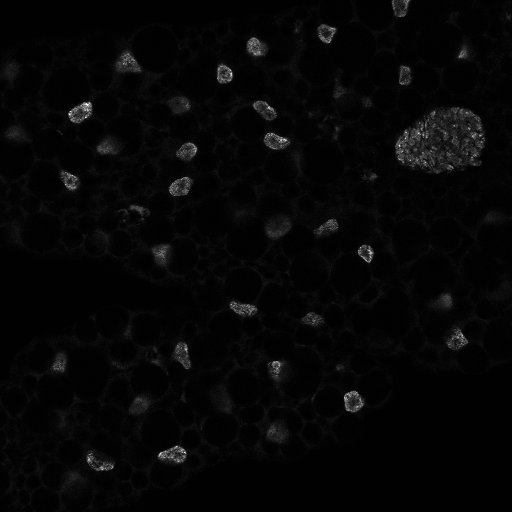

Supplement: Supplementary file 5 — Source Data for Figure 2 [file EMBR-24-e57695-s010.zip › Figure 2/Figure 2 D-E/C1-pMad; 1-3, HACKr4mChRi d6; 4-6, HACKr4Akt d6_A01_G001_0001-1.jpg]

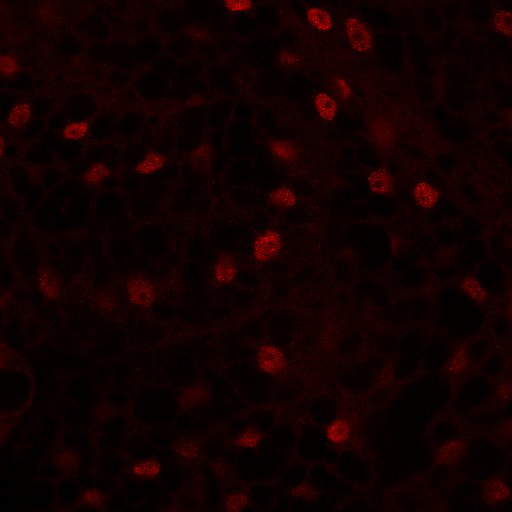

Supplement: Supplementary file 5 — Source Data for Figure 2 [file EMBR-24-e57695-s010.zip › Figure 2/Figure 2 D-E/C2-pMad; 1-3, HACKr4mChRi d6; 4-6, HACKr4Akt d6_A01_G005_0001-1.jpg]

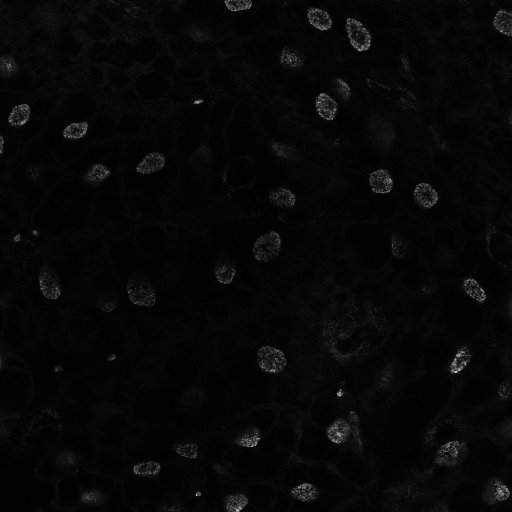

Supplement: Supplementary file 5 — Source Data for Figure 2 [file EMBR-24-e57695-s010.zip › Figure 2/Figure 2 D-E/C1-pMad; 1-3, HACKr4mChRi d6; 4-6, HACKr4Akt d6_A01_G005_0001-1.jpg]

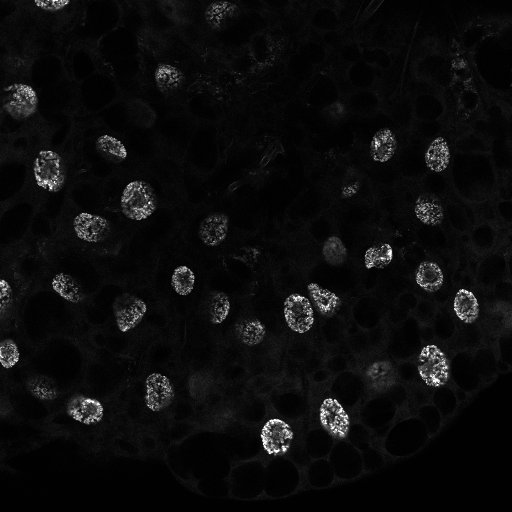

Supplement: Supplementary file 5 — Source Data for Figure 2 [file EMBR-24-e57695-s010.zip › Figure 2/Figure 2 A-B/C1-pAkt; 1-3, HACKr4mChRi d6; 4-6, HACKr4Akt d6_A01_G004_0001-1.jpg]

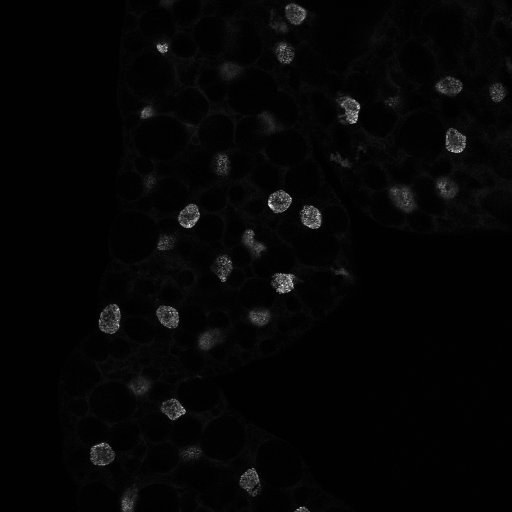

Supplement: Supplementary file 5 — Source Data for Figure 2 [file EMBR-24-e57695-s010.zip › Figure 2/Figure 2 A-B/C1-pAkt; 1-3, HACKr4mChRi d6; 4-6, HACKr4Akt d6_A01_G001_0001-1.jpg]

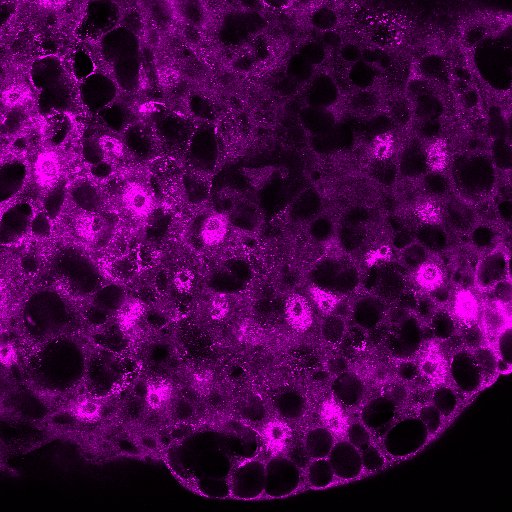

Supplement: Supplementary file 5 — Source Data for Figure 2 [file EMBR-24-e57695-s010.zip › Figure 2/Figure 2 A-B/C2-pAkt; 1-3, HACKr4mChRi d6; 4-6, HACKr4Akt d6_A01_G004_0001-1.jpg]

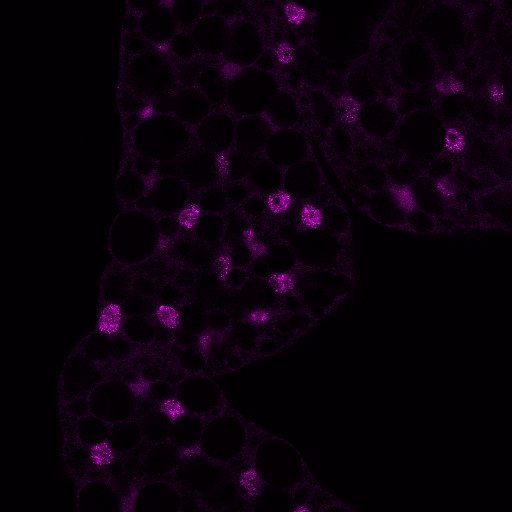

Supplement: Supplementary file 5 — Source Data for Figure 2 [file EMBR-24-e57695-s010.zip › Figure 2/Figure 2 A-B/C2-pAkt; 1-3, HACKr4mChRi d6; 4-6, HACKr4Akt d6_A01_G001_0001-1.jpg]

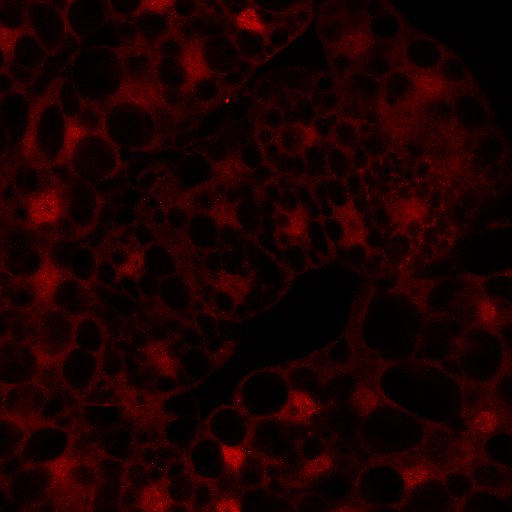

Supplement: Supplementary file 5 — Source Data for Figure 2 [file EMBR-24-e57695-s010.zip › Figure 2/Figure 2 G-H/C2-pMad; 1-2. HACKr4mChRi; 3-4. HACKr4Akt_A01_G003_0001-1.jpg]

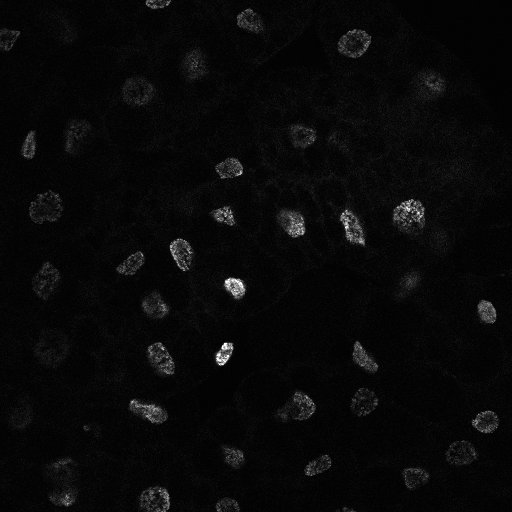

Supplement: Supplementary file 5 — Source Data for Figure 2 [file EMBR-24-e57695-s010.zip › Figure 2/Figure 2 G-H/C1-pMad; 1-2. HACKr4mChRi; 3-4. HACKr4Akt_A01_G003_0001-1.jpg]

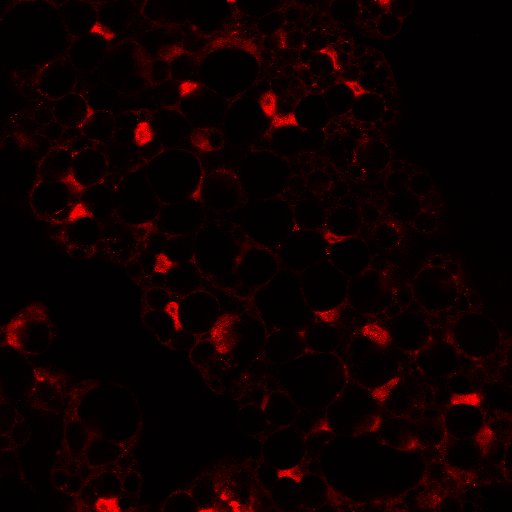

Supplement: Supplementary file 5 — Source Data for Figure 2 [file EMBR-24-e57695-s010.zip › Figure 2/Figure 2 G-H/C2-pMad; 1-2. HACKr4mChRi; 3-4. HACKr4Akt_A01_G001_0001-1.jpg]

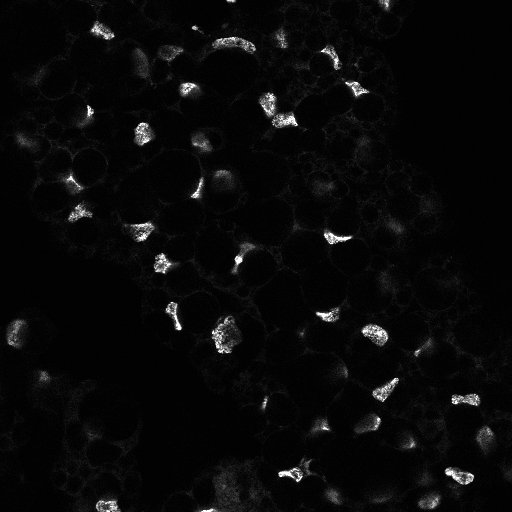

Supplement: Supplementary file 5 — Source Data for Figure 2 [file EMBR-24-e57695-s010.zip › Figure 2/Figure 2 G-H/C1-pMad; 1-2. HACKr4mChRi; 3-4. HACKr4Akt_A01_G001_0001-1.jpg]

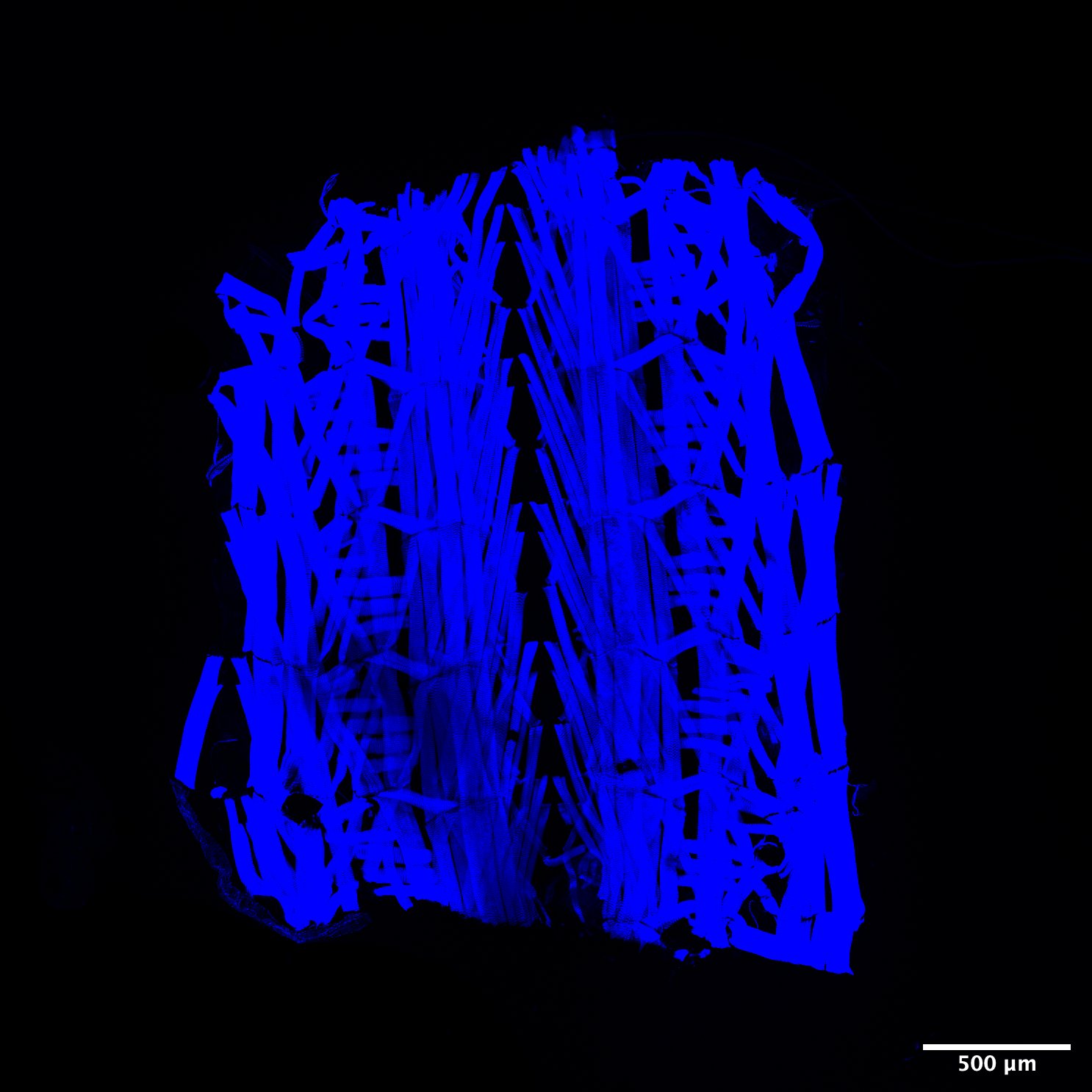

Supplement: Supplementary file 6 — Source Data for Figure 3 [file EMBR-24-e57695-s005.zip › Figure 3/C-D/w1118 scale bar.jpg]

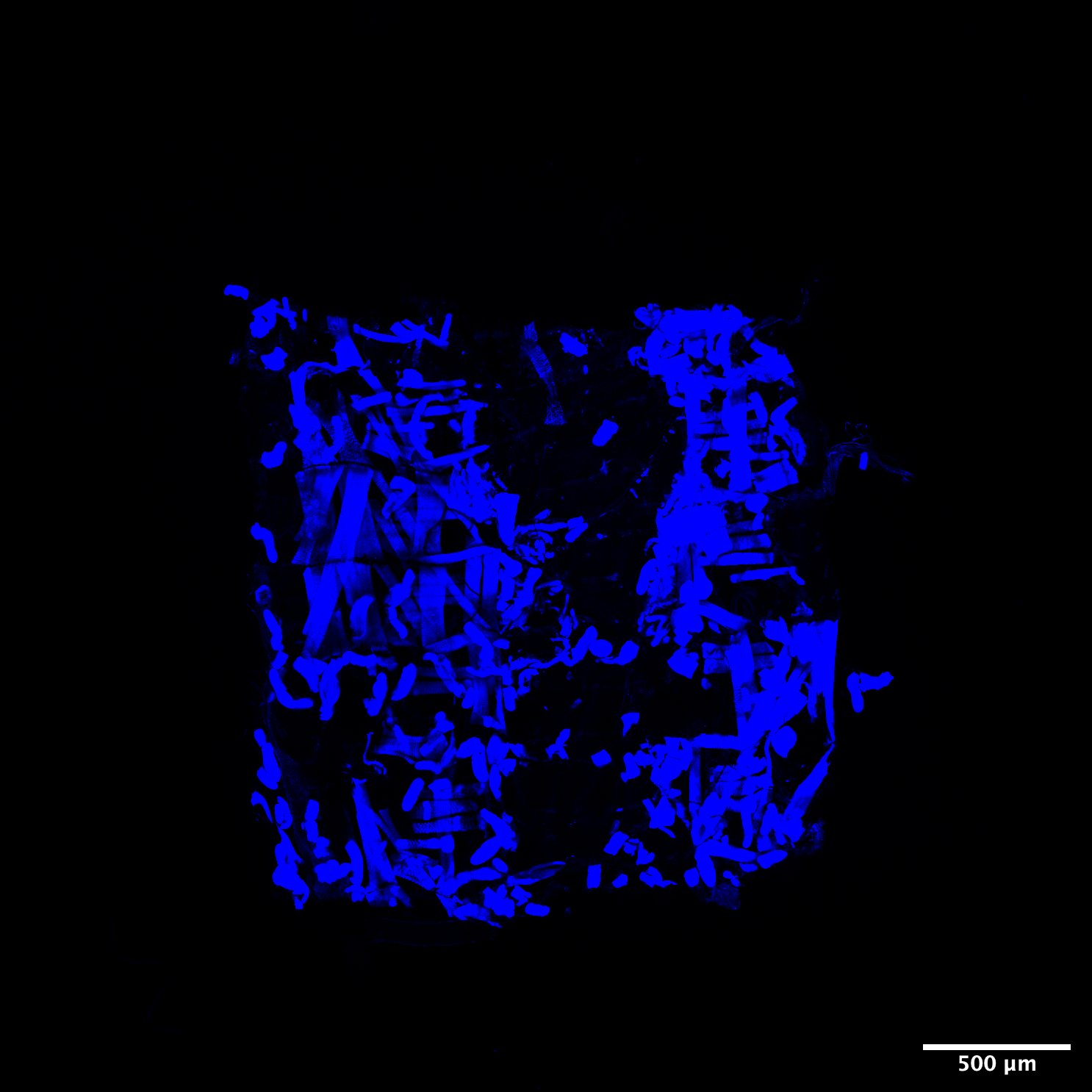

Supplement: Supplementary file 6 — Source Data for Figure 3 [file EMBR-24-e57695-s005.zip › Figure 3/C-D/HACKr4mChRi scale bar.jpg]

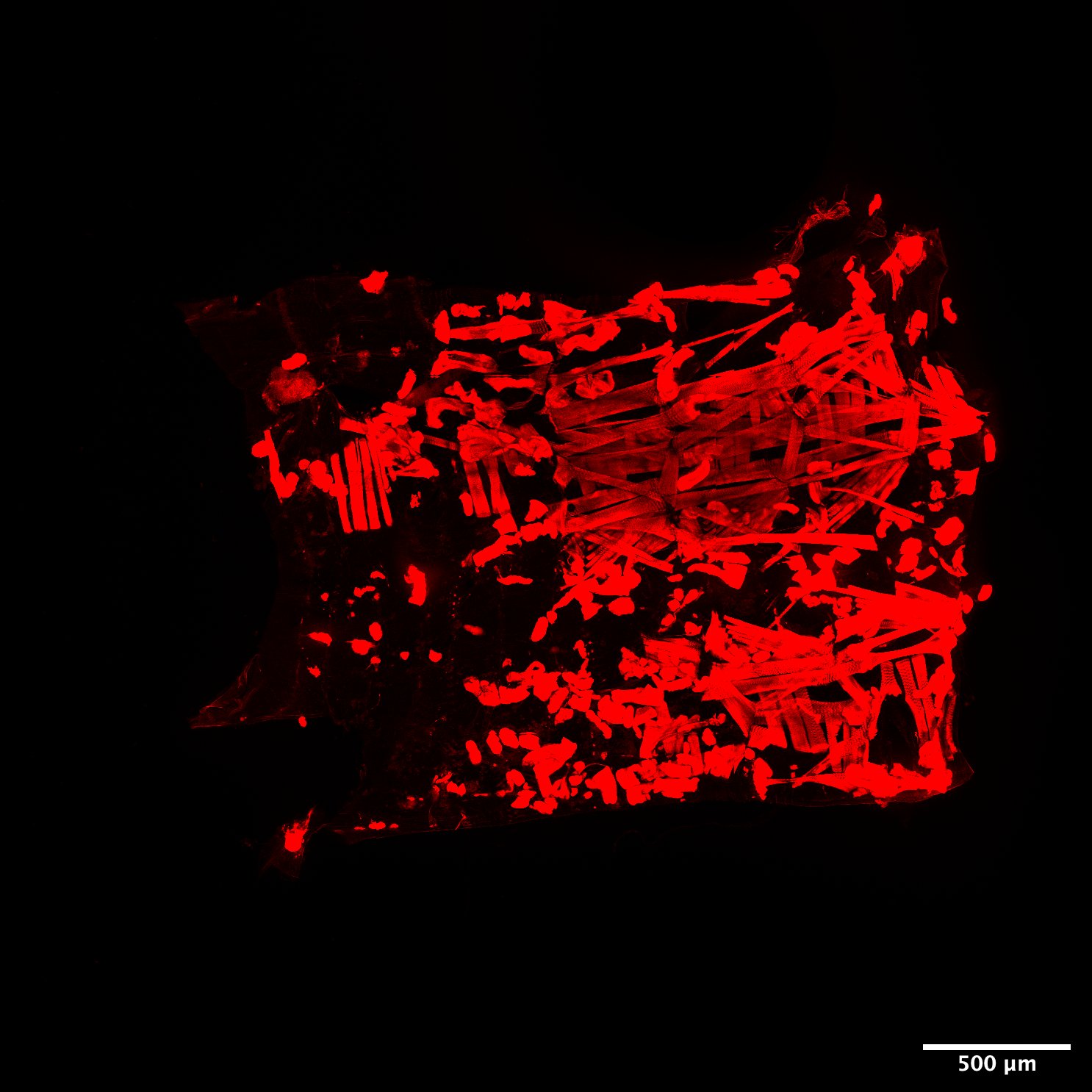

Supplement: Supplementary file 6 — Source Data for Figure 3 [file EMBR-24-e57695-s005.zip › Figure 3/I-J/HACKr4>laczRi scale bar.jpg]

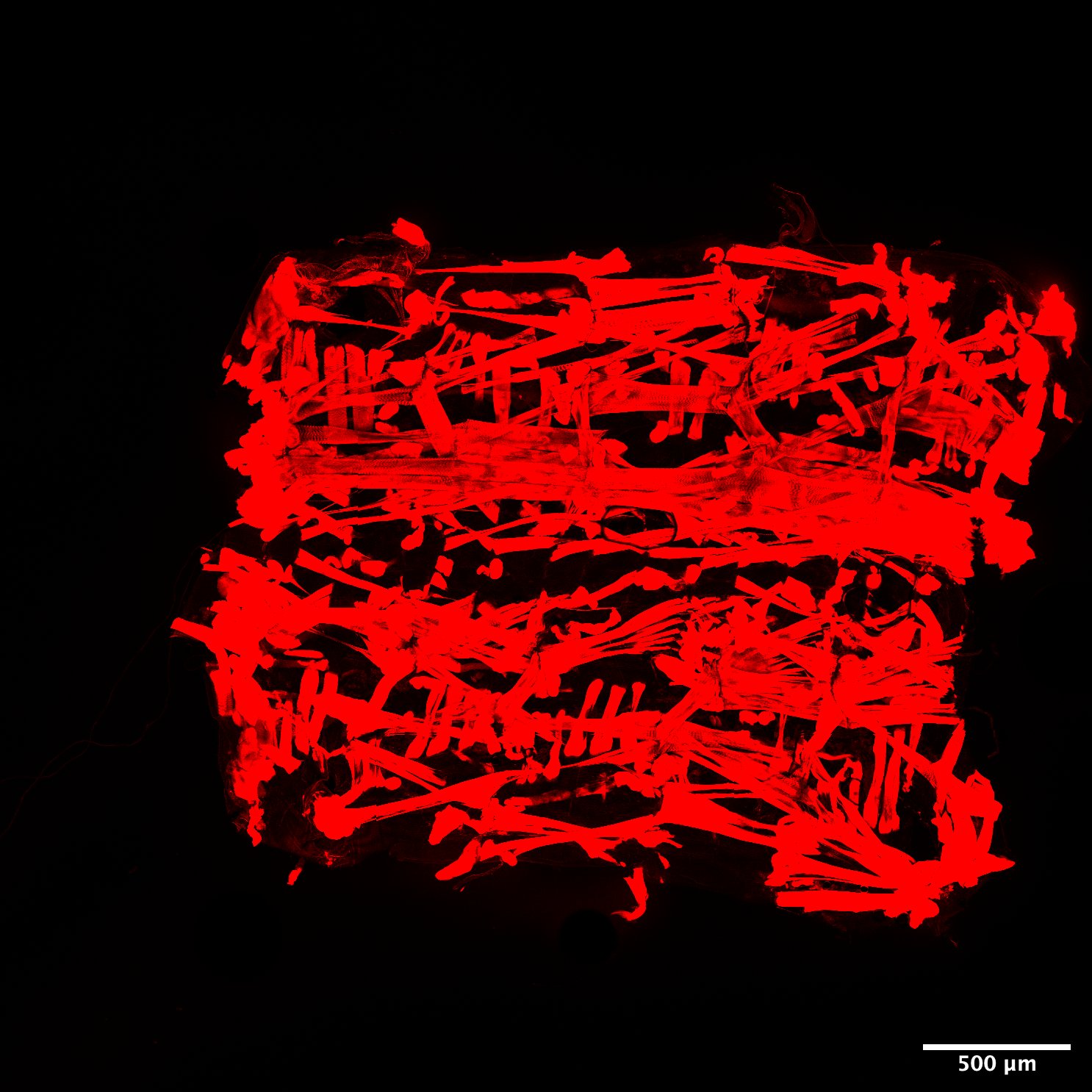

Supplement: Supplementary file 6 — Source Data for Figure 3 [file EMBR-24-e57695-s005.zip › Figure 3/I-J/HACKr4>InRCA scale bar.jpg]

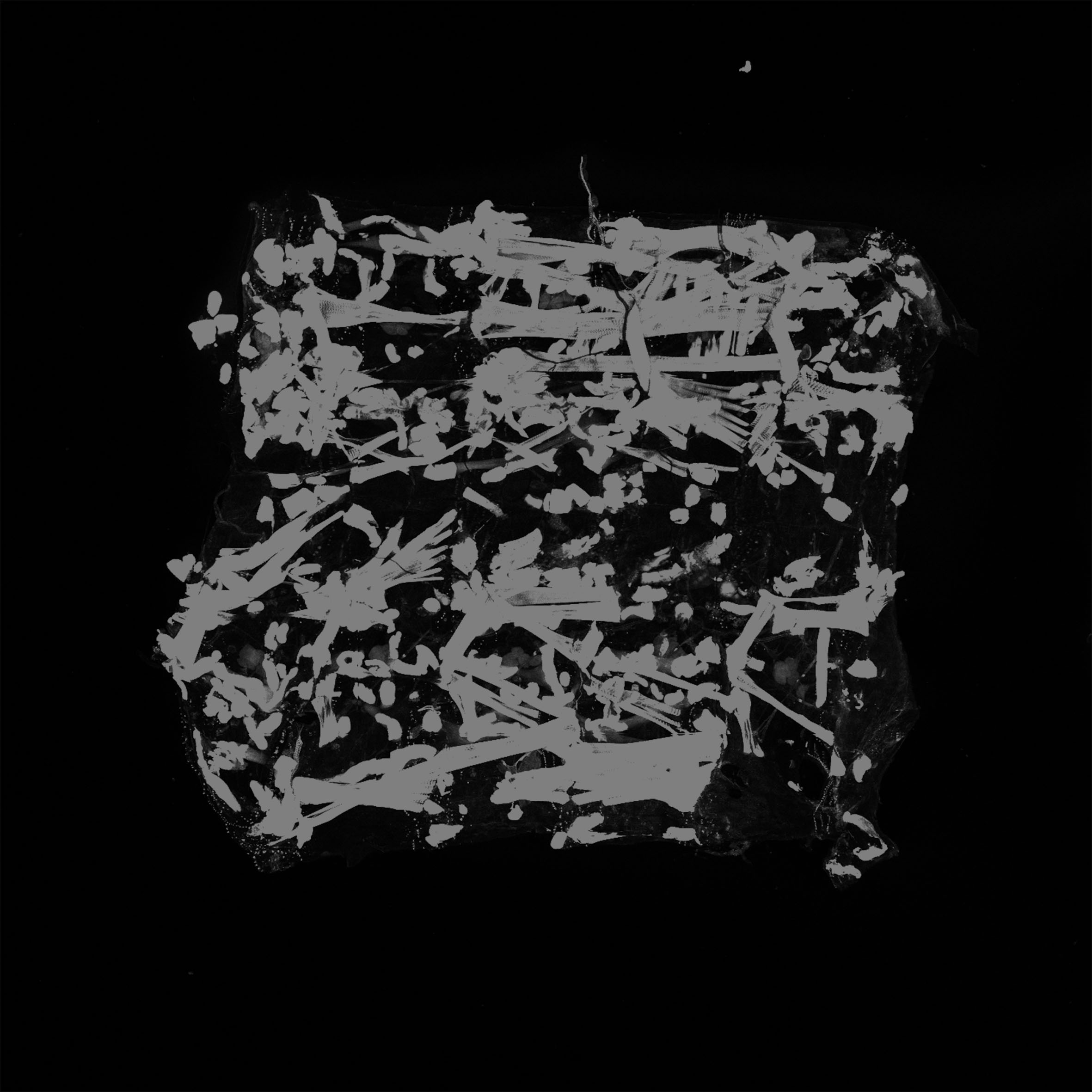

Supplement: Supplementary file 6 — Source Data for Figure 3 [file EMBR-24-e57695-s005.zip › Figure 3/F-G/HACKr4>mChRi d7 copy.jpg]

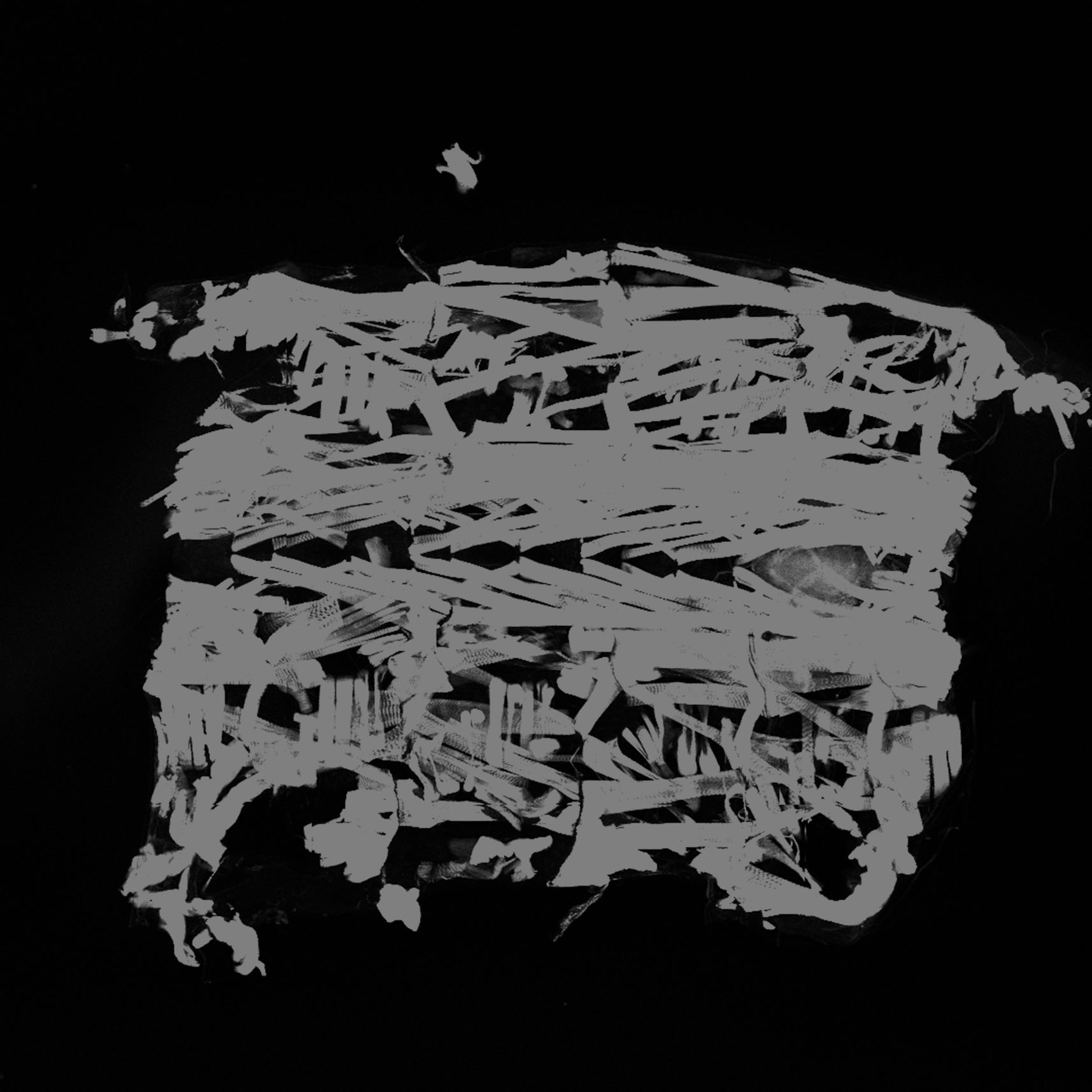

Supplement: Supplementary file 6 — Source Data for Figure 3 [file EMBR-24-e57695-s005.zip › Figure 3/F-G/HACKr4>Akt d7 copy.jpg]

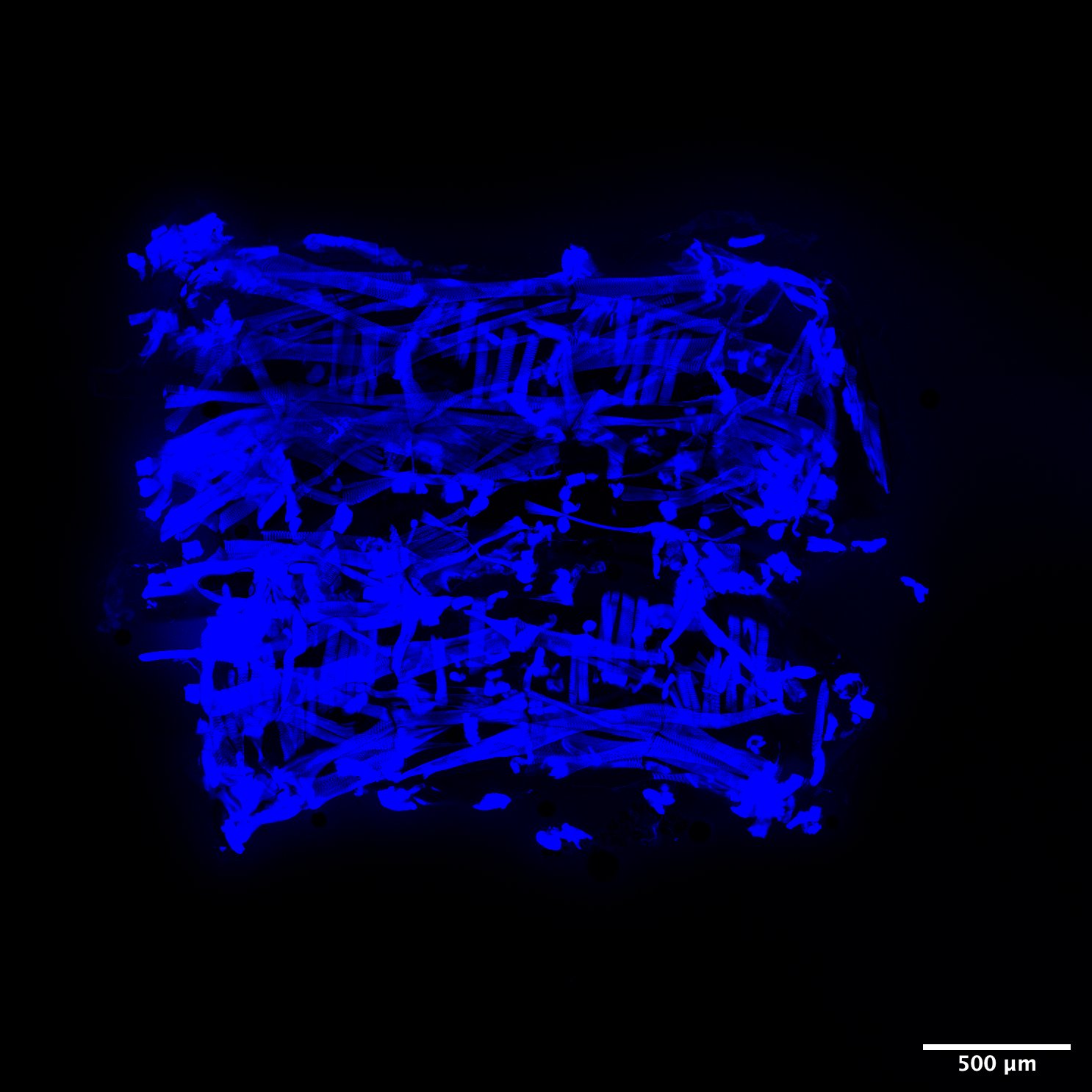

Supplement: Supplementary file 6 — Source Data for Figure 3 [file EMBR-24-e57695-s005.zip › Figure 3/L-N/madRi scale bar.jpg]

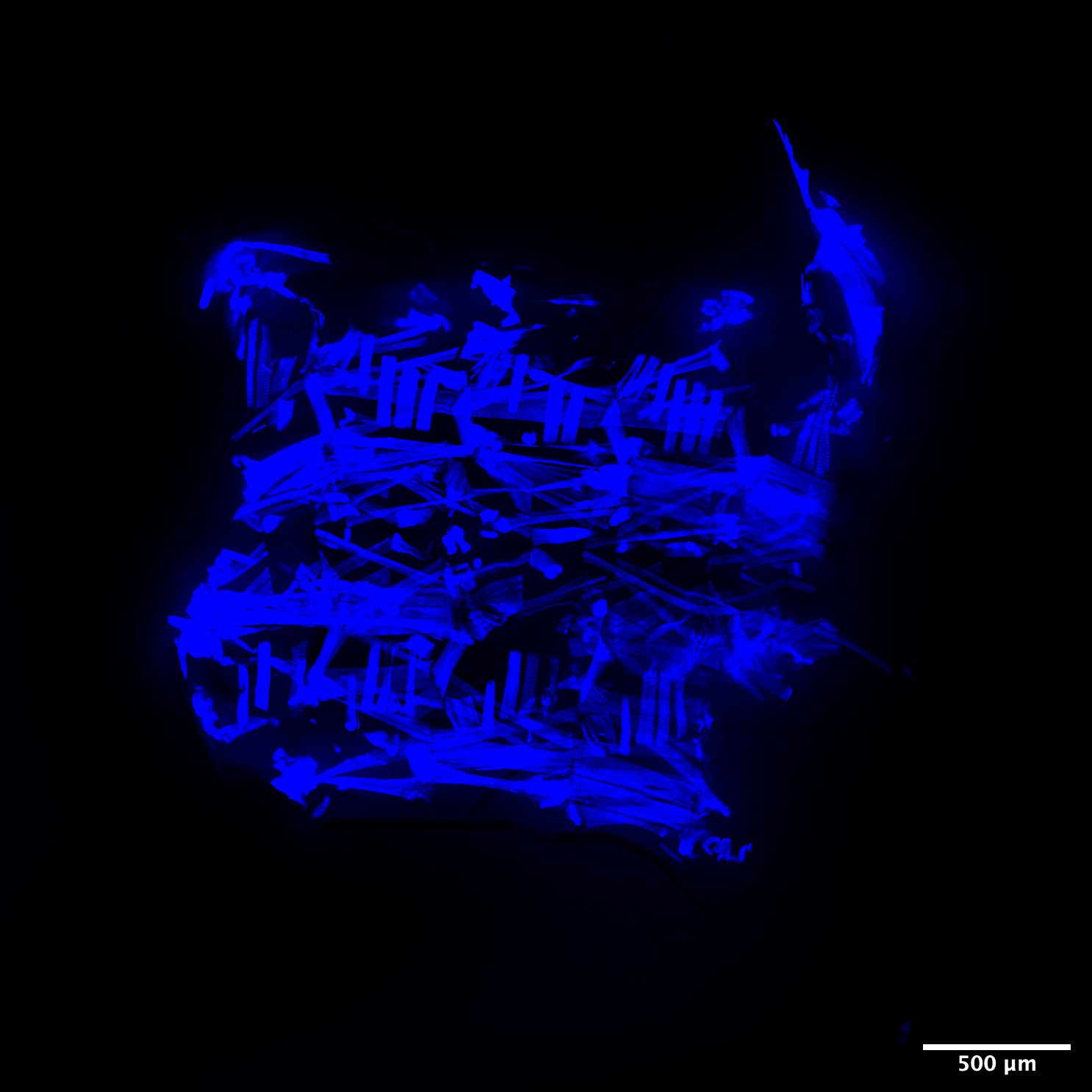

Supplement: Supplementary file 6 — Source Data for Figure 3 [file EMBR-24-e57695-s005.zip › Figure 3/L-N/InRCA scale bar.jpg]

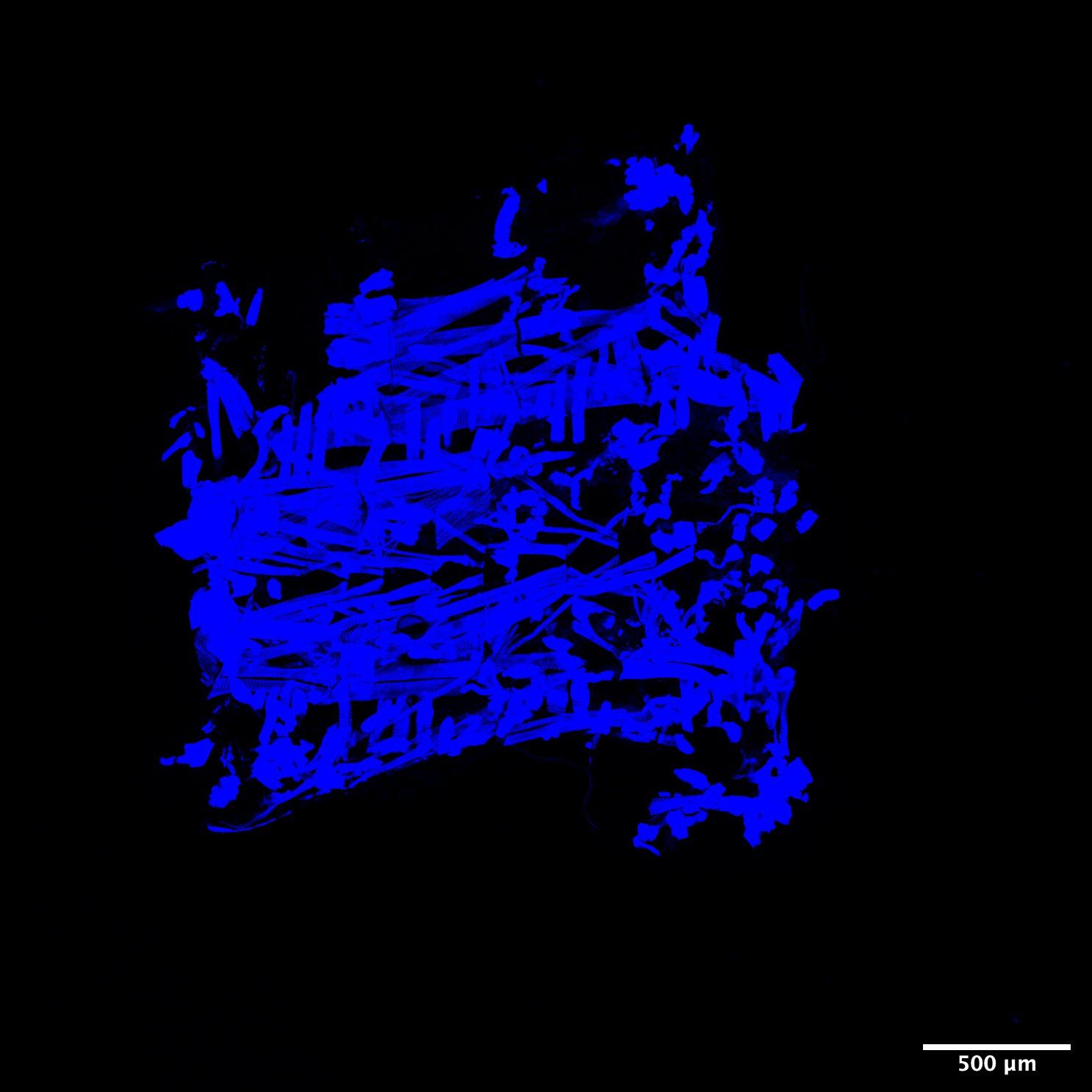

Supplement: Supplementary file 6 — Source Data for Figure 3 [file EMBR-24-e57695-s005.zip › Figure 3/L-N/InRCAmadRi scale bar.jpg]

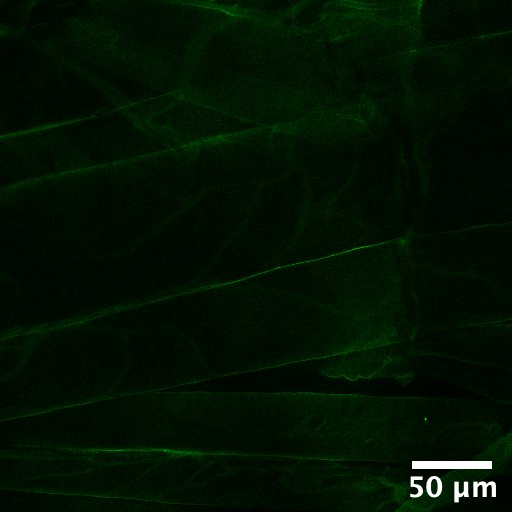

Supplement: Supplementary file 7 — Source Data for Figure 4 [file EMBR-24-e57695-s007.zip › Figure 4/C-D/HACKr4mChRi scale bar.jpg]

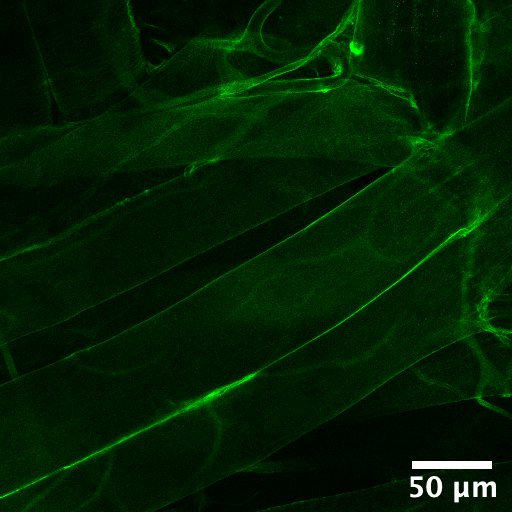

Supplement: Supplementary file 7 — Source Data for Figure 4 [file EMBR-24-e57695-s007.zip › Figure 4/C-D/HACKr4madRi scale bar.jpg]

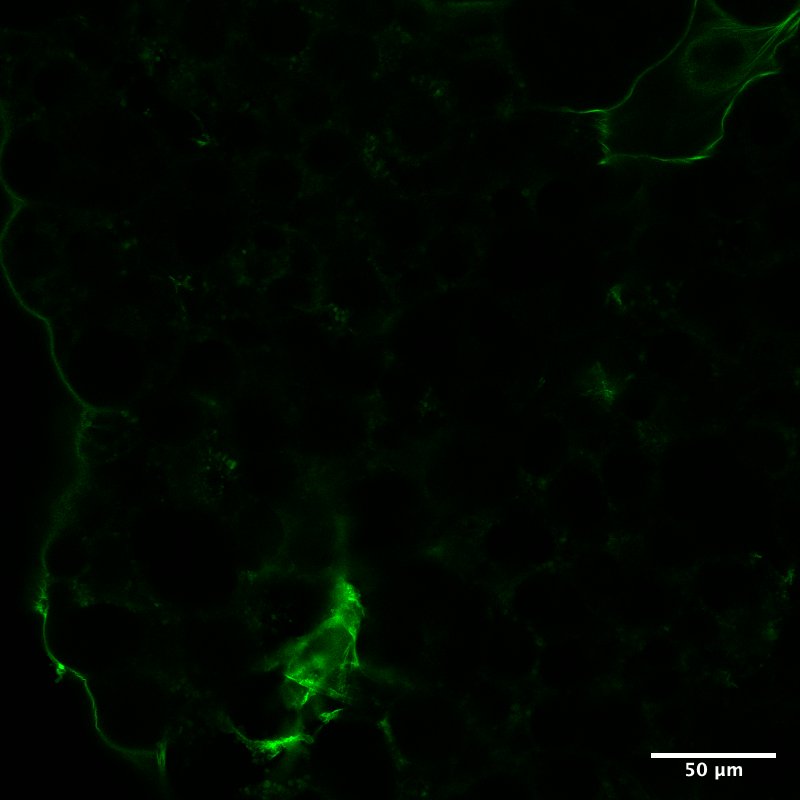

Supplement: Supplementary file 7 — Source Data for Figure 4 [file EMBR-24-e57695-s007.zip › Figure 4/H-I/HACKr4rab10Ri scale bar.jpg]

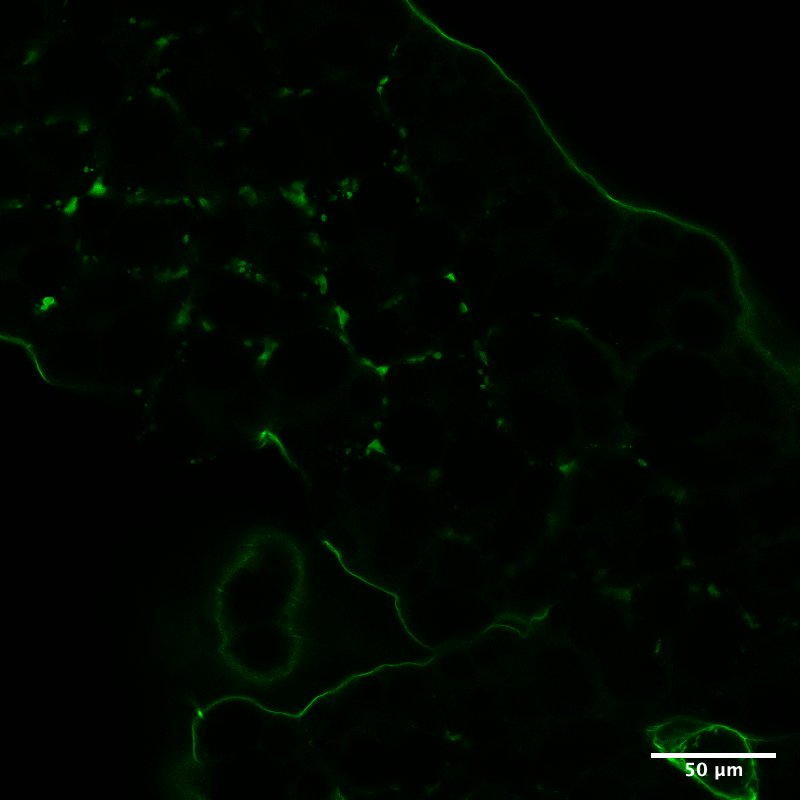

Supplement: Supplementary file 7 — Source Data for Figure 4 [file EMBR-24-e57695-s007.zip › Figure 4/H-I/HACKr4mChRi scale bar.jpg]

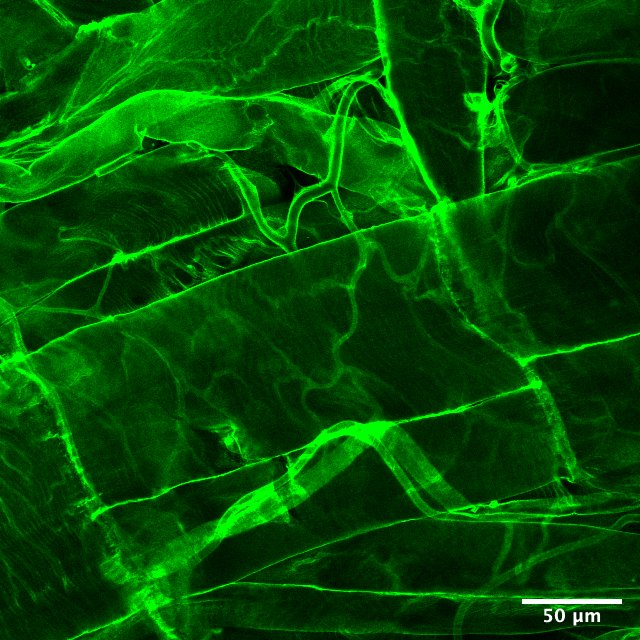

Supplement: Supplementary file 7 — Source Data for Figure 4 [file EMBR-24-e57695-s007.zip › Figure 4/J-K/HACKrab10Ri scale bar.jpg]

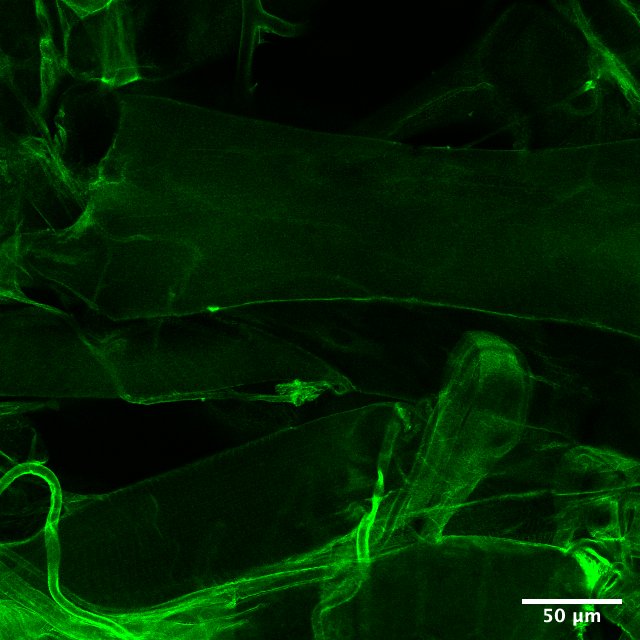

Supplement: Supplementary file 7 — Source Data for Figure 4 [file EMBR-24-e57695-s007.zip › Figure 4/J-K/HACKr4mChRi scale bar.jpg]

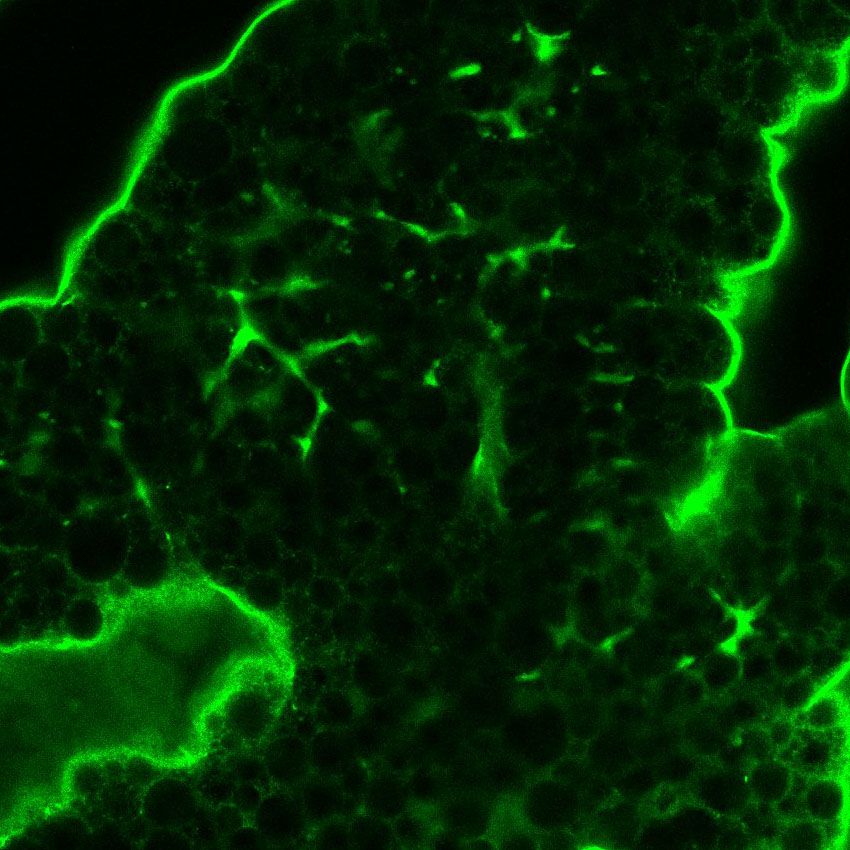

Supplement: Supplementary file 7 — Source Data for Figure 4 [file EMBR-24-e57695-s007.zip › Figure 4/P-R/mcherryri r4hack ndg phallodin_0001-1.jpg]

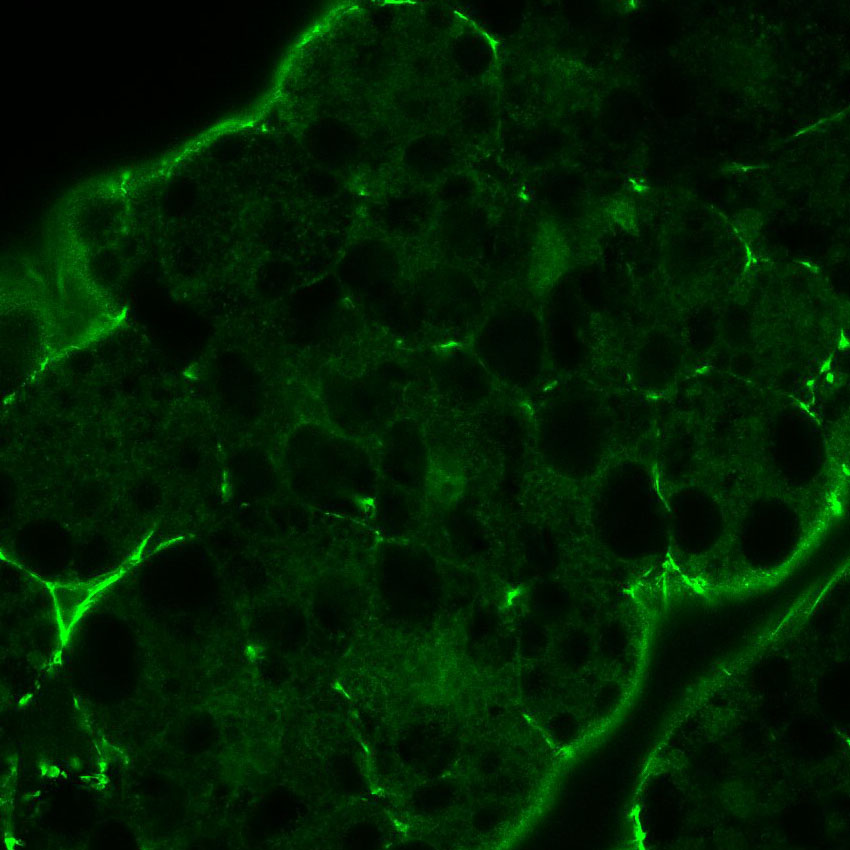

Supplement: Supplementary file 7 — Source Data for Figure 4 [file EMBR-24-e57695-s007.zip › Figure 4/P-R/UAs sparc r4 hack fb phallodin ndg_0001-1.jpg]

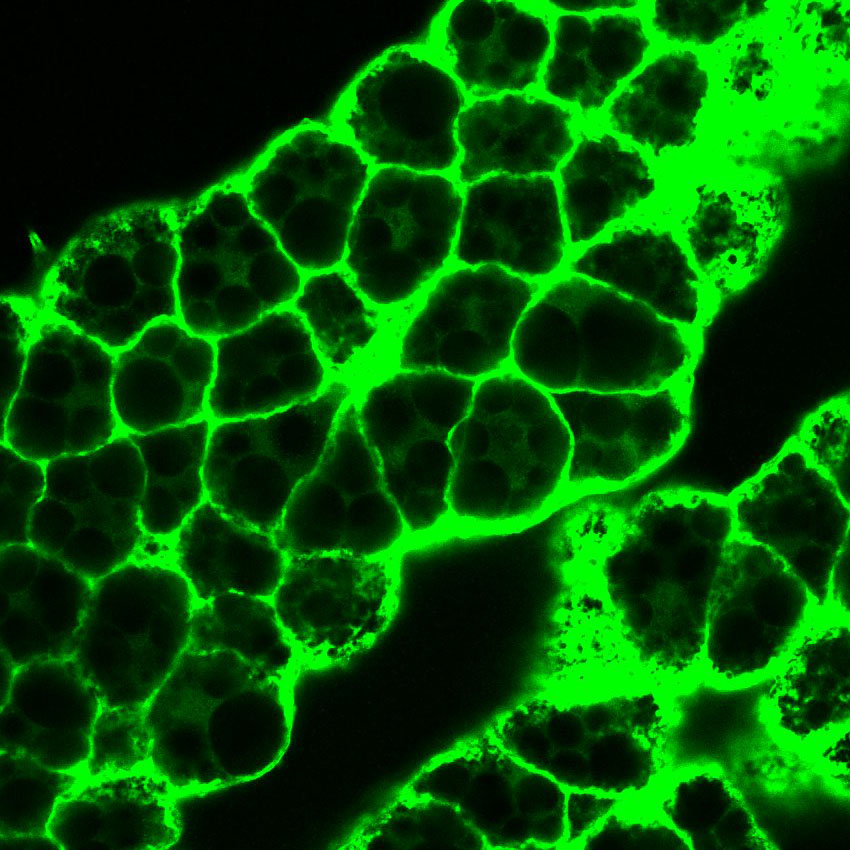

Supplement: Supplementary file 7 — Source Data for Figure 4 [file EMBR-24-e57695-s007.zip › Figure 4/P-R/sparc RNAi r4 hack fb phallodin ndg-1.jpg]

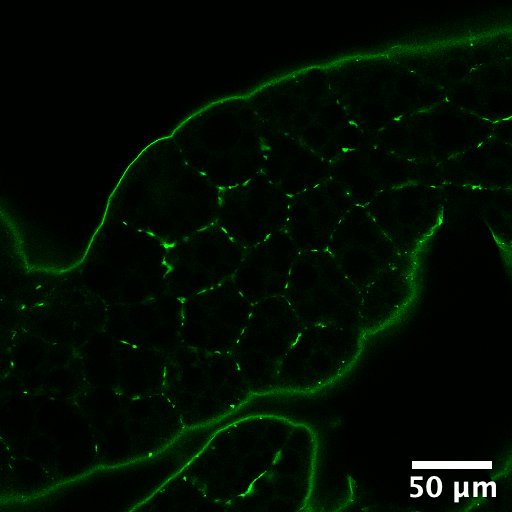

Supplement: Supplementary file 7 — Source Data for Figure 4 [file EMBR-24-e57695-s007.zip › Figure 4/A-B/HACKr4mChRi FB Ndg scale bar.jpg]

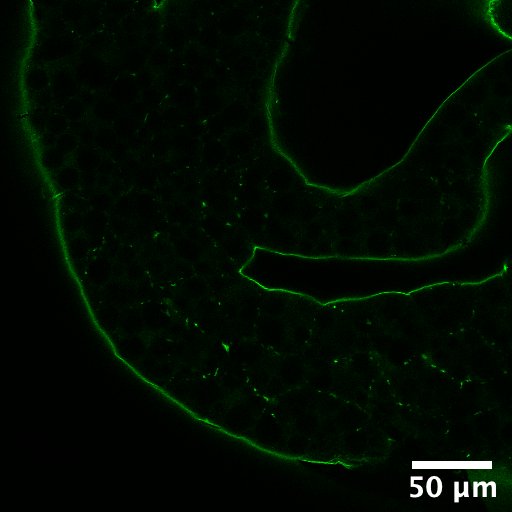

Supplement: Supplementary file 7 — Source Data for Figure 4 [file EMBR-24-e57695-s007.zip › Figure 4/A-B/HACKr4madRi FB Ndg scale bar.jpg]

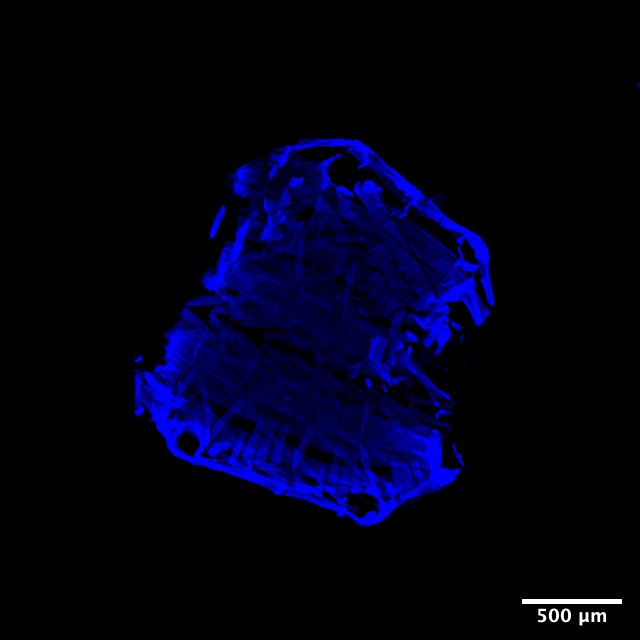

Supplement: Supplementary file 7 — Source Data for Figure 4 [file EMBR-24-e57695-s007.zip › Figure 4/L-M/HACKr4rab10Ri scale bar.jpg]

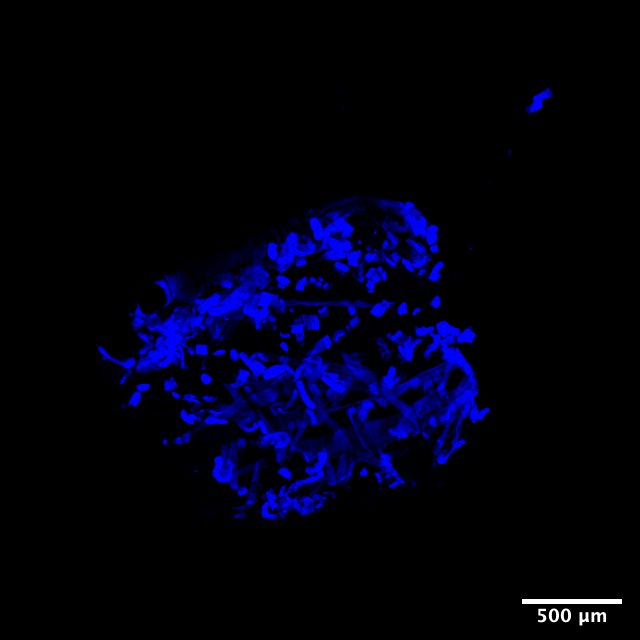

Supplement: Supplementary file 7 — Source Data for Figure 4 [file EMBR-24-e57695-s007.zip › Figure 4/L-M/HACKr4mChRi scale bar.jpg]

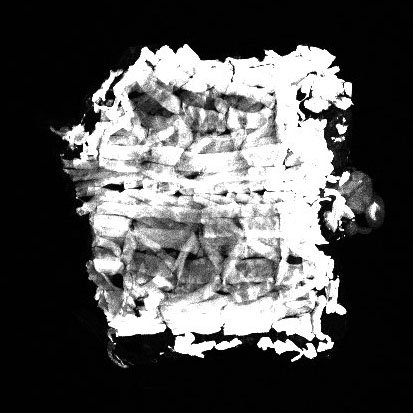

Supplement: Supplementary file 7 — Source Data for Figure 4 [file EMBR-24-e57695-s007.zip › Figure 4/T-V/MAX_r4 hack uas sparc fillet_A01_G001_0001.jpg]

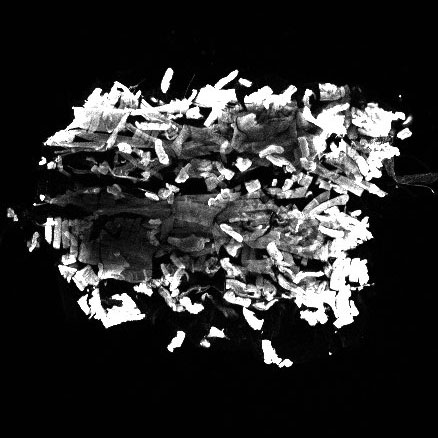

Supplement: Supplementary file 7 — Source Data for Figure 4 [file EMBR-24-e57695-s007.zip › Figure 4/T-V/MAX_r4 hack mcherryri fillet_A01_G002_0001.jpg]

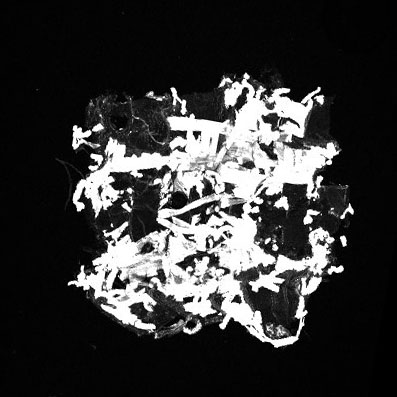

Supplement: Supplementary file 7 — Source Data for Figure 4 [file EMBR-24-e57695-s007.zip › Figure 4/T-V/MAX_r4 hack sparcRNAi fillet_A01_G002_0001.jpg]

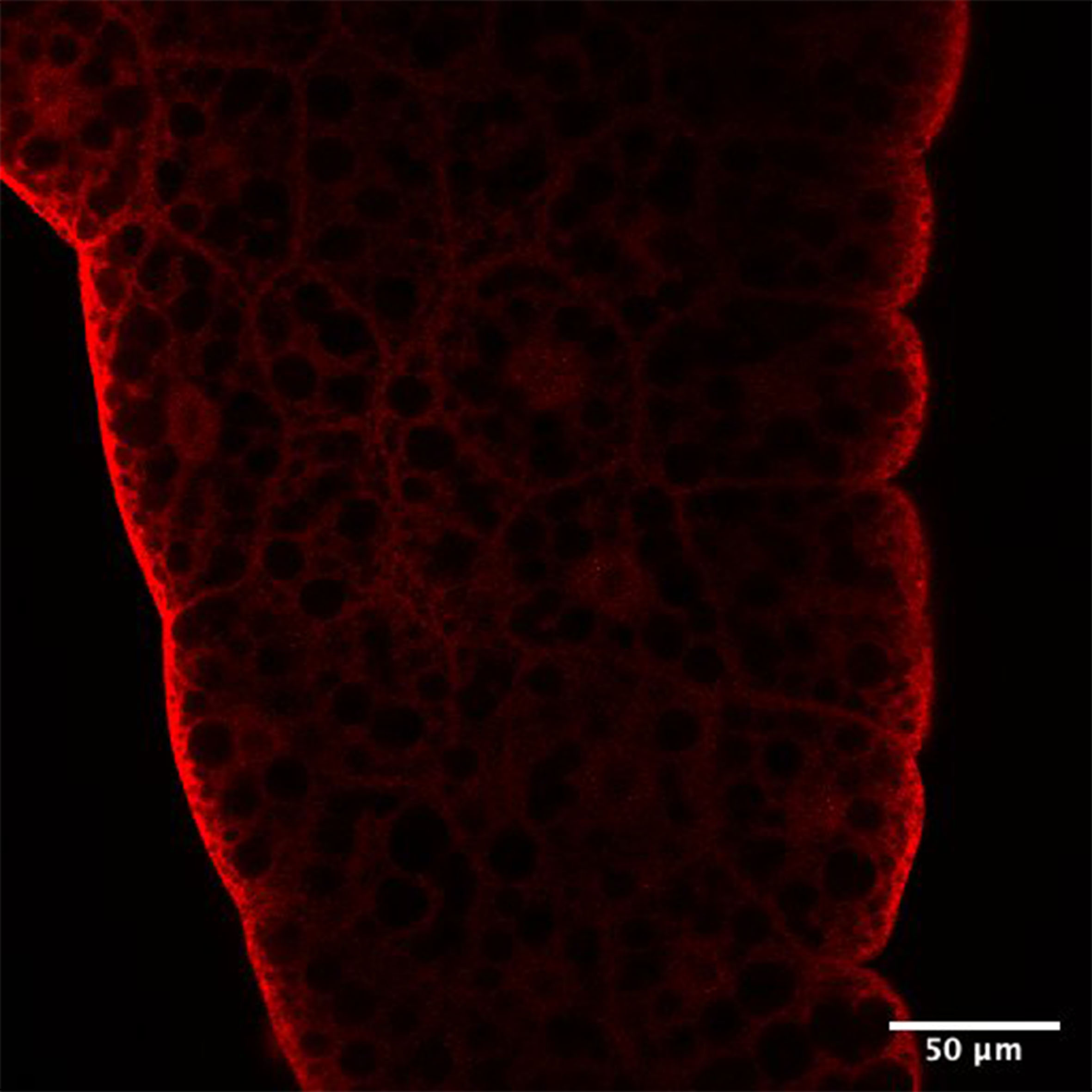

Supplement: Supplementary file 8 — Source Data for Figure 5 [file EMBR-24-e57695-s001.zip › Figure 5/D-E/CGInRCA pMad scale bar copy.jpg]

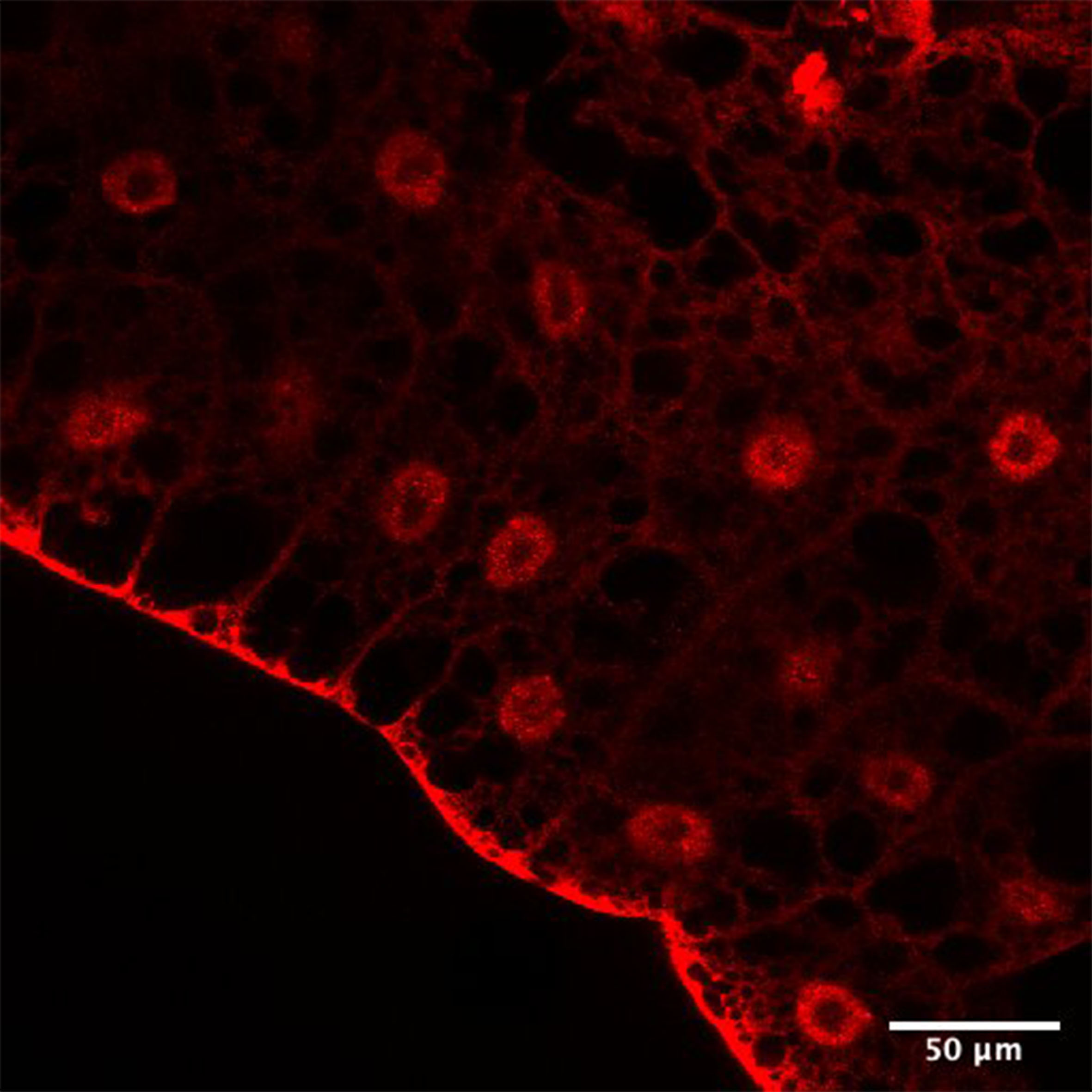

Supplement: Supplementary file 8 — Source Data for Figure 5 [file EMBR-24-e57695-s001.zip › Figure 5/D-E/CGmChRi for InRCA pMad scale bar copy.jpg]

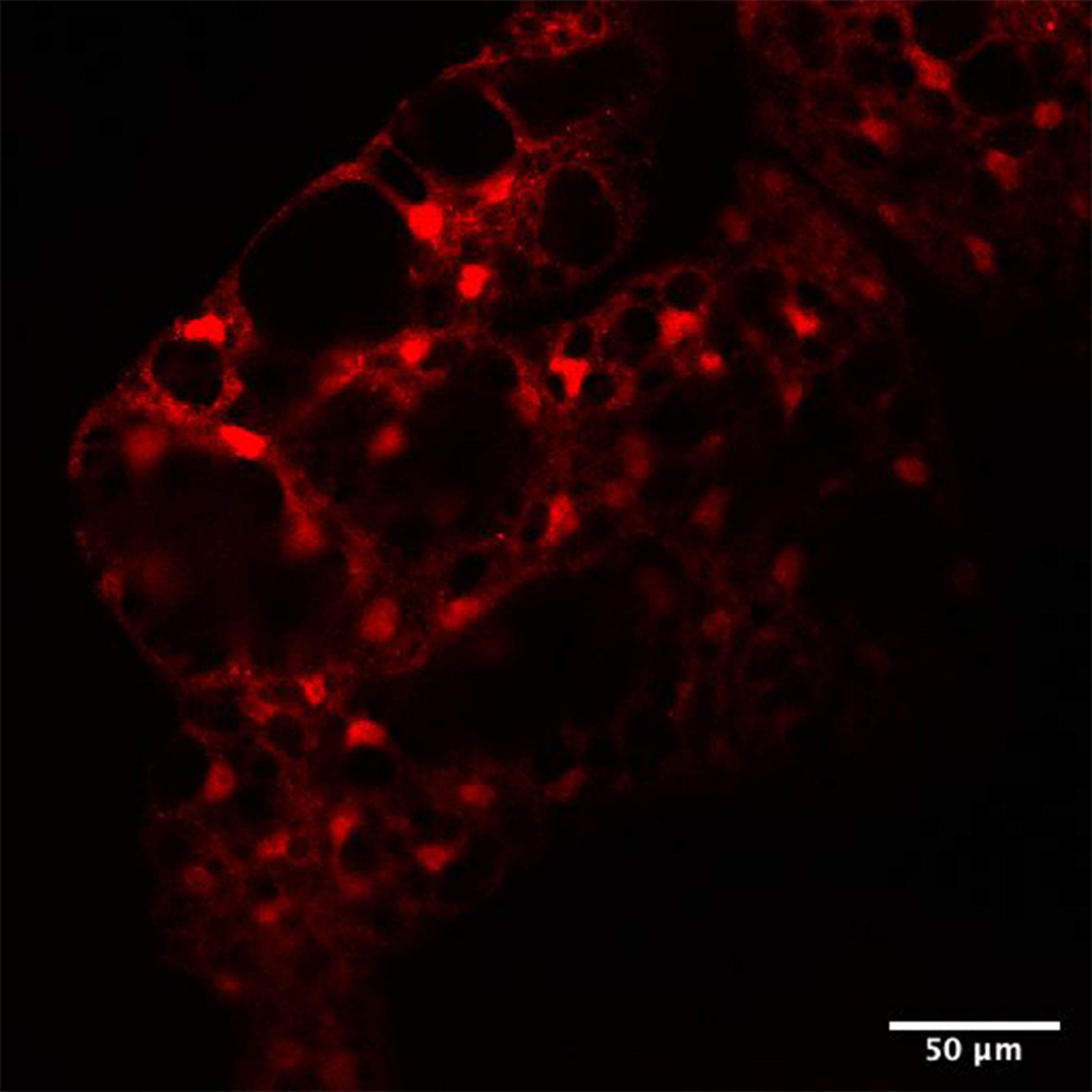

Supplement: Supplementary file 8 — Source Data for Figure 5 [file EMBR-24-e57695-s001.zip › Figure 5/G-H/CGp60DN pMad scale bar copy.jpg]

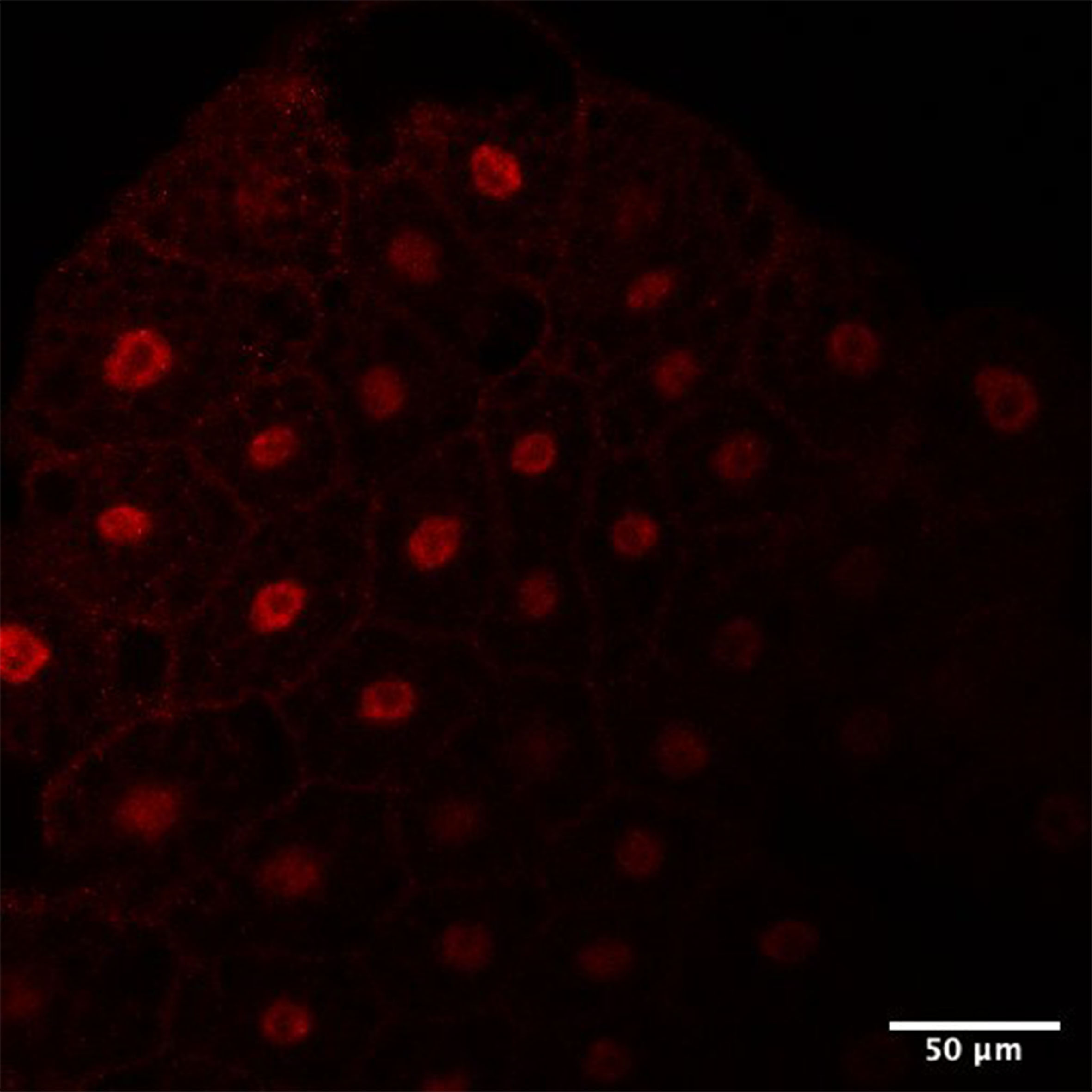

Supplement: Supplementary file 8 — Source Data for Figure 5 [file EMBR-24-e57695-s001.zip › Figure 5/G-H/CG mChRi pmad scale bar copy.jpg]

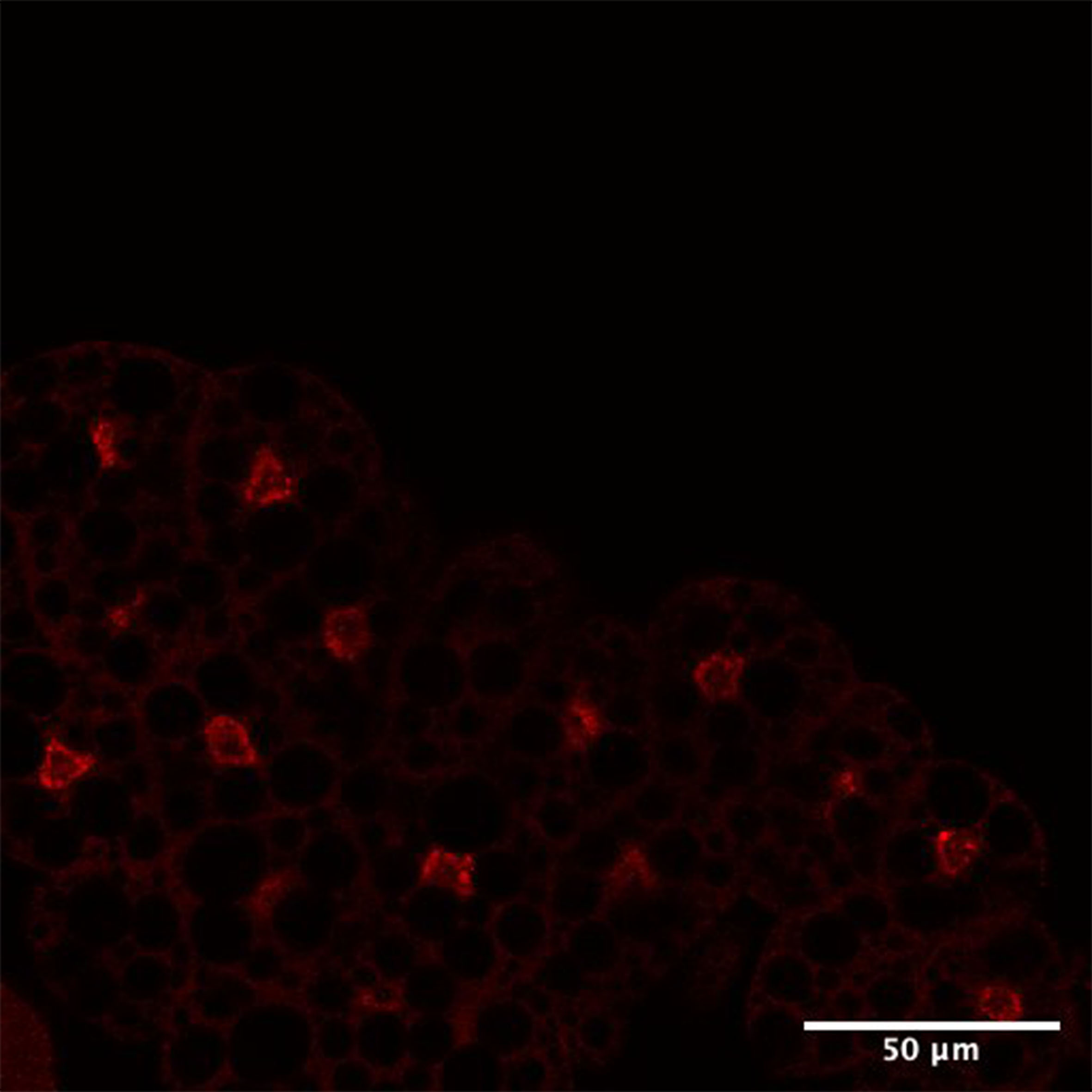

Supplement: Supplementary file 8 — Source Data for Figure 5 [file EMBR-24-e57695-s001.zip › Figure 5/A-B/Starved pMad scale bar copy.jpg]

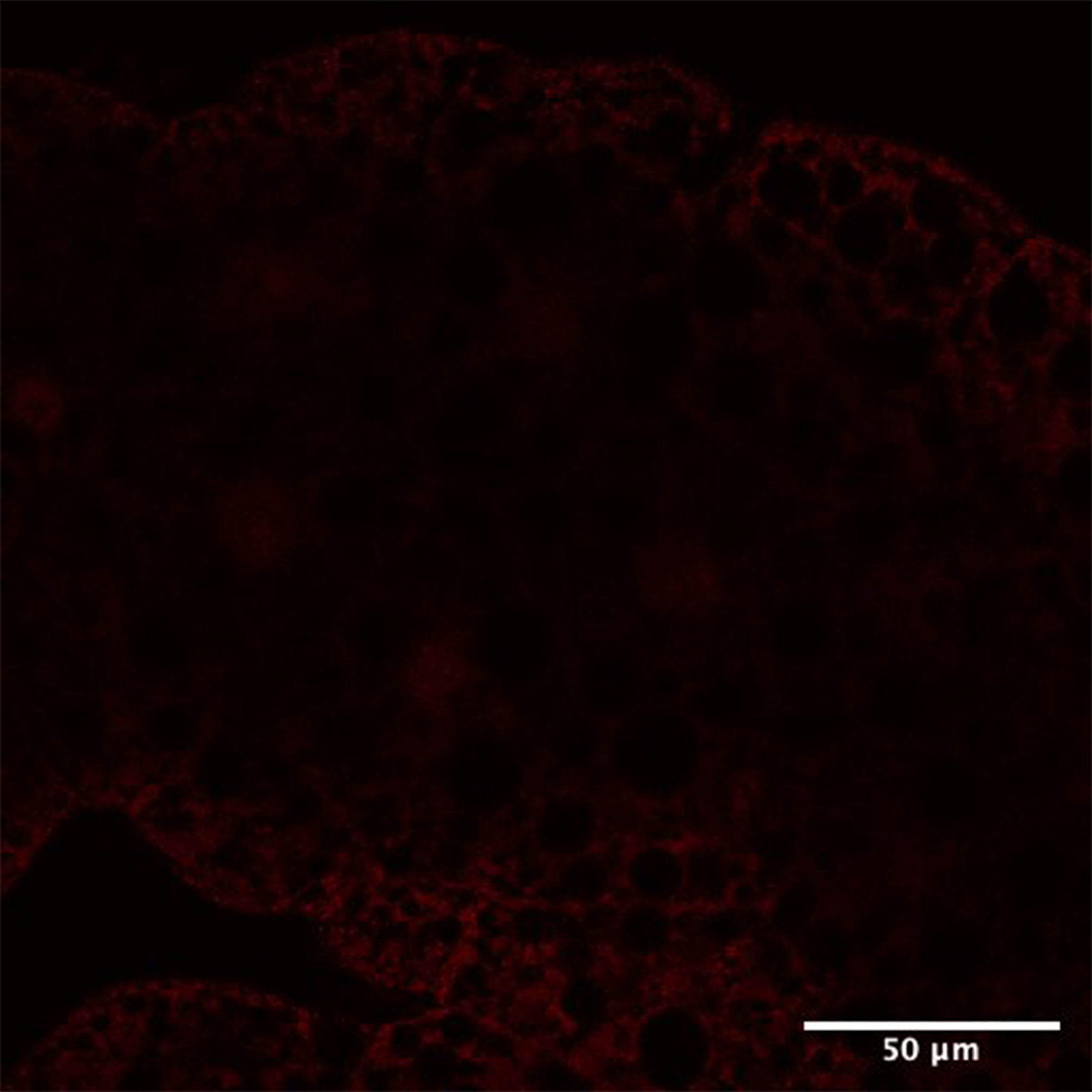

Supplement: Supplementary file 8 — Source Data for Figure 5 [file EMBR-24-e57695-s001.zip › Figure 5/A-B/Fed pMad scale bar copy.jpg]

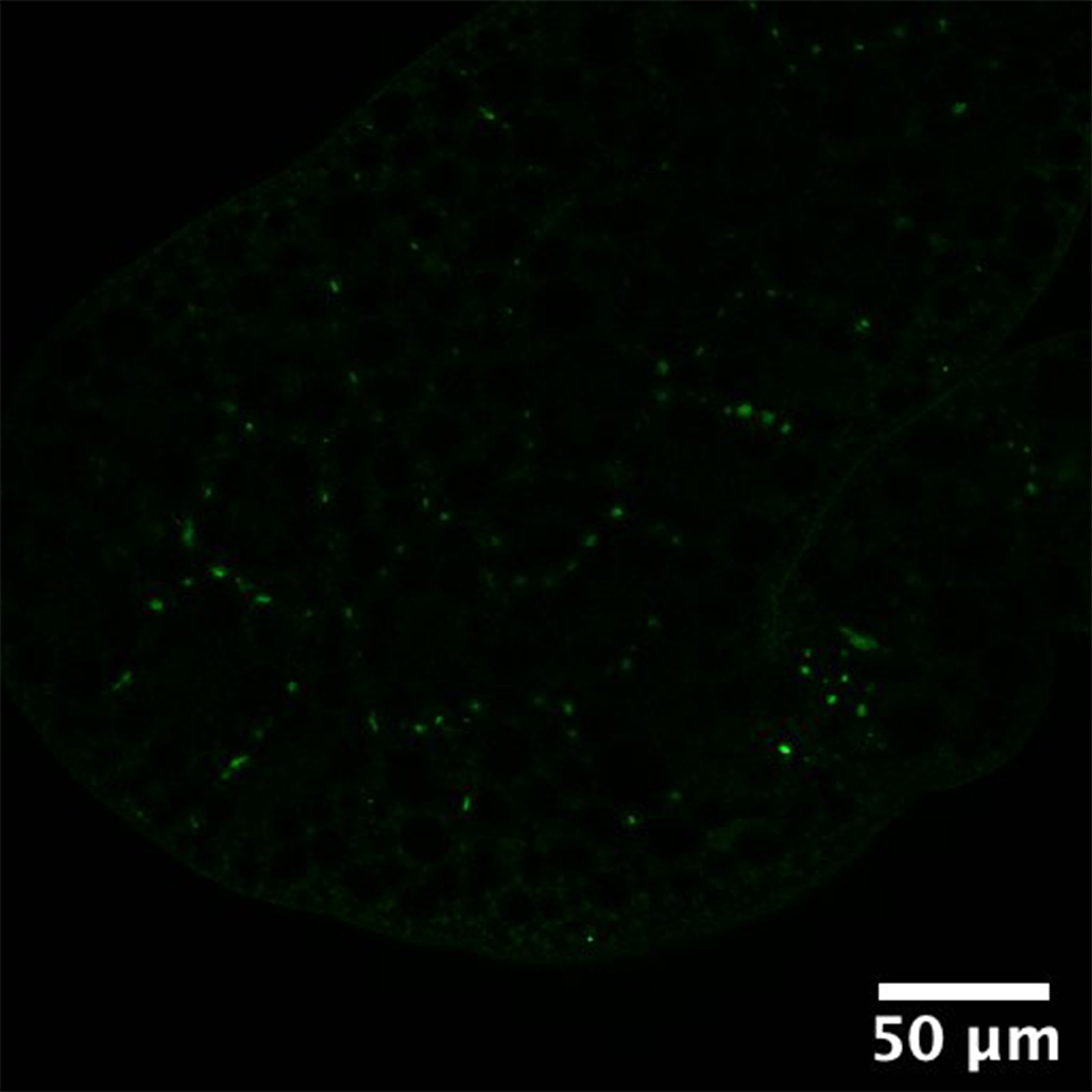

Supplement: Supplementary file 9 — Source Data for Figure 6 [file EMBR-24-e57695-s011.zip › Figure 6/E-F/mcherryRi scale bar copy.jpg]

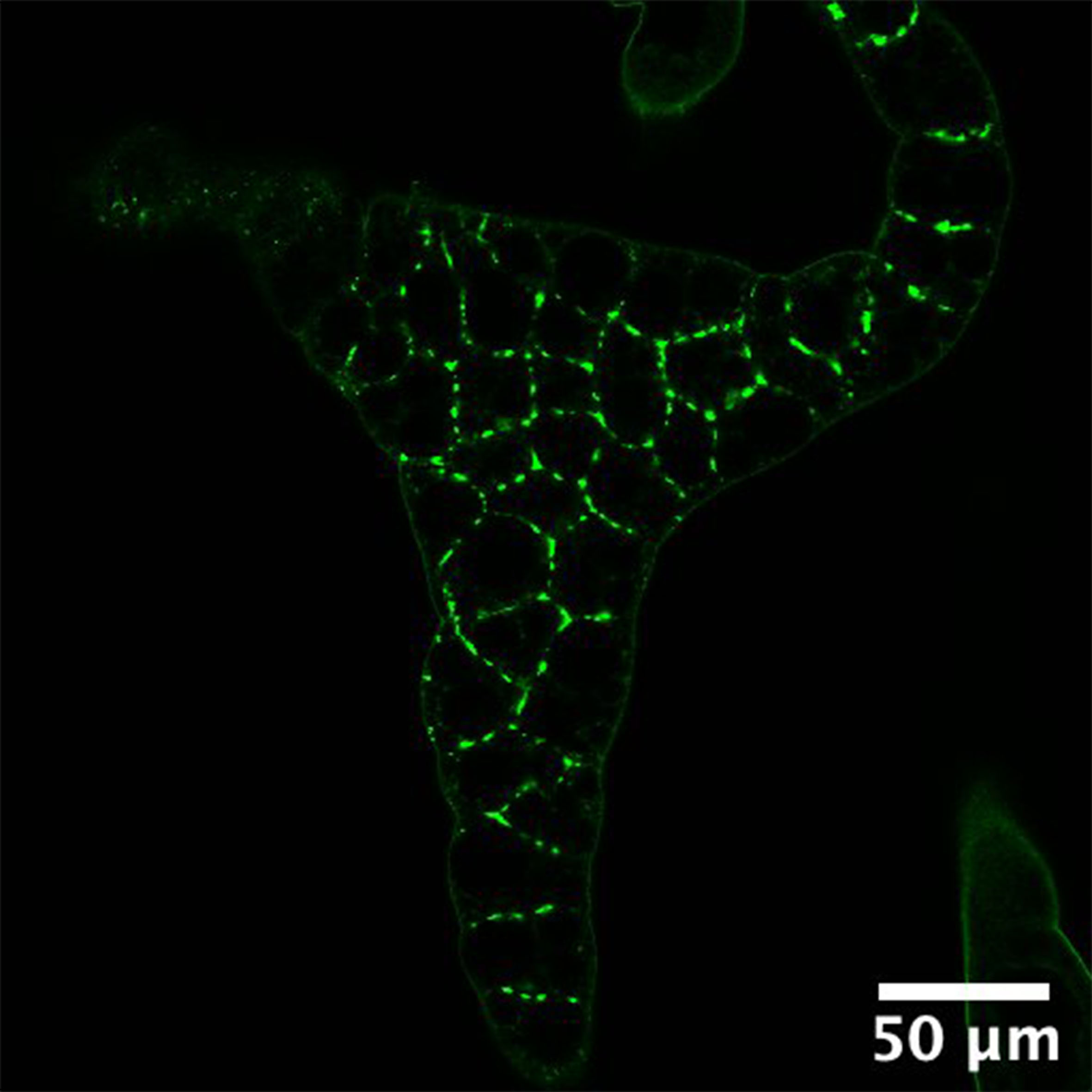

Supplement: Supplementary file 9 — Source Data for Figure 6 [file EMBR-24-e57695-s011.zip › Figure 6/E-F/p60_0001 scale bar copy.jpg]

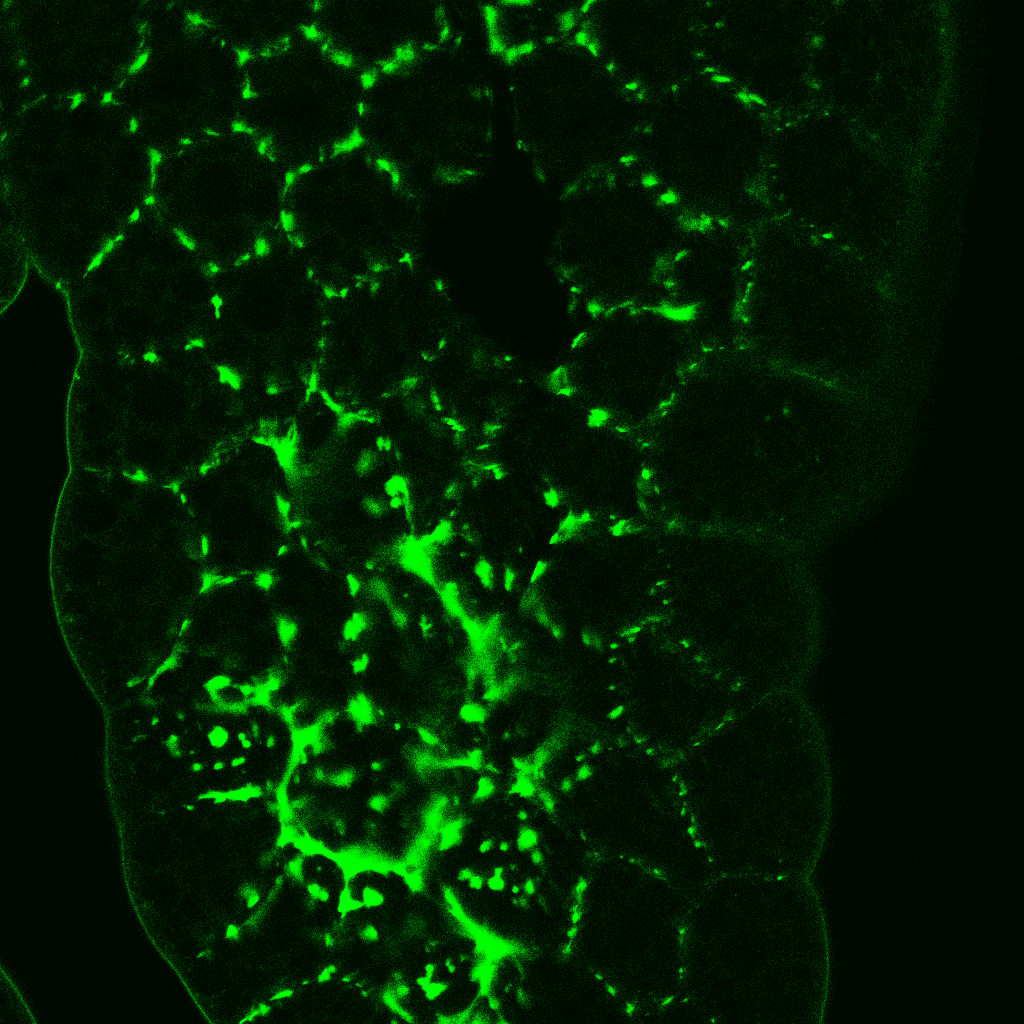

Supplement: Supplementary file 9 — Source Data for Figure 6 [file EMBR-24-e57695-s011.zip › Figure 6/I-K/p60 copy.png]

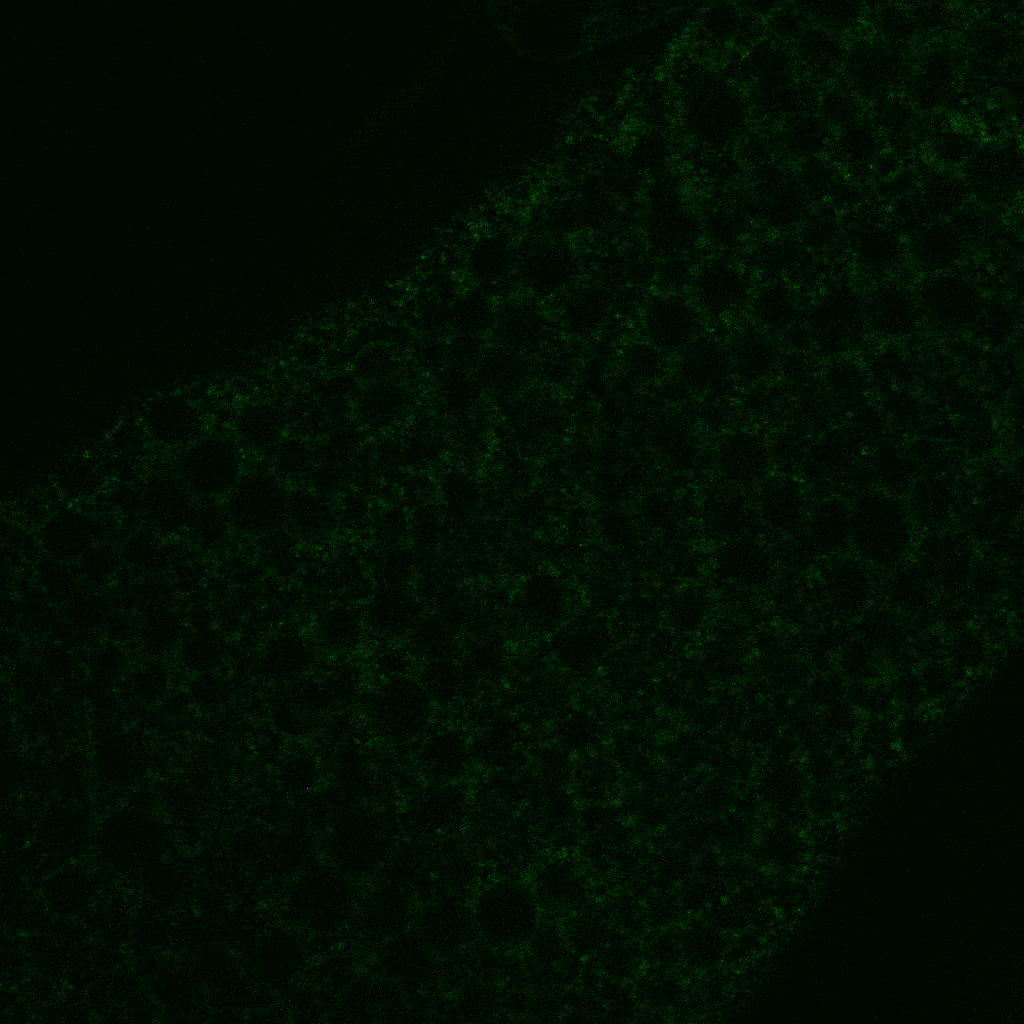

Supplement: Supplementary file 9 — Source Data for Figure 6 [file EMBR-24-e57695-s011.zip › Figure 6/I-K/p60 madri copy.png]

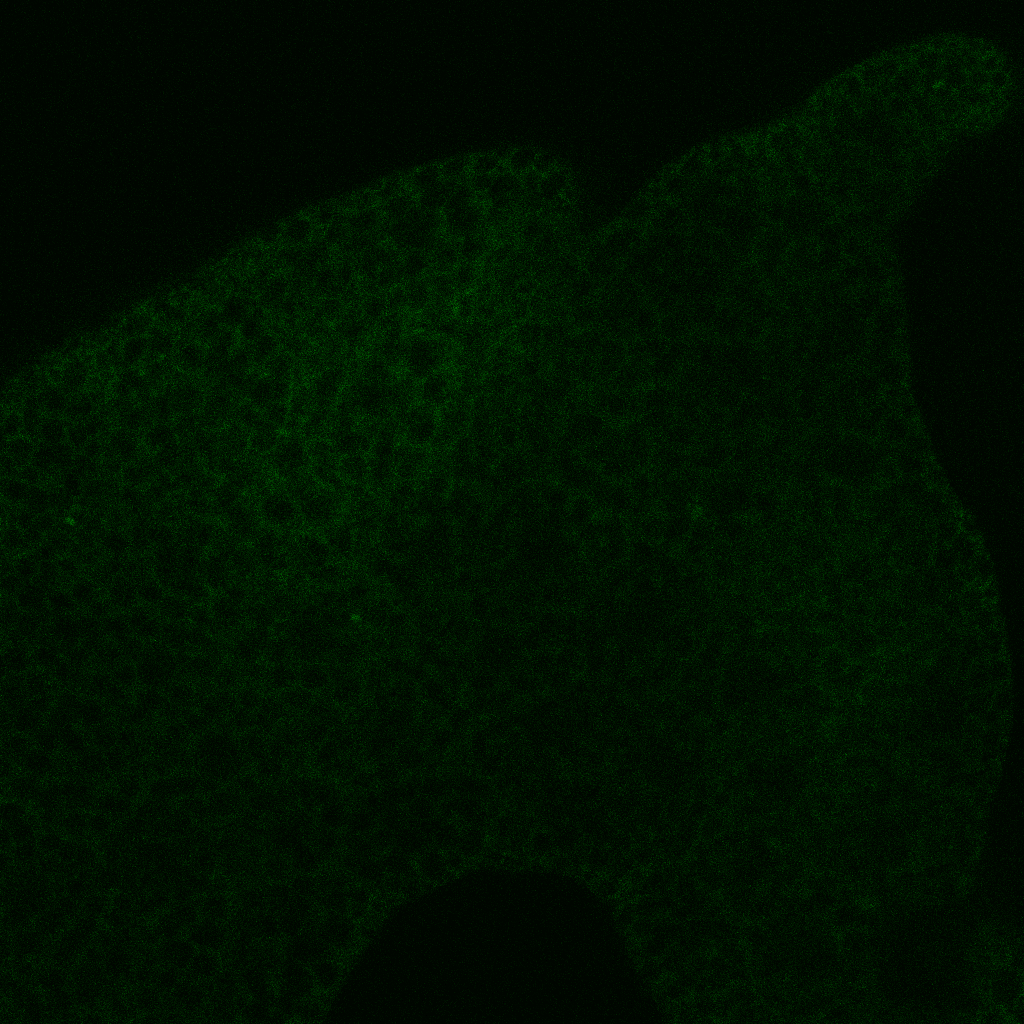

Supplement: Supplementary file 9 — Source Data for Figure 6 [file EMBR-24-e57695-s011.zip › Figure 6/I-K/madri copy.png]

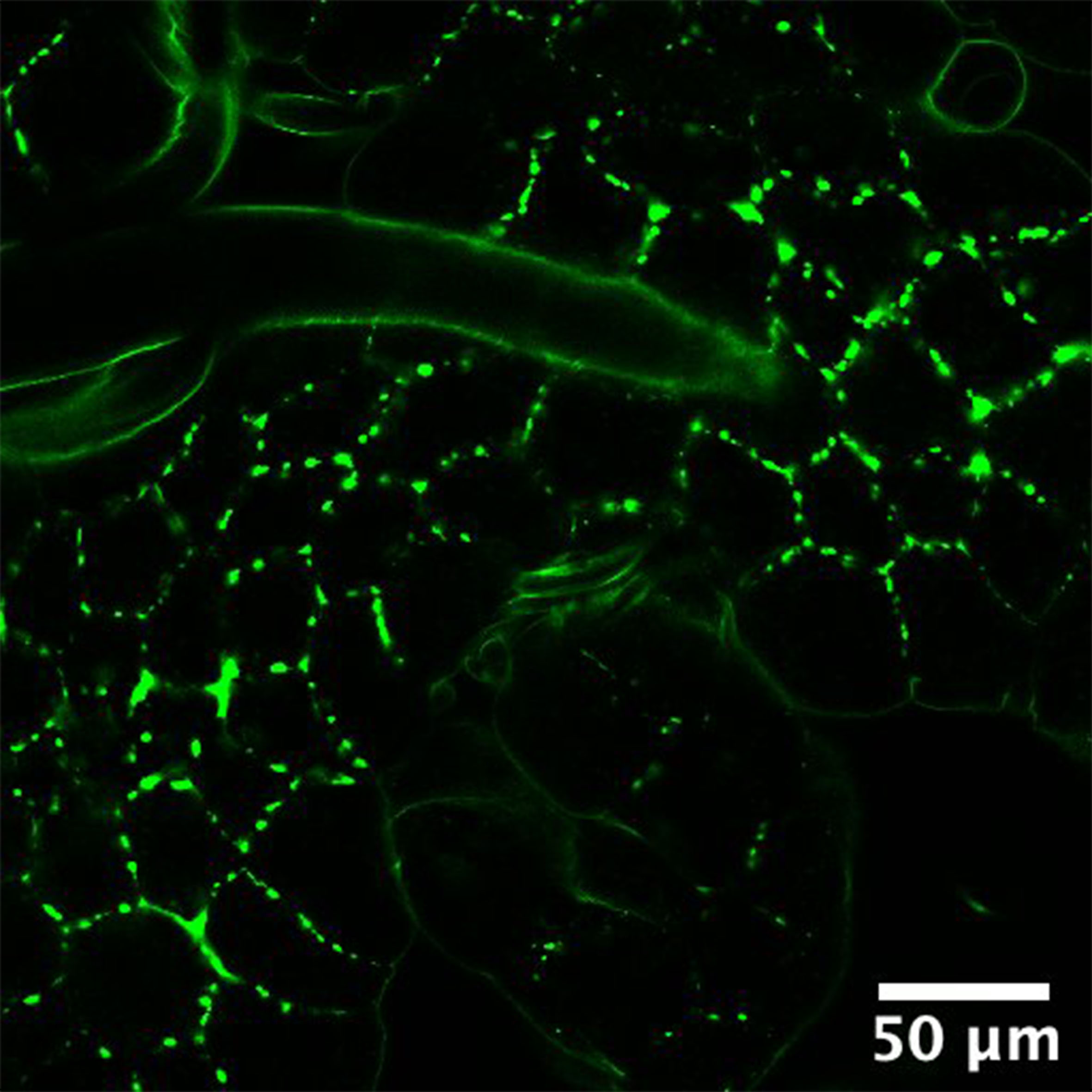

Supplement: Supplementary file 9 — Source Data for Figure 6 [file EMBR-24-e57695-s011.zip › Figure 6/A-B/torDN_0002 scale bar copy.jpg]

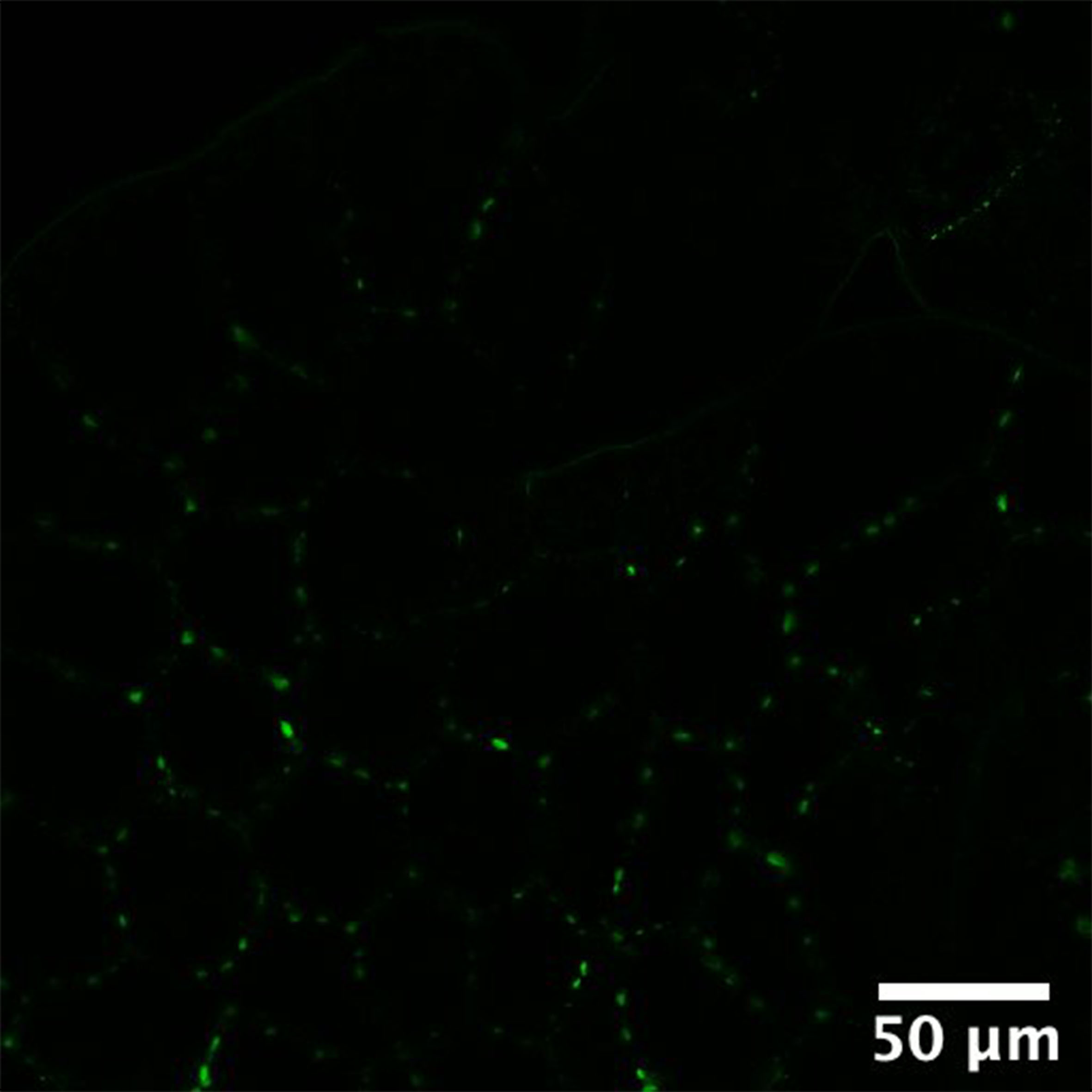

Supplement: Supplementary file 9 — Source Data for Figure 6 [file EMBR-24-e57695-s011.zip › Figure 6/A-B/mcherryRi scale bar copy.jpg]

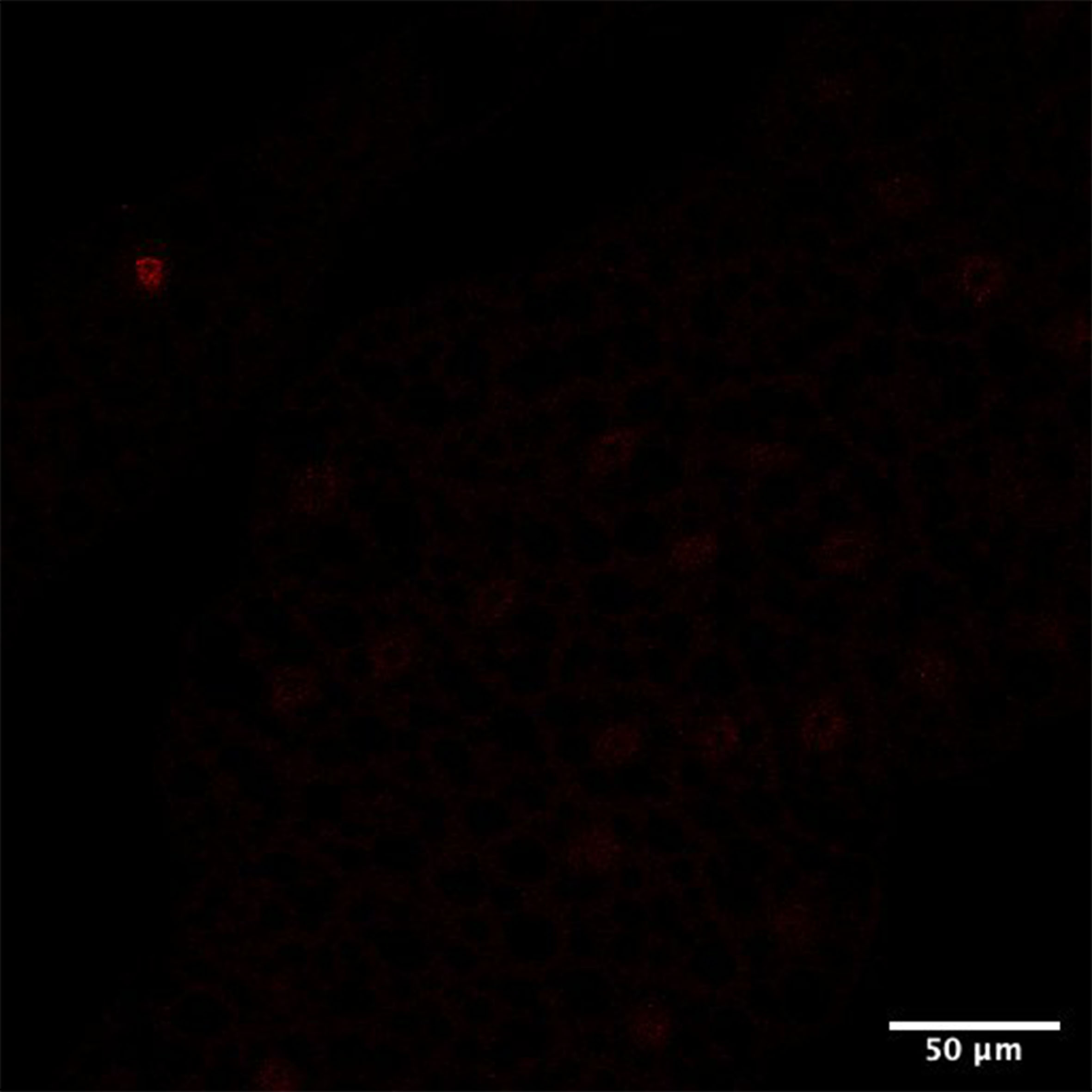

Supplement: Supplementary file 10 — Source Data for Figure 7 [file EMBR-24-e57695-s008.zip › Figure 7/D-E/CG>mChRi pmad scale bar copy.jpg]

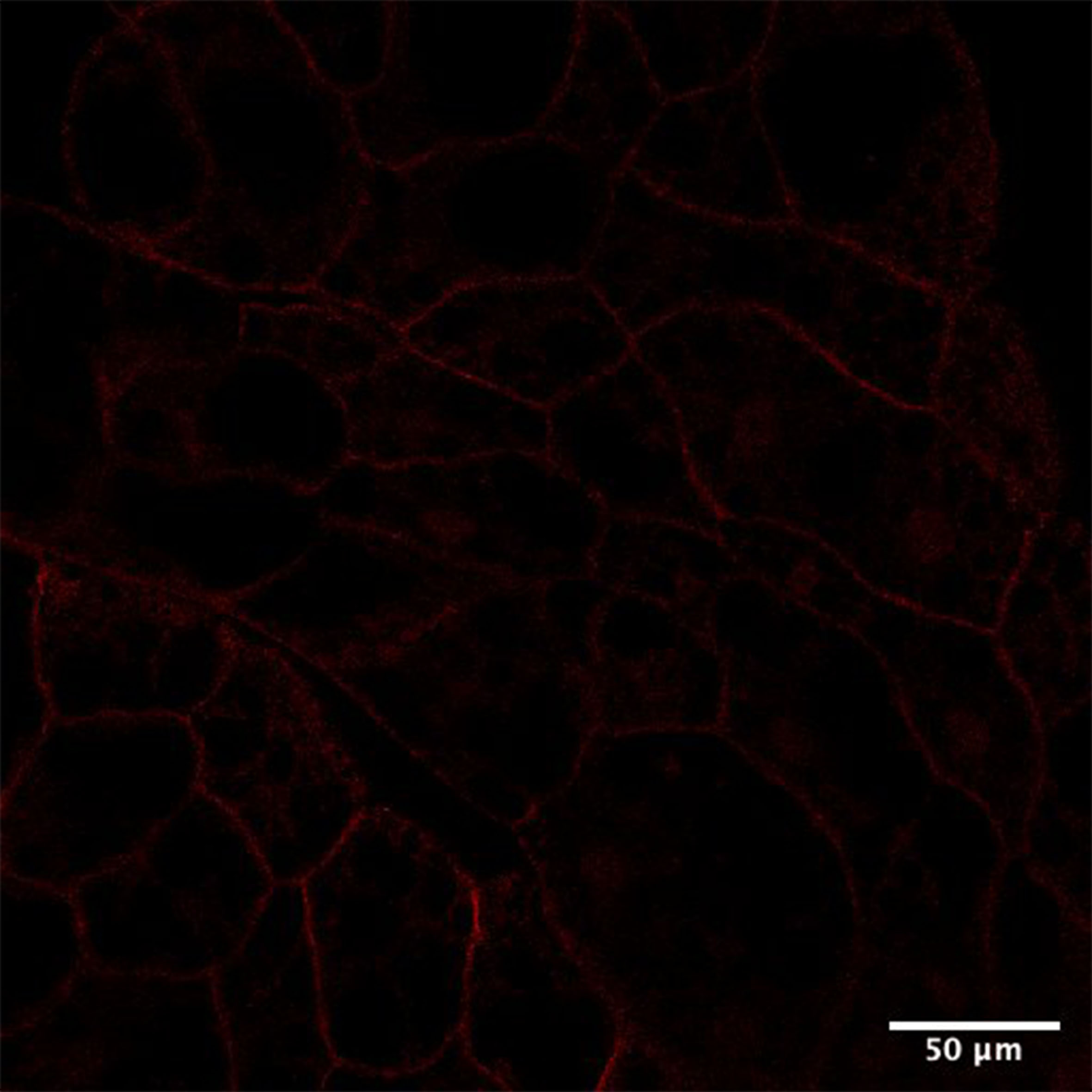

Supplement: Supplementary file 10 — Source Data for Figure 7 [file EMBR-24-e57695-s008.zip › Figure 7/D-E/CG>thorCA pmad scale bar copy.jpg]

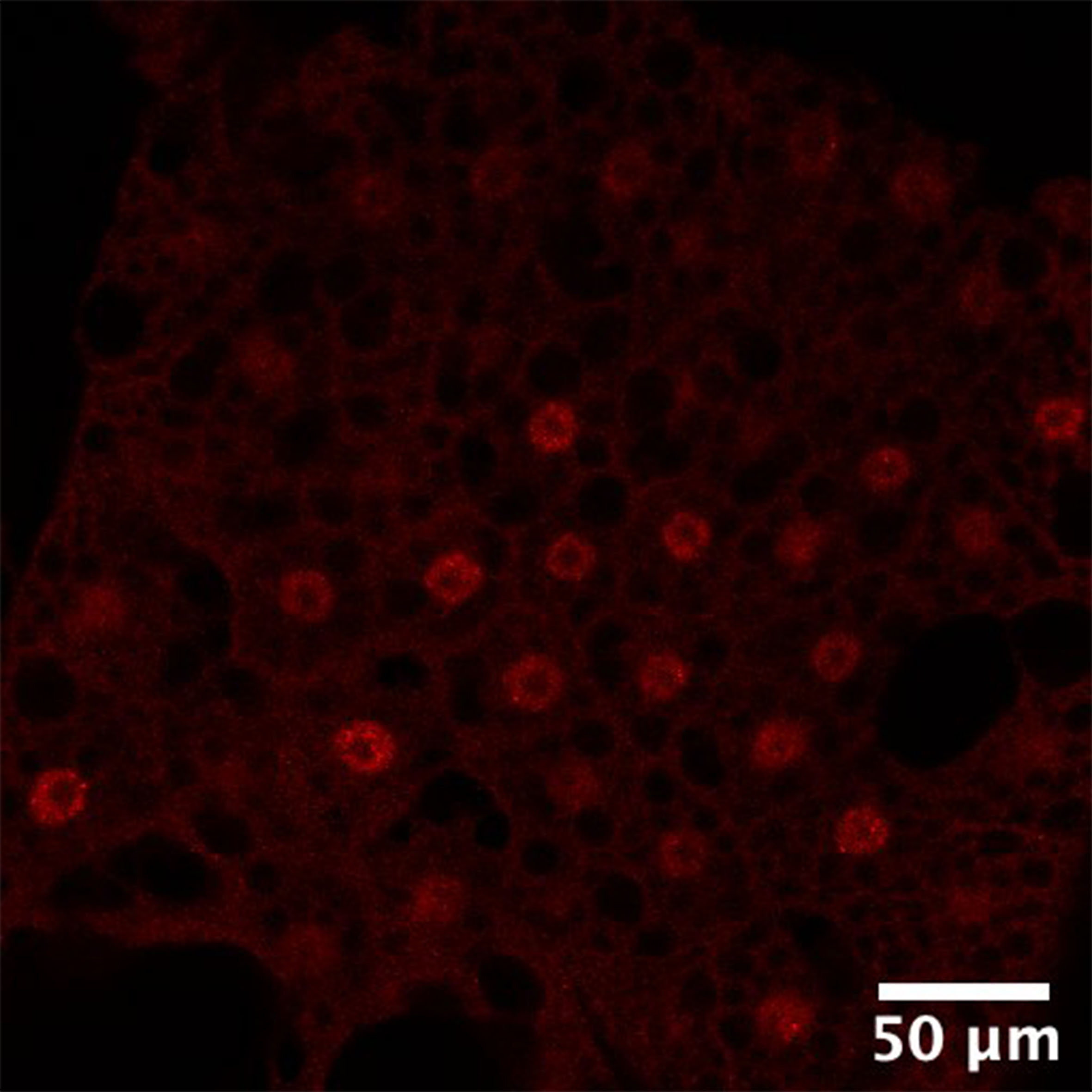

Supplement: Supplementary file 10 — Source Data for Figure 7 [file EMBR-24-e57695-s008.zip › Figure 7/J-K/CGmChRi scale bar copy.jpg]

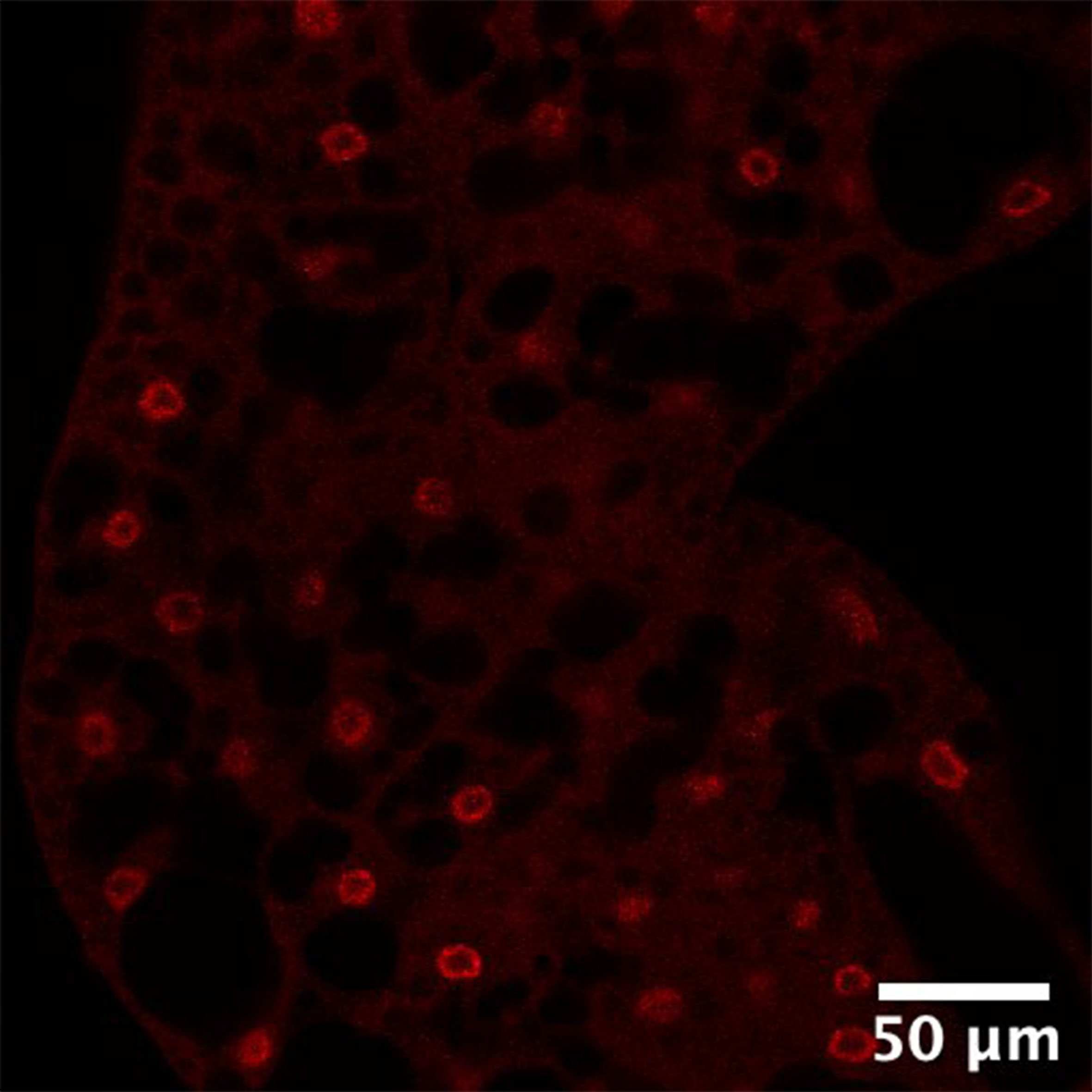

Supplement: Supplementary file 10 — Source Data for Figure 7 [file EMBR-24-e57695-s008.zip › Figure 7/J-K/CG>FOXO scale bar copy.jpg]

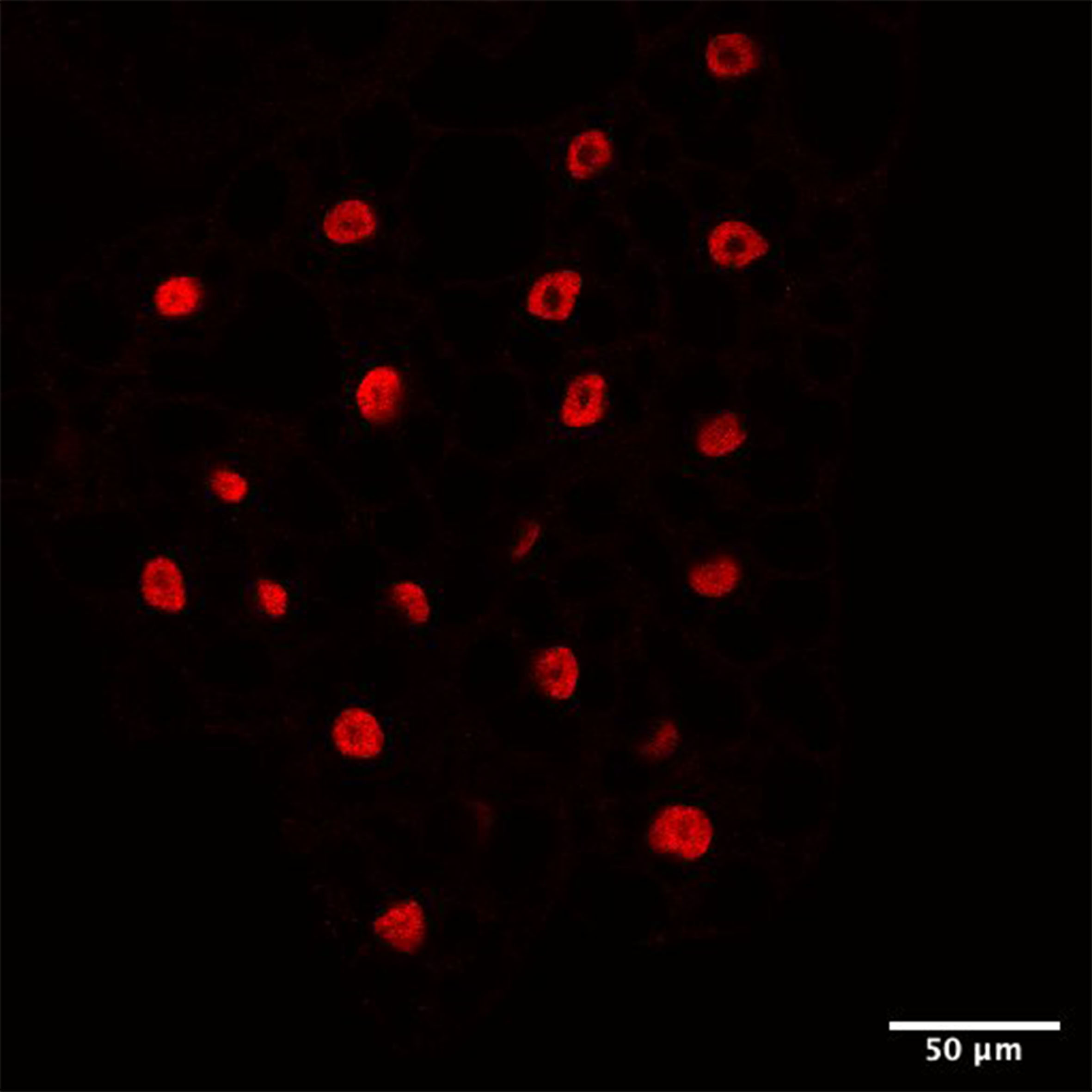

Supplement: Supplementary file 10 — Source Data for Figure 7 [file EMBR-24-e57695-s008.zip › Figure 7/G-H/CGmChRi for S6KCA pMad scale bar copy.jpg]

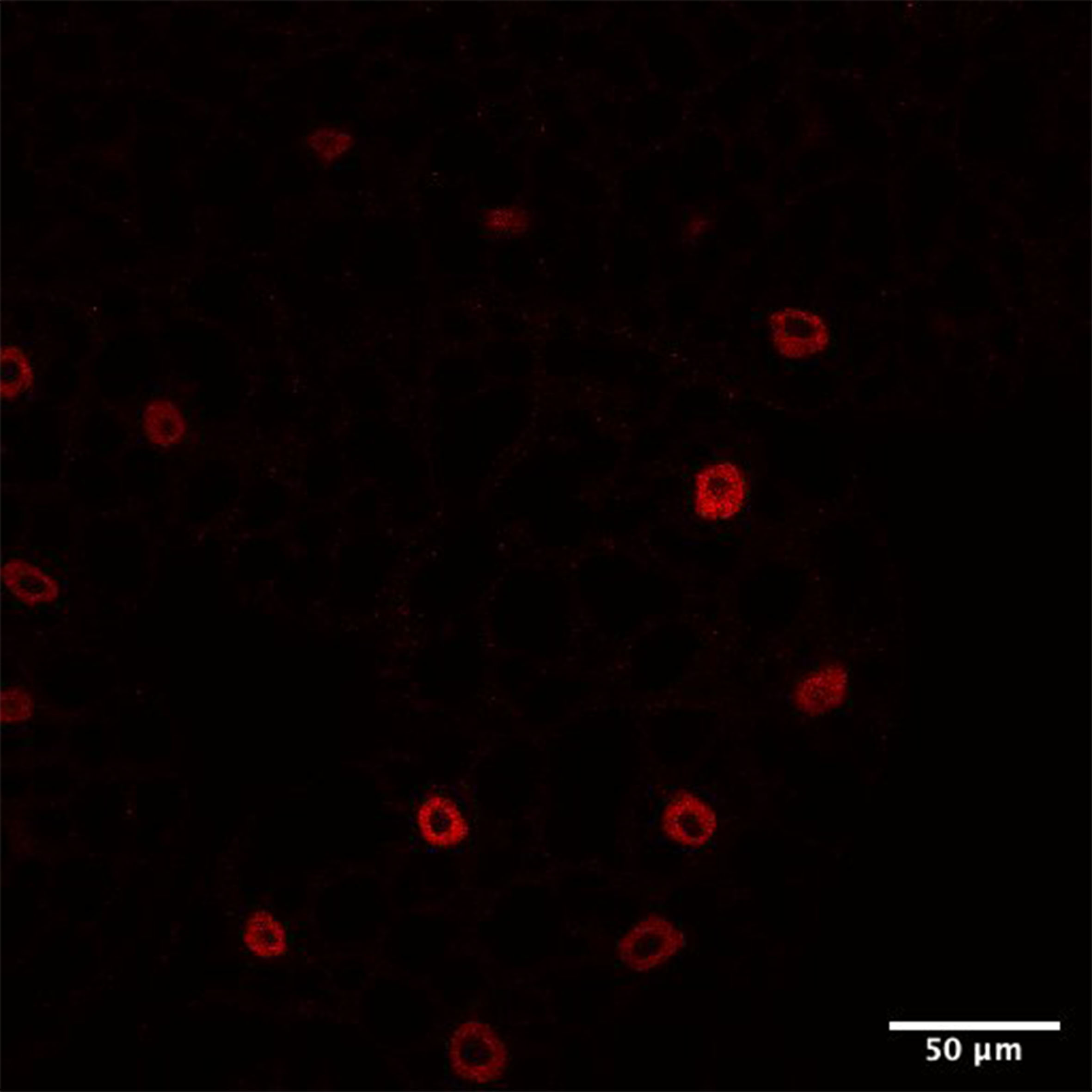

Supplement: Supplementary file 10 — Source Data for Figure 7 [file EMBR-24-e57695-s008.zip › Figure 7/G-H/CG>S6KCA fb pMad scale bar copy.jpg]

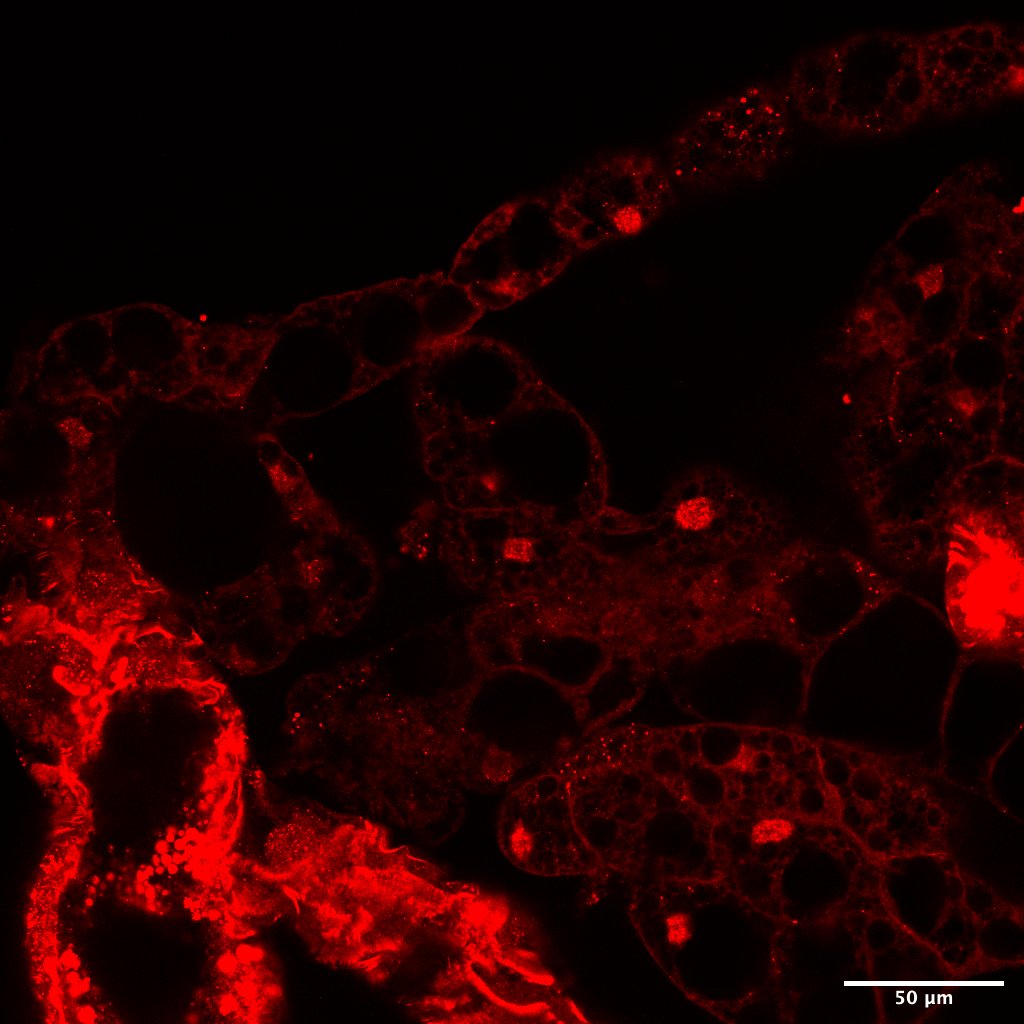

Supplement: Supplementary file 10 — Source Data for Figure 7 [file EMBR-24-e57695-s008.zip › Figure 7/A-B/CG>torDN pmad scale bar.jpg]

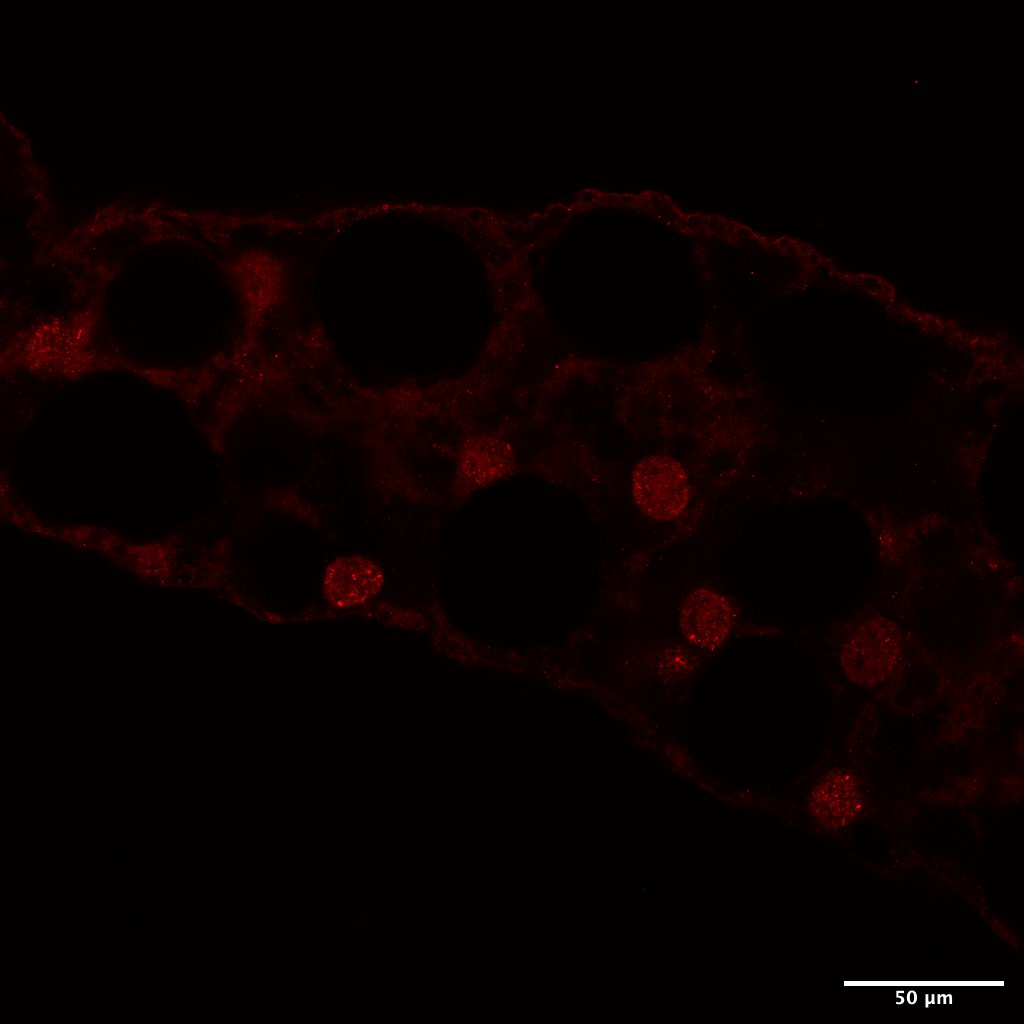

Supplement: Supplementary file 10 — Source Data for Figure 7 [file EMBR-24-e57695-s008.zip › Figure 7/A-B/CG>mChRi pmad scale bar.jpg]

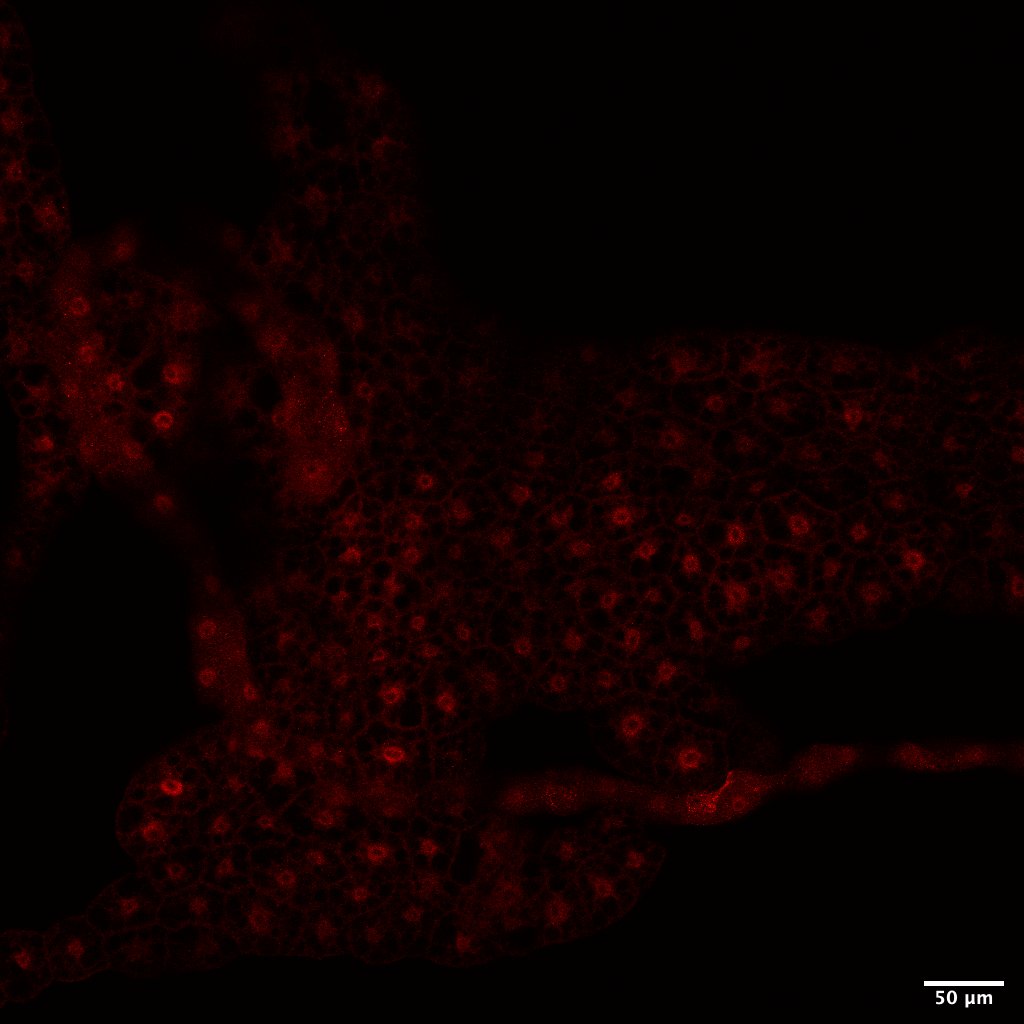

Supplement: Supplementary file 10 — Source Data for Figure 7 [file EMBR-24-e57695-s008.zip › Figure 7/M-P/R4>TorDN/C2-tordn-1 scale.jpg]

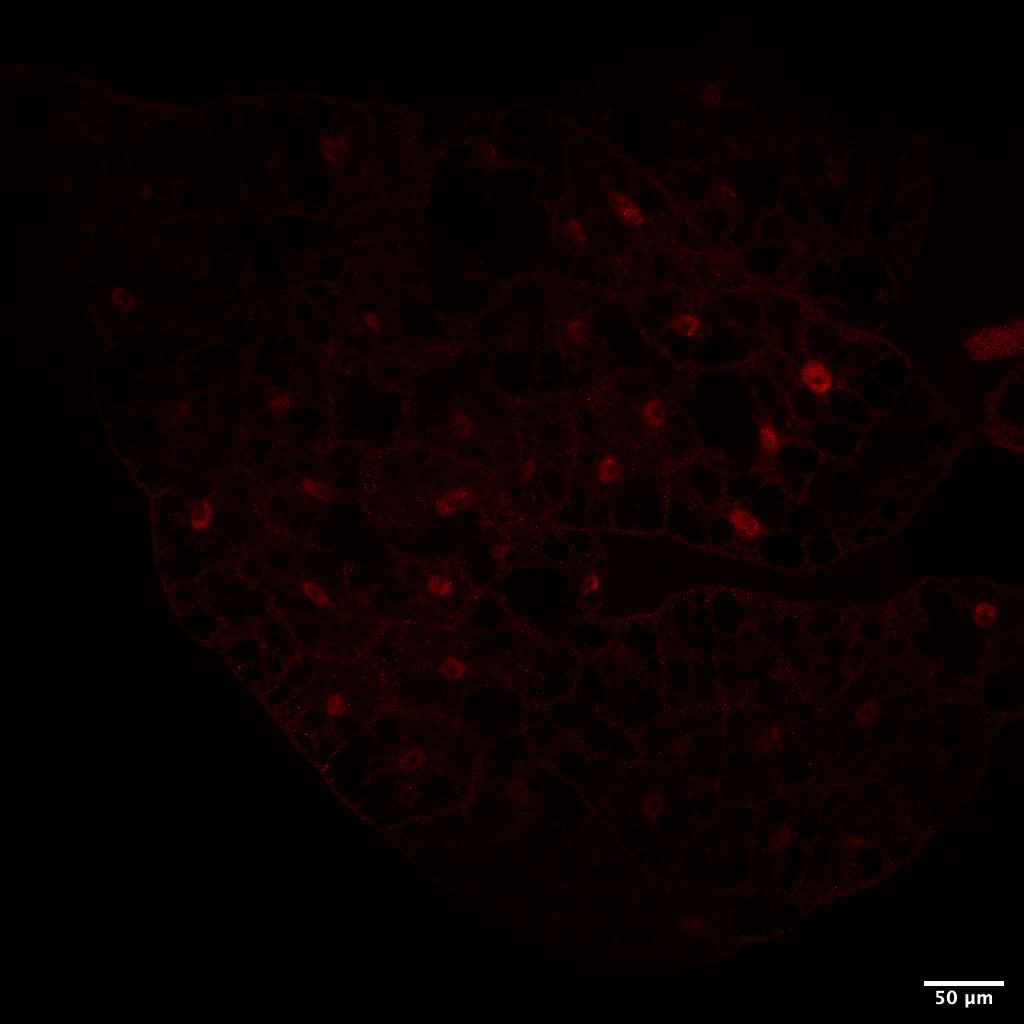

Supplement: Supplementary file 10 — Source Data for Figure 7 [file EMBR-24-e57695-s008.zip › Figure 7/M-P/R4>InRCA;TorDN/C2-inRCA tordn_0001-1 scale.jpg]

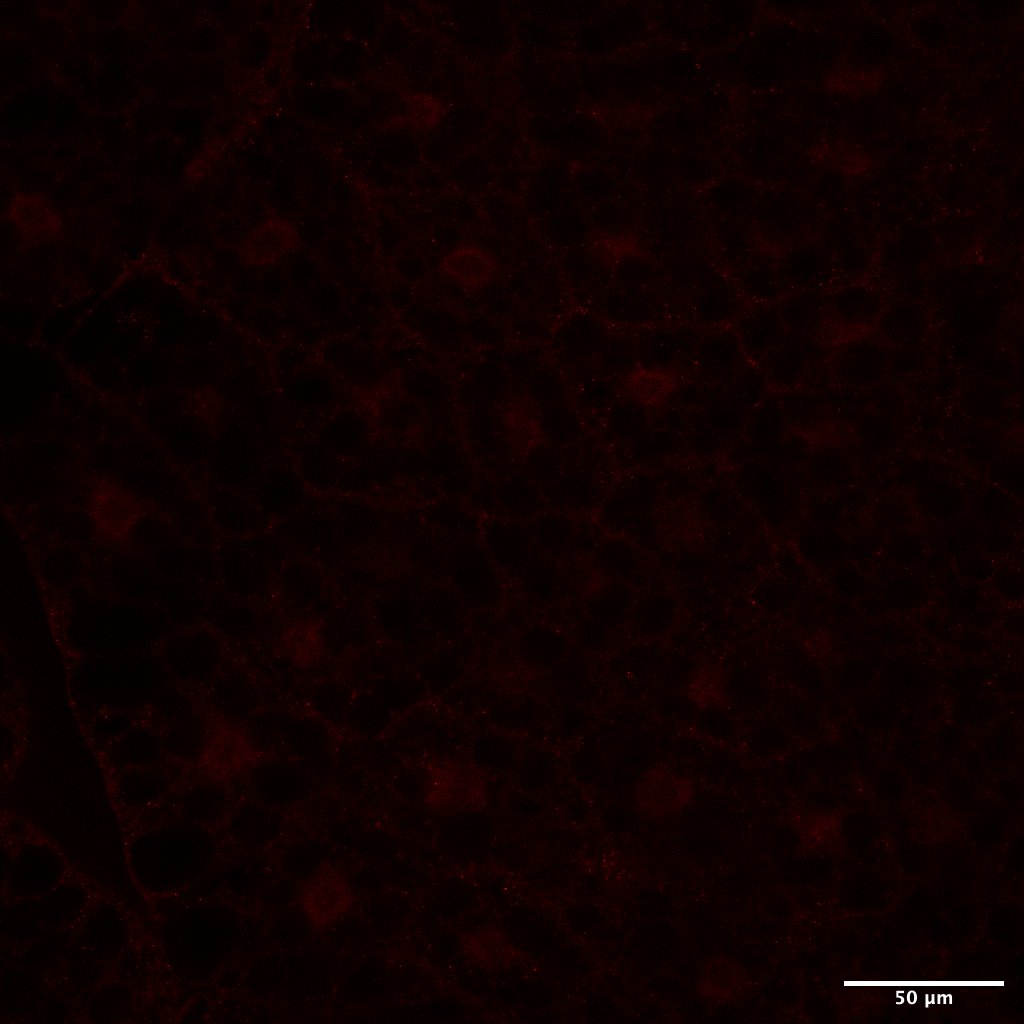

Supplement: Supplementary file 10 — Source Data for Figure 7 [file EMBR-24-e57695-s008.zip › Figure 7/M-P/R4>mcherryri/C2-mcherry r4 pmad dapi_0003-1 scale.jpg]

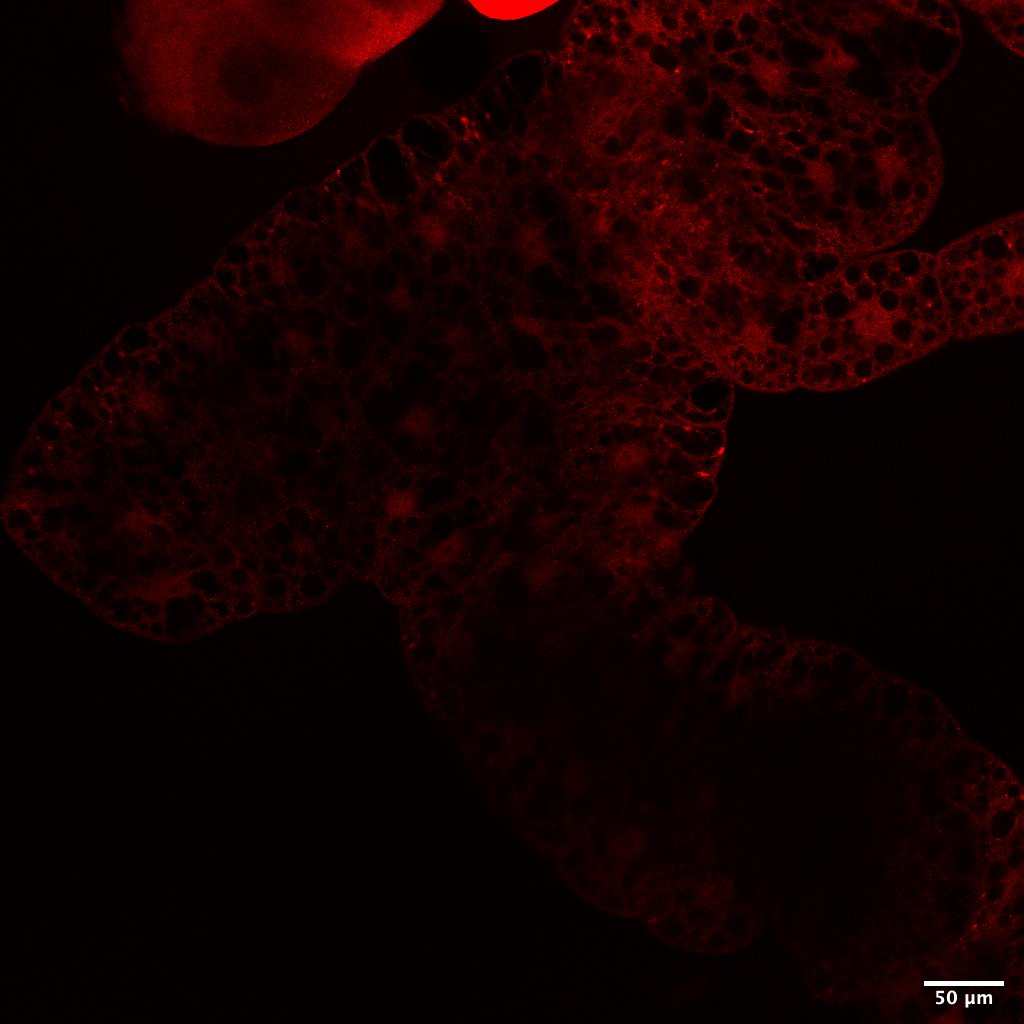

Supplement: Supplementary file 10 — Source Data for Figure 7 [file EMBR-24-e57695-s008.zip › Figure 7/M-P/R4>InRCA;cherryri/C2-inRCA_0001-1 scale.jpg]

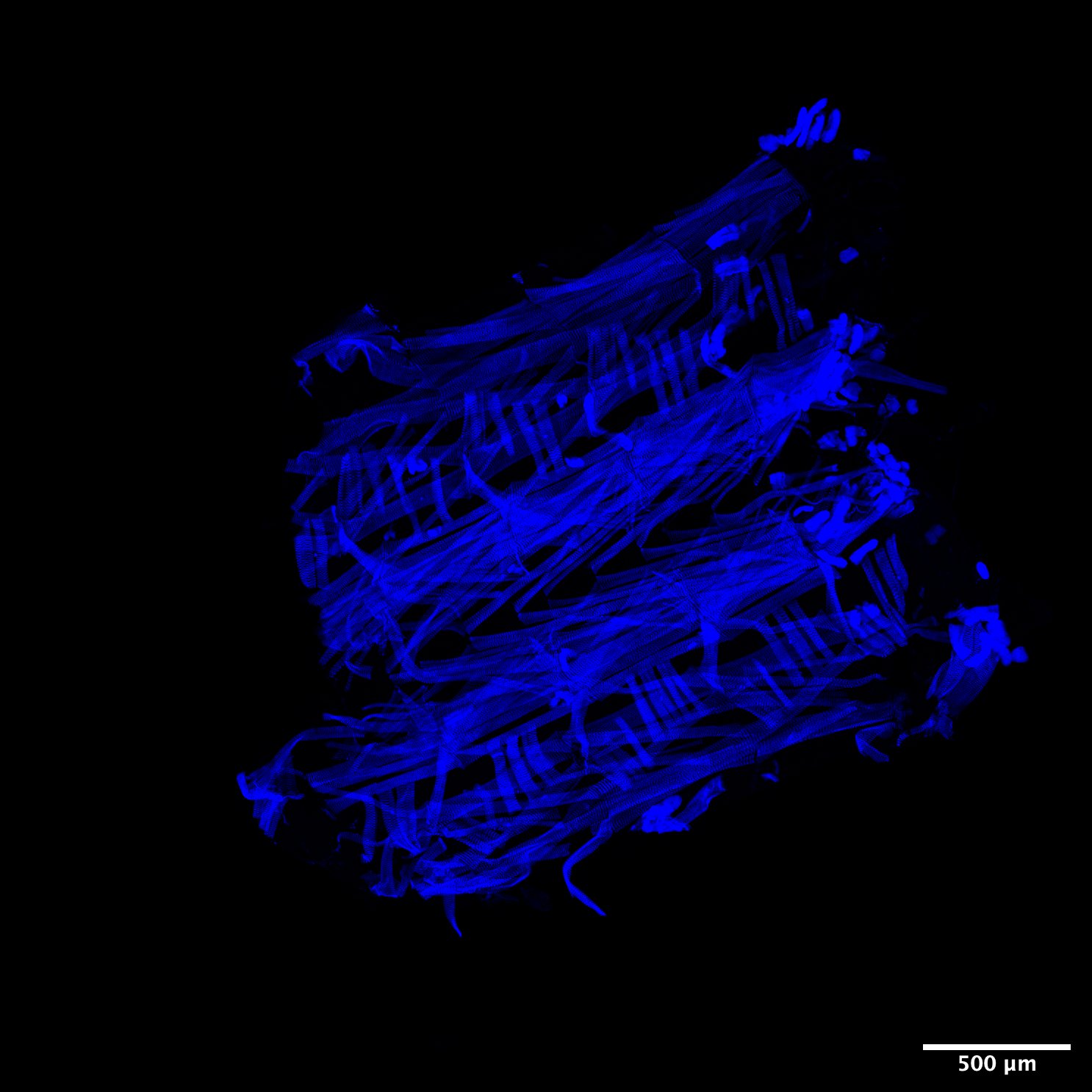

Supplement: Supplementary file 11 — Source Data for Figure 8 [file EMBR-24-e57695-s006.zip › Figure 8/F-G, I, J, L, M/mef2>UASSog scale bar.jpg]

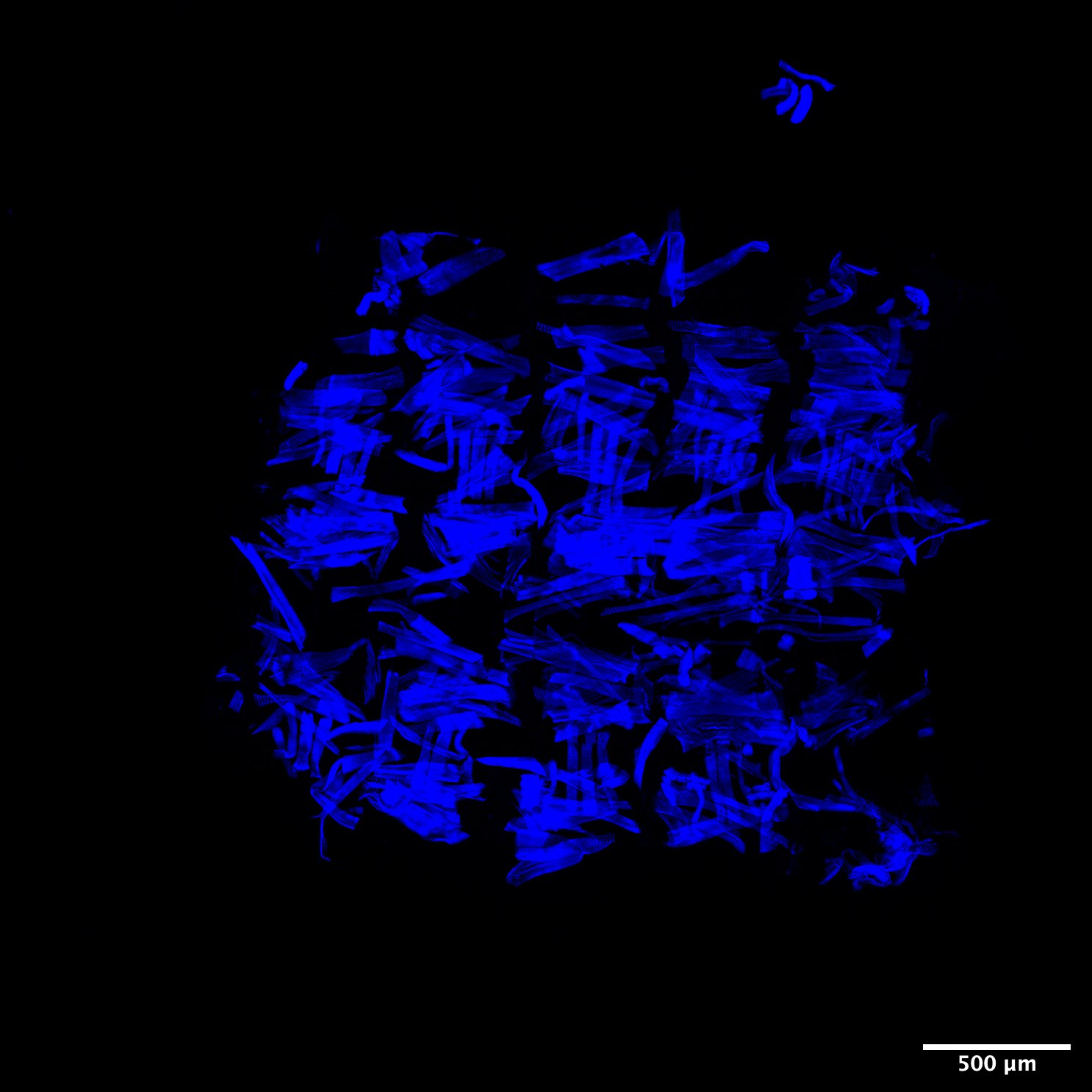

Supplement: Supplementary file 11 — Source Data for Figure 8 [file EMBR-24-e57695-s006.zip › Figure 8/F-G, I, J, L, M/mef2>Luciferase scale bar.jpg]

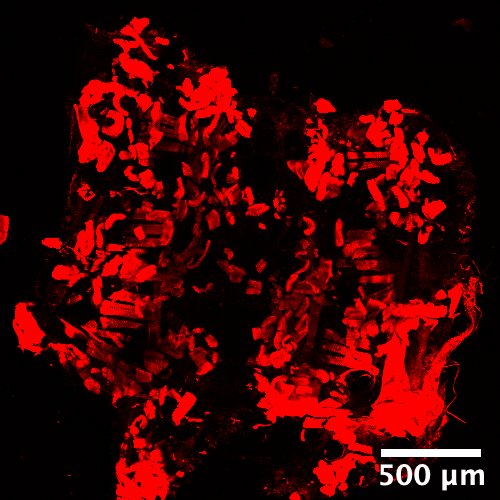

Supplement: Supplementary file 11 — Source Data for Figure 8 [file EMBR-24-e57695-s006.zip › Figure 8/F-G, I, J, L, M/dlg control scale bar.jpg]

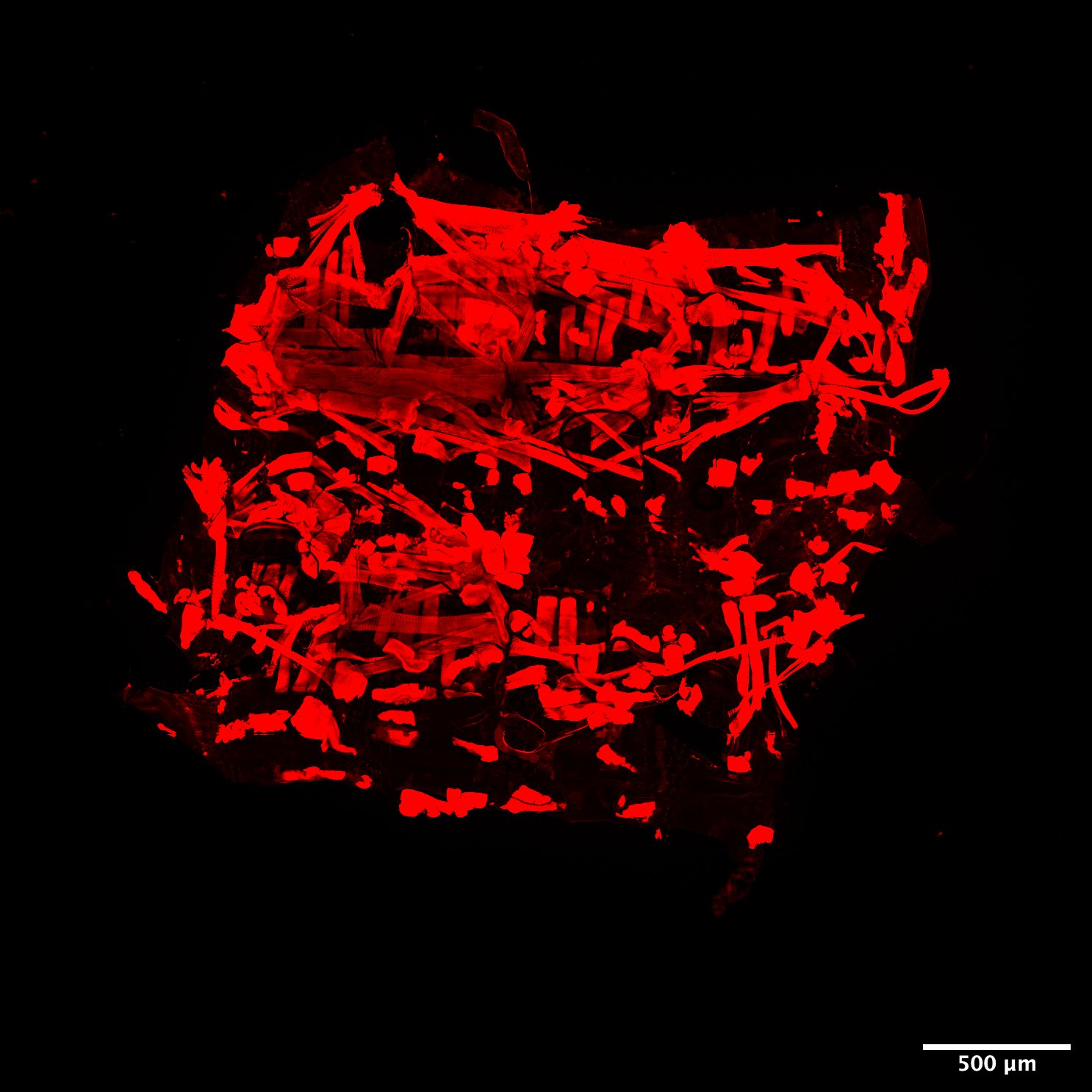

Supplement: Supplementary file 11 — Source Data for Figure 8 [file EMBR-24-e57695-s006.zip › Figure 8/F-G, I, J, L, M/r4 control scale bar.jpg]

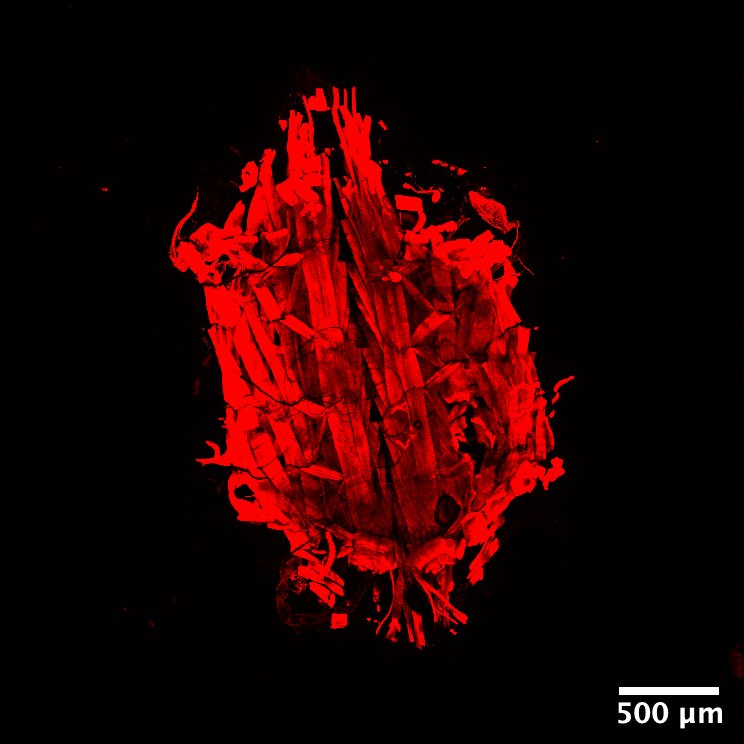

Supplement: Supplementary file 11 — Source Data for Figure 8 [file EMBR-24-e57695-s006.zip › Figure 8/F-G, I, J, L, M/dlg>sog scale bar.jpg]

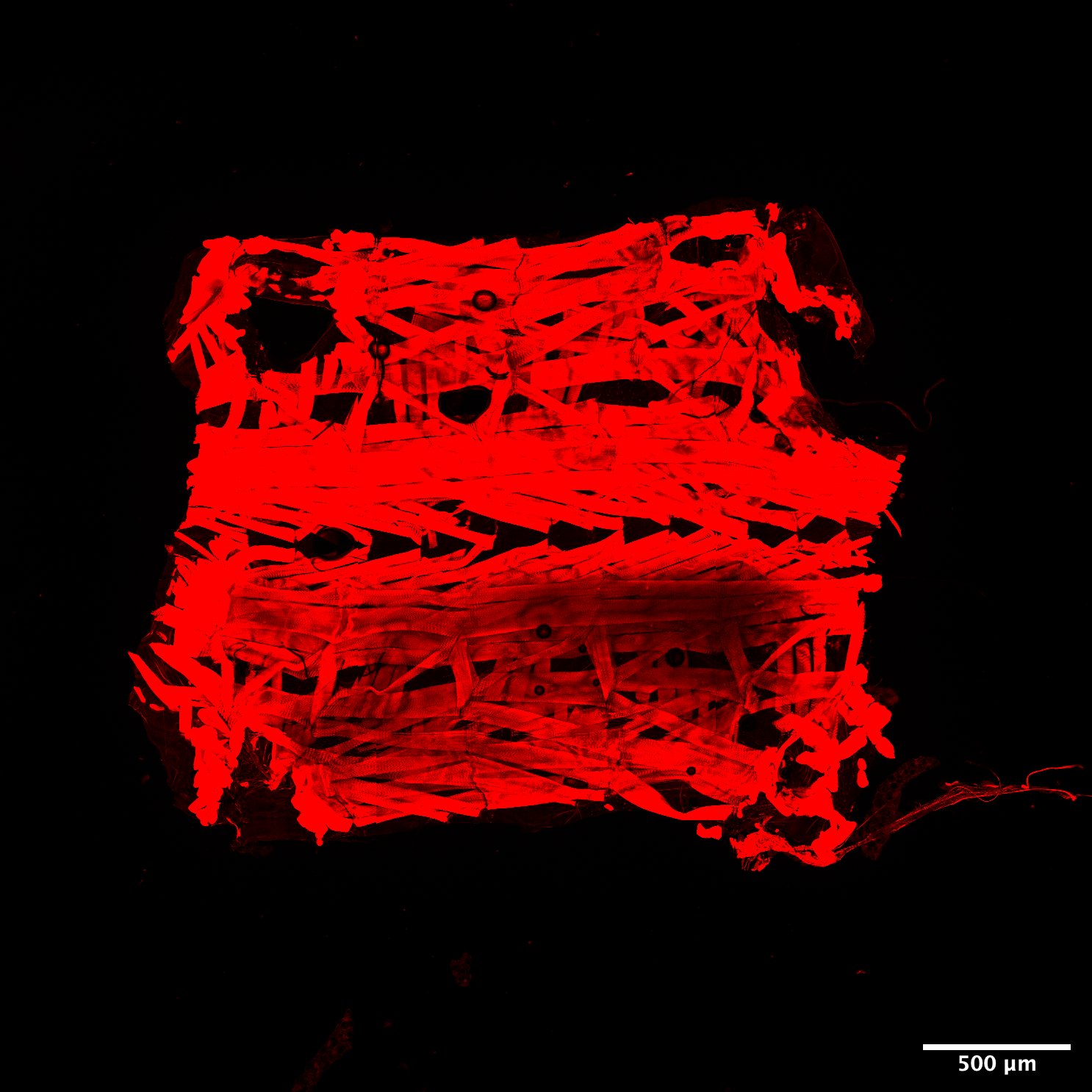

Supplement: Supplementary file 11 — Source Data for Figure 8 [file EMBR-24-e57695-s006.zip › Figure 8/F-G, I, J, L, M/HACKr4sog scale bar.jpg]

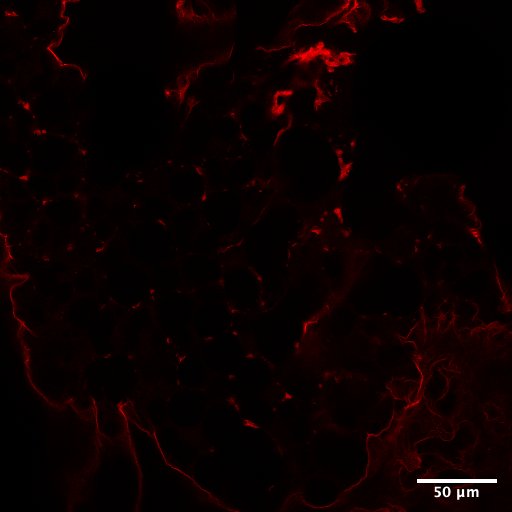

Supplement: Supplementary file 11 — Source Data for Figure 8 [file EMBR-24-e57695-s006.zip › Figure 8/O-P/HACKr4mChRi scale bar.jpg]

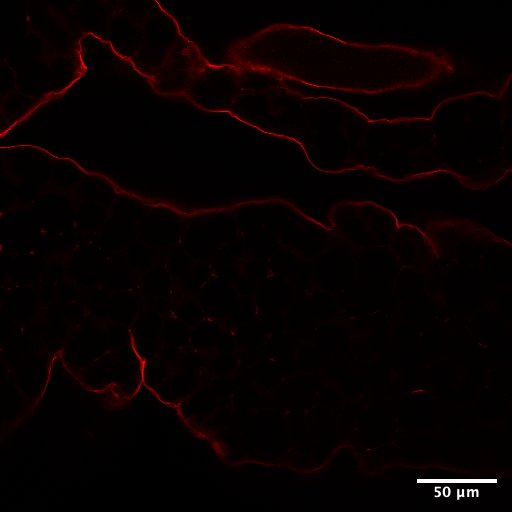

Supplement: Supplementary file 11 — Source Data for Figure 8 [file EMBR-24-e57695-s006.zip › Figure 8/O-P/HACKr4sog scale bar.jpg]

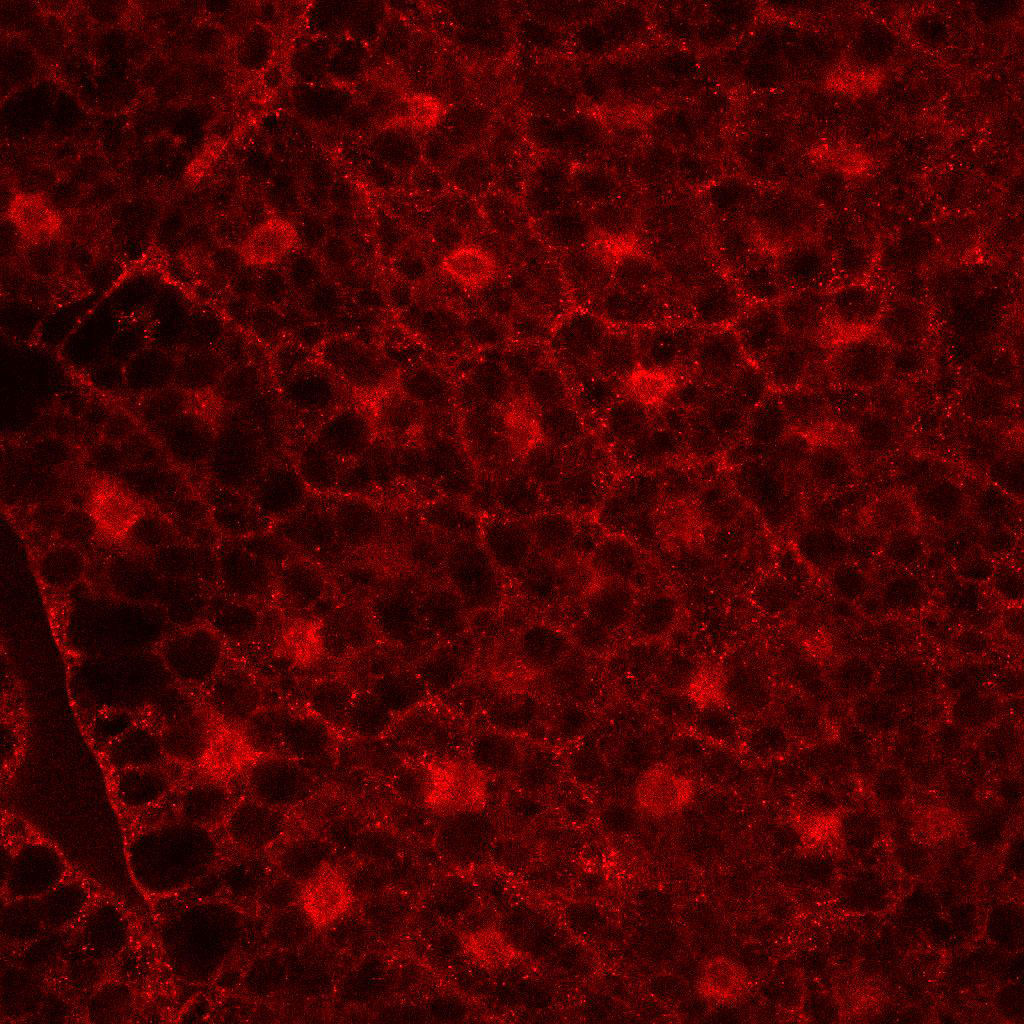

Supplement: Supplementary file 11 — Source Data for Figure 8 [file EMBR-24-e57695-s006.zip › Figure 8/A-D/C2-mcherry r4 pmad dapi_0003-1.jpg]

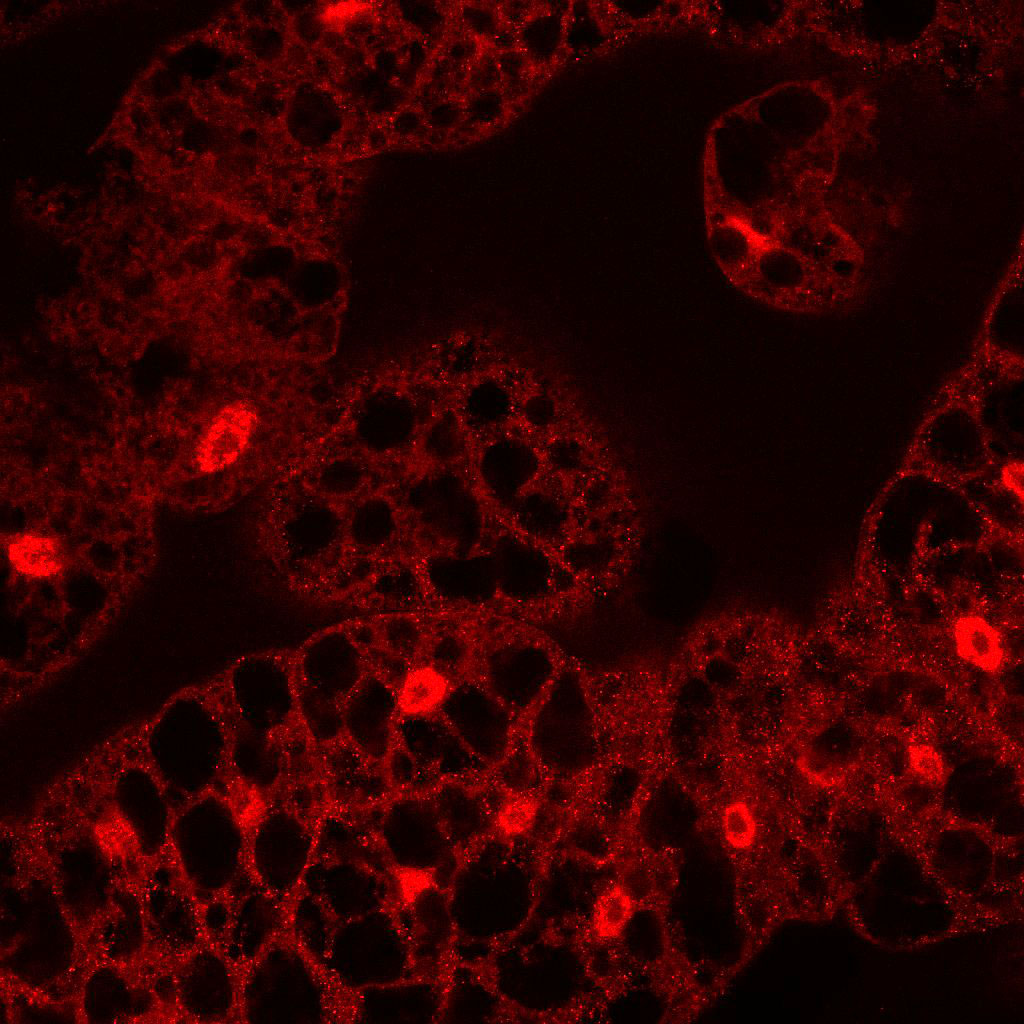

Supplement: Supplementary file 11 — Source Data for Figure 8 [file EMBR-24-e57695-s006.zip › Figure 8/A-D/C2-sogRNai mcherryri r4 pmad dapi_0002-1.jpg]

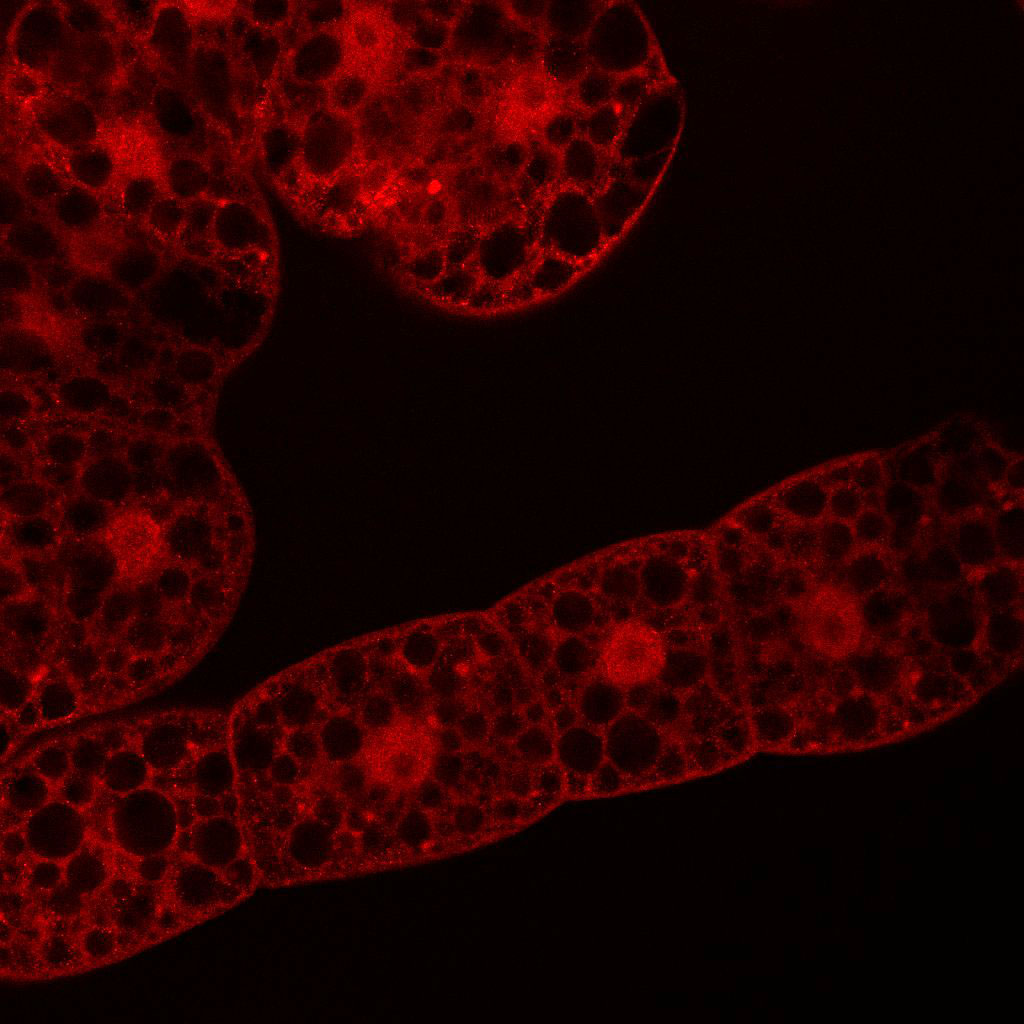

Supplement: Supplementary file 11 — Source Data for Figure 8 [file EMBR-24-e57695-s006.zip › Figure 8/A-D/C2-InRca cherryri r4 pmad dapi_0007.jpg]

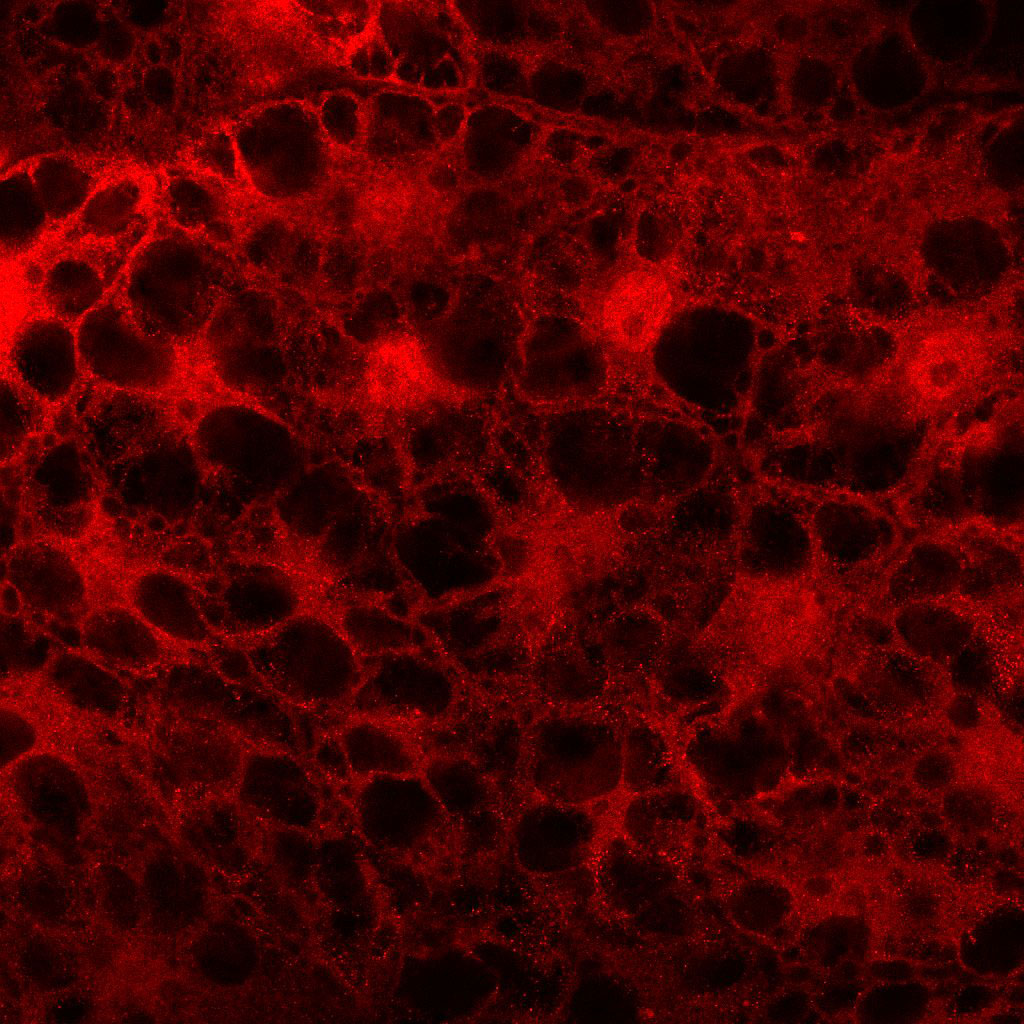

Supplement: Supplementary file 11 — Source Data for Figure 8 [file EMBR-24-e57695-s006.zip › Figure 8/A-D/C2-sogRNai inRCA r4 pmad dapi_0001-1.jpg]
