# Supplementary material for: Convergent insulin and TGF‐β signalling drives cancer cachexia by promoting aberrant fat body ECM accumulation in a Drosophila tumour model
Source: EMBO Rep. 2023 Nov 28;24(12):e57695. doi: 10.15252/embr.202357695 (PMC10702797; doi:10.15252/embr.202357695)
Supplement: Supplementary file 12 [file EMBR-24-e57695-s003.pdf]

EMBO reports

Convergent Insulin and TGF-β signalling drives cancer cachexia

Daniel Bakopoulos, Sofya Golenkina, Callum Dark, Elizabeth Christie, Besaiz Sánchez-Sánchez, Brian Stramer, and Louise Cheng
DOI: 10.15252/embr.202357695

Corresponding author(s): Louise Cheng (louise.cheng@petermac.org)

Review Timeline:

Transfer from Review Commons:	21st Jun 23
Editorial Decision:	30th Jun 23
Revision Received:	30th Aug 23
Editorial Decision:	13th Oct 23
Revision Received:	16th Oct 23
Accepted:	26th Oct 23

Editor: Deniz Senyilmaz Tiebe

Transaction Report: This manuscript was transferred to EMBO reports following peer review at Review Commons.

Review #1

1. Evidence, reproducibility and clarity:

Evidence, reproducibility and clarity (Required)

In this manuscript, Bakopolous et al. investigated on the function of Insulin and TGF beta signaling in the converging regulation of sog (BMP antagonist) and how it controls ECM remodeling. Therefore, the authors used a Drosophila model of cachexia established in Lodge et al., 2021. The authors have shown that the tumors increase Impl2 and Gbb in the fatbody leading to the inhibition of insulin signaling and activation of TGF-β signaling respectively. This lead to the accumulation of ECM proteins that contributes to muscle ECM deficit and muscle detachment. These findings are a major advance in the field of cachexia and of broad interest. The authors demonstrate that state-of-the-art genetics in flies allows acquisition of genetically precise data along with important and complex discoveries on signaling pathways with relevance not only for basic, but for biomedical research as well.

The manuscript is concise and very well written. The experiments overt a clear logical order and are comprehensively described. The authors provide exhaustive data to support their novel claims of broad interest to the scientific community. Please find below some minor recommendations and experiments that could shed further light on some aspects of this manuscript.

Major:

- The statement (line 149'Together, our data suggest that systemic ecdysone levels are unlikely to be involved in modulating tumour-induced muscle detachment or to mediate the role of fatbody Insulin signalling in regulating muscle detachment.') is derived from an experiment with sterol free diet (in which 20HE is genetically addressed) and a pleiotropic experiment (PG>RasG12V). In neither paper nor the current manuscript, 20HE levels have been directly addressed.
Therefore, this statement needs further experimental support and discussion. Ecdysone is a critical hormone during development and especially growth-related effects central to this study. The authors should consider doing pharmacology or augment their claims here with genetic manipulation experiments of 20HE related genes in larvae (Leopold, Rewitz, Rideout, Drummond-Barbosa, Schuldiner labs) and adult animals using genetics, pharmacology or direct assessment of 20HE levels (RIPA, Edgar and Reiff labs).
- In Fig.7 the authors used a sog-LacZ stock to show transcriptional activation in

fatbody cells. This stock is based on P-element insertion in the according regulatory regions and supposed to express lacZ with an nls. I can clearly see lacZ in nuclei in Fig. 7H, whereas this is very hard to see in nuclei in Fig7i in the tumour model. In addition, lacZ is known for its high stability and not the best option. As this finding is vital for central claims of this study, it should be complemented by either qPCR for sog on fat body cells or using another readout by converting one of the two Mimic lines (BL42189/44958) into GFP sensors for sog.
- I have similar problems with Fig.7B-F, as phosphorylated Mad should be translocated to the nucleus. In 7F the authors measure pMad over Dapi, which is the right way but it is hard to see pMad in the nucleaus apart from Fig7B, wheras in D and E, where the authors measure higher levels, I cannot identify clear pMad in nuclei. These images either need to show the Dapi channel or more representative images should be chosen like in Fig.4 with arrows pointing to measured nuclei. Fig.7C something went wrong with the compression of this image.
- The proper function of RNAi stocks targeting genes like sog, mad, etc. is vital for this study as these lines are used throughout the study. Functional evidence of specific knockdown efficiency should be provided or references given in which these stocks were shown to provide functional knockdown on transcript or protein level.
- Fig.S7 discusses appearance of gbb/Bmp7 and Sog/CHRD in human patients. The analysis the authors performed shows a correlation between both factors, but is hampered by the fact that datasets for peripheral tissues of cachexia patients are unavailable. The authors may consider sorting these after tumor entities in which cachexia occurs frequently vs. low occurrence and then check for both genes.
- Fig.5 M-P pMAd is not indicated in the Panels only the legend.
- Please follow FlyBase nomenclature, e.g. dlg1 for discs large 1 and unify in the whole manuscript and figure for all genes.
- For endogenous fusion proteins like Viking-GFP (e.g. vkg::GFP) choose a format to clearly decipher them from transcriptional readout stocks like sog-lacZ.
- The quantifications in most figures are quite small with tiny lettering and XY axis are difficult to read in letter/A4 size.

Minor:

1. Adjust in-figure caption alignments
2. Line 104: add comma RasV12, dlgRNAi
3. Line 114: replace little ◊ not significant (n.s.)
4. Line 334: 'sogRNAi overexpression' to my knowledge, RNAi are expressed, not overexpressed.
5. Line 454: italicize r4>
6. Fig S4E: remove frame
7. Figures 6: It would be better to number and explain the pathway presented in the

figure in text and fig legend.
8. Just a personal preference. Lettering of images in images is commonly done horizontally, here it appears like a mix between vertical and horizontal.

2. Significance:

Significance (Required)

In this manuscript, Bakopolous et al. investigated on the function of Insulin and TGF beta signaling in the converging regulation of sog (BMP antagonist) and how it controls ECM remodeling. Therefore, the authors used a Drosophila model of cachexia established in Lodge et al., 2021. The authors have shown that the tumors increase Impl2 and Gbb in the fatbody leading to the inhibition of insulin signaling and activation of TGF-β signaling respectively. This lead to the accumulation of ECM proteins that contributes to muscle ECM deficit and muscle detachment. These findings are a major advance in the field of cachexia and of broad interest. The authors demonstrate that state-of-the-art genetics in flies allows acquisition of genetically precise data along with important and complex discoveries on signaling pathways with relevance not only for basic, but for biomedical research as well.

The manuscript is concise and very well written. The experiments overt a clear logical order and are comprehensively described. The authors provide exhaustive data to support their novel claims of broad interest to the scientific community

3. How much time do you estimate the authors will need to complete the suggested revisions:

Estimated time to Complete Revisions (Required)

(Decision Recommendation)

Between 3 and 6 months

4. *Review Commons* values the work of reviewers and encourages them to get credit for their work. Select 'Yes' below to register your reviewing activity at [Web of Science Reviewer Recognition Service] (formerly Publons); note that the content of your review will not be visible on Web of Science.

Web of Science Reviewer Recognition

Yes

Review #2

1. Evidence, reproducibility and clarity:

Evidence, reproducibility and clarity (Required)

Summary

In this paper, the authors show how the interaction of two signaling pathways, insulin/PI3K and TGF-β signaling, in the fatbody plays an important role in cachectic muscle detachment in tumor-bearing animals. The Drosophila tumor models and the genetic experimental tools are sophisticated and the conclusion is well supported by the data from these genetic experiments. They found that the TGF-β signaling activation (phosphorylation of Mad) is negatively regulated by insulin/PI3K signaling in the fatbody. They also identified the functional involvements of two molecules secreted from tumour tissues, Impl2 (a negative regulator of insulin signaling) and Gbb (one of the TGF-β ligands), in protein synthesis and ECM accumulation in the fatbody, respectively. They also showed that the cachectic fatbody traps ECM proteins and prevents ECM secretion to the muscle, causing muscle degradation. Finally, they identified a secreted BMP antagonist, Sog, as an important player in this process. They found that Sog is reduced in the hemolymph of tumour-bearing animals and that Sog expression is regulated by insulin signaling. Furthermore, Sog overexpression in the tumours, fatbody, and muscle rescues cachectic muscle detachment.

Major comment

Their genetic experiments clearly showed that the reduction of insulin signaling activity in the fatbody induces upregulation of TGF-β signaling and Collagen accumulation. Then, how does TGF-β signaling induce Collagen accumulation? They showed that Rab10 knockdown and SPARC overexpression reduced the accumulation of fatbody ECM. Are Rab10 and SPARC expression regulated by TGF-β signaling?

Minor comments

1. Line 90: "Disc Large (Dlg) RNAi in the eye" must be "Discs Large (Dlg1) RNAi in the eye imaginal discs".
2. Figures 1D and 1L are from the same image. Also, Figures 1C and 1M are from the same image. Are both of them necessary to be shown in the different panels?
3. Why are the staining patterns of anti-pAkt shown in Figures 1L and 1U so different? pAkt is not detected in the nuclei in Fig. 1L but its nuclear signal is clear in Fig. 1U.
4. Figure 1: Images of counter staining for nuclei like DAPI should be also included for all these fatbody images.
5. Line 101: "Tumour specific ImpL2 inhibition was sufficient to reduce fatbody pAkt levels." Is this correct? ImpL2 inhibition in tumors should elevate the pAKT level in fatbody.
6. Figure S1~S4: These figures and their legends do not correspond to each other.
7. Line 189: The pAkt level in the muscle of tumour-bearing animals should be examined to confirm the activity of the insulin signaling is downregulated.
8. Line 189: If the authors conclude that muscle insulin signaling predominantly regulates translation and atrophy, OPP assay for the muscle cells should be examined in the same experimental settings.
9. Line 247: The expression level of Rab10 and SPARC should be examined in the fatbody of tumour-bearing animals to see whether Rab10 is upregulated and SPARC is downregulated.
10. Line 247: If Rab10 upregulation and SPARC downregulation are the causes of the accumulation of ECM proteins in the fatbody of tumour-bearing animals, how the overexpressed Collagen proteins can be secreted from the fatbody cells?
11. Line 347: Sog is a secreted BMP antagonist. Thus, it can be expected that the Sog overexpression downregulates TGF-β signaling in fatbody and muscle tissues. If the rescued phenotypes with Sog overexpression can be explained by this logic, pMad level should be examined in these experiments.

2. Significance:

Significance (Required)

I found these results from their genetic experiments described here very interesting and of high quality. Although the mechanism by which the TGF-β signaling induces ECM accumulation in fatbody is not clear, this study represents several important advances to understand the key processes in tumor-induced muscle degradation. These data will attract broad audiences not only from cancer biology but also from the research fields including interorgan interactions, systemic signaling in homeostasis, and developmental biology.

3. How much time do you estimate the authors will need to complete the suggested revisions:

Estimated time to Complete Revisions (Required)

(Decision Recommendation)

Between 1 and 3 months

4. *Review Commons* values the work of reviewers and encourages them to get credit for their work. Select 'Yes' below to register your reviewing activity at Web of Science Reviewer Recognition Service (formerly Publons); note that the content of your review will not be visible on Web of Science.

Web of Science Reviewer Recognition

Yes

Review #3

1. Evidence, reproducibility and clarity:

Evidence, reproducibility and clarity (Required)

Summary:

Provide a short summary of the findings and key conclusions (including methodology and model system(s) where appropriate).

This paper uses a Drosophila tumor model induced by the expression of RasV12+Scrib-IR or RasV12+Dlg-IR in the eye imaginal disc to understand how inter-organ communication affects cachexia in the fat body and muscle. The tumor has previously been shown to secrete the factors ImpL2 and Gbb which decreases insulin signalling and increases TGF-beta signalling in the fat body, respectively, and

results in fat body and muscle defects. Here they dissect the role of insulin and TGF-beta signalling in the fat body in regulating muscle integrity further. They show that these two pathways converge via Sog in the fat body of tumor-bearing animals and result in aberrant ECM accumulation in the fat body which hinders ECM secretion. This then results in the muscle receiving less fat body-derived ECM which causes muscle attachment defects. Interestingly, these muscle defects can be ameliorated by activating insulin signalling or inhibiting TGF-beta signalling or even by increasing ECM secretion in the fat body. The authors also provide some evidence that the insulin and TGF-beta signalling pathways can converge in non-tumor settings.

Major comments:

- Are the key conclusions convincing?

Most of the conclusions are convincing. It is not clear however whether the ECM accumulation in the fat body of tumor animals is fibrotic and whether it is extracellular or in the cell cortex.
- Should the authors qualify some of their claims as preliminary or speculative, or remove them altogether?
- The authors state in line 71 'This deposition of disorganized ECM leads to fibrotic ECM
accumulation.' The authors haven't really provided evidence for the ECM being fibrotic. The authors could either rephrase this or provide additional experimental evidence of fibrosis in the fat body.
- Would additional experiments be essential to support the claims of the paper? Request additional experiments only where necessary for the paper as it is, and do not ask authors to open new lines of experimentation.
- The authors state in line 147" Finally, in tumor-bearing animals fed a sterol-free diet, that underwent a prolonged 3rd instar stage due to reduced ecdysone levels (Parkin and Burnet, 1986), we activated insulin signalling in the fatbody via Akt overexpression (QRasV12, scribRNAi). We found that this manipulation caused a significant decrease in pMad levels in the fatbody and a rescue of muscle detachment (Figure S1 D-I), similar to animals fed a standard diet (Figure 1 O-Q, Figure 2 F-H)." Since it's not already known what the extent of muscle integrity defect there is in tumors with additional sterol free diet, it would be important to show a non-tumor control for comparison in FigS1F. This would also then make it clear to what extent the defect is rescued by Akt overexpression.
- The authors state in line 158 'Upon the knockdown of Impl2, we found that tumor gbb was not significantly altered (Figure S3A).' Even though this shows an indication that Gbb levels are not reduced, the n number is too low to state that it is non-

significant. The authors should increase the n number here.
- The authors state in line 171 'Conversely, knockdown of gbb alone or knockdown of gbb together with ImpL2 significantly rescued the Nidogen overaccumulation defects observed at the plasma membrane of fatbody from tumor-bearing animals, while ImpL2RNAi alone did not (Figure S2 Q-U).' This is a somewhat misleading representation, since again no non-tumor control was used, so the extent of the rescue by gbb knowdown is not obvious. In FigS2P Nidogen levels in the tumor seem ~100% higher than in control. But in FigS2U, in which no control was included, the tumor+gbb knowdown seems ~ 20% lower than tumor. So it is probably a more moderate rescue, but that's only possible to assess by including a non-tumor control in FigS2U. Also the images in FigS2Q-T don't seem representative since they appear to show a much bigger difference in fluorescence intensity than ~20%. Please show more representative images.
- The authors state in line 174 'Finally, co-knockdown of gbb and ImpL2 in the tumor significantly rescued the reduction in OPP and Nidogen levels observed in the muscles of tumor-bearing animals (Figure S3 B-I).'
Again, the single knockdowns and the non-tumor control are not shown here in Fig3E and I and should be included for comparison and to see the contribution of each knockdown and to be able to judge the extent of the rescue.
- Regarding Fig3O: Is there a significant tumor muscle attachment defect here? In this graph the tumor only looks about 10% lower than the WT (rather than 40% in Fig2E). The other issue is the extremely low n number for WT. I would recommend increasing the n number for WT here and to indicate in the graph whether the tumor is significantly different to WT (or non-significant, in which case RabRNAi wouldn't actually 'rescue' the defect). In the present form, this graph is not very convincing.
- Regarding Fig3W: A non-tumor control would be important to include to be able to judge the extent of muscle attachment defects and the extent of the rescue for UAS-Sparc. This will allow to assess the severity of muscle integrity defect in this particular experiment (since it appears to vary in different experiments e.g. muscle defect in tumor 40% in Fig2E and ~10% in Fig3O) and to assess the extent of rescue for the various genotypes.
- The authors show an accumulation of ECM in the fat body of tumors. It is not clear, whether this ECM accumulates intracellularly near the cell surface or extracellularly. The authors should assess this, maybe by doing electron microscopy.
- Are the suggested experiments realistic in terms of time and resources? It would help if you could add an estimated cost and time investment for substantial experiments.
- These suggested experiments should be quite straightforward since they are mostly just repeating previous experiments with the appropriate controls and n numbers. I would think that they can be done within a few months. The electron microscopy should not take more than a few weeks and not be costly.
- Are the data and the methods presented in such a way that they can be reproduced?

- The details on how old animals used in each experiment were, are not easy to find and not written very clearly. They should be included in the each figure legend rather than summarising those details in the methods.
- Also, in line 788 in the methods, several stocks are indicated as coming from particular labs (e.g. UAS-FOXO (Kieran Harvey), UAS-GFP (Kieran Harvey), UAS-lacZRNAi (Kieran Harvey), UAS-RasV12 (Helena Richardson), UAS-cg25C;UAS-Vkg (Brian Stramer)).
However, it is not clear whether these labs actually made these stocks and if so whether it has already been described in their papers how the lines were made. If the lines are unpublished, the detailed information should be given on how the lines were made. Or if the lines are published, the authors should provide the reference.
- Are the experiments adequately replicated and statistical analysis adequate?
In general, the n number is rather low in several experiments, especially n of 3 for many controls. And as I mentioned before, rescues of tumor phenotypes are often shown without including a non-tumor control, making it hard to judge the extent of the rescue. Sometimes this information can be found in other figures, but the reader should not have to search for it. And also the severity of the phenotype can vary from experiment to experiment.

Minor comments:

Specific experimental issues that are easily addressable.

- Are prior studies referenced appropriately?

Yes, as far as I can tell.
- Are the text and figures clear and accurate?
- In the literature, people usually call it 'fat body' rather than 'fatbody'.
- The authors state in line 265 "Vkg accumulated in the membranes of fatbody where p60 was overexpressed using r4-GAL4 (Figure 5 A-C)."
This must be a typo. I think it is shown in Fig5E-G. Unless it's labelled wrongly in the figure and B, C and D show p60 rather than TorDN.
- The authors state in line 188 'This manipulation significantly rescued muscle integrity (Figure S4 A-C) and muscle atrophy (Figure S4 D-F), without affecting muscle ECM levels (Figure S4 G-H).' According to the graph in FigS4H this does actually 'affect muscle ECM levels' significantly, as in that it reduced Nidogen levels further. The authors could rephrase this.
- Do you have suggestions that would help the authors improve the presentation of their data and conclusions?

2. Significance:

Significance (Required)

- Describe the nature and significance of the advance (e.g. conceptual, technical, clinical) for the field.

The field of inter-organ communication in cancer is a very interesting and trending research field. Several labs including this one have provided new insights into how the tumor, the fat body and the muscle communicate and affect each other and how this can cause cachexia. Previous work from the Chen lab already showed that the tumor secretes the factors ImpL2 and Gbb which decreases insulin signalling and increases TGF-beta signalling in the fat body, respectively and results in fat body and muscle defects. Here they dissect this role of insulin and TGF-beta signalling in the fat body in regulating muscle integrity during cachexia further. They show that these two pathways converge via Sog in the fat body of tumor-bearing animals and result in aberrant ECM accumulation in the fat body which hinders ECM secretion. As a result of this, the muscle receives less fat body-derived ECM and displays muscle attachment defects. Interestingly, the authors show that these muscle defects can be ameliorated by activating insulin signalling or inhibiting TGF-beta signalling or even by increasing ECM secretion in the fat body. This has potentially important implications for the clinic since it suggests that targeting ECM secretion or ECM remodeling in the fat tissue could be a promising treatment for cachexia. Moreover, the authors also provide some evidence that the insulin and TGF-beta signalling pathways can converge in tumor and non-tumor settings. This might also reveal new drug targets to treat cachexia.

- Place the work in the context of the existing literature (provide references, where appropriate).

The Chen lab showed previously that MMP1 secreted from the tumor induces ECM disruption in the fat body as well as muscle, ultimately causing fat body remodeling and muscle wasting (Lodge et al. 2021). They showed that this is via TGF-beta activation in the fat body. Another contributing factor is tumor-secreted Impl2 which decreases Insulin signalling in the fat body and tumor. However, it remained unknown, how ECM accumulation in the fat body might cause muscle wasting. In this paper, the authors look into this.

- State what audience might be interested in and influenced by the reported findings.

This paper would be of interest for scientists and clinicians interested in inter-organ communication in cancer, particularly in the context of cachexia.

- Define your field of expertise with a few keywords to help the authors contextualize your point of view. Indicate if there are any parts of the paper that you do not have sufficient expertise to evaluate.

My expertise lies in the field of Drosophila fat body and ECM, and to some extent tumors but less so signalling pathways.

3. How much time do you estimate the authors will need to complete the suggested revisions:

Estimated time to Complete Revisions (Required)

(Decision Recommendation)

Between 1 and 3 months

4. *Review Commons* values the work of reviewers and encourages them to get credit for their work. Select 'Yes' below to register your reviewing activity at Web of Science Reviewer Recognition Service (formerly Publons); note that the content of your review will not be visible on Web of Science.

Web of Science Reviewer Recognition

Yes

Revision Plan

Manuscript number: RC-2023-01974
Corresponding author(s): Louise Cheng

[The "revision plan" should delineate the revisions that authors intend to carry out in response to the points raised by the referees. It also provides the authors with the opportunity to explain their view of the paper and of the referee reports.

The document is important for the editors of affiliate journals when they make a first decision on the transferred manuscript. It will also be useful to readers of the reprint and help them to obtain a balanced view of the paper.

If you wish to submit a full revision, please use our "Full Revision" template. ***It is important to use the appropriate template to clearly inform the editors of your intentions.****]*

1. General Statements [optional]

This section is optional. Insert here any general statements you wish to make about the goal of the study or about the reviews.

2. Description of the planned revisions

Insert here a point-by-point reply that explains what revisions, additional experimentations and analyses are planned to address the points raised by the referees.

Reviewer 1:

Major:

- The statement (line 149'Together, our data suggest that systemic ecdysone levels are unlikely to be involved in modulating tumour-induced muscle detachment or to mediate the role of fatbody Insulin signalling in regulating muscle detachment.') is derived from an experiment with sterol free diet (in which 20HE is genetically addressed) and a pleiotropic experiment (PG>RasG12V). In neither paper nor the current manuscript, 20HE levels have been directly addressed.

Therefore, this statement needs further experimental support and discussion. Ecdysone is a critical hormone during development and especially growth-related effects central to this study. The authors should consider doing pharmacology or augment their claims here with genetic manipulation experiments of 20HE related genes in larvae (Leopold, Rewitz, Rideout, Drummond-Barbosa, Schuldiner labs) and adult animals using genetics, pharmacology or direct assessment of 20HE levels (RIPA, Edgar and Reiff labs).

The main point we were trying to convey is that we do not think global ecdysone levels plays a role in modulating fatbody insulin or tgfb signalling, which in turn affects muscle detachment. We are not claiming that edysone levels is not changing in control vs. tumour bearing animals. In fact, we predict that 20HE levels will be different in tumour bearing vs. control animals (as

tumour bearing animals undergo developmental delay), but this is not the main point of our conclusions. We believe that our conclusions are supported by the experiment demonstrating global ecdysone alterations (via feeding sterol-free food) did not affect how fatbody Akt activation altered tgfb signalling and enhanced muscle integrity (Figure S1). Therefore, we don't think measuring 20HE helps to support our conclusions. Pharmacological inhibition via feeding ecdysone inhibitors effectively demonstrate a similar point to feeding sterol-free food which we have already performed. We are happy to try direct manipulation of 20HE related genes (eip75B-RNAi) in the fatbody to see if this affects muscle detachment or pAkt and pMad levels in tumour bearing animals.

- In Fig.7 the authors used a sog-LacZ stock to show transcriptional activation in fatbody cells. This stock is based on P-element insertion in the according regulatory regions and supposed to express lacZ with an nls. I can clearly see lacZ in nuclei in Fig. 7H, whereas this is very hard to see in nuclei in Fig7i in the tumour model. In addition, lacZ is known for its high stability and not the best option. As this finding is vital for central claims of this study, it should be complemented by either qPCR for sog on fat body cells or using another readout by converting one of the two Mimic lines (BL42189/44958) into GFP sensors for sog.
We will add a counterstain to these images. We will also perform qPCR in the fatbody of control and cachectic animals to assess whether Sog transcription is altered. We agree converting one of the Mimic lines to a GFP sensor would be a good option, but this experiment would require getting new fly lines into Australia, which takes at least 2 months because of quarantine laws. We don't believe this experiment would change the general conclusions of the paper, therefore would prefer not to do this experiment.

- I have similar problems with Fig.7B-F, as phosphorylated Mad should be translocated to the nucleus. In 7F the authors measure pMad over Dapi, which is the right way but it is hard to see pMad in the nucleaus apart from Fig7B, wheras in D and E, where the authors measure higher levels, I cannot identify clear pMad in nuclei. These images either need to show the Dapi channel or more representative images should be chosen like in Fig.4 with arrows pointing to measured nuclei. Fig.7C something went wrong with the compression of this image.
We will show more representative examples and fix Fig 7C.

- The proper function of RNAi stocks targeting genes like sog, mad, etc. is vital for this study as these lines are used throughout the study. Functional evidence of specific knockdown efficiency should be provided or references given in which these stocks were shown to provide functional knockdown on transcript or protein level.
We agree with the reviewer that this is an important point. We will demonstrate the knockdown of sog and mad (and other RNAis) used in the study by either referring to published data or demonstrate knockdown ourselves.

- Fig.S7 discusses appearance of gbb/Bmp7 and Sog/CHRD in human patients. The analysis the authors performed shows a correlation between both factors, but is hampered by the fact that datasets for peripheral tissues of cachexia patients are unavailable. The authors may

Revision Plan

consider sorting these after tumor entities in which cachexia occurs frequently vs. low occurrence and then check for both genes.
We will try this analysis.

Fig.5 M-P pMAd is not indicated in the Panels only the legend.
We will fix this error.

- Please follow FlyBase nomenclature, e.g. dlg1 for discs large 1 and unify in the whole manuscript and figure for all genes.
We will fix this error.

- For endogenous fusion proteins like Viking-GFP (e.g. vkg::GFP) choose a format to clearly decipher them from transcriptional readout stocks like sog-lacZ.
We will fix this error.

- The quantifications in most figures are quite small with tiny lettering and XY axis are difficult to read in letter/A4 size.
We will enlarge font size.

Minor:
1. Adjust in-figure caption alignments
2. Line 104: add comma RasV12, dlgRNAi
3. Line 114: replace little □ not significant (n.s.)
4. Line 334: 'sogRNAi overexpression' to my knowledge, RNAi are expressed, not overexpressed.
5. Line 454: italicize r4>
6. Fig S4E: remove frame
7. Figures 6: It would be better to number and explain the pathway presented in the figure in text and fig legend.
8. Just a personal preference. Lettering of images in images is commonly done horizontally, here it appears like a mix between vertical and horizontal.
We will fix these minor errors.

Reviewer 2:
Major comment
Their genetic experiments clearly showed that the reduction of insulin signaling activity in the fatbody induces upregulation of TGF-β signaling and Collagen accumulation. Then, how does TGF-β signaling induce Collagen accumulation?
From the experiments we have carried out, we do not have insights into how TGF-B signalling induce Collagen accumulation.
They showed that Rab10 knockdown and SPARC overexpression reduced the accumulation of fatbody ECM. Are Rab10 and SPARC expression regulated by TGF-β signaling?

Revision Plan

We can address this point by assessing if Rab10 and SPARC expression is altered in cachectic fatbody.

Minor comments

1. Line 90: "Disc Large (Dlg) RNAi in the eye" must be "Discs Large (Dlg1) RNAi in the eye imaginal discs". *We will fix this error*

2. Figures 1D and 1L are from the same image. Also, Figures 1C and 1M are from the same image. Are both of them necessary to be shown in the different panels?
The duplication of 1C and 1M, was an error, we thank the reviewer for picking this up. We will fix this error. We will use different images for 1D and 1L.

3. Why are the staining patterns of anti-pAkt shown in Figures 1L and 1U so different? pAkt is not detected in the nuclei in Fig. 1L but its nuclear signal is clear in Fig. 1U.
We will show more representative images of these staining.
4. Figure 1: Images of counter staining for nuclei like DAPI should be also included for all these fatbody images. *We will show counter staining for DAPI.*
5. Line 101: "Tumour specific ImpL2 inhibition was sufficient to reduce fatbody pAkt levels." Is this correct? ImpL2 inhibition in tumors should elevate the pAKT level in fatbody. *This was a mistake, we will fix this error.*
6. Figure S1~S4: These figures and their legends do not correspond to each other. *We thank the reviewer in picking up this error, there was an error in inserting the images into the text. S2 and S3 were swapped. We will fix this error.*
7. Line 189: The pAkt level in the muscle of tumour-bearing animals should be examined to confirm the activity of the insulin signaling is downregulated.
We will include this data.
8. Line 189: If the authors conclude that muscle insulin signaling predominantly regulates translation and atrophy, OPP assay for the muscle cells should be examined in the same experimental settings.
We will carry out OPP assay upon Akt overexpression in the muscle.
9. Line 247: The expression level of Rab10 and SPARC should be examined in the fatbody of tumour-bearing animals to see whether Rab10 is upregulated and SPARC is downregulated.
10. Line 247: If Rab10 upregulation and SPARC downregulation are the causes of the accumulation of ECM proteins in the fatbody of tumour-bearing animals, how the overexpressed Collagen proteins can be secreted from the fatbody cells? *We are not sure, but the overexpression of Collagen proteins is at an extremely high level, therefore, it is possible that some of it can be processed and secreted despite Rab10 upregulation and SPARC downregulation. We have carried out an experiment to overexpress Collagen proteins in the muscle, in this case, this manipulation did not rescue. This indicates that processing of Collagen in the fatbody is important, however, we do not know how the processing is regulated.*
11. Line 347: Sog is a secreted BMP antagonist. Thus, it can be expected that the Sog overexpression downregulates TGF-β signaling in fatbody and muscle tissues. If the rescued

phenotypes with Sog overexpression can be explained by this logic, pMad level should be examined in these experiments.
We have shown this data in Figure R-T. We will refer back to this data in Line 347.

Reviewer 3:
Major comments:
- Are the key conclusions convincing?
Most of the conclusions are convincing. It is not clear however whether the ECM accumulation in the fat body of tumor animals is fibrotic and whether it is extracellular or in the cell cortex.

- Should the authors qualify some of their claims as preliminary or speculative, or remove them altogether?
-The authors state in line 71 'This deposition of disorganized ECM leads to fibrotic ECM accumulation.' The authors haven't really provided evidence for the ECM being fibrotic. The authors could either rephrase this or provide additional experimental evidence of fibrosis in the fat body.
We will tone down the claim that the ECM accumulation is fibrotic.

- Would additional experiments be essential to support the claims of the paper? Request additional experiments only where necessary for the paper as it is, and do not ask authors to open new lines of experimentation.
-The authors state in line 147" Finally, in tumor-bearing animals fed a sterol-free diet, that underwent a prolonged 3rd instar stage due to reduced ecdysone levels (Parkin and Burnet, 1986), we activated insulin signalling in the fatbody via Akt overexpression (QRasV12, scribRNAi). We found that this manipulation caused a significant decrease in pMad levels in the fatbody and a rescue of muscle detachment (Figure S1 D-I), similar to animals fed a standard diet (Figure 1 O-Q, Figure 2 F-H)." Since it's not already known what the extent of muscle integrity defect there is in tumors with additional sterol free diet, it would be important to show a non-tumor control for comparison in FigS1F. This would also then make it clear to what extent the defect is rescued by Akt overexpression.
We will include a non-tumour control for Fig S1F.

-The authors state in line 158 'Upon the knockdown of Impl2, we found that tumor gbb was not significantly altered (Figure S3A).' Even though this shows an indication that Gbb levels are not reduced, the n number is too low to state that it is non-significant. The authors should increase the n number here.
N=3 is generally enough to see a difference, we will include data done in parallel which shows Impl2 RNAi is sufficient to induce a reduction in Impl2 RNA levels. This will demonstrate that n=3 is sufficient to demonstrate a reduction in transcript levels if there is a reduction.

-The authors state in line 171 'Conversely, knockdown of gbb alone or knockdown of gbb together with ImpL2 significantly rescued the Nidogen overaccumulation defects observed at the

plasma membrane of fatbody from tumor-bearing animals, while ImpL2RNAi alone did not (Figure S2 Q-U).' This is a somewhat misleading representation, since again no non-tumor control was used, so the extent of the rescue by gbb knowdown is not obvious. In FigS2P Nidogen levels in the tumor seem ~100% higher than in control. But in FigS2U, in which no control was included, the tumor+gbb knowdown seems ~ 20% lower than tumor. So it is probably a more moderate rescue, but that's only possible to assess by including a non-tumor control in FigS2U. Also the images in FigS2Q-T don't seem representative since they appear to show a much bigger difference in fluorescence intensity than ~20%. Please show more representative images.

We will include a non-tumour control for S2Q-T and show more representative pictures.

-The authors state in line 174 'Finally, co-knockdown of gbb and ImpL2 in the tumor significantly rescued the reduction in OPP and Nidogen levels observed in the muscles of tumor-bearing animals (Figure S3 B-I).'

*Again, the single knockdowns and the non-tumor control are not shown in Fig**S**3E and I and should be included for comparison and to see the contribution of each knockdown and to be able to judge the extent of the rescue.*

We will include the single knockdowns and a wildtype control

-Regarding Fig3O: Is there a significant tumor muscle attachment defect here? In this graph the tumor only looks about 10% lower than the WT (rather than 40% in Fig2E). The other issue is the extremely low n number for WT. I would recommend increasing the n number for WT here and to indicate in the graph whether the tumor is significantly different to WT (or non-significant, in which case RabRNAi wouldn't actually 'rescue' the defect). In the present form, this graph is not very convincing.

We will increase the n number for WT for this experiment. The reduction in muscle detachment is 10% rather than 40% here is because this experiment was done at day 6, which we will indicate in the figure legend. The 40% reduction in Fig2E is because these samples were processed at day7. Rab10RNAi experiment was carried out at day 6, because by day7, the Rab10RNAi rescue is so good, most of the tumour bearing animals have pupated, thus the experiment could only be carried out at day6.

- Regarding Fig3W: A non-tumor control would be important to include to be able to judge the extent of muscle attachment defects and the extent of the rescue for UAS-Sparc. This will allow to assess the severity of muscle integrity defect in this particular experiment (since it appears to vary in different experiments e.g. muscle defect in tumor 40% in Fig2E and ~10% in Fig3O) and to assess the extent of rescue for the various genotypes.

We will include a non-tumour control for 3W.

-The authors show an accumulation of ECM in the fat body of tumors. It is not clear, whether this ECM accumulates intracellularly near the cell surface or extracellularly. The authors should assess this, maybe by doing electron microscopy.

Revision Plan

We do not have an EM facility that can accommodate this experiment, thus doing EM is not an option for us. However, we can address whether the accumulation of ECM is intracellular or extracellular by performing an experiment, where we try perform antibody staining against Viking-GFP without permeabilizing the cells. If Viking is detected without permeabilization, it would indicate the accumulations are extracellular. This approach has been previously used to address this question in Zang et al., elife, 2015.

- Are the suggested experiments realistic in terms of time and resources? It would help if you could add an estimated cost and time investment for substantial experiments.
-These suggested experiments should be quite straightforward since they are mostly just repeating previous experiments with the appropriate controls and n numbers. I would think that they can be done within a few months. The electron microscopy should not take more than a few weeks and not be costly.

- Are the data and the methods presented in such a way that they can be reproduced?
-The details on how old animals used in each experiment were, are not easy to find and not written very clearly. They should be included in the each figure legend rather than summarising those details in the methods.
We will add the number of days in the figure legend.

-Also, in line 788 in the methods, several stocks are indicated as coming from particular labs (e.g. UAS-FOXO (Kieran Harvey), UAS-GFP (Kieran Harvey), UAS-lacZRNAi (Kieran Harvey), UAS-RasV12 (Helena Richardson), UAS-cg25C;UAS-Vkg (Brian Stramer)).
However, it is not clear whether these labs actually made these stocks and if so whether it has already been described in their papers how the lines were made. If the lines are unpublished, the detailed information should be given on how the lines were made. Or if the lines are published, the authors should provide the reference.
We will fix these references.

- Are the experiments adequately replicated and statistical analysis adequate?
In general, the n number is rather low in several experiments, especially n of 3 for many controls. And as I mentioned before, rescues of tumor phenotypes are often shown without including a non-tumor control, making it hard to judge the extent of the rescue. Sometimes this information can be found in other figures, but the reader should not have to search for it. And also the severity of the phenotype can vary from experiment to experiment.
We will include a non-tumour control when appropriate to address this.

Minor comments:
- Specific experimental issues that are easily addressable.
- Are prior studies referenced appropriately?
Yes, as far as I can tell.

Revision Plan

- Are the text and figures clear and accurate?
-In the literature, people usually call it 'fat body' rather than 'fatbody'.
We will fix this error.

-The authors state in line 265 "Vkg accumulated in the membranes of fatbody where p60 was overexpressed using r4-GAL4 (Figure 5 A-C)."
This must be a typo. I think it is shown in Fig5E-G. Unless it's labelled wrongly in the figure and B, C and D show p60 rather than TorDN.
We will fix this error.

-The authors state in line 188 'This manipulation significantly rescued muscle integrity (Figure S4 A-C) and muscle atrophy (Figure S4 D-F), without affecting muscle ECM levels (Figure S4 G-H).' According to the graph in FigS4H this does actually 'affect muscle ECM levels' significantly, as in that it reduced Nidogen levels further. The authors could rephrase this.
We will reword this statement.

3. Description of the revisions that have already been incorporated in the transferred manuscript

Please insert a point-by-point reply describing the revisions that were already carried out and included in the transferred manuscript. If no revisions have been carried out yet, please leave this section empty.

4. Description of analyses that authors prefer not to carry out

Please include a point-by-point response explaining why some of the requested data or additional analyses might not be necessary or cannot be provided within the scope of a revision. This can be due to time or resource limitations or in case of disagreement about the necessity of such additional data given the scope of the study. Please leave empty if not applicable.

Reviewer 1:
In Fig.7 the authors used a sog-LacZ stock to show transcriptional activation in fatbody cells. This stock is based on P-element insertion in the according regulatory regions and supposed to express lacZ with an nls. I can clearly see lacZ in nuclei in Fig. 7H, whereas this is very hard to see in nuclei in Fig7i in the tumour model. In addition, lacZ is known for its high stability and not the best option. As this finding is vital for central claims of this study, it should be complemented by either qPCR for sog on fat body cells or using another readout by converting one of the two Mimic lines (BL42189/44958) into GFP sensors for sog.
We will add a counterstain to these images. We will also perform qPCR in the fatbody of control and cachectic animals to assess whether Sog transcription is altered. We agree converting one of the Mimic lines to a GFP sensor would be a good option, but this experiment would require

Revision Plan

getting new fly lines into Australia, which takes at least 2 months because of quarantine laws. We don't believe this experiment would change the general conclusions of the paper, therefore would prefer not to do this experiment.

Dear Dr. Cheng,

Thank you for transferring your manuscript to EMBO Reports, which was previously reviewed at Review Commons. Referees express interest in the proposed regulation of cachexia by insulin and TGFbeta signaling pathways in the fat body of tumor bearing flies. However, they also raise concerns that need to be addressed to consider publication here.

Having looked at all files, we would like to invite you to submit a revised manuscript as in your revision plan. Please revise your manuscript with the understanding that the referee concerns (as in their reports) must be fully addressed and their suggestions taken on board. Please address all referee concerns in a complete point-by-point response. Acceptance of the manuscript will depend on a positive outcome of a second round of review. It is EMBO reports policy to allow a single round of major experimental revision only and acceptance or rejection of the manuscript will therefore depend on the completeness of your responses included in the next, final version of the manuscript.

We realize that it is difficult to revise to a specific deadline. In the interest of protecting the conceptual advance provided by the work, we recommend a revision within 3 months. Please discuss the revision progress ahead of this time with me if you require more time to complete the revisions, or if you have questions or comments regarding the revision (also by video chat).

IMPORTANT NOTE: we perform an initial quality control of all revised manuscripts before re-review. Your manuscript will FAIL this control and the handling will be DELAYED if the following APPLIES:
1. A data availability section providing access to data deposited in public databases is missing (where applicable).
2. Your manuscript contains statistics and error bars based on n=2. Please use scatter plots in these cases.

You can submit the revision either as a Scientific Report or as a Research Article. For Scientific Reports, the revised manuscript can contain up to 5 main figures and 5 Expanded View figures, and it should not exceed 27000 characters. If the revision leads to a manuscript with more than 5 main figures it will be published as a Research Article. In this case the Results and Discussion section should be separate. If a Scientific Report is submitted, these sections have to be combined. This will help to shorten the manuscript text by eliminating some redundancy that is inevitable when discussing the same experiments twice. In either case, all materials and methods should be included in the main manuscript file.

When submitting your revised manuscript, please carefully review the instructions that follow below. Failure to include requested items will delay the evaluation of your revision.

1) a .docx formatted version of the manuscript text (including legends for main figures, EV figures and tables). Please make sure that the changes are highlighted to be clearly visible.

2) individual production quality figure files as .eps, .tif, .jpg (one file per figure). See https://wol-prod-cdn.literatumonline.com/pb-assets/embo-site/EMBOPress_Figure_Guidelines_061115-1561436025777.pdf for more info on how to prepare your figures.

3) We replaced Supplementary Information with Expanded View (EV) Figures and Tables that are collapsible/expandable online. A maximum of 5 EV Figures can be typeset. EV Figures should be cited as 'Figure EV1, Figure EV2" etc... in the text and their respective legends should be included in the main text after the legends of regular figures.

- For the figures that you do NOT wish to display as Expanded View figures, they should be bundled together with their legends in a single PDF file called *Appendix*, which should start with a short Table of Content. Appendix figures should be referred to in the main text as: "Appendix Figure S1, Appendix Figure S2" etc. See detailed instructions regarding expanded view here:

- Additional Tables/Datasets should be labeled and referred to as Table EV1, Dataset EV1, etc. Legends have to be provided in a separate tab in case of .xls files. Alternatively, the legend can be supplied as a separate text file (README) and zipped together with the Table/Dataset file.

4) a .docx formatted letter INCLUDING the reviewers' reports and your detailed point-by-point responses to their comments. As part of the EMBO publication's Transparent Editorial Process, EMBO reports publishes online a Review Process File (RPF) to accompany accepted manuscripts. This File will be published in conjunction with your paper and will include the referee reports, your point-by-point response and all pertinent correspondence relating to the manuscript.
https://www.embopress.org/page/journal/14693178/authorguide#transparentprocess
You are able to opt out of this by letting the editorial office know (emboreports@embo.org). If you do opt out, the Review Process File link will point to the following statement: "No Review Process File is available with this article, as the authors have chosen not to make the review process public in this case."

5) a complete author checklist, which you can download from our author guidelines

https://www.embopress.org/page/journal/14693178/authorguide. Please insert information in the checklist that is also reflected in the manuscript. The completed author checklist will also be part of the RPF.

6) Please note that all corresponding authors are required to supply an ORCID ID for their name upon submission of a revised manuscript (). Please find instructions on how to link your ORCID ID to your account in our manuscript tracking system in our Author guidelines

7) Before submitting your revision, primary datasets produced in this study need to be deposited in an appropriate public database (see https://www.embopress.org/page/journal/14693178/authorguide#datadeposition). Please remember to provide a reviewer password if the datasets are not yet public. The accession numbers and database should be listed in a formal "Data Availability" section placed after Materials & Method (see also https://www.embopress.org/page/journal/14693178/authorguide#datadeposition). Please note that the Data Availability Section is restricted to new primary data that are part of this study. * Note - All links should resolve to a page where the data can be accessed. *
If your study has not produced novel datasets, please mention this fact in the Data Availability Section.

8) At EMBO Press we ask authors to provide source data for the main figures. Our source data coordinator will contact you to discuss which figure panels we would need source data for and will also provide you with helpful tips on how to upload and organize the files.

Additional information on source data and instruction on how to label the files are available:
https://www.embopress.org/page/journal/14693178/authorguide#sourcedata

9) Our journal encourages inclusion of *data citations in the reference list* to directly cite datasets that were re-used and obtained from public databases. Data citations in the article text are distinct from normal bibliographical citations and should directly link to the database records from which the data can be accessed. In the main text, data citations are formatted as follows: "Data ref: Smith et al, 2001" or "Data ref: NCBI Sequence Read Archive PRJNA342805, 2017". In the Reference list, data citations must be labeled with "[DATASET]". A data reference must provide the database name, accession number/identifiers and a resolvable link to the landing page from which the data can be accessed at the end of the reference. Further instructions are available at http://www.embopress.org/page/journal/14693178/authorguide#referencesformat

10) Regarding data quantification (see Figure Legends:
https://www.embopress.org/page/journal/14693178/authorguide#figureformat)

The following points must be specified in each figure legend:

- the name of the statistical test used to generate error bars and P values,

- the number (n) of independent experiments (please specify technical or biological replicates) underlying each data point,

- the nature of the bars and error bars (s.d., s.e.m.),

- If the data are obtained from n Program fragment delivered error ``Can't locate object method "less" via package "than" (perhaps you forgot to load "than"?) at //ejpvfs23/sites23b/embor_www/letters/embor_decision_revise_and_review.txt line 56.' 2, use scatter blots showing the individual data points.

Discussion of statistical methodology can be reported in the materials and methods section, but figure legends should contain a basic description of n, P and the test applied.

- Please also include scale bars in all microscopy images.

11) The journal requires a statement specifying whether or not authors have competing interests (defined as all potential or actual interests that could be perceived to influence the presentation or interpretation of an article). In case of competing interests, this must be specified in your disclosure statement. Further information: https://www.embopress.org/competing-interests

12) Please also note our reference format:
http://www.embopress.org/page/journal/14693178/authorguide#referencesformat

We would also welcome the submission of cover suggestions, or motifs to be used by our Graphics Illustrator in designing a cover.

I look forward to seeing a revised version of your manuscript when it is ready. Please let me know if you have questions or comments regarding the revision.

Kind regards,

Deniz Senyilmaz Tiebe

Deniz Senyilmaz Tiebe, PhD
Editor
EMBO Reports

Manuscript number: RC-2023-01974/EMBOR-2023-57695
Corresponding author(s): Louise Cheng

[The "revision plan" should delineate the revisions that authors intend to carry out in response to the points raised by the referees. It also provides the authors with the opportunity to explain their view of the paper and of the referee reports.

The document is important for the editors of affiliate journals when they make a first decision on the transferred manuscript. It will also be useful to readers of the reprint and help them to obtain a balanced view of the paper.

If you wish to submit a full revision, please use our "Full Revision" template. ***It is important to use the appropriate template to clearly inform the editors of your intentions.****]*

1. General Statements [optional]

This section is optional. Insert here any general statements you wish to make about the goal of the study or about the reviews.

2. Description of the planned revisions

Insert here a point-by-point reply that explains what revisions, additional experimentations and analyses are planned to address the points raised by the referees.

Reviewer 1:

Major:

- The statement (line 149'Together, our data suggest that systemic ecdysone levels are unlikely to be involved in modulating tumour-induced muscle detachment or to mediate the role of fatbody Insulin signalling in regulating muscle detachment.') is derived from an experiment with sterol free diet (in which 20HE is genetically addressed) and a pleiotropic experiment (PG>RasG12V). In neither paper nor the current manuscript, 20HE levels have been directly addressed.

Therefore, this statement needs further experimental support and discussion. Ecdysone is a critical hormone during development and especially growth-related effects central to this study. The authors should consider doing pharmacology or augment their claims here with genetic manipulation experiments of 20HE related genes in larvae (Leopold, Rewitz, Rideout, Drummond-Barbosa, Schuldiner labs) and adult animals using genetics, pharmacology or direct assessment of 20HE levels (RIPA, Edgar and Reiff labs).

The main point we were trying to convey is that we do not think global ecdysone levels plays a role in modulating fatbody insulin or tgfb signalling, which in turn affects muscle detachment. We are not claiming that edysone levels is not changing in the fat body of control vs. tumour bearing animals. In fact, we predict that 20HE levels will be different in tumour bearing vs. control

animals (as tumour bearing animals undergo developmental delay). We modified our conclusions: (line 150-151) which now says: alterations in global ecdysone signalling does not significantly alter the Akt and TGF-β signalling in the fat body of tumour bearing animals. We believe that our conclusions are supported by the experiment demonstrating global ecdysone alterations (via feeding sterol-free food) did not affect how fatbody Akt activation altered tgfb signalling and enhanced muscle integrity (Figure Appendix S1).
Pharmacological inhibition via feeding ecdysone inhibitors effectively demonstrate a similar point to feeding sterol-free food which we have already performed.
We have tried direct manipulation of 20HE related genes (eip75B-RNAi) in the fatbody (using a previously validated RNAi, Hoedjes et al., 2021). However, this did not significantly affect pAkt and pMad levels in tumour bearing animals, nor did this affect muscle detachment. We have included the data as a reviewer's only figure 1 A-C.

- In Fig.7 the authors used a sog-LacZ stock to show transcriptional activation in fatbody cells. This stock is based on P-element insertion in the according regulatory regions and supposed to express lacZ with an nls. I can clearly see lacZ in nuclei in Fig. 7H, whereas this is very hard to see in nuclei in Fig7i in the tumour model. In addition, lacZ is known for its high stability and not the best option. As this finding is vital for central claims of this study, it should be complemented by either qPCR for sog on fat body cells or using another readout by converting one of the two Mimic lines (BL42189/44958) into GFP sensors for sog.
To assess whether Sog levels are altered in the fatbody, we have performed proteomics and qPCR in the fatbody of WT and tumour bearing animals. We found that contrary to our sog-lacZ data, both transcription and protein readouts of Sog appears to be elevated in the tumour fat body, rather than downregulated as we initially showed with sog-lacZ. We have withdrawn the sog-lacZ data and have included the proteomics and qPCR data in reviewer's only Figure 1 E.
We had to re-interpret our model of the events in the fat body. In the wildtype fatbody, the link via Sog is still valid, where activation of insulin signalling inhibits Sog, and this in turn activates TGFB. In the tumour bearing animals, we think what is important is that circulating Sog levels are low, this can cause an upregulation of TGF-β in the fatbody. We have performed additional experiments to assess how circulating Sog levels is regulated. We found that this is mainly regulated by tumour derived Mmp1. Its inhibition via Timp brings Sog levels back to wildtype levels. Therefore, in addition to the role of Mmp1 in directly regulating Gbb levels, it also regulates haemolymph Sog levels, we have included this new data in Figure 8. Genetically, overexpression of Sog in the fatbody, muscle or tumour can improve tumour-induced cachexia.

- I have similar problems with Fig.7B-F, as phosphorylated Mad should be translocated to the nucleus. In 7F the authors measure pMad over Dapi, which is the right way but it is hard to see pMad in the nucleaus apart from Fig7B, wheras in D and E, where the authors measure higher levels, I cannot identify clear pMad in nuclei. These images either need to show the Dapi channel or more representative images should be chosen like in Fig.4 with arrows pointing to measured nuclei. Fig.7C something went wrong with the compression of this image.
We have shown more representative examples and fixed Fig 7C.

- The proper function of RNAi stocks targeting genes like sog, mad, etc. is vital for this study as these lines are used throughout the study. Functional evidence of specific knockdown efficiency should be provided or references given in which these stocks were shown to provide functional knockdown on transcript or protein level.
We have demonstrated the knockdown efficiency of sogRNAi and madRNAi in Figure EV5.

- Fig.S7 discusses appearance of gbb/Bmp7 and Sog/CHRD in human patients. The analysis the authors performed shows a correlation between both factors, but is hampered by the fact that datasets for peripheral tissues of cachexia patients are unavailable. The authors may consider sorting these after tumor entities in which cachexia occurs frequently vs. low occurrence and then check for both genes.
We have added this analysis and this is now the new Appendix Figure 3.

Fig.5 M-P pMAd is not indicated in the Panels only the legend.
We have fixed this.

- Please follow FlyBase nomenclature, e.g. dlg1 for discs large 1 and unify in the whole manuscript and figure for all genes.
We have fixed this error.

- For endogenous fusion proteins like Viking-GFP (e.g. vkg::GFP) choose a format to clearly decipher them from transcriptional readout stocks like sog-lacZ.
We have fixed this error.

- The quantifications in most figures are quite small with tiny lettering and XY axis are difficult to read in letter/A4 size.
We have enlarged font size where possible.

Minor:
1. Adjust in-figure caption alignments
2. Line 104: add comma RasV12, dlgRNAi
3. Line 114: replace little □ not significant (n.s.)
4. Line 334: 'sogRNAi overexpression' to my knowledge, RNAi are expressed, not overexpressed.
5. Line 454: italicize r4>
6. Fig S4E: remove frame
7. Figures 6: It would be better to number and explain the pathway presented in the figure in text and fig legend.
We have added numbers and explained this in the figure legend.
8. Just a personal preference. Lettering of images in images is commonly done horizontally, here it appears like a mix between vertical and horizontal.
We have changed the lettering when it is possible, due to the small size of the figures, it is not always possible to change the lettering

Reviewer 2:

Major comment

Their genetic experiments clearly showed that the reduction of insulin signaling activity in the fatbody induces upregulation of TGF-β signaling and Collagen accumulation. Then, how does TGF-β signaling induce Collagen accumulation?

We have performed proteomics in the fat body of tumour bearing animals, and compared the proteins differentially expressed in mcherry RNAi vs. mad RNAi. We found by inhibiting TGF-beta signalling, a large number of secretary proteins were upregulated, including exo84, sec16, sec5 etc. So, it is likely that TGF-β signaling affects collagen accumulation by direct regulation of protein secretion in the fat body. This data is included as reviewers only Figure 1 D.

They showed that Rab10 knockdown and SPARC overexpression reduced the accumulation of fatbody ECM. Are Rab10 and SPARC expression regulated by TGF-β signaling?

We have measured Rab10 and SPARC transcription levels when we activated TGF-Beta signalling. This data is included in Figure EV4.

Minor comments

1. Line 90: "Disc Large (Dlg) RNAi in the eye" must be "Discs Large (Dlg1) RNAi in the eye imaginal discs". *We have fixed this error.*

2. Figures 1D and 1L are from the same image. Also, Figures 1C and 1M are from the same image. Are both of them necessary to be shown in the different panels?

The duplication of 1C and 1M, was an error, we thank the reviewer for picking this up. We have fixed this error.

3. Why are the staining patterns of anti-pAkt shown in Figures 1L and 1U so different? pAkt is not detected in the nuclei in Fig. 1L but its nuclear signal is clear in Fig. 1U.

We have shown more representative images of these staining.

4.Figure 1: Images of counter staining for nuclei like DAPI should be also included for all these fatbody images. *We have added a DAPI channel in Figure 1.*

5. Line 101: "Tumour specific ImpL2 inhibition was sufficient to reduce fatbody pAkt levels." Is this correct? ImpL2 inhibition in tumors should elevate the pAKT level in fatbody. *We have fixed this error.*

6. Figure S1~S4: These figures and their legends do not correspond to each other.

We have fixed this error.

7. Line 189: The pAkt level in the muscle of tumour-bearing animals should be examined to confirm the activity of the insulin signaling is downregulated.

We have looked at the status of insulin signalling in the muscle in a separate manuscript, Figure 2 from https://doi.org/10.1101/2023.06.23.546217. Foxo is upregulated in tumour-bearing animals, demonstrating that insulin signalling is downregulated.

8. Line 189: If the authors conclude that muscle insulin signaling predominantly regulates translation and atrophy, OPP assay for the muscle cells should be examined in the same experimental settings.
We have carried out OPP assay upon Akt overexpression in the muscle and have now included this data in Figure EV3.

9. Line 247: The expression level of Rab10 and SPARC should be examined in the fatbody of tumour-bearing animals to see whether Rab10 is upregulated and SPARC is downregulated.
We have examined in the tumour bearing animals whether Rab10 and SPARC levels are changed. We found using a Rab10-GFP, that Rab10 level is elevated. SPARC transcription is not significantly altered (not shown) however, upon examining its localisation, it appears to accumulate in the fatbody (Figure EV4). It has been previously suggested that SPARC plays a role as chaperone protein for Viking (https://www.sciencedirect.com/science/article/pii/S0012160620300683?via%3Dihub), therefore, it is perhaps not surprising that SPARC is also stuck and accumulates in the fatbody. Upon the overexpression of SPARC, via yet unknown mechanisms, is able to override collagen accumulation in the fatbody.

10. Line 247: If Rab10 upregulation and SPARC downregulation are the causes of the accumulation of ECM proteins in the fatbody of tumour-bearing animals, how the overexpressed Collagen proteins can be secreted from the fatbody cells? The overexpression of Collagen is at a higher than normal level, therefore, it is possible that some of it can be processed and secreted despite the general block in secretion. We have carried out an experiment to overexpress Collagen in the muscle of tumour bearing animals, in this case, this manipulation was not able to rescue muscle detachment (data not shown). This indicates that processing of Collagen in the fatbody is important, however, we do not know how the processing is regulated.

11. Line 347: Sog is a secreted BMP antagonist. Thus, it can be expected that the Sog overexpression downregulates TGF-β signaling in fatbody and muscle tissues. If the rescued phenotypes with Sog overexpression can be explained by this logic, pMad level should be examined in these experiments.
We have shown this data in Figure 2.

Reviewer 3:
Major comments:
- Are the key conclusions convincing?
Most of the conclusions are convincing. It is not clear however whether the ECM accumulation in the fat body of tumor animals is fibrotic and whether it is extracellular or in the cell cortex.

- Should the authors qualify some of their claims as preliminary or speculative, or remove them altogether?
-The authors state in line 71 'This deposition of disorganized ECM leads to fibrotic ECM accumulation.' The authors haven't really provided evidence for the ECM being fibrotic. The authors could either rephrase this or provide additional experimental evidence of fibrosis in the fat body.
We have deleted the line on that ECM is fibrotic, although now we have some data supporting that the ECM accumulation is extra-cellular (Figure EV4).

- Would additional experiments be essential to support the claims of the paper? Request additional experiments only where necessary for the paper as it is, and do not ask authors to open new lines of experimentation.
-The authors state in line 147" Finally, in tumor-bearing animals fed a sterol-free diet, that underwent a prolonged 3rd instar stage due to reduced ecdysone levels (Parkin and Burnet, 1986), we activated insulin signalling in the fatbody via Akt overexpression (QRasV12, scribRNAi). We found that this manipulation caused a significant decrease in pMad levels in the fatbody and a rescue of muscle detachment (Figure S1 D-I), similar to animals fed a standard diet (Figure 1 O-Q, Figure 2 F-H)." Since it's not already known what the extent of muscle integrity defect there is in tumors with additional sterol free diet, it would be important to show a non-tumor control for comparison in FigS1F. This would also then make it clear to what extent the defect is rescued by Akt overexpression.
We have included a non-tumour control for Fig S1F.

-The authors state in line 158 'Upon the knockdown of Impl2, we found that tumor gbb was not significantly altered (Figure S3A).' Even though this shows an indication that Gbb levels are not reduced, the n number is too low to state that it is non-significant. The authors should increase the n number here.
N=3 is generally enough to see a difference, we have included data done in parallel which shows Gbb RNAi is sufficient to induce a reduction in Gbb RNA levels (Figure EV2 A). This shows that if there is a reduction in transcript levels, we would have detected it with n=3.

-The authors state in line 171 'Conversely, knockdown of gbb alone or knockdown of gbb together with ImpL2 significantly rescued the Nidogen overaccumulation defects observed at the plasma membrane of fatbody from tumor-bearing animals, while ImpL2RNAi alone did not (Figure S2 Q-U).' This is a somewhat misleading representation, since again no non-tumor control was used, so the extent of the rescue by gbb knowdown is not obvious. In FigS2P Nidogen levels in the tumor seem ~100% higher than in control. But in FigS2U, in which no control was included, the tumor+gbb knowdown seems ~ 20% lower than tumor. So it is probably a more moderate rescue, but that's only possible to assess by including a non-tumor control in FigS2U. Also the images in FigS2Q-T don't seem representative since they appear to show a much bigger difference in fluorescence intensity than ~20%. Please show more representative images.

We have included a non-tumour control for S2Q-T (now EV1 Q-U) and show more representative pictures.

-The authors state in line 174 'Finally, co-knockdown of gbb and ImpL2 in the tumor significantly rescued the reduction in OPP and Nidogen levels observed in the muscles of tumor-bearing animals (Figure S3 B-I).'
*Again, the single knockdowns and the non-tumor control are not shown in Fig**S**3E and I and should be included for comparison and to see the contribution of each knockdown and to be able to judge the extent of the rescue.*
We have included the single knockdowns and a wildtype control, now EV2.

-Regarding Fig3O: Is there a significant tumor muscle attachment defect here? In this graph the tumor only looks about 10% lower than the WT (rather than 40% in Fig2E). The other issue is the extremely low n number for WT. I would recommend increasing the n number for WT here and to indicate in the graph whether the tumor is significantly different to WT (or non-significant, in which case RabRNAi wouldn't actually 'rescue' the defect). In the present form, this graph is not very convincing.
We have increased the n number for WT for this experiment (now Figure 4O), and the WT is significantly different from tumour bearing. The reduction in muscle detachment is 10% rather than 40% here because this experiment was done at day 6, and the 40% reduction in Fig2E (now Figure 3E) was performed at day7. The experiment was carried out at day 6 here, because by day7, the Rab10RNAi rescue is so good, most of the tumour bearing animals have pupated.

- Regarding Fig3W: A non-tumor control would be important to include to be able to judge the extent of muscle attachment defects and the extent of the rescue for UAS-Sparc. This will allow to assess the severity of muscle integrity defect in this particular experiment (since it appears to vary in different experiments e.g. muscle defect in tumor 40% in Fig2E and ~10% in Fig3O) and to assess the extent of rescue for the various genotypes.
We have included a non-tumour control for 3W (now 4W).

-The authors show an accumulation of ECM in the fat body of tumors. It is not clear, whether this ECM accumulates intracellularly near the cell surface or extracellularly. The authors should assess this, maybe by doing electron microscopy.
We do not have an EM facility that can accommodate this experiment, thus doing EM is not an option for us. However, we have tried to address whether the accumulation of ECM is intracellular or extracellular by performing an anti-GFP staining against Viking-GFP without detergent. We show that Viking-GFP is detected without PBST, suggesting the ECM accumulation is extracellular. This data is shown in Figure EV4 A-A'.

- Are the suggested experiments realistic in terms of time and resources? It would help if you could add an estimated cost and time investment for substantial experiments.

-These suggested experiments should be quite straightforward since they are mostly just repeating previous experiments with the appropriate controls and n numbers. I would think that they can be done within a few months. The electron microscopy should not take more than a few weeks and not be costly.

- Are the data and the methods presented in such a way that they can be reproduced?
-The details on how old animals used in each experiment were, are not easy to find and not written very clearly. They should be included in the each figure legend rather than summarising those details in the methods.
We have added the number of days in the figure legend.

-Also, in line 788 in the methods, several stocks are indicated as coming from particular labs (e.g. UAS-FOXO (Kieran Harvey), UAS-GFP (Kieran Harvey), UAS-lacZRNAi (Kieran Harvey), UAS-RasV12 (Helena Richardson), UAS-cg25C;UAS-Vkg (Brian Stramer)).
However, it is not clear whether these labs actually made these stocks and if so whether it has already been described in their papers how the lines were made. If the lines are unpublished, the detailed information should be given on how the lines were made. Or if the lines are published, the authors should provide the reference.
We have fixed these references

- Are the experiments adequately replicated and statistical analysis adequate?
In general, the n number is rather low in several experiments, especially n of 3 for many controls. And as I mentioned before, rescues of tumor phenotypes are often shown without including a non-tumor control, making it hard to judge the extent of the rescue. Sometimes this information can be found in other figures, but the reader should not have to search for it. And also the severity of the phenotype can vary from experiment to experiment.
We have included non-tumour controls as advised by the reviewer

Minor comments:
- Specific experimental issues that are easily addressable.
- Are prior studies referenced appropriately?
Yes, as far as I can tell.

- Are the text and figures clear and accurate?
-In the literature, people usually call it 'fat body' rather than 'fatbody'.
We have fixed this.

-The authors state in line 265 "Vkg accumulated in the membranes of fatbody where p60 was overexpressed using r4-GAL4 (Figure 5 A-C)."
This must be a typo. I think it is shown in Fig5E-G. Unless it's labelled wrongly in the figure and B, C and D show p60 rather than TorDN.
We have corrected this wrong call-out.

-The authors state in line 188 'This manipulation significantly rescued muscle integrity (Figure S4 A-C) and muscle atrophy (Figure S4 D-F), without affecting muscle ECM levels (Figure S4 G-H).' According to the graph in FigS4H this does actually 'affect muscle ECM levels' significantly, as in that it reduced Nidogen levels further. The authors could rephrase this.

We have amended this statement.

Reviewer Figure 1:

Figure for referee with unpublished data and its description has been removed upon request by the authors.

Dear Louise,

Thank you for submitting your revised manuscript. It has now been seen by all of the original referees.

As you can see, the referees find that the study is significantly improved during revision and recommends publication. However, I need you to address the points below before I can accept the manuscript.

• Please address the remaining concern of referee #2. Please also provide a point-by-point response.
• We note that there are currently 5 keywords. However, we can accommodate maximum 5 keywords. Please remove one keyword.
• As per our guidelines, please add a 'Data Availability Section', where you state that no data were deposited in a public database.
• We note some discrepancies between the manuscript submission system and the manuscript in terms of spellings of author names (e.g. Sofia Golenkina in the manuscript vs. Sofya Golenkina in the system; Elizabeth L Christie in the ms vs. Liz Christie in the system).
• Please remove the Author Contributions/CRediT section from the manuscript.
• We note the phrase 'data not shown' in pages 7, 10, 11 and 13, which is not allowed as per our editorial policies. Please either show the data or remove the statements.
• The note that the funding information is not complete in the manuscript submission system - i.e. the Peter MacCallum Cancer Foundation is currently missing
• We note that the Author Checklist is currently missing the information regarding the author name, journal name and the manuscript number (upper left boxes).
• Please make sure that the callouts for the appendix figures have prefix S (e.g. Appendix Figure S1)
• Please rename the 'Methods' section as 'Materials and Methods'.
• During our routine figure checks we noted the possible re-use of cells between 7M and 8A (mCherryRNAi), which is only allowed if the figures are derived from the same experiment, in which case it should be clearly stated in the figure legends.
• Papers published in EMBO Reports include a 'synopsis' and 'bullet points' to further enhance discoverability. Both are displayed on the html version of the paper and are freely accessible to all readers. The synopsis includes a short standfirst summarizing the study in 1 or 2 sentences (max 35 words) that summarize the paper and are provided by the authors and streamlined by the handling editor. I would therefore ask you to include your synopsis blurb and 3-5 bullet points listing the key experimental findings.
• In addition, please provide an image for the synopsis. This image should provide a rapid overview of the question addressed in the study but still needs to be kept fairly modest since the image size cannot exceed 550x400 pixels.
• Our production/data editors have asked you to clarify several points in the figure legends (see attached document). Please incorporate these changes in the attached word document and return it with track changes activated.

Thank you again for giving us to consider your manuscript for EMBO Reports, I look forward to your minor revision.

Kind regards,

Deniz

--
Deniz Senyilmaz Tiebe, PhD
Editor
EMBO Reports

Referee #1:

This paper uses a Drosophila tumor model which is induced by the expression of RasV12+Scrib-IR or RasV12+Dlg-IR in the eye imaginal disc to understand how inter-organ communication affects cachexia in the fat body and muscle. The tumor has previously been shown to secrete the factors ImpL2 and Gbb which decreases insulin signalling and increases TGF-beta signalling in the fat body, respectively, and results in fat body and muscle defects. Here the authors dissect the role of insulin and TGF-beta signalling in the fat body in regulating muscle integrity further. They show that these two pathways converge via Sog in the fat body of WT animals to regulate ECM remodeling. In tumor-bearing animals Sog modulates TGF-beta signaling to regulate ECM accumulation in the fat body which hinders ECM secretion. This then results in the muscle receiving less fat body-derived ECM which causes muscle attachment defects. Interestingly, these muscle defects can be ameliorated by activating insulin signalling or inhibiting TGF-beta signalling or even by increasing ECM secretion in the fat body. The authors also provide some evidence that the insulin and TGF-beta signalling pathways can converge in non-tumor settings.

This revised manuscript seems appropriate for publication at EMBO reports. The authors have addressed the reviewer's comments in a satisfactory manner.

The paper provides important novels insights into the importance of ECM remodeling in the fat tissue and how it relates to cancer cachexia. These new insights on inter-organ communication and how different organs can be affected by tumours and how they can even have downstream effects on other organs other are of great scientific significance and might have important clinical implications. Scientists in the field of ECM, cancer and inter-organ communication will benefit the most from this work, which would also be of interest to some medics in the cancer field.

Referee #2:

In this revised version, the authors addressed the majority of issues raised in the initial version.
For:
- The proper function of RNAi stocks targeting genes like sog, mad, etc. is vital for this study as these lines are used throughout the study. Functional evidence of specific knockdown efficiency should be provided or references given in which these stocks were shown to provide functional knockdown on transcript or protein level.
* We have demonstrated the knockdown efficiency of sogRNAi and madRNAi in Figure EV5.
-- Here, i was asking for a direct assessment of regulation of sog and mad on the respective transcript/protein, not on phosphorylated Mad levels, to show a direct function.

Referee #3:

The newly added experimental data and the corrections improved this manuscript. The proteomics data showing the upregulation of secretary regulators in the fat body of Q-scrib-Ras flies is convincing to support the conclusion. It would be good if the authors could include the proteomics data in the main figures to discuss TGF-β function in collagen accumulation.

Regarding the Rab10 and SPARC expression levels, the authors said the new data is in Figure EV4, but I do not see it. I believe the EV5B is the data the authors mentioned.

I found that all the minor points that I have suggested were appropriately corrected in the revised version.

Referee #2

In this revised version, the authors addressed the majority of issues raised in the initial version.
except For:
- The proper function of RNAi stocks targeting genes like sog, mad, etc. is vital for this study as
these lines are used throughout the study. Functional evidence of specific knockdown efficiency
should be provided or references given in which these stocks were shown to provide functional
knockdown on transcript or protein level.
* We have demonstrated the knockdown efficiency of sogRNAi and madRNAi in Figure EV5.
-- Here, i was asking for a direct assessment of regulation of sog and mad on the respective transcript/protein, not on phosphorylated Mad levels, to show a direct function.

We have now added in EV5 knockdown efficiency of sogRNAi by qPCR and a western blot showing that madRNAi effectively knockdown mad, there is no pMad detected.

Referee #3

The proteomics data showing the upregulation of secretary regulators in the fat body of Q-scrib-Ras flies is convincing to support the conclusion. It would be good if the authors could include the proteomics data in the main figures to discuss TGF-β function in collagen accumulation.

We would prefer not to include this data in the main figure, as it is included in a ms under preparation.

2nd Revision - Editorial Decision 26th Oct 2023

Dear Louise,

Thank you for submitting your revised manuscript. I have now looked at everything and all is fine. Therefore, I am very pleased to accept your manuscript for publication in EMBO Reports.

Congratulations on a nice work!

Kind regards,

Deniz
--
Deniz Senyilmaz Tiebe, PhD
Scientific Editor
EMBO Reports

--

At the end of this email I include important information about how to proceed. Please ensure that you take the time to read the information and complete and return the necessary forms to allow us to publish your manuscript as quickly as possible.

As part of the EMBO publication's Transparent Editorial Process, EMBO reports publishes online a Review Process File to accompany accepted manuscripts. As you are aware, this File will be published in conjunction with your paper and will include the referee reports, your point-by-point response and all pertinent correspondence relating to the manuscript.

If you do NOT want this File to be published, please inform the editorial office within 2 days, if you have not done so already, otherwise the File will be published by default [contact: emboreports@embo.org]. If you do opt out, the Review Process File link will point to the following statement: "No Review Process File is available with this article, as the authors have chosen not to make the review process public in this case."

Thank you again for your contribution to EMBO reports and congratulations on a successful publication. Please consider us again in the future for your most exciting work.

****

THINGS TO DO NOW:

Please note that you will be contacted by Wiley Author Services to complete licensing and payment information. The required 'Page Charges Authorization Form' is available here: https://www.embopress.org/pb-assets/embo-site/er_apc.pdf - please download and complete the form and return to embopressproduction@wiley.com

EMBO Press participates in many Publish and Read agreements that allow authors to publish Open Access with reduced/no publication charges. Check your eligibility: https://authorservices.wiley.com/author-resources/Journal-Authors/open-access/affiliation-policies-payments/index.html

You will receive proofs by e-mail approximately 2-3 weeks after all relevant files have been sent to our Production Office; you should return your corrections within 2 days of receiving the proofs.

Please inform us if there is likely to be any difficulty in reaching you at the above address at that time. Failure to meet our deadlines may result in a delay of publication, or publication without your corrections.

All further communications concerning your paper should quote reference number EMBOR-2023-57695V3 and be addressed to emboreports@wiley.com.

Should you be planning a Press Release on your article, please get in contact with emboreports@wiley.com as early as possible, in order to coordinate publication and release dates.

EMBO Press Author Checklist

Corresponding Author Name: Louise Cheng
Journal Submitted to: EMBO Reports
Manuscript Number: EMBOR-2023-57695V2 | [RC-2023-01974]

USEFUL LINKS FOR COMPLETING THIS FORM
The EMBO Journal - Author Guidelines
EMBO Reports - Author Guidelines
Molecular Systems Biology - Author Guidelines
EMBO Molecular Medicine - Author Guidelines

Reporting Checklist for Life Science Articles (updated January
This checklist is adapted from Materials Design Analysis Reporting (MDAR) Checklist for Authors. MDAR establishes a minimum set of requirements in transparent reporting in the life sciences (see Statement of Task: 10.31222/osf.io/9sm4x). Please follow the journal's guidelines in preparing your
Please note that a copy of this checklist will be published alongside your article.

Abridged guidelines for figures
1. Data
The data shown in figures should satisfy the following conditions:
- the data were obtained and processed according to the field's best practice and are presented to reflect the results of the experiments in an accurate and unbiased manner.
- ideally, figure panels should include only measurements that are directly comparable to each other and obtained with the same assay.
- plots include clearly labeled error bars for independent experiments and sample sizes. Unless justified, error bars should not be shown for technical
- if n<5, the individual data points from each experiment should be plotted. Any statistical test employed should be justified.
- Source Data should be included to report the data underlying figures according to the guidelines set out in the authorship guidelines on Data

2. Captions
Each figure caption should contain the following information, for each panel where they are relevant:
- a specification of the experimental system investigated (eg cell line, species name).
- the assay(s) and method(s) used to carry out the reported observations and measurements.
- an explicit mention of the biological and chemical entity(ies) that are being measured.
- an explicit mention of the biological and chemical entity(ies) that are altered/varied/perturbed in a controlled manner.
- the exact sample size (n) for each experimental group/condition, given as a number, not a range;
- a description of the sample collection allowing the reader to understand whether the samples represent technical or biological replicates (including how many animals, litters, cultures, etc.).
- a statement of how many times the experiment shown was independently replicated in the laboratory.
- definitions of statistical methods and measures:
  - common tests, such as t-test (please specify whether paired vs. unpaired), simple χ2 tests, Wilcoxon and Mann-Whitney tests, can be unambiguously identified by name only, but more complex techniques should be described in the methods section;
  - are tests one-sided or two-sided?
  - are there adjustments for multiple comparisons?
  - exact statistical test results, e.g., P values = x but not P values < x;
  - definition of 'center values' as median or average;
  - definition of error bars as s.d. or s.e.m.

Please complete ALL of the questions below.
Select "Not Applicable" only when the requested information is not relevant for your study.

Materials

Newly Created Materials	Information included in the manuscript?	In which section is the information available? (Reagents and Tools Table, Materials and Methods, Figures, Data Availability Section)
New materials and reagents need to be available; do any restrictions apply?	Not Applicable	

Antibodies	Information included in the manuscript?	In which section is the information available? (Reagents and Tools Table, Materials and Methods, Figures, Data Availability Section)
For **antibodies** provide the following information:<br>- Commercial antibodies: RRID (if possible) or supplier name, catalogue number and or/clone number<br>- Non-commercial: RRID or citation	Yes	methods

DNA and RNA sequences	Information included in the manuscript?	In which section is the information available? (Reagents and Tools Table, Materials and Methods, Figures, Data Availability Section)
**Short novel DNA or RNA including primers, probes**: provide the sequences.	Not Applicable	

Cell materials	Information included in the manuscript?	In which section is the information available? (Reagents and Tools Table, Materials and Methods, Figures, Data Availability Section)
**Cell lines:** Provide species information, strain. Provide accession number in repository **OR** supplier name, catalog number, clone number, and/**OR** RRID.	Not Applicable	
**Primary cultures:** Provide species, strain, sex of origin, genetic modification status.	Not Applicable	
Report if the cell lines were recently **authenticated** (e.g., by STR profiling) and tested for mycoplasma contamination.	Not Applicable	

Experimental animals	Information included in the manuscript?	In which section is the information available? (Reagents and Tools Table, Materials and Methods, Figures, Data Availability Section)
**Laboratory animals or Model organisms:** Provide species, strain, sex, age, genetic modification status. Provide accession number in repository **OR** supplier name, catalog number, clone number, **OR** RRID.	Yes	methods
**Animal observed in or captured from the field:** Provide species, sex, and age where possible.	Not Applicable	
Please detail **housing and husbandry conditions**.	Yes	methods

Plants and microbes	Information included in the manuscript?	In which section is the information available? (Reagents and Tools Table, Materials and Methods, Figures, Data Availability Section)
**Plants:** provide species and strain, ecotype and cultivar where relevant, unique accession number if available, and source (including location for collected wild specimens).	Not Applicable	
**Microbes:** provide species and strain, unique accession number if available, and source.	Not Applicable	

Human research participants	Information included in the manuscript?	In which section is the information available? (Reagents and Tools Table, Materials and Methods, Figures, Data Availability Section)
If collected and within the bounds of privacy constraints report on age, sex and gender or ethnicity for all study participants.	Not Applicable	

Core facilities	Information included in the manuscript?	In which section is the information available? (Reagents and Tools Table, Materials and Methods, Figures, Data Availability Section)
If your work benefited from core facilities, was their service mentioned in the acknowledgments section?	Yes	acknowledgement

Design

Study protocol	Information included in the manuscript?	In which section is the information available? (Reagents and Tools Table, Materials and Methods, Figures, Data Availability Section)

If study protocol has been **pre-registered, provide DOI in the manuscript**. For clinical trials, provide the trial registration number **OR** cite DOI.	Not Applicable	
Report the **clinical trial registration number** (at ClinicalTrials.gov or equivalent), where applicable.	Not Applicable	

Laboratory protocol	Information included in the manuscript?	In which section is the information available? (Reagents and Tools Table, Materials and Methods, Figures, Data Availability Section)
Provide DOI OR other citation details if **external detailed step-by-step protocols** are available.	Not Applicable	

Experimental study design and statistics	Information included in the manuscript?	In which section is the information available? (Reagents and Tools Table, Materials and Methods, Figures, Data Availability Section)
Include a statement about **sample size** estimate even if no statistical methods were used.	Yes	Figures and methods
Were any steps taken to minimize the effects of subjective bias when allocating animals/samples to treatment (e.g. **randomization procedure**)? If yes, have they been described?	Yes	Figures and methods
Include a statement about **blinding** even if no blinding was done.	Not Applicable	
Describe **inclusion/exclusion criteria** if samples or animals were excluded from the analysis. Were the criteria pre-established?<br><br>If sample or data points were omitted from analysis, report if this was due to attrition or intentional exclusion and provide justification.	Not Applicable	
For every figure, are **statistical tests** justified as appropriate? Do the data meet the assumptions of the tests (e.g., normal distribution)? Describe any methods used to assess it. Is there an estimate of variation within each group of data? Is the variance similar between the groups that are being statistically compared?	Yes	Figures and methods

Sample definition and in-laboratory replication	Information included in the manuscript?	In which section is the information available? (Reagents and Tools Table, Materials and Methods, Figures, Data Availability Section)
In the figure legends: state number of times the experiment was **replicated** in laboratory.	Yes	Figures
In the figure legends: define whether data describe **technical or biological replicates**.	Yes	methods

Ethics

Ethics	Information included in the manuscript?	In which section is the information available? (Reagents and Tools Table, Materials and Methods, Figures, Data Availability Section)
Studies involving **human participants**: State details of **authority granting ethics approval** (IRB or equivalent committee(s), provide reference number for approval.	Not Applicable	
Studies involving **human participants**: Include a statement confirming that **informed consent** was obtained from all subjects and that the experiments conformed to the principles set out in the WMA Declaration of Helsinki and the Department of Health and Human Services Belmont Report.	Not Applicable	
Studies involving **human participants:** For publication of **patient photos**, include a statement confirming that consent to publish was obtained.	Not Applicable	
Studies involving experimental **animals**: State details of **authority granting ethics approval** (IRB or equivalent committee(s), provide reference number for approval. Include a statement of compliance with ethical regulations.	Not Applicable	
Studies involving **specimen and field samples:** State if relevant **permits** obtained, provide details of authority approving study; if none were required, explain why.	Not Applicable	

Dual Use Research of Concern (DURC)	Information included in the manuscript?	In which section is the information available? (Reagents and Tools Table, Materials and Methods, Figures, Data Availability Section)
Could your study fall under dual use research restrictions? Please check biosecurity documents and list of **select agents and toxins** (CDC): https://www.selectagents.gov/sat/list.htm	Not Applicable	
If you used a select agent, is the security level of the lab appropriate and reported in the manuscript?	Not Applicable	
If a study is subject to dual use research of concern regulations, is the name of the **authority granting approval and reference number** for the regulatory approval provided in the manuscript?	Not Applicable	

Reporting

The MDAR framework recommends adoption of discipline-specific guidelines, established and endorsed through community initiatives. Journals have their own policy about requiring specific guidelines and recommendations to complement MDAR.

Adherence to community standards	Information included in the manuscript?	In which section is the information available? (Reagents and Tools Table, Materials and Methods, Figures, Data Availability Section)
State if relevant guidelines or checklists (e.g., **ICMJE, MIBBI, ARRIVE, PRISMA**) have been followed or provided.	Not Applicable	
For **tumor marker prognostic studies,** we recommend that you follow the **REMARK** reporting guidelines (see link list at top right). See author guidelines, under 'Reporting Guidelines'. Please confirm you have followed these guidelines.	Not Applicable	
For **phase II and III randomized controlled trials**, please refer to the **CONSORT** flow diagram (see link list at top right) and submit the CONSORT checklist (see link list at top right) with your submission. See author guidelines, under 'Reporting Guidelines'. Please confirm you have submitted this list.	Not Applicable	

Data Availability

Data availability	Information included in the manuscript?	In which section is the information available? (Reagents and Tools Table, Materials and Methods, Figures, Data Availability Section)
Have **primary datasets** been deposited according to the journal's guidelines (see 'Data Deposition' section) and the respective accession numbers provided in the Data Availability Section?	Not Applicable	
Were **human clinical and genomic datasets** deposited in a public access-controlled repository in accordance to ethical obligations to the patients and to the applicable consent agreement?	Not Applicable	
Are **computational models** that are central and integral to a study available without restrictions in a machine-readable form? Were the relevant accession numbers or links provided?	Not Applicable	
If publicly available data were reused, provide the respective **data citations in the reference list**.	Not Applicable	
